# New Benzofuran Oligomers from the Roots of *Eupatorium heterophyllum* Collected in China

**DOI:** 10.3390/molecules27248856

**Published:** 2022-12-13

**Authors:** Yiming Hu, Yoshinori Saito, Yosuke Matsuo, Xun Gong, Takashi Tanaka

**Affiliations:** 1 Graduate School of Biomedical Sciences, Nagasaki University, 1-14 Bunkyo-machi, Nagasaki 852-8521, Japan; 2 Kunming Institute of Botany, Chinese Academy of Sciences, Kunming 650201, China

**Keywords:** *Eupatorium heterophyllum*, Asteraceae, benzofuran oligomer, structure elucidation, Hengduan mountain

## Abstract

The chemical constituents of two root samples of *Eupatorium heterophyllum* DC. collected in Yunnan Province, China, were investigated. Five new oligomeric benzofurans (**1**–**5**), nine new benzofuran/dihydrobenzofuran derivatives, and a new thymol analog were isolated, and their structures were determined using extensive spectroscopic techniques, such as 1D and 2D NMR spectroscopy and DFT calculations of the CD spectra. Most of the new compounds, including oligomeric benzofurans (**1**–**5**), were obtained from only one of the root samples. Furthermore, this is the first example that produces oligomeric benzofurans in this plant. These results imply that diversification of secondary metabolites in *E. heterophyllum* is ongoing. Plausible biosynthetic pathways for **1**–**5** are also proposed.

## 1. Introduction

Plants in the Hengduan mountains area are useful for studying plant diversity and evolution. We have been studying inter- and intra-specific diversity in chemical constituents of some Asteraceae plants growing in this area, including *Ligularia* [1,2], *Cremanthodium* [3], *Saussurea* [4,5], and *Eupatorium* [6,7,8,9]. To date, the presence of intra-specific diversity has been discovered at various levels in most species, affording numerous new compounds produced by limited populations within a species.

*Eupatorium heterophyllum* DC. (Asteraceae) is a perennial herb widely distributed in the Hengduan Mountains, including in the Yunnan, Sichuan, and Gansu provinces and the Xizang Autonomous Region of China [10]. Only our group has previously reported detailed phytochemical studies on this plant [6,7,8,9]. Sesquiterpene lactones were isolated as major constituents from several samples of the aerial parts of *E. heterophyllum* collected in northwestern Yunnan and southwestern Sichuan [7], and benzofurans were isolated from the roots of this species in northwestern Yunnan [6] and northern Sichuan [8]. The characteristics of the chemical compositions are similar to those of *E. chinense* [11,12,13], suggesting that *E. heterophyllum* and *E. chinense* are related chemotaxonomically. Intra-specific diversity in the root chemicals of *E. heterophyllum* was observed in minor constituents, and a variety of benzofuran/dihydrobenzofuran derivatives, propynyl thiophenes, acetylenic compounds, and oxygenated thymol were obtained. Benzofurans are a significant group of heterocyclic compounds with wide ranges of biological activities [14], such as antibacterial [15], antifungal [16,17], and antifeedant activities [18,19]. These findings prompted us to conduct additional phytochemical studies on this plant growing in other locations. In this study, two additional root samples of *E. heterophyllum* were collected at different locations in Yunnan Province of China as part of our ongoing research into the chemical diversity of the genus *Eupatorium*. Fifteen new compounds, including five benzofuran oligomers (**1**–**5**), were isolated from the MeOH extract of the roots of the collected samples. Herein, we report the isolation and structural elucidation of these compounds. Plausible biosynthetic pathways for **1**–**5** are also proposed.

## 2. Results and Discussion

### 2.1. Samples

Two *E. heterophyllum* samples were collected in Lanping County (sample 1) and Lijiang City (sample 2) of Yunnan Province. These sampling locations were approximately 50–100 km southeast from those of our previous Yunnan samples [6]. The dried roots of each sample were cut into pieces and extracted with MeOH, and the compounds were separated using silica-gel column chromatography and normal-phase HPLC to yield 49 compounds, 15 of which were new. The structures of the new compounds (**1**–**5**, **14**, **17**, **21**, **25**, **26**, **31**, **32**, **34**, **36**, and **39**) were elucidated as follows.

### 2.2. Structure Elucidation

**Figure 1:**
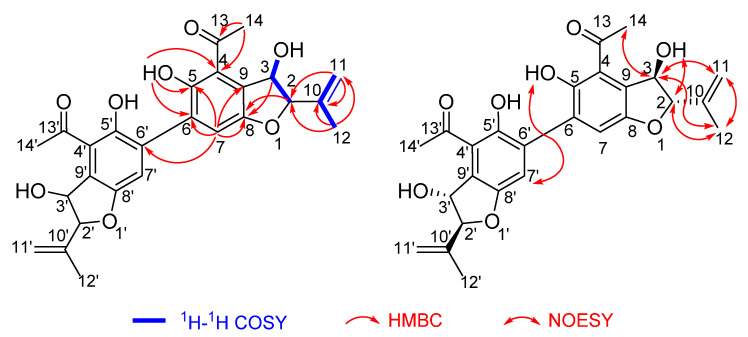
Key 2D NMR correlations for **1**.

Compound **1** was obtained as yellow amorphous powder. The [M+Na]^+^ peak at *m*/*z* 489.1527 in HRFABMS revealed that its molecular formula is C_26_H_26_O_8_. The presence of hydroxy (3416 cm^−1^) and conjugated carbonyl (1651 cm^−1^) groups suggested using the IR spectrum. The ^1^H NMR spectrum (Table 1) revealed resonances attributable to two hydroxy groups [δ_H_ 11.72 (1H, s) and 2.84 (1H, br s)], an aromatic proton [δ_H_ 7.14 (1H, s)], two oxymethines [δ_H_ 5.34 and 5.00 (each 1H, s)], an exo-methylene [δ_H_ 5.10 and 4.94 (each 1H, s)], and two methyls [δ_H_ 2.88 and 1.79 (each 3H, s)]. These spectroscopic features were very similar to those of **29** [8], except for the appearance of a singlet aromatic proton signal and the disappearance of a pair of *ortho*-coupled aromatic proton signals in the ^1^H NMR spectrum of **1**. Only 13 carbon resonances, including a carbonyl, three methines, a methylene, two methyls, and six quaternary carbons (Table 1), were detected in the ^13^C NMR and HSQC spectra that **1** exhibited. Moreover, **1** exhibited adequate negative optical rotation ([α${]}_{D}^{11}$ −23.9). These observations and the molecular formula of **1** suggest that **1** is a homodimer of **29** with a symmetrical structure, in which the benzene rings of each monomeric unit are directly connected. The structure of the monomeric unit and its connection to another unit via C-6 and C-6’ was established from the ^1^H ^1^H COSY and HMBC correlations shown in Figure 1, which were further supported by the downfield shift of C-6 to δ_C_ 130.3 and the NOESY correlation between 5-OH and H-7’. The relative stereochemistry of **1** was proposed to be 2,3-*trans*, based on the small *J*_H-2-H-3_ (br s) observed [20,21], as well as the NOE correlation between H-2 and 3-OH; H-3 and H-11/H_3_-12. Therefore, the relative structure of compound **1** was established, as shown in Figure 1.

Compound **2** was obtained as yellow amorphous powder. The same molecular formula of C_26_H_26_O_8_ as compound **1** was determined using HRFABMS and ^13^C NMR data. The IR spectrum indicated the presence of hydroxy (3411 cm^−1^) and conjugated carbonyl (1656 cm^−1^) groups. The UV spectrum showed maximum absorptions at 222 and 337 nm.

**Figure 2:**
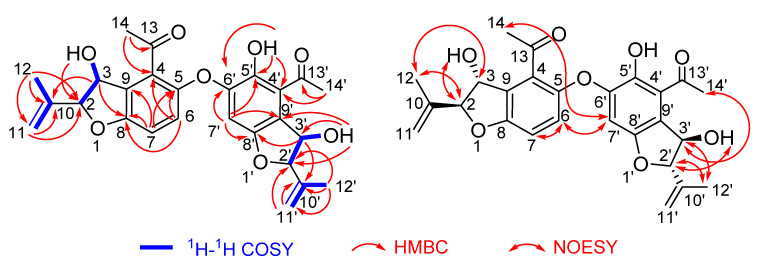
Key 2D NMR correlations for **2**.

The ^1^ H NMR spectrum (Table 1) revealed three hydroxy groups [δ_H_ 10.49 (1H, s), 4.37 (1H, br s), and 2.93 (1H, d, 7.1 Hz)], three aromatic protons [δ_H_ 7.03 (1H, d, 8.8 Hz), 6.91 (1H, d, 8.8 Hz), and 6.66 (1H, s)], four oxymethines [δ_H_ 5.29 (1H, dd, 7.1, 2.6 Hz), 5.21 (1H, br s), 5.04 (1H, s), and 4.95 (1H, br s)], two exo-methylenes [δ_H_ 5.10, 5.04, 4.92 and 4.92 (each 1H, s)], and four methyls [δ_H_ 2.86, 2.69, 1.78 and 1.75 (each 3H, s)]. The ^13^C NMR spectrum of compound **2** revealed twenty-six carbon signals, including two carbonyls, seven methines, two methylene, four methyls, and eleven quaternary carbons (Table 1). These observations, along with the ^1^H ^1^H COSY and HMBC correlations (Figure 2), indicated that **2** is composed of two 2,3-dihydrobenzofuran moieties, similar to **29** [8]. The connection of the two benzofuran moieties via an ether bond between C-5 and C-6’ in **2** was confirmed by the disappearance of the 5-OH signal in the ^1^H NMR spectrum and the NOESY correlations between H-7’ and H-6/H_3_-14. Based on a similar consideration as **1**, the relative stereochemistry of C-2/C-3 and C-2’/C-3’ was discovered to be *trans*. Therefore, the relative structure of compound **2** was determined as depicted in Figure 2.

**Figure 3:**
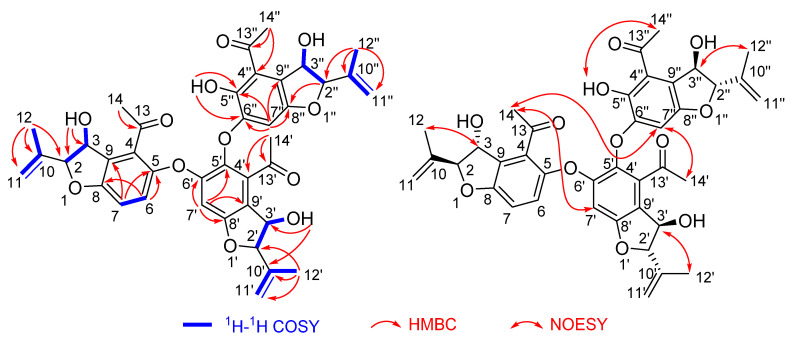
Key 2D NMR correlations for **3**.

Compound **3** was obtained as yellow amorphous powder, and its molecular formula, C_39_H_38_O_12_, was calculated from the [M + Na]^+^ ion peak observed at *m*/*z* 721.2260 in HR-FABMS. The ^1^H and ^13^C NMR spectra of **3** (Table 1) revealed signals attributable to the three 2,3-dihydrobenzofuran moieties, indicating that **3** is a trimeric benzofuran derivative. The comparison of the NMR data of **3** with those of **2** implied that **3** shares a common structure with **2** and is connected to another 2,3-dihydrobenzofuran unit (Figure 3). The linkages of the benzofuran units via oxygen atoms between C-5 and C-6’ and between C-5’ and C-6” were determined by NOESY correlations between H_3_-14 and H-7’/H-7”; H_3_-14’ and H-7”. Therefore, the relative structure of compound **3** was determined, as shown in Figure 3.

**Figure 4:**
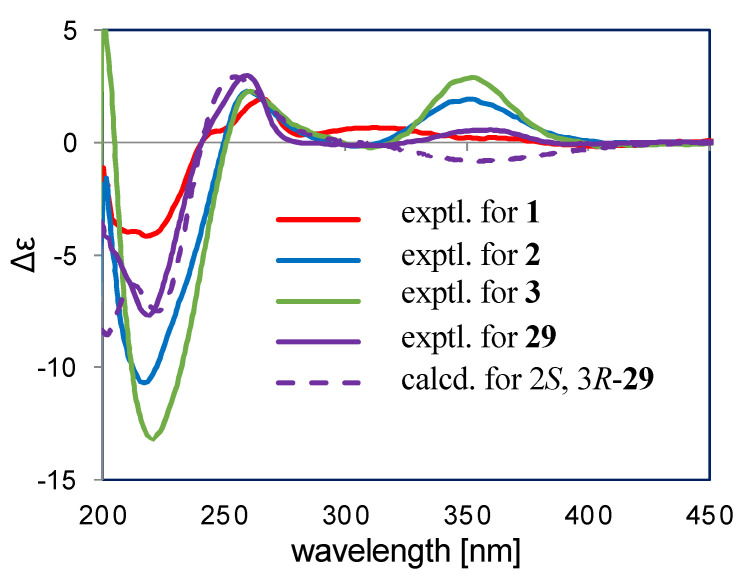
Experimental ECD spectra of **1**–**3** and **29** and calculated ECD spectrum of 2*S*,3*R*-**29**.

Because compounds **1**–**3** were considered oligomers of **29**, their absolute configurations were determined by comparing their ECD spectra to that of **29** [8]. As shown in Figure 4, the experimental ECD spectra of **1**–**3** and **29** are in good agreement with the theoretical ECD spectrum of (2*S*,3*R*)-**29**. Therefore, the absolute configurations of **1**–**3** were established to be (2*S*,3*R*,2’*S*,3’*R*)-**1**, (2*S*,3*R*,2’*S*,3’*R*)-**2**, and (2*S*,3*R*,2’*S*,3’*R*,2”*S*,3”*R*)-**3**, respectively. The calculated ECD spectra of (2*R*,3*R*)-**29** and each optimized conformer of (2*S*,3*R*)-**29** are shown in Supplementary Materials: Figures S19 and S20.

**Figure 5:**
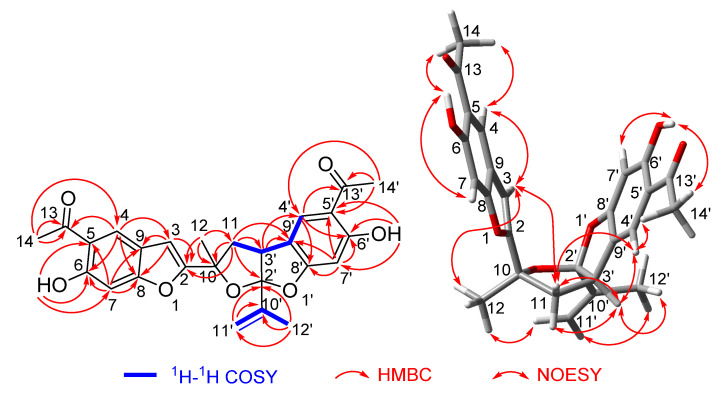
Key 2D NMR correlations for **4**.

Compound **4** was obtained as yellow amorphous powder. Its molecular formula was determined to be C_26_H_24_O_7_ by the [M + H]^+^ peak at *m*/*z* 449.1600 in its HRFABMS, indicating 15 degrees of unsaturation. Its ^1^H and ^13^C NMR data (Table 2), as well as the HMBC correlations (Figure 5) from H-4 to C-3/C-6/C-8/C-13; 6-OH to C-5/C-6/C-7; H-4’ to C-6’/C-8’/C-13’, and 6’-OH to C-5’/C-6’/C-7’, suggested that **4** consists of two 5-acetyl-6-hydroxybenzofuran units, such as euparin (**15**) [22,23], one of the major constituents of this plant [9]. Furthermore, the ^1^H ^1^H COSY cross-peak between H_2_-11/H-3’, HMBC correlations from H_2_-11 to C-2/C-10/C-2’/C-3’/C-9’, and the molecular formula of **4** indicated that these benzofuran units are linked by a tetrahydrofuran ring composed of C-10 (δ_C_ 84.6), C-11 (δ_C_ 43.1), C-3’ (δ_C_ 49.8), C-2’ (δ_C_ 122.8), and oxygen between C-10 and C-2’. The NOESY correlations of H-3’/H_3_-12’, H_3_-12/H-11’a, H_3_-12’/H-11’b, H-11a/H-3, and H-11a/H-4’ and the coupling constants of H-3’ [δ_H_ 3.87 (1H, d, 8.8 Hz)], H-11a [δ_H_ 2.84 (1H, dd, 13.2, 1.2 Hz)], and H-11b [δ_H_ 2.45 (1H, dd, 13.2, 8.8 Hz)] established the relative configurations as (10*S* *,2’*S* *,3’*R* *)-**4** (Figure 5).

Compound **5** was obtained as yellow amorphous powder. The [M + H]^+^ peak at *m*/*z* 461.1601 in HRFABMS determined its molecular formula to be C_27_H_24_O_7_, indicating 16 degrees of unsaturation. The IR spectrum revealed the presence of hydroxy (3245 cm^−1^) and conjugated carbonyl groups (1672 cm^−1^), as well as an acetylenic moiety (2225 cm^−1^).

**Figure 6:**
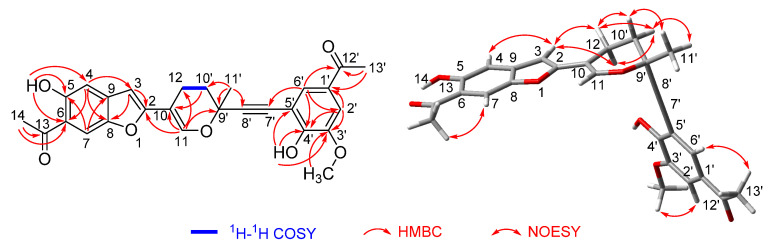
Key 2D NMR correlations for **5**.

The ^1^H NMR spectrum of **5** exhibited signals derived from two hydroxy groups [δ_H_ 12.26 and 6.28 (each 1H, s)], six aromatic/olefinic protons [δ_H_ 7.70 (1H, s), 7.58 (1H, d, 1.9 Hz), 7.47 (1H, d, 1.9 Hz), 7.37 (1H, s), 6.94 (1H, s), and 6.35 (1H, s)], two methylenes [δ_H_ 2.82 (1H, dddd, 16.2, 11.2, 6.0, 1.8 Hz), 2.44 (1H, dddd, 16.2, 5.4, 2.6, 1.0 Hz), 2.29 (1H, ddd, 13.5, 6.0, 2.6 Hz), and 2.02 (1H, ddd, 13.5, 11.2, 5.4 Hz)], a methoxy group [δ_H_ 3.92 (3H, s)], and three methyls [δ_H_ 2.66, 2.53, and 1.79 (each 3H, s)] (Table 2). The ^13^C NMR spectrum revealed 27 carbon signals, including two carbonyls, six methines, two methylenes, four methyls, and thirteen quaternary carbons (Table 2). These observations indicated that the structure of **5** is composed of benzofuran and acetylenic moieties, such as **27** [24] and **37** [6], respectively. The substitution pattern of the benzene rings in **5** were determined from the HMBC correlations shown in Figure 6. Moreover, the ^1^H ^1^H COSY correlation between H_2_-12 and H_2_-10’, as well as the HMBC correlations from H_2_-10’ to C-10 and from H-11 to C-9’, revealed the presence of a dihydropyran ring linking the benzofuran and acetylenic parts. The conformation of the dihydropyran ring was established by the NOE correlations shown in Figure 6.

Compounds **4** and **5** exhibited significantly weaker Cotton effects in their experimental ECD spectra than the calculated spectra of (10*S*,2’*S*,3’*R*)-**4** and (9’*R*)-**5** (Figure S33a), respectively, indicating that they are racemates. To confirm this, chiral HPLC analyses of **4** and **5** were performed, resulting in the detection of enantiomers in a ratio of approximately 1:1 for **4** and **5** (Figure S33b).

**Figure 7:**
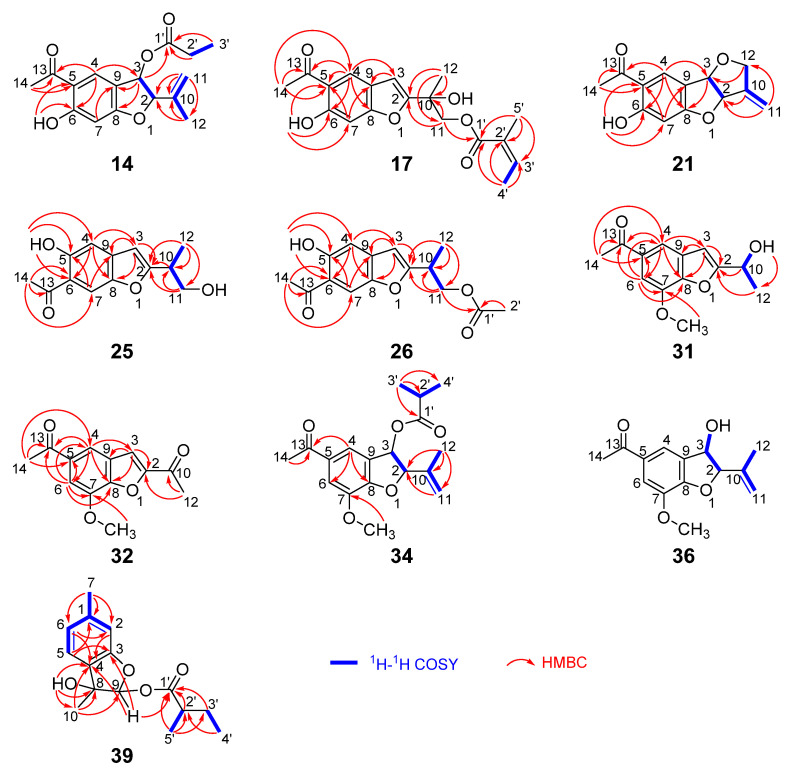
Key ^1^H ^1^H COSY and HMBC correlations for 10 new compounds.

**Figure 8:**
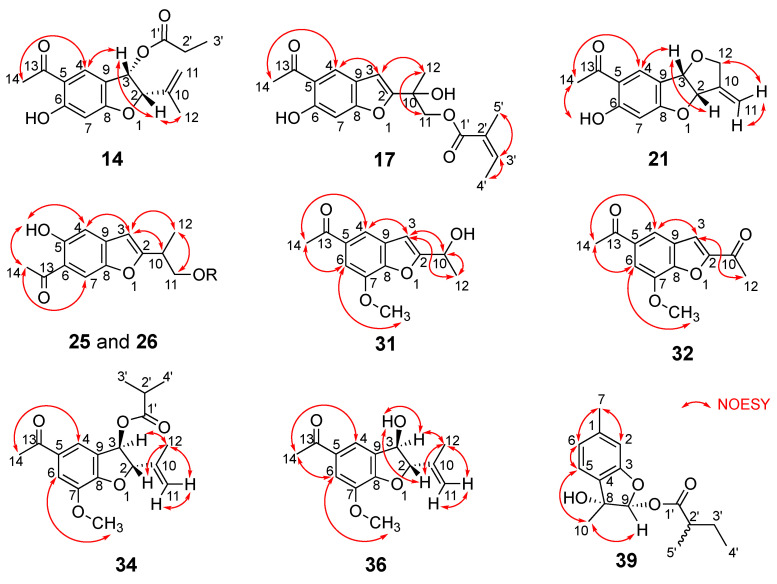
Key NOESY correlations for 10 new compounds.

Compound **14** was obtained as colorless amorphous powder. Based on the [M+H]^+^ peak at *m*/*z* 291.1231 in its HRFABMS, its molecular formula was determined to be C_16_H_18_O_5_. The ^1^H and ^13^C NMR data (Table 3) were similar to those of known compound **13 [25]**; however, the appearance of signals attributed to a propionyl group [δ_H_ 2.31 (2H, q, 7.6 Hz), 1.12 (1H, t, 7.6 Hz); δ_C_ 174.1, 27.7, 9.0] in the NMR spectrum of **14** instead of those attributed to an isobutanoyl group in **13** suggested that **14** was a 3-propionyloxy analog of **13** (Figure 7). The 2,3-*cis* nature was indicated by *J*_2,3_ (6.4 Hz) [20,21] and the NOE correlation between H-2 and H-3 (Figure 8). Therefore, the structure of compound **14** was determined as shown in Figure 8.

Compound **17** was obtained as yellowish amorphous powder. Its molecular formula was determined to be C_18_H_20_O_6_ by the [M + Na]^+^ peak at *m*/*z* 355.1160 in its HRFABMS. The ^1^H and ^13^C NMR data of **17** (Table 3) were similar to those of known compound **19 [26]**, except for the presence of additional signals assignable to an angeloyloxy group [δ_H_ 6.09 (1H, qq, 7.3, 1.4 Hz), 1.89 (3H, dq, 7.3, 1.4 Hz), 1.83 (3H, quint, 1.4); δ_C_ 167.9, 127.1, 139.5, 15.8, 20.5]. The downfield shift of H_2_-11 [δ_H_ 4.53 (1H, d, 11.5 Hz), 4.38 (1H, d, 11.5 Hz)] was also observed in the ^1^H NMR spectrum of **17**. Therefore, **17** was determined to be an 11-*O*-angeloyl derivative of compound **19**, as supported by the HMBC correlations shown in Figure 7, particularly from H_2_-11 to C-1’.

Compound **21** was obtained as yellow amorphous powder. Its molecular formula was determined to be C_13_H_12_O_4_ based on the [M + H]^+^ peak at *m*/*z* 233.0814 in its HRFABMS. Careful comparison of the ^1^H NMR data of **21** (Table 3) with those of platypodantherone [27] revealed that **21** was a 6-*O*-demethyl derivative of platypodantherone because of the absence of a methoxy signal and the appearance of a hydrogen-bonded phenolic hydroxy signal (δ_H_ 12.99). This structure was confirmed by the HMBC from H_2_-12 to C-3 and other 2D NMR correlations (Figure 7).

The molecular formulae of compounds **25** and **26** were determined to be C_13_H_14_O_4_ and C_15_H_16_O_5_, respectively, using HRFABMS. The structural similarity of compound **25** to that of 11-hydroxy-10,11-dihydro-euparin [28] was deduced from the ^1^H NMR data (Table 4); however, the resonance of an aromatic proton (δ_H_ 7.78) was slightly downfield-shifted in **25** as compared to that of 11-hydroxy-10,11-dihydro-euparin. The HMBC and NOESY correlations (Figure 7 and Figure 8) revealed compound **25** as a 6-acetyl-5-hydroxy-isomer of 11-hydroxy-10,11-dihydro-euparin. The ^1^H and ^13^C NMR spectra of **26** were distinguished from those of **25** by the presence of acetate-derived signals [δ_H_ 2.05 (3H, s); δ_C_ 170.9, 20.9] and the downfield-shift of H_2_-11 [δ_H_ 4.36 (1H, dd, 11.0, 6.8 Hz), 4.27 (1H, dd, 11.0, 6.3 Hz)] (Table 4). Therefore, **26** was identified as an 11-*O*-acetyl derivative of **25**, which was further supported by the HMBC correlation from H_2_-11 to C-1’ (Figure 7).

Compounds **31** and **32** were obtained as yellow amorphous powders. From the [M + H]^+^ peaks at *m*/*z* 235.0970 and 233.0814 in their HRFABMS, their molecular formulae were determined to be C_13_H_14_O_4_ and C_13_H_12_O_4_, respectively. The presence of the 5-acetyl-7-methoxybenzofuran core in the structures of **31** and **32** was deduced from their ^1^H and ^13^C NMR spectra (Table 4) and HMBC correlations (Figure 7). Furthermore, the ^1^H ^1^H COSY correlations between H_3_-12/H-10/10-OH in **31** and the HMBC correlations from H_3_-12 to C-2/C-10 in **31** and **32** revealed that a 1-hydroxyethyl group was substituted at C-2 in **31**, whereas an acetyl group was substituted in **32**. Therefore, the structures of **31** and **32** were established, as shown in Figure 8.

The molecular formulae of compounds **34** and **36** were determined to be C_18_H_22_O_5_ and C_14_H_16_O_4_, respectively, using HRFABMS. Their ^1^H and ^13^C NMR data (Table 5) suggested structural similarities to the known compound **33 [6]**; however, the signals derived from an angeloyloxy group in **33** were replaced by those of an isobutanoyloxy group [δ_H_ 2.57 (sept, *J* = 7.1 Hz), 1.20 (d, *J* = 7.1 Hz), 1.17 (d, *J* = 7.1 Hz); δ_C_ 176.8, 33.9, 18.9, 18.8] in **34** and a hydroxy group [δ_H_ 2.16 (1H, d, 7.8)] in **36**. The 2,3-*trans* nature was indicated by *J*_2,3_ (2.8 Hz in **34** and 4.0 Hz in **36**), which was the same value as *J*_2β,3α_ in **33 [6]** and 7-hydroxytoxol [29,30], respectively. This stereochemistry was supported using the NOESY correlations between H-3 and H_3_-12 (Figure 8).

Compound **39** was obtained as white amorphous powder. Its molecular formula was determined to be C_15_H_20_O_4_ by the quasi-molecular ion at *m*/*z* 247.1334 [M-H_2_O+H]^+^ in its HRFABMS. The ^1^H and ^13^C NMR spectra of **39** were closely related to those of 3,9β-epoxy-9α-isobutanoyloxymentha-1,3,5-trien-8a-ol, a recently reported thymol derivative [8], except for the disappearance of the signals attributable to an isobutanoyloxy group substituted at C-9 and the appearance of those of a 2-methylbutanoyloxy group (Table 5). The relative configuration of the furan moiety was determined by the NOESY correlation between H-9 and H_3_-10 (Figure 8). A detailed analysis of the ^1^H and ^13^C NMR spectra of **39** revealed that it was a mixture of C-2’ epimers (*ca*. 3:1 based on the integration of ^1^H NMR signals).

**Figure 9:**
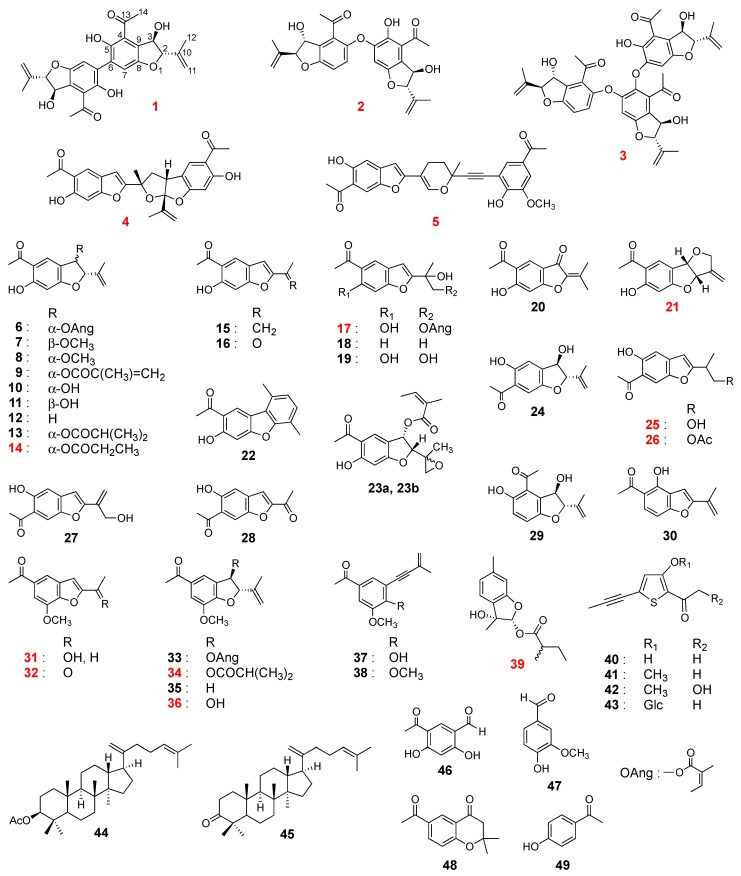
Compounds isolated from the roots of *E. heterophyllum* (new compounds shown in red).

The structures of 34 known compounds, including 24 benzofurans (**6** [23], **7** [31], **8** [8], **9** [25], **10** [32], **11** [29], **12** [33], **13** [25], **15** [22,23], **16** [34], **18** [35], **19** [26], **20** [36], **22** [15,37], **23** [8], **24** [8], **27** [24], **28** [38], **29** [8], **30** [39], **33** [6], **35** [40], **37** [6], and **38** [6]), 4 thiophenes (**40** [41], **41** [41], **42** [8], **43** [6]), 2 triterpenoids (**44** [42] and **45** [43]), and 4 other aromatic compounds (**46** [44], **47** [45], **48** [46], **49** [47]) were identified by comparing their NMR data with those reported in the literature (Figure 9).

### 2.3. Discussion

**Scheme 1:**
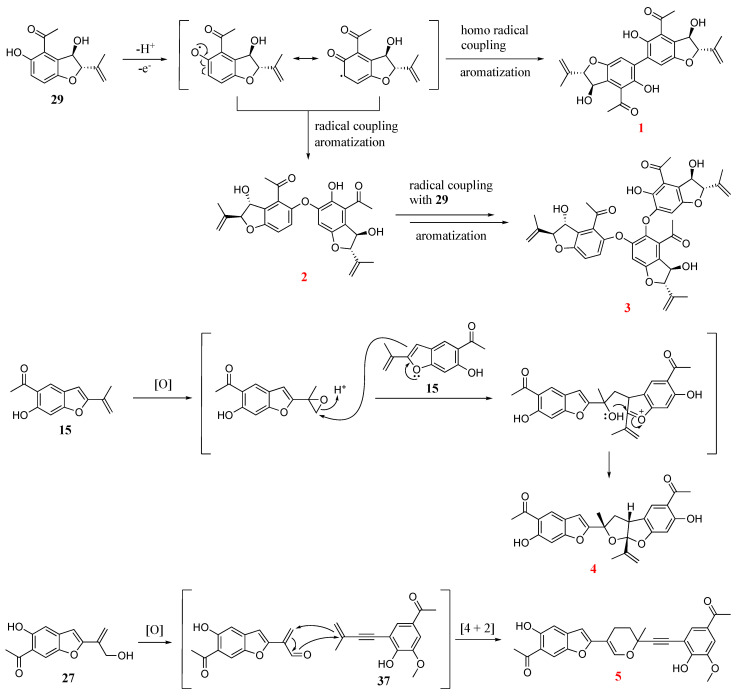
Plausible biogenetic pathway for **1**–**5**.

Plausible biosynthetic pathways for the new benzofuran oligomers (**1**–**5**) are depicted in Scheme 1. Compound **1** can be a homocoupling product of two *ortho*-radicals generated by one-electron oxidation of known compound **29**. Similarly, compound **2** is likely to be formed by the radical coupling of **29**-derived phenoxy- and *ortho*-radicals, and subsequent radical coupling of **29** with **2** will afford trimer **3**. Compound **4** can be produced by the nucleophilic attack of euparin (**15**) on an epoxide, derived from another molecule of **15**, via ring-opening of the epoxide, followed by the construction of another furan ring. Compound **5** will be yielded via a [4 + 2] cycloaddition reaction between **27**-derived aldehyde and **37**.

In this study, 49 compounds, including 15 new compounds, were isolated from 2 root samples of *E. heterophyllum* collected in Yunnan Province (Figure 9). The major constituents of both samples were benzofuran/dihydrobenzofuran derivatives, such as **6** and **15**. Sample 2 also contained a significant amount of thiophenes (e.g., **43**). These characteristics of chemical composition were very similar to those of our previous *E. heterophyllum* samples collected in Yunnan and Sichuan provinces [6,8]. However, it is worth noting that sample 2 is the only sample to date that produces oligomeric benzofurans: two dimeric benzofuran diastereomers [9]. Compounds **1**–**5** were obtained from this sample, but not from sample 1 or other previous samples [6,8], implying that the diversification of secondary metabolites in *E. heterophyllum* is ongoing.

*Eupatorium heterophyllum* is generally regarded as a synonym of *E. mairei* [10]. In contrast, Kawahara et al. have proposed that *E. heterophyllum* is distinguished from *E. mairei* and may be a hybrid originating from *E. mairei* and *E. chinense* [48]. As described above, the chemical compositions of *E. heterophyllum* are similar to those of *E. chinense* [11,12,13]. Moreover, some research groups have recently reported the isolation of various oligomeric and related benzofuran compounds from *E. chinense* of various origins [49,50,51,52,53]. These findings indicate a close relationship between *E. heterophyllum* and *E. chinense*, which would lend support to Kawahara’s theory. Further chemical studies on *E. heterophyllum* collected from other regions are in progress.

## 3. Materials and Methods

### 3.1. General Experimental Procedures

A JASCO P1020 NK digital polarimeter was used to measure the optical rotations. The diffuse reflectance method was used to record IR spectra on a JASCO FT/IR-410 spectrophotometer. UV spectra were obtained on a JASCO V-560 UV/Vis spectrophotometer. ECD spectra were measured on a JASCO J-725N spectrophotometer. ^1^H and ^13^C NMR spectra were recorded on a JEOL JNM-AL 400 (^1^H: 400 MHz) or Varian Unity *plus* 500 (^1^H: 500 MHz, ^13^C: 126 MHz, respectively) spectrometer using CDCl_3_. Chemical shift values are given in δ (ppm), using the solvent peak signals (CDCl_3_: TMS) as references, and coupling constants (*J*) are reported in Hz. A JEOL JMS-700 MStation was used to record mass spectra, including high-resolution spectra. Column chromatography was performed on Silica gel 60 (100–210 mesh, Kanto Chemical Co., Inc., Tokyo, Japan). Preparative HPLC was performed on a JASCO chromatograph (*n*-hexane–EtOAc, CHCl_3_–EtOAc) equipped with a JASCO PU-2086 pump, a JASCO UV-970 detector, a JASCO RI-2031 detector, and various columns: COSMOSIL 5SL-II (10 × 250 mm, Nacalai Tesque Inc., Kyoto, Japan), COSMOSIL 5SL-II (4.6 × 250 mm, Nacalai Tesque Inc., Kyoto, Japan), YMC-Pack Diol-120-NP (4.6 × 250 mm, YMC Co., Ltd., Kyoto, Japan), and Inertsil CN-3 (4.6 × 250 mm, GL Sciences, Tokyo, Japan).

### 3.2. Plant Materials

Samples were collected in Yunnan Province of P.R. China in August 2014 and authenticated by Dr. Takayuki Kawahara, Forestry and Forest Products Research Institute (Japan Forest Technology Association, General Incorporated Association in present affiliation). Sample 1 was collected in Lanping Bai and Pumi Autonomous County, and sample 2 was collected in Gucheng District, Lijiang City, approximately 40 km distant from one another. The voucher specimen numbers for samples 1 and 2 were 2014-10 and 2014-48, respectively. These were deposited in Kunming Institute of Botany, Kunming, China.

### 3.3. Extraction and Isolation

The dried roots of sample 2 (48.1 g) were cut into small pieces and extracted twice with MeOH at room temperature. After removal of the solvent under reduced pressure not exceeding 30 °C, a concentrated and combined MeOH extract (7.5 g) was obtained, which was separated on a silica gel column (*n*-hexane–EtOAc, 1:0, 99:1, 98:2, 95:5, 93:7, 9:1, 85:15, 8:2, 7:3, 1:1; EtOAc–MeOH, 1:0, 9:1, 7:3, 0:1) to afford nine subfractions (Fr. 1–9). **15** (198.2 mg), **6**/**9** (1100.9 mg, mixture 7:3), and **43** (1230.1 mg, purity 50%) were obtained as Fr. 2, 3, and 8, respectively. Fr. 1 (132.0 mg) was separated using HPLC (COSMOSIL 5SL-II, 10 × 250 mm, *n*-hexane–EtOAc, 98:2) into six fractions: 1-0 to 1-5. **44** (32.6 mg), **40** (37.8 mg), and **15** (1.2 mg) were obtained as Fr. 1-2, 1-3, 1-5, respectively. Fr. 1-4 (10.0 mg) was separated using HPLC (COSMOSIL 5SL-II, 4.6 × 250 mm, *n*-hexane–EtOAc, 99:1) into four fractions: 1-4-0 to 1-4-3. Fr. 1-4-0 was **30** (1.9 mg). Fr. 1-4-1 (5.8 mg) was further purified using YMC-Pack Diol-120-NP (4.6 × 250 mm, *n*-hexane–EtOAc, 95:5) to yield **45** (3.6 mg) and **22** (1.3 mg). Fr. 4 (230.9 mg) was separated using HPLC (COSMOSIL 5SL-II, 10 × 250 mm, *n*-hexane–EtOAc, 85:15) into four fractions: 4-0 to 4-3. Fr. 4-3 was **7** (128.2 mg). Fr. 4-1 (62.7 mg) was purified using HPLC (COSMOSIL 5SL-II, 10 × 250 mm, *n*-hexane–EtOAc, 95:5) to yield **15** (0.7 mg), **6** (14.2 mg), **9** (10.1 mg), and **13** (11.0 mg). Fr. 4–2 (18.8 mg) was purified using HPLC (COSMOSIL 5SL-II, 10 × 250 mm, *n*-hexane–EtOAc, 85:15) into five fractions: 4-2-0 to 4-2-4. **41** (3.7 mg) and **33**/**38** (6.2 mg, mixture 2:1) were obtained as Fr. 4-2-2 and 4-2-3, respectively. Fr. 4-2-1 (1.5 mg) was further purified using YMC-Pack Diol-120-NP (4.6 × 250 mm, *n*-hexane–EtOAc, 95:5) to yield **14** (0.4 mg) and **20** (0.5 mg). Fr. 4-2-4 (2.2 mg) was further purified using YMC-Pack Diol-120-NP (4.6 × 250 mm, *n*-hexane–EtOAc, 9:1) to yield **34** (0.2 mg) and **26** (0.8 mg). Fr. 5 (66.4 mg) was separated using HPLC (COSMOSIL 5SL-II, 10 × 250 mm, *n*-hexane–EtOAc, 85:15) into seven fractions: 5-0 to 5-6. **6**/**15** (3.1 mg, mixture 10:3), **9** (1.4 mg), **24** (5.0 mg), **46** (3.3 mg), and **21** (1.6 mg) were obtained as Fr. 5-1, 5-2, 5-4–5-6, respectively. Fr. 5-3 (27.2 mg) was purified using YMC-Pack Diol-120-NP (4.6 × 250 mm, *n*-hexane–EtOAc, 85:15) into five fractions: 5-3-0 to 5-3-4. **7**/**8** (3.9 mg, mixture 3:2) and **29** (6.5 mg) were obtained as Fr. 5-3-1 and 5-3-4, respectively. Fr. 5-3-2 (5.6 mg) was further purified using Inertsil CN-3 (4.6 × 250 mm, *n*-hexane–EtOAc, 97:3) to yield **48** (0.8 mg), **35** (1.4 mg), and **23a**/**23b** (0.7 mg/1.3 mg, respectively). Fr. 5-3-3 (3.9 mg) was further purified using YMC-Pack Diol-120-NP (4.6 × 250 mm, *n*-hexane–EtOAc, 9:1) to yield **28** (0.6 mg). Fr. 6 (280.9 mg) was separated using HPLC (COSMOSIL 5SL-II, 10 × 250 mm, *n*-hexane–EtOAc, 7:3) into eight fractions: 6-0 to 6-7. Fr. 6-1 (47.4 mg) was purified using HPLC (COSMOSIL 5SL-II, 4.6 × 250 mm, *n*-hexane–EtOAc, 85:15) to yield **6**/**15/9** (5.6 mg, mixture 10:3:3), **7**/**29** (4.2 mg, mixture 10:3), and **24** (4.1 mg). Fr. 6-2 (31.9 mg) was purified using YMC-Pack Diol-120-NP (4.6 × 250 mm, *n*-hexane–EtOAc, 8:2) into four fractions: 6-2-0 to 6-2-3. **37**/**47** (8.7 mg, mixture 10:3) and **49** (1.6 mg) were obtained as Fr. 6-2-2 and 6-2-3, respectively. Fr. 6-2-1 (2.9 mg) was further purified using Inertsil CN-3 (4.6 × 250 mm, *n*-hexane–EtOAc, 9:1) to yield **4** (1.3 mg). Fr. 6-3 (21.6 mg) was purified using YMC-Pack Diol-120-NP (4.6 × 250 mm, *n*-hexane–EtOAc, 7:3) into four fractions: 6-3-0 to 6-3-3. Fr. 6-3-3 was **1** (2.2 mg). Fr. 6-3-1 (4.7 mg) was further purified using Inertsil CN-3 (4.6 × 250 mm, *n*-hexane–EtOAc, 9:1) to yield **42** (0.4 mg) and **17** (2.0 mg). Fr. 6-3-2 (2.0 mg) was further purified using Inertsil CN-3 (4.6 × 250 mm, *n*-hexane–EtOAc, 7:3) to yield **2** (1.1 mg). Fr. 6-4 (43.3 mg) was purified using HPLC (COSMOSIL 5SL-II, 4.6 × 250 mm, CHCl_3_–EtOAc, 98:2) into three fractions: 6-4-0 to 6-4-2. Fr. 6-4-2 was **18** (6.0 mg). Fr. 6-4-1 (20.4 mg) was further purified using YMC-Pack Diol-120-NP (4.6 × 250 mm, *n*-hexane–EtOAc, 85:15) to yield **16** (10.2 mg) and **32** (2.2 mg). Fr. 6-5 (48.0 mg) was purified on COSMOSIL 5SL-II (4.6 × 250 mm, CHCl_3_–EtOAc, 98:2) to give five fractions: 6-5-0 to 6-5-4. **10** (12.4 mg) and **11** (11.0 mg) were obtained as Fr. 6-5-2 and 6-5-4, respectively. Fr. 6-5-3 (3.0 mg) was further purified using Inertsil CN-3 (4.6 × 250 mm, *n*-hexane–EtOAc, 7:3) to yield **25** (0.8 mg) and **27** (0.4 mg). Fr. 6-6 (18.7 mg) was purified on COSMOSIL 5SL-II (4.6 × 250 mm, CHCl_3_–EtOAc, 98:2) to give five fractions: 6-6-0 to 6-6-4. Fr. 6-6-3 was **31** (1.4 mg). Fr. 6-6-1 (3.1 mg) was further purified using YMC-Pack Diol-120-NP (4.6 × 250 mm, *n*-hexane–EtOAc, 7:3) to yield **5** (1.4 mg). Fr. 6-6-4 (2.1 mg) was further purified using YMC-Pack Diol-120-NP (4.6 × 250 mm, *n*-hexane–EtOAc, 7:3) to yield **36** (0.4 mg) and **3** (0.8 mg).

Similarly, the MeOH extract (2.8 g) of sample 1 (29.6 g) yielded **6** (66.2 mg), **7** (28.8 mg), **8** (0.3 mg), **9** (8.1 mg), **10** (2.1 mg), **11** (5.4 mg), **12** (0.9 mg), **15** (55.7 mg), **16** (7.5 mg), **19** (7.8 mg), **22** (0.9 mg), **29** (1.1 mg), **33** (0.7 mg), **38** (0.4 mg), **39** (0.3 mg), **40** (1.6 mg), **41** (0.7 mg), **44** (10.2 mg), **46** (0.7 mg), and **47** (0.7 mg).

### 3.4. Chiral HPLC Analyses of Compounds **4** and **5**

Each compound was analyzed using HPLC with a Chiralcel OJ-RH column (4.6 × 150 mm, Daicel, Japan), which was eluted with 50% CH_3_CN (for **4**) or 20% CH_3_CN (for **5**) in 50 mM H_3_PO_4_ at 40 °C (flow rate, 0.8 mL/min and detection, JASCO photodiode array detector MD-2010), resulting in the detection of both enantiomers (**4**: *t*_R_ 13.4 min and *t*_R_ 14.9 min; **5**: *t*_R_ 4.0 min and *t*_R_ 4.8 min) with an integral ratio of ca. 1:1.

### 3.5. Calculation of ECD Spectra

A conformational search was performed using the Monte Carlo method and the MMFF94 force field with Spartan ′14 (Wavefunction, Irvine, CA, USA). The obtained low-energy conformers within 6 kcal/mol were optimized at the B3LYP/6-31G (d,p) level in MeOH (PCM). The vibrational frequencies were also calculated at the same level to confirm their stability, and no imaginary frequencies were found. The energies, oscillator strengths, and rotational strengths of the low-energy conformers were calculated using TDDFT at the CAM-B3LYP/6-31G (d,p) in MeOH (PCM) level, and weight-averaged. The ECD spectra were simulated using GaussView [54] by the overlapping Gaussian function with 0.35 eV exponential half-width, and UV correction was performed (redshifted by 15 nm). All DFT calculations were performed using Gaussian 09 [55].

### 3.6. Compound Data

Compound **1**

Yellow amorphous powder; ${\left[\alpha \right]}_{D}^{11}$ -23.9 (*c* 0.24, CHCl_3_); FT-IR 3416, 1651 cm^−1^; MS (FAB) *m*/*z*: 489 [M+Na]^+^; HRMS (FAB) Obs. *m*/*z* 489.1527 (Calcd for C_26_H_26_O_8_Na 489.1525); UV (CH_3_OH) λ_max_ (log ε) 231 (4.24), 260 (4.02), 391 (3.73) nm; ECD (CH_3_OH, *c* = 5.2 × 10^−5^ mol/L) λ_max_ (*Δ*ε): 406 (−0.2), 360 (+0.2), 318 (+0.6), 266 (+2.0), 218 (−4.2) nm; ^1^H and ^13^C NMR: see Table 1.

Compound **2**

Yellow amorphous powder; ${\left[\alpha \right]}_{D}^{28}$ +5.4 (*c* 0.10, CHCl_3_); FT-IR 3411, 1656, 1600, 1464, 1439, 1254, 1093 cm^−1^; MS (FAB) *m*/*z*: 466 [M]^+^; HRMS (FAB) Obs. *m*/*z* 466.1604 (Calcd for C_26_H_26_O_8_ 466.1628); UV (CH_3_OH) λ_max_ (log ε) 222 (4.39), 337 (3.71) nm; CD (CH_3_OH, *c* = 4.3 × 10^−5^ mol/L) λ_max_ (*Δ*ε): 352 (+1.9), 304 (−0.2), 260 (+2.3), 218 (−11.0) nm; ^1^H and ^13^C NMR: see Table 1.

Compound **3**

Yellow amorphous powder; ${\left[\alpha \right]}_{D}^{28}$ −59.7 (*c* 0.08, CHCl_3_); FT-IR 3474, 1668, 1596, 1457, 1438, 1251, 1081 cm^−1^; MS (FAB) *m*/*z*: 721 [M + Na]^+^; HRMS (FAB) Obs. *m*/*z* 721.2260 (Calcd for C_39_H_38_O_12_Na 721.2261); UV (CH_3_OH) λ_max_ (log ε) 221 (4.56), 335 (3.83) nm; CD (CH_3_OH, *c* = 3.0 × 10^−5^ mol/L) λ_max_ (*Δ*ε): 353 (+2.9), 311 (−0.2), 261 (+2.3), 221 (−13.2) nm; ^1^H and ^13^C NMR: see Table 1.

Compound **4**

Yellow amorphous powder; FT-IR 3361, 1659, 1651 cm^−1^; MS (FAB) *m*/*z*: 449 [M + H]^+^; HRMS (FAB) Obs. *m*/*z* 449.1600 (Calcd for C_26_H_25_O_7_ 449.1600); UV (CH_3_OH) λ_max_ (log ε) 223 (4.39), 234 (4.48), 266 (4.07), 329 (3.75) nm; ^1^H and ^13^C NMR: see Table 2.

Compound **5**

Yellow amorphous powder; FT-IR 3245, 2225, 1672, 1622 cm^−1^; MS (FAB) *m*/*z*: 461 [M + H]^+^; HRMS (FAB) Obs. *m*/*z* 461.1601 (Calcd for C_27_H_25_O_7_ 461.1600); UV (CH_3_OH) λ_max_ (log ε) 243 (4.44), 354 (4.24) nm; ^1^H and ^13^C NMR: see Table 2.

Compound **14**

Colorless amorphous powder; ${\left[\alpha \right]}_{D}^{28}$ +45.9 (*c* 0.05, CHCl_3_); FT-IR 3453–2650, 1731, 1651 cm^−1^; MS (FAB) *m*/*z*: 291 [M+H]^+^; HRMS (FAB) Obs. *m*/*z* 291.1231 (Calcd for C_16_H_19_O_5_ 291.1232); ^1^H and ^13^C NMR: see Table 3.

Compound **17**

Yellowish amorphous powder; ${\left[\alpha \right]}_{D}^{28}$ +15.9 (*c* 0.24, CHCl_3_); FT-IR 3472, 1713, 1651 cm^−1^; MS (FAB) *m*/*z*: 355 [M + Na]^+^; HRMS (FAB) Obs. *m*/*z* 355.1160 (Calcd for C_18_H_20_O_6_Na 355.1158); ^1^H and ^13^C NMR: see Table 3.

Compound **21**

Yellow amorphous powder; ${\left[\alpha \right]}_{D}^{28}$ −189.2 (*c* 0.17, CHCl_3_); FT-IR 3443–2646, 1651 cm^−1^; MS (FAB) *m*/*z*: 233 [M + H]^+^; HRMS (FAB) Obs. *m*/*z* 233.0814 (Calcd for C_13_H_13_O_4_ 233.0814); ^1^H and ^13^C NMR: see Table 3.

Compound **25**

Yellowish amorphous powder; ${\left[\alpha \right]}_{D}^{29}$ +7.0 (*c* 0.12, CHCl_3_); FT-IR 3410, 1650 cm^−1^; MS (FAB) *m*/*z*: 235 [M + H]^+^; HRMS (FAB) Obs. *m*/*z* 235.0970 (Calcd for C_13_H_15_O_4_ 235.0970); ^1^H and ^13^C NMR: see Table 4.

Compound **26**

Yellowish amorphous powder; ${\left[\alpha \right]}_{D}^{29}$ −11.5 (*c* 0.08, CHCl_3_); FT-IR 3541–2635, 1731, 1633 cm^−1^; MS (FAB) *m*/*z*: 277 [M+H]^+^; HRMS (FAB) Obs. *m*/*z* 277.1076 (Calcd for C_15_H_17_O_5_ 277.1076); ^1^H and ^13^C NMR: see Table 4.

Compound **31**

Yellow amorphous powder; ${\left[\alpha \right]}_{D}^{28}$ +2.3 (*c* 0.20, CHCl_3_); FT-IR 3485, 1731 cm^−1^; MS (FAB) *m*/*z*: 235 [M + H]^+^; HRMS (FAB) Obs. *m*/*z* 235.0970 (Calcd for C_13_H_15_O_4_ 235.0970); ^1^H and ^13^C NMR: see Table 4.

Compound **32**

Yellow amorphous powder; FT-IR 1683, 1595, 1566, 1474, 1360, 1331, 1174 cm^−1^; MS (FAB) *m*/*z*: 233 [M + H]^+^; HRMS (FAB) Obs. *m*/*z* 233.0814 (Calcd for C_13_H_13_O_4_ 233.0814); ^1^H and ^13^C NMR: see Table 4.

Compound **34**

Yellowish amorphous powder; ${\left[\alpha \right]}_{D}^{29}$ −65.2 (*c* 0.03, CHCl_3_); FT-IR 1745, 1681 cm^−1^; MS (FAB) *m*/*z*: 319 [M + H]^+^; HRMS (FAB) Obs. *m*/*z* 319.1543 (Calcd for C_18_H_23_O_5_ 319.1545); ^1^H and ^13^C NMR: see Table 5.

Compound **36**

Yellowish amorphous powder; ${\left[\alpha \right]}_{D}^{29}$ +37.00 (*c* 0.05, CHCl_3_); FT-IR 3410, 1680, 1601, 1497 cm^−1^; MS (FAB) *m*/*z*: 249 [M + H]^+^; HRMS (FAB) Obs. *m*/*z* 249.1126 (Calcd for C_14_H_17_O_4_ 249.1127); ^1^H NMR: see Table 5.

Compound **39**

White amorphous powder; ${\left[\alpha \right]}_{D}^{29}$ +25.34 (*c* 0.08, CHCl_3_); FT-IR 3305, 1746 cm^−1^; MS (FAB) *m*/*z*: 247 [M-H_2_O+H]^+^; HRMS (FAB) Obs. *m*/*z* 247.1334 (Calcd for C_15_H_19_O_3_ 247.1334); ^1^H and ^13^C NMR: see Table 5.

## Figures and Tables

**Table 1:** ^1^ H (500 MHz) and ^13^ C (126 MHz) NMR data of **1**–**3** in CDCl_3_.

	1				2				3		
Position	δ_H_	(*mult., J* in Hz)	δ_C_	Position	δ_H_	(*mult., J* in Hz)	δ_C_	Position	δ_H_	(*mult., J* in Hz)	δ_C_
2, 2′	5.00	(1H, br s)	92.9	2	5.04	(1H, br s)	92.2	2	4.97	(1H, br s)	91.86
3, 3′	5.34	(1H, br s)	77.7	3	5.21	(1H, br s)	76.3	3	5.06	(1H, br s)	76.22
4, 4′			119.2	4			128.1	4			126.31
5, 5′			153.4	5			148.8	5			149.28
6, 6′			130.3	6	6.91	(1H, d, 8.8)	122.1	6	6.76	(1H, d, 8.8)	118.89
7, 7′	7.14	(1H, s)	119.8	7	7.03	(1H, d, 8.8)	115.5	7	6.88	(1H, d, 8.8)	114.94
8, 8′			153.4	8			157.2	8			156.35
9, 9′			126.6	9			130.3	9			129.56
10, 10′			141.1	10			141.4	10			141.24 ^(a)^
11, 11′	5.10	(1H, s)	112.6	11	5.10	(1H, s)	112.0	11	5.04	(1H, s)	112.14
	4.94	(1H, s)			4.92	(1H, s)			4.88	(1H, s)	
12, 12′	1.79	(3H, s)	17.8	12	1.78	(3H, s)	17.6	12	1.72	(3H, s)	17.51
13, 13′			204.6	13			202.0	13			202.48
14, 14′	2.88	(3H, s)	31.1	14	2.69	(3H, s)	32.1	14	2.49	(3H, s)	32.23
3-OH, 3′-OH	2.84	(1H, br s)		3-OH	4.37	(1H, br s)		3-OH	4.46	(1H, d, 1.2)	
5-OH, 5′-OH	11.72	(1H, s)		2′	4.95	(1H, br s)	93.0	2′	5.06	(1H, br s)	92.72
				3′	5.29	(1H, dd, 7.1, 2.6)	77.5	3′	5.24	(1H, dd, 3.4, 1.7)	75.98
				4′			119.2	4′			130.41
				5′			148.0	5′			147.93
				6′			147.2	6′			139.88
				7′	6.66	(1H, s)	106.4	7′	6.81	(1H, s)	107.59
				8′			153.0	8′			157.70
				9′			120.6	9′			126.03
				10′			141.0	10′			141.09
				11′	5.04	(1H, s)	112.5	11′	5.13	(1H, s)	112.23
					4.92	(1H, s)			4.96	(1H, s)	
				12′	1.75	(3H, s)	17.7	12′	1.82	(3H, s)	17.74
				13′			203.6	13′			201.64
				14′	2.86	(3H, s)	31.3	14′	2.67	(3H, s)	30.98
				3′-OH	2.93	(1H, d, 7.1)		3′-OH	4.32	(1H, d, 1.7)	
				5′-OH	10.49	(1H, s)		2″	4.91	(1H, br s)	93.22
								3″	5.14	(1H, dd, 9.3, 2.4)	77.28
								4″			118.39
								5″			146.83
								6″			146.83
								7″	6.58	(1H, s)	104.86
								8″			152.60
								9″			120.32
								10″			141.09 ^(a)^
								11″	4.98	(1H, s)	112.06
									4.87	(1H, s)	
								12″	1.71	(3H, s)	17.67
								13″			204.18
								14″	2.78	(3H, s)	32.22
								3″-OH	4.04	(1H, d, 9.3)	
								5″-OH	11.25	(1H, s)	

^(a)^ interchangeable.

**Table 2:** ^1^H (500 MHz) and ^13^C (126 MHz) NMR data of **4** and **5** in CDCl_3_.

	4				5		
Position	δ_H_	(*mult., J* in Hz)	δ_C_	Position	δ_H_	(*mult., J* in Hz)	δ_C_
2			160.2	2			161.7
3	6.31	(1H, d, 0.8)	102.3	3	6.35	(1H, s)	99.0
4	7.70	(1H, s)	123.5	4	6.94	(1H, s)	106.5
5			116.8	5			158.7
6			160.8	6			115.4
7	6.79	(1H, br s)	99.1	7	7.70	(1H, s)	111.0
8			159.2	8			147.3
9			120.8	9			137.5
10			84.6	10			104.7
11	2.84	(1H, dd, 13.2, 1.2)	43.1	11	7.37	(1H, s)	144.4
	2.45	(1H, dd, 13.2, 8.8)		12	2.82	(1H, dddd, 16.2, 11.2, 6.0, 1.8)	18.9
12	1.74	(3H, s)	27.9		2.44	(1H, dddd, 16.2, 5.4, 2.6, 1.0)	
13			204.0	13			203.4
14	2.64	(3H, s)	26.9	14	2.66	(3H, s)	26.7
6-OH	12.42	(1H, s)		5-OH	12.26	(1H, s)	
2′			122.8	1′			129.6
3′	3.87	(1H, d, 8.8)	49.8	2′	7.47	(1H, d, 1.9)	110.0
4′	7.30	(1H, d, 1.0)	126.0	3′			146.7
5′			113.9	4′			151.6
6′			165.9	5′			108.1
7′	6.17	(1H, s)	97.9	6′	7.58	(1H, d, 1.9)	127.1
8′			165.5	7′			78.4
9′			120.9	8′			94.8
10′			141.7	9′			71.9
11′	5.46	(1H, s)	113.8	10′	2.29	(1H, ddd, 13.5, 6.0, 2.6)	33.2
	5.10	(1H, t, 1.5)			2.02	(1H, ddd, 13.5, 11.2, 5.4)	
12′	1.86	(3H, s)	18.2	11′	1.79	(3H, s)	28.1
13′			201.8	12′			196.1
14′	2.44	(3H, s)	26.1	13′	2.53	(3H, s)	26.2
5′-OH	12.76	(1H, s)		3′-OCH_3_	3.92	(3H, s)	56.3
				4′-OH	6.28	(1H, s)	

**Table 3:** ^1^H (500 MHz) and ^13^C (126 MHz) NMR data of **14**, **17**, and **21** in CDCl_3_.

	14			17			21		
Position	δ_H_	(*mult., J* in Hz)	δ_C_	δ_H_	(*mult., J* in Hz)	δ_C_	δ_H_	(*mult., J* in Hz)	δ_C_
2	5.14	(1H, d, 6.4)	89.2			160.6	5.47	(1H, d, 6.1)	88.4
3	6.26	(1H, d, 6.4)	72.2	6.64	(1H, d, 1.0)	102.5	5.67	(1H, d, 6.1)	81.9
4	7.81	(1H, s)	130.0	7.93	(1H, s)	123.7	7.81	(1H, s)	129.2
5			114.7			117.0			114.8
6			166.7			161.1			167.0
7	6.46	(1H, s)	98.9	6.99	(1H, br s)	99.8	6.38	(1H, s)	98.5
8			166.7			159.5			166.8
9			117.8			120.8			118.3
10			138.0			71.3			145.3
11	5.20	(1H, d, 1.0)	114.7	4.53	(1H, d, 11.5)	69.7	4.36	(1H, d, 12.5)	69.2
	5.12	(1H, d, 1.0)		4.38	(1H, d, 11.5)		4.22	(1H, d, 12.5)	
12	1.81	(3H, s)	19.2	1.67	(3H, s)	23.9	5.56	(1H, s)	112.9
							5.38	(1H, s)	
13			202.7			204.0			202.5
14	2.57	(3H, s)	26.4	2.69	(3H, s)	26.9	2.58	(3H, s)	26.3
1′			174.1			167.9			
2′	2.31	(2H, q, 7.6)	27.7			127.1			
3′	1.12	(3H, t, 7.6)	9.0	6.09	(1H, qq, 7.3, 1.4)	139.5			
4′				1.89	(3H, dq, 7.3, 1.4)	15.8			
5′				1.83	(3H, quint, 1.4)	20.5			
6-OH	13.04	(1H, s)		12.46	(1H, s)		12.99	(1H, s)	

**Table 4:** ^1^H (500 MHz) and ^13^C (126 MHz) NMR data of **25**, **26**, **31**, and **32** in CDCl_3_.

	25			26			31			32		
Position	δ_H_	(*mult., J* in Hz)	δ_C_	δ_H_	(*mult., J* in Hz)	δ_C_	δ_H_	(*mult., J* in Hz)	δ_C_	δ_H_	(*mult., J* in Hz)	δ_C_
2			166.8			165.7			162.0			154.2
3	6.46	(1H, s)	102.8	6.42	(1H, s)	102.6	6.70	(1H, s)	102.7	7.56	(1H, s)	112.9
4	7.01	(1H, s)	107.4	7.01	(1H, s)	107.5	7.80	(1H, d, 1.5)	115.9	7.93	(1H, d, 1.5)	117.4
5			158.4			158.4			133.8			134.7
6			115.8			115.9	7.48	(1H, d, 1.5)	105.5	7.62	(1H, d, 1.5)	108.0
7	7.78	(1H, s)	111.8	7.78	(1H, s)	111.8			145.3			146.2
8			147.8			147.7			146.8			147.8
9			136.7			136.7			129.4			128.3
10	3.18	(1H, sext, 6.5)	36.9	3.32	(1H, sext, 6.8)	33.7	5.06	(1H, m)	64.0			188.6
11	3.88	(2H, m)	66.0	4.36	(1H, dd, 11.0, 6.8)	66.3						
				4.27	(1H, dd, 11.0, 6.3)							
12	1.39	(3H, d, 7.1)	14.9	1.39	(3H, d, 7.1)	15.4	1.66	(3H, d, 6.6)	21.4	2.66	(3H, s)	26.7
13			203.7			203.8			197.6			197.1
14	2.68	(3H, s)	26.7	2.68	(3H, s)	26.7	2.66	(3H, s)	26.6	2.68	(3H, s)	26.6
1′						170.9						
2′				2.05	(3H, s)	20.9						
5-OH	12.16	(1H, s)		12.16	(1H, s)							
7-OCH_3_							4.06	(3H, s)	56.1	4.08	(3H, s)	56.2
10-OH							2.18	(1H, d, 4.9)				

**Table 5:** ^1^H (500 MHz) and ^13^C (126 MHz) NMR data of **34**, **36**, and **39** in CDCl_3_.

	34			36 ^(a)^		39				
Position	δ_H_	(*mult., J* in Hz)	δ_C_	δ_H_	(*mult., J* in Hz)	δ_H_ (Major/Minor)		(*mult., J* in Hz)	δ_C_ (Major/Minor)	
1									141.60	
2	5.13	(1H, d, 2.8)	92.1	5.04	(1H, d, 4.0)	6.761		(1H, d, 0.5)	111.67	
3	6.17	(1H, d, 2.8)	77.3	5.22	(1H, dd, 7.8, 4.0)				158.47	
4	7.62	(1H, d, 1.5)	120.7	7.65	(1H, d, 1.5)				127.58	
5			N.D. ^(b)^			7.222		(1H, d, 7.6)	122.68	
6	7.59	(1H, d, 1.5)	113.0	7.57	(1H, d, 1.5)	6.848		(1H, dd, 7.6, 0.5)	123.07	
7			144.8			2.353		(3H, s)	21.72	
8			154.0						79.73	
9			N.D. ^(b)^			6.506		(1H, s)	104.85/104.80	
10			140.5			1.626		(3H, s)	20.12	
11	5.10	(1H, s)	114.1	5.13	(1H, d, 0.8)					
	4.98	(1H, s)		4.97	(1H, d, 0.8)					
12	1.76	(3H, s)	17.5	1.76	(3H, s)					
13			196.4							
14	2.56	(3H, s)	26.4	2.58	(3H, s)					
1′			176.8						175.28	
2′	2.57	(1H, sept, 7.1)	33.9			2.407/2.398		(1H, sext, 7.0)	41.02/40.97	
3′	1.20	(3H, d, 7.1)	18.9			1.688		(1H, m)	26.49/26.35	
						1.498		(1H, m)		
4′	1.17	(3H, d, 7.1)	18.8			0.913/0.907		(3H, t, 7.0)	11.42	
5′						1.157/1.164		(3H, d, 7.0)	16.07/16.38	
3-OH				2.16	(1H, d, 7.8)					
7-OCH_3_	3.96	(3H, s)	56.2	3.96	(3H, s)					
8-OH						2.033/2.036		(1H, s)		

^(a) 13^C NMR was not measured due to a small amount. ^(b)^ N.D. = not detected.

## Data Availability

Not applicable.

## Supplementary Materials

The following supporting information can be downloaded at: https://www.mdpi.com/article/10.3390/molecules27248856/s1, Figures S1–S18, S21–S32, and S34–S102: 1D and 2D NMR spectra of compounds **1**–**5**, **14**, **17**, **21**, **25**, **26**, **31**, **32**, **34**, **36**, **39**, **20**, and **28**.; Figure S19: Experimental ECD spectrum of **29** and calculated ECD spectra of 2*S*,3*R*-**29** and 2*R*,3*R*-**29**.; Figure S20: Optimized structures (B3LYP/6-31G(d,p) in MeOH (PCM)) and calculated ECD spectra (CAM-B3LYP/6-31G(d,p) in MeOH (PCM)) for each conformer of **29** (**29A**–**29F**).; Figure S33: Experimental and calculated ECD spectra and chiral HPLC analysis of **4** and **5**.

## Sample Availability

Samples of the compounds are not available from the authors.
