# Supplementary material for: New Benzofuran Oligomers from the Roots of Eupatorium heterophyllum Collected in China
Source: Molecules. 2022 Dec 13;27(24):8856. doi: 10.3390/molecules27248856 (PMC9787490; doi:10.3390/molecules27248856)

Supporting Information

New Benzofuran Oligomers from the Roots of *Eupatorium heterophyllum* Collected in China

Yiming Hu [1], Yoshinori Saito [1,*], Yosuke Matsuo [1], Xun Gong [2] and Takashi Tanaka [1,*]

[1] Graduate School of Biomedical Sciences, Nagasaki University, 1-14 Bunkyo-machi, Nagasaki 852-8521, Japan
[2] Kunming Institute of Botany, Chinese Academy of Sciences, Kunming 650201, China
[*] Correspondence: saiyoshi@nagasaki-u.ac.jp (Y.S.); t-tanaka@nagasaki-u.ac.jp (T.T.);
Tel.: +81-95-819-2433 (Y.S.); +81-95-819-2432 (T.T.)

Index

Figure S1. ^{1}H NMR spectrum of **1** (measured in $CDCl_3$, 500 MHz). 1
Figure S2. ^{13}C NMR spectrum of **1** (measured in $CDCl_3$, 126 MHz). 2
Figure S3. ^{1}H-^{1}H COSY spectrum of **1** (measured in $CDCl_3$, 500 MHz). 3
Figure S4. HSQC spectrum of **1** (measured in $CDCl_3$, 500 MHz). 4
Figure S5. HMBC spectrum of **1** (measured in $CDCl_3$, 500 MHz). 5
Figure S6. NOESY spectrum of **1** (measured in $CDCl_3$, 500 MHz) 6
Figure S7. ^{1}H NMR spectrum of **2** (measured in $CDCl_3$, 500 MHz). 7
Figure S8. ^{13}C NMR spectrum of **2** (measured in $CDCl_3$, 126 MHz). 8
Figure S9. ^{1}H-^{1}H COSY spectrum of **2** (measured in $CDCl_3$, 500 MHz). 9
Figure S10. HSQC spectrum of **2** (measured in $CDCl_3$, 500 MHz). 10
Figure S11. HMBC spectrum of **2** (measured in $CDCl_3$, 500 MHz). 11

Figure S12. NOESY spectrum of **2** (measured in $CDCl_3$, 500 MHz). 12
Figure S13. ^{1}H NMR spectrum of **3** (measured in $CDCl_3$, 500 MHz). 13
Figure S14. ^{13}C NMR spectrum of **3** (measured in $CDCl_3$, 126 MHz). 14
Figure S15. ^{1}H-^{1}H COSY spectrum of **3** (measured in $CDCl_3$, 500 MHz). 15
Figure S16. HSQC spectrum of **3** (measured in $CDCl_3$, 500 MHz). 16
Figure S17. HMBC spectrum of **3** (measured in $CDCl_3$, 500 MHz). 17
Figure S18. NOESY spectrum of **3** (measured in $CDCl_3$, 500 MHz). 18
Figure S19. Experimental ECD spectra of **29** and calculated ECD spectra of 2*S*,3*R*-**29** and 2*R*,3*R*-**29**. 19
Figure S20. Optimized structures (B3LYP/6-31G(d,p) in MeOH (PCM)) and calculated ECD spectra (CAM-B3LYP/6-31G(d,p) in MeOH (PCM)) for each conformer of **29 (29A–29F)**. 20
Figure S21. ^{1}H NMR spectrum of **4** (measured in $CDCl_3$, 500 MHz). 21
Figure S22. ^{13}C NMR spectrum of **4** (measured in $CDCl_3$, 126 MHz). 22
Figure S23. ^{1}H-^{1}H COSY spectrum of **4** (measured in $CDCl_3$, 500 MHz). 23
Figure S24. HSQC spectrum of **4** (measured in $CDCl_3$, 500 MHz). 24
Figure S25. HMBC spectrum of **4** (measured in $CDCl_3$, 500 MHz). 25
Figure S26. NOESY spectrum of **4** (measured in $CDCl_3$, 500 MHz). 26
Figure S27. ^{1}H NMR spectrum of **5** (measured in $CDCl_3$, 500 MHz). 27
Figure S28. ^{13}C NMR spectrum of **5** (measured in $CDCl_3$, 126 MHz). 28
Figure S29. ^{1}H-^{1}H COSY spectrum of **5** (measured in $CDCl_3$, 500 MHz). 29
Figure S30. HSQC spectrum of **5** (measured in $CDCl_3$, 500 MHz). 30
Figure S31. HMBC spectrum of **5** (measured in $CDCl_3$, 500 MHz). 31
Figure S32. NOESY spectrum of **5** (measured in $CDCl_3$, 500 MHz). 32
Figure S33. Experimental and calculated ECD spectra and chiral HPLC analysis of **4** and **5**. 33

Figure S34. ^{1}H NMR spectrum of **14** (measured in $CDCl_3$, 500 MHz). 34
Figure S35. ^{13}C NMR spectrum of **14** (measured in $CDCl_3$, 126 MHz). 35
Figure S36. ^{1}H-^{1}H COSY spectrum of **14** (measured in $CDCl_3$, 500 MHz). 36
Figure S37. HSQC spectrum of **14** (measured in $CDCl_3$, 500 MHz). 37
Figure S38. HMBC spectrum of **14** (measured in $CDCl_3$, 500 MHz). 38
Figure S39. NOESY spectrum of **14** (measured in $CDCl_3$, 500 MHz). 39
Figure S40. ^{1}H NMR spectrum of **17** (measured in $CDCl_3$, 500 MHz). 40
Figure S41. ^{13}C NMR spectrum of **17** (measured in $CDCl_3$, 126 MHz). 41
Figure S42. ^{1}H-^{1}H COSY spectrum of **17** (measured in $CDCl_3$, 500 MHz). 42
Figure S43. HSQC spectrum of **17** (measured in $CDCl_3$, 500 MHz). 43
Figure S44. HMBC spectrum of **17** (measured in $CDCl_3$, 500 MHz). 44
Figure S45. NOESY spectrum of **17** (measured in $CDCl_3$, 500 MHz). 45
Figure S46. ^{1}H NMR spectrum of **21** (measured in $CDCl_3$, 500 MHz). 46
Figure S47. ^{13}C NMR spectrum of **21** (measured in $CDCl_3$, 126 MHz). 47
Figure S48. ^{1}H-^{1}H COSY spectrum of **21** (measured in $CDCl_3$, 500 MHz). 48
Figure S49. HSQC spectrum of **21** (measured in $CDCl_3$, 500 MHz). 49
Figure S50. HMBC spectrum of **21** (measured in $CDCl_3$, 500 MHz). 50
Figure S51. NOESY spectrum of **21** (measured in $CDCl_3$, 500 MHz). 51
Figure S52. ^{1}H NMR spectrum of **25** (measured in $CDCl_3$, 500 MHz). 52
Figure S53. ^{13}C NMR spectrum of **25** (measured in $CDCl_3$, 126 MHz). 53
Figure S54. ^{1}H-^{1}H COSY spectrum of **25** (measured in $CDCl_3$, 500 MHz). 54
Figure S55. HSQC spectrum of **25** (measured in $CDCl_3$, 500 MHz). 55
Figure S56. HMBC spectrum of **25** (measured in $CDCl_3$, 500 MHz). 56

Figure S57. NOESY spectrum of **25** (measured in $CDCl_3$, 500 MHz). 57
Figure S58. ^{1}H NMR spectrum of **26** (measured in $CDCl_3$, 500 MHz). 58
Figure S59. ^{13}C NMR spectrum of **26** (measured in $CDCl_3$, 126 MHz). 59
Figure S60. ^{1}H-^{1}H COSY spectrum of **26** (measured in $CDCl_3$, 500 MHz). 60
Figure S61. HSQC spectrum of **26** (measured in $CDCl_3$, 500 MHz). 61
Figure S62. HMBC spectrum of **26** (measured in $CDCl_3$, 500 MHz). 62
Figure S63. NOESY spectrum of **26** (measured in $CDCl_3$, 500 MHz). 63
Figure S64. ^{1}H NMR spectrum of **31** (measured in $CDCl_3$, 500 MHz). 64
Figure S65. ^{13}C NMR spectrum of **31** (measured in $CDCl_3$, 126 MHz). 65
Figure S66. ^{1}H-^{1}H COSY spectrum of **31** (measured in $CDCl_3$, 500 MHz). 66
Figure S67. HSQC spectrum of **31** (measured in $CDCl_3$, 500 MHz). 67
Figure S68. HMBC spectrum of **31** (measured in $CDCl_3$, 500 MHz). 68
Figure S69. NOESY spectrum of **31** (measured in $CDCl_3$, 500 MHz). 69
Figure S70. ^{1}H NMR spectrum of **32** (measured in $CDCl_3$, 500 MHz). 70
Figure S71. ^{13}C NMR spectrum of **32** (measured in $CDCl_3$, 126 MHz). 71
Figure S72. ^{1}H-^{1}H COSY spectrum of **32** (measured in $CDCl_3$, 500 MHz). 72
Figure S73. HSQC spectrum of **32** (measured in $CDCl_3$, 500 MHz). 73
Figure S74. HMBC spectrum of **32** (measured in $CDCl_3$, 500 MHz). 74
Figure S75. NOESY spectrum of **32** (measured in $CDCl_3$, 500 MHz). 75
Figure S76. ^{1}H NMR spectrum of **34** (measured in $CDCl_3$, 500 MHz). 76
Figure S77. ^{13}C NMR spectrum of **34** (measured in $CDCl_3$, 126 MHz). 77
Figure S78. ^{1}H-^{1}H COSY spectrum of **34** (measured in $CDCl_3$, 500 MHz). 78
Figure S79. HSQC spectrum of **34** (measured in $CDCl_3$, 500 MHz). 79

Figure S80. HMBC spectrum of **34** (measured in $CDCl_3$, 500 MHz). 80
Figure S81. NOESY spectrum of **34** (measured in $CDCl_3$, 500 MHz). 81
Figure S82. ^{1}H NMR spectrum of **36** (measured in $CDCl_3$, 500 MHz). 82
Figure S83. ^{1}H-^{1}H COSY spectrum of **36** (measured in $CDCl_3$, 500 MHz). 83
Figure S84. NOESY spectrum of **36** (measured in $CDCl_3$, 500 MHz). 84
Figure S85. ^{1}H NMR spectrum of **39** (measured in $CDCl_3$, 500 MHz). 85
Figure S86. ^{13}C NMR spectrum of **39** (measured in $CDCl_3$, 126 MHz). 86
Figure S87. ^{1}H-^{1}H COSY spectrum of **39** (measured in $CDCl_3$, 500 MHz). 87
Figure S88. HSQC spectrum of **39** (measured in $CDCl_3$, 500 MHz). 88
Figure S89. HMBC spectrum of **39** (measured in $CDCl_3$, 500 MHz). 89
Figure S90. NOESY spectrum of **39** (measured in $CDCl_3$, 500 MHz). 90
Figure S91. ^{1}H NMR spectrum of **20** (measured in $CDCl_3$, 500 MHz). 91
Figure S92. ^{13}C NMR spectrum of **20** (measured in $CDCl_3$, 126 MHz). 92
Figure S93. ^{1}H-^{1}H COSY spectrum of **20** (measured in $CDCl_3$, 500 MHz). 93
Figure S94. HSQC spectrum of **20** (measured in $CDCl_3$, 500 MHz). 94
Figure S95. HMBC spectrum of **20** (measured in $CDCl_3$, 500 MHz). 95
Figure S96. NOESY spectrum of **20** (measured in $CDCl_3$, 500 MHz). 96
Figure S97. ^{1}H NMR spectrum of **28** (measured in $CDCl_3$, 500 MHz). 97
Figure S98. ^{13}C NMR spectrum of **28** (measured in $CDCl_3$, 126 MHz). 98
Figure S99. ^{1}H-^{1}H COSY spectrum of **28** (measured in $CDCl_3$, 500 MHz). 99
Figure S100. HSQC spectrum of **28** (measured in $CDCl_3$, 500 MHz). 100
Figure S101. HMBC spectrum of **28** (measured in $CDCl_3$, 500 MHz). 101
Figure S102. NOESY spectrum of **28** (measured in $CDCl_3$, 500 MHz). 102

Figure S1. ^{1}H NMR spectrum of **1** (measured in $CDCl_3$, 500 MHz).

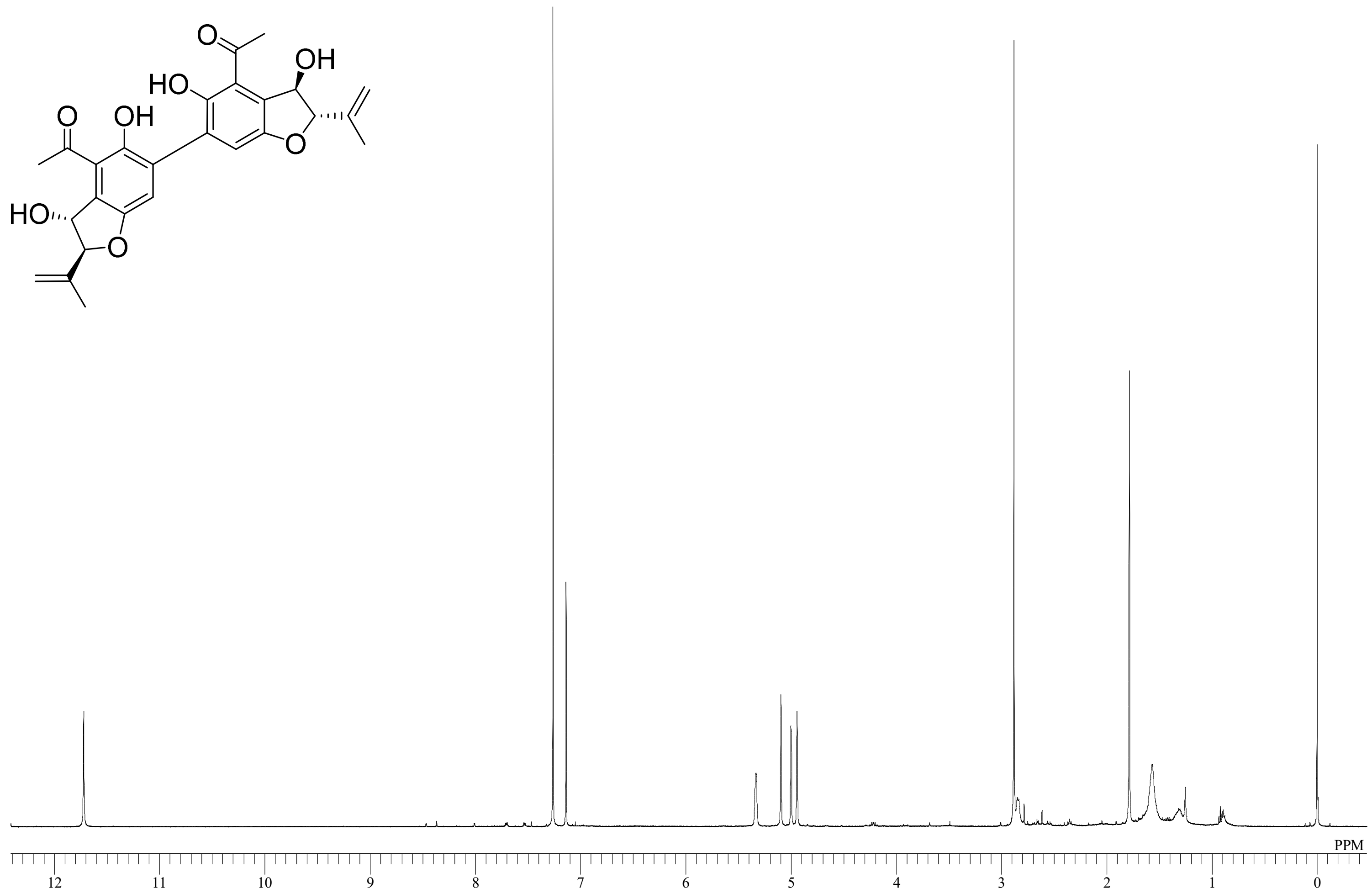

Figure S2. ^{13}C NMR spectrum of **1** (measured in $CDCl_3$, 126 MHz).

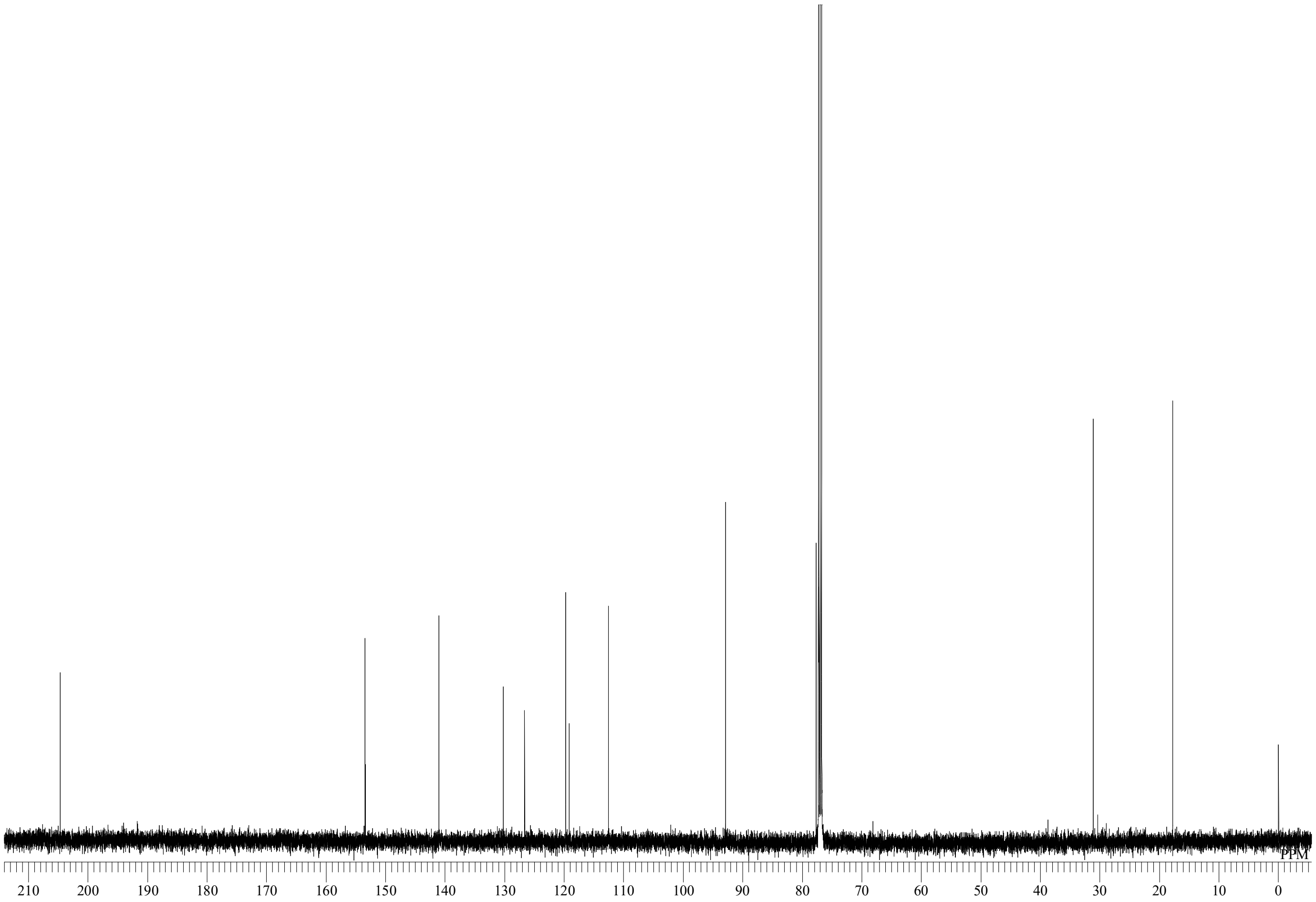

Figure S3. ^{1}H-^{1}H COSY spectrum of **1** (measured in $CDCl_3$, 500 MHz).

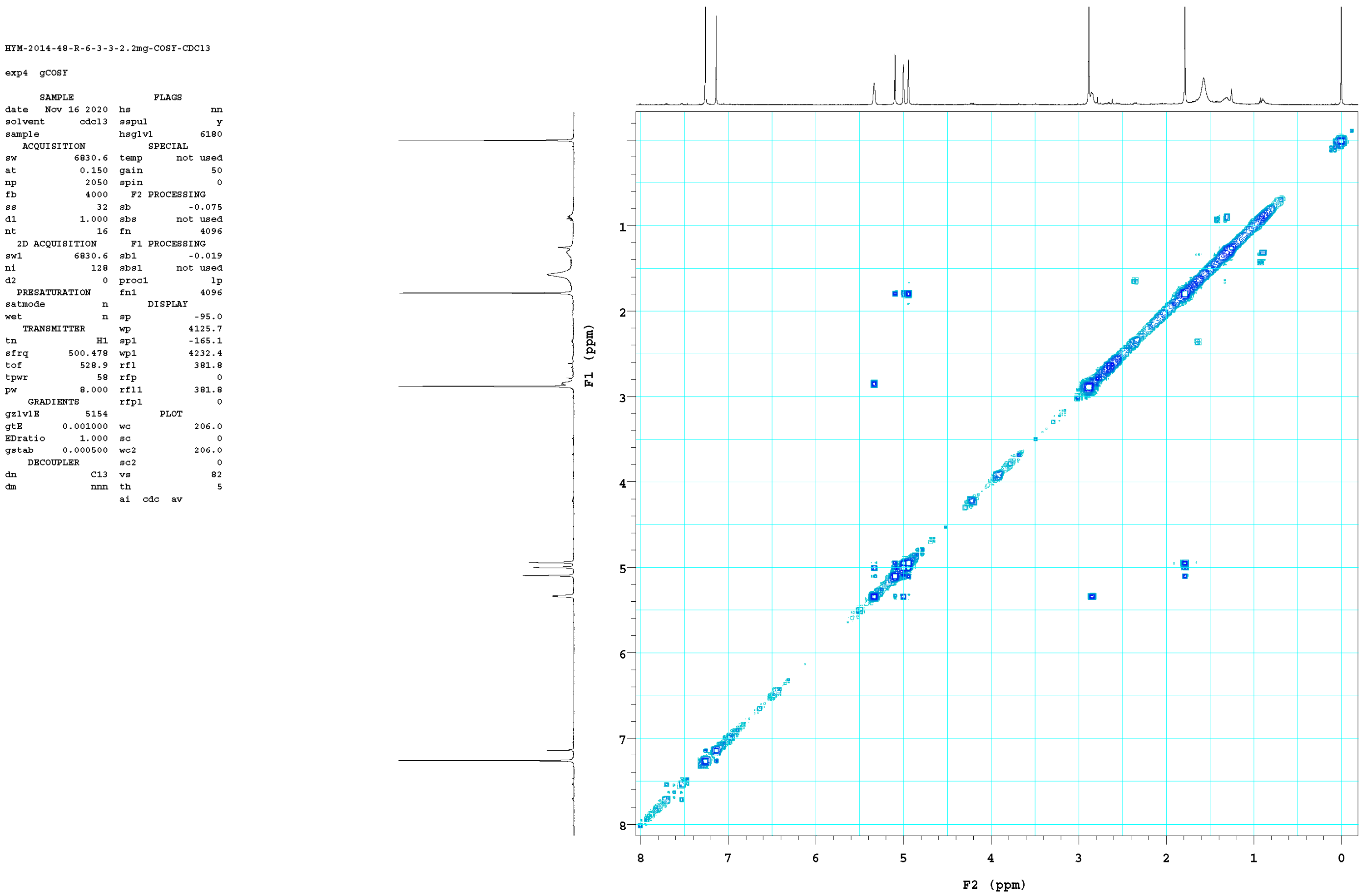

Figure S4. HSQC spectrum of **1** (measured in $CDCl_3$, 500 MHz).

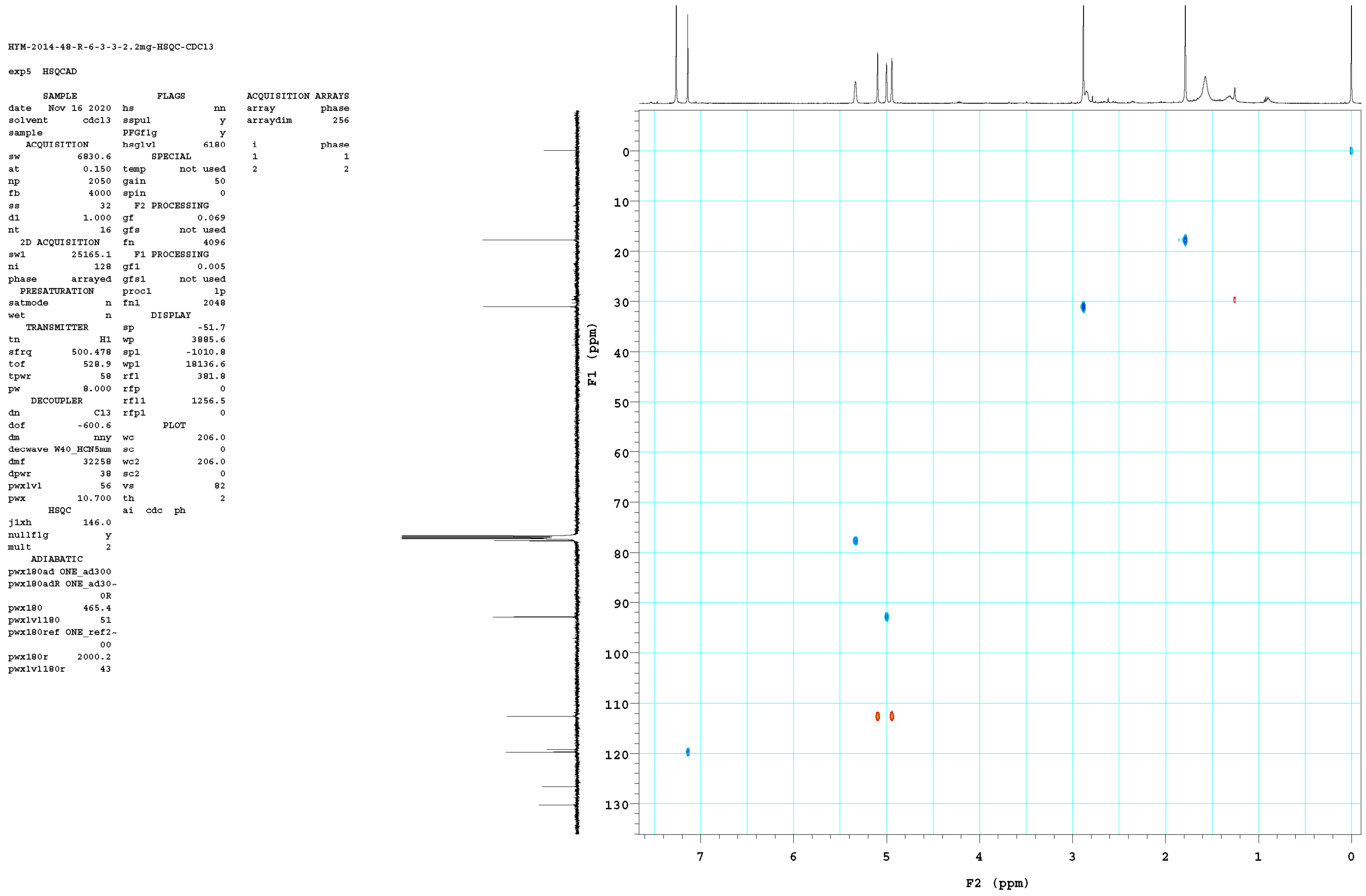

Figure S5. HMBC spectrum of **1** (measured in $CDCl_3$, 500 MHz).

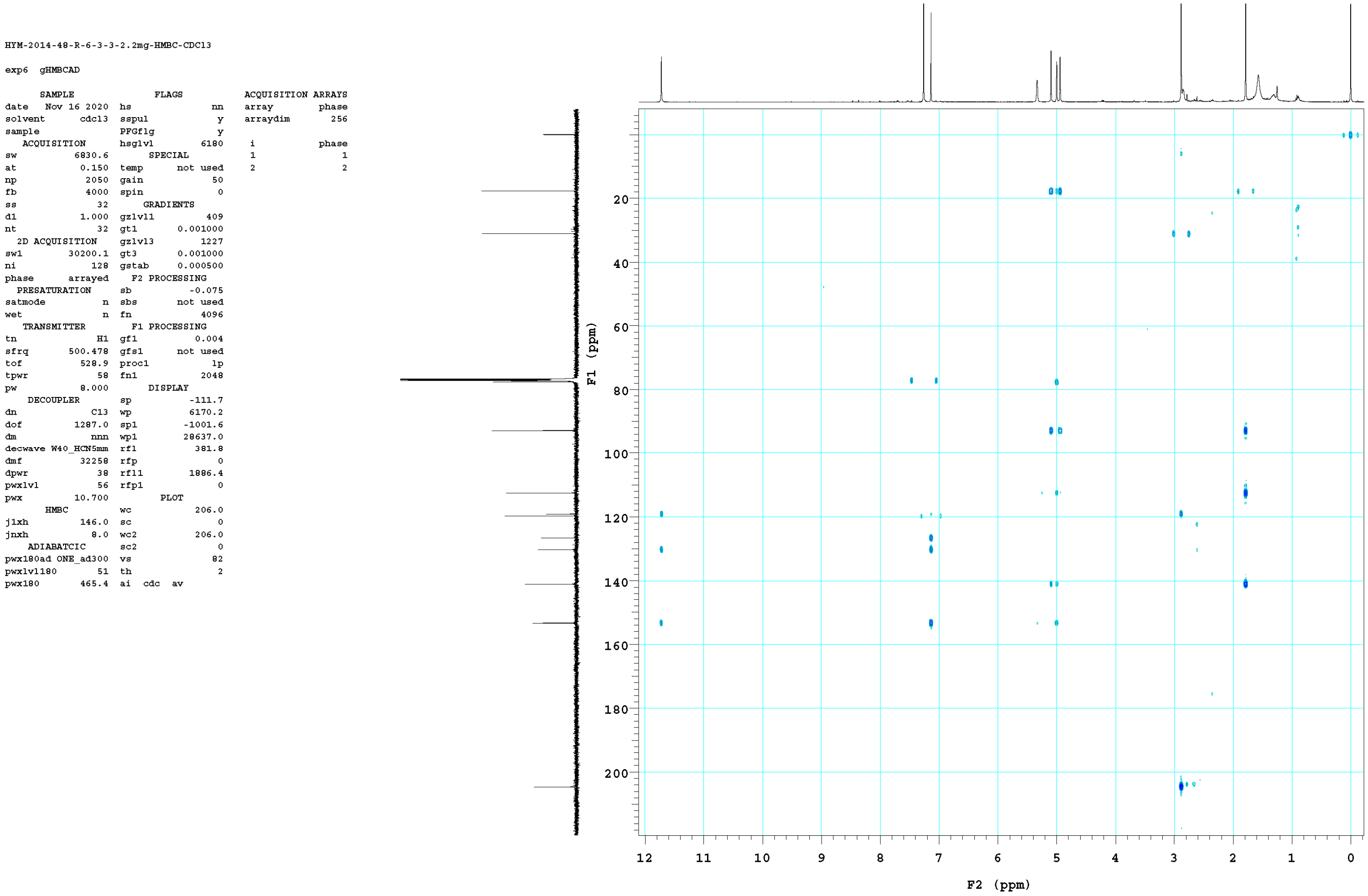

Figure S6. NOESY spectrum of **1** (measured in $CDCl_3$, 500 MHz).

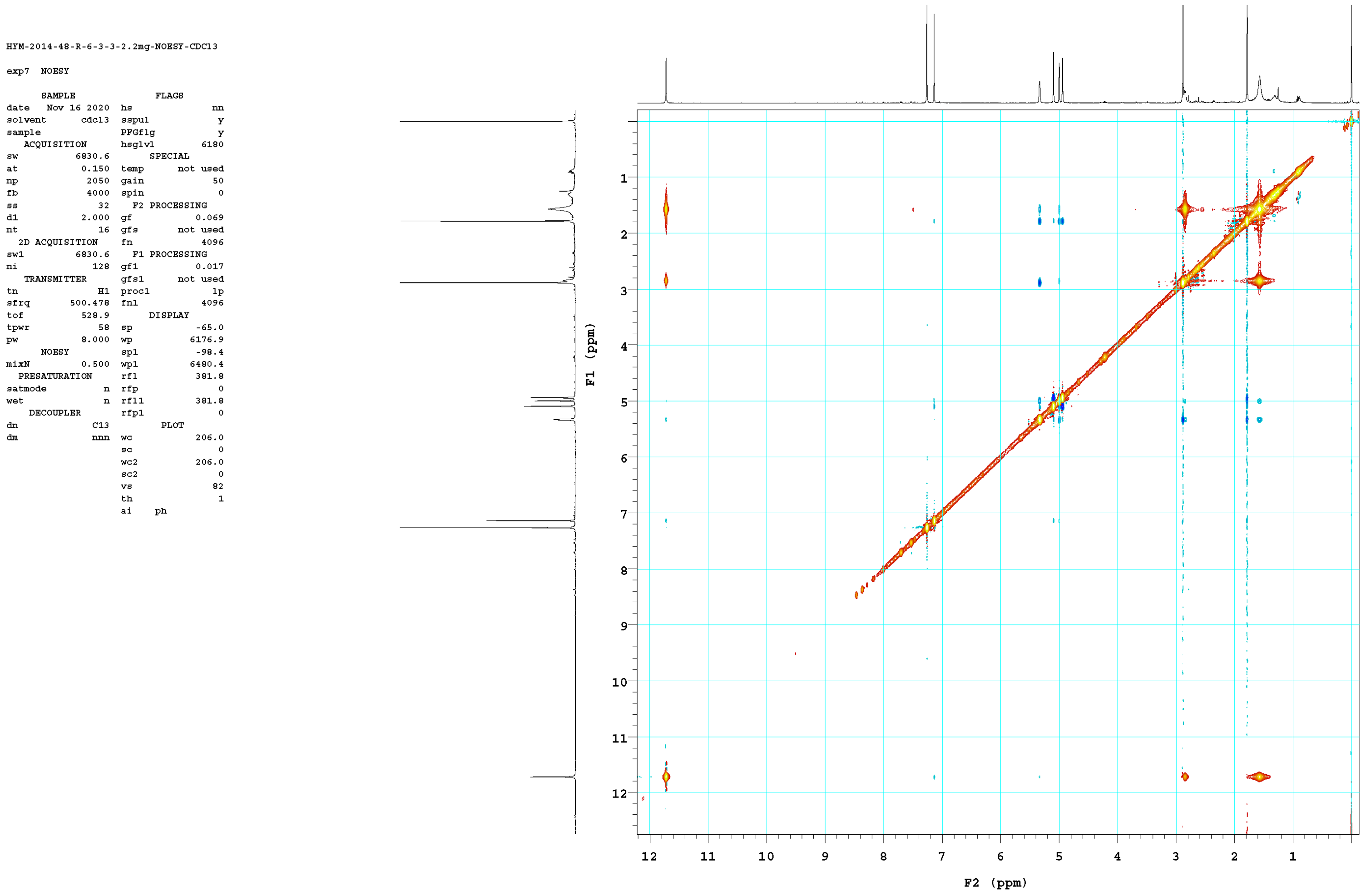

Figure S7. ^{1}H NMR spectrum of **2** (measured in $CDCl_3$, 500 MHz).

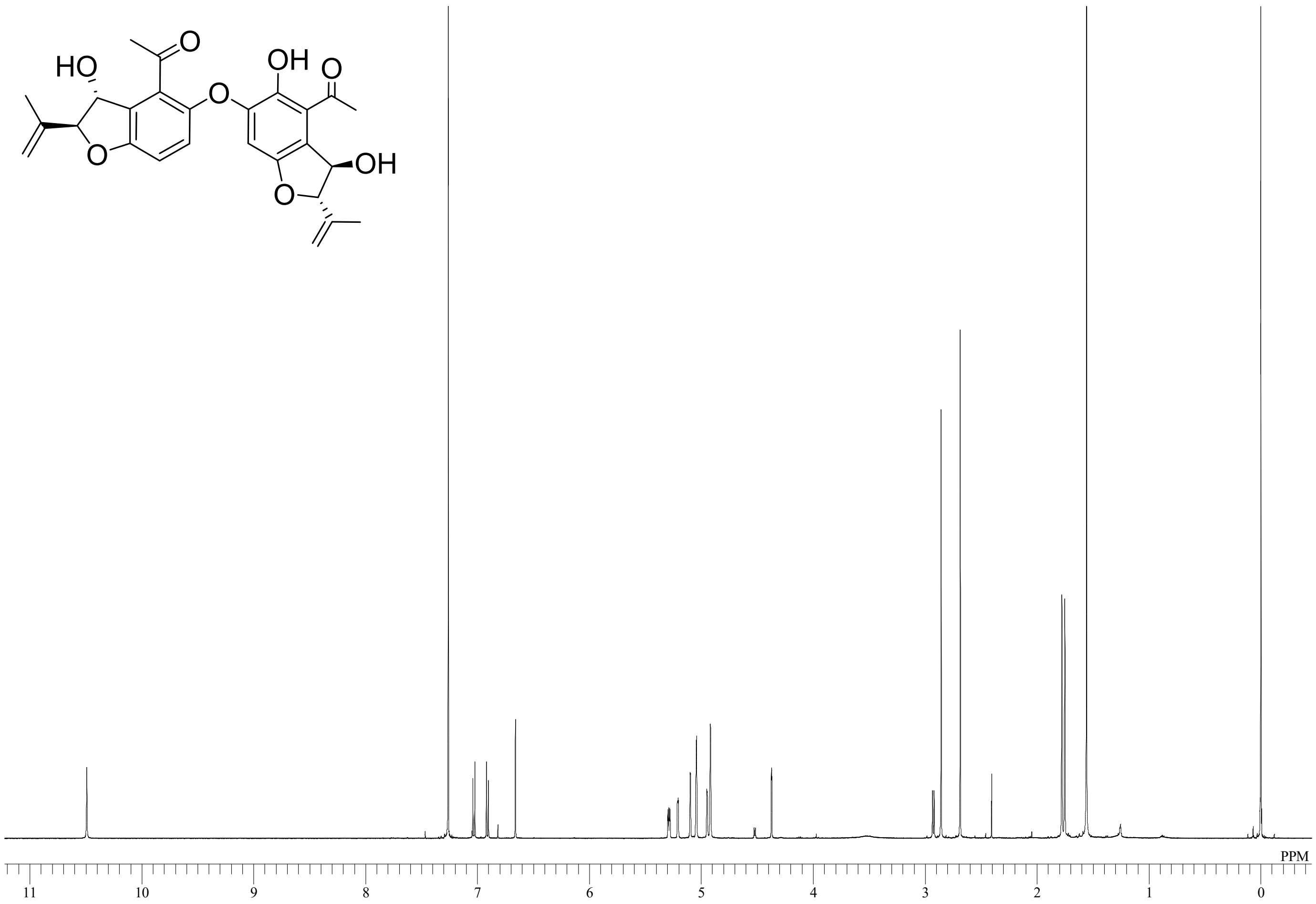

Figure S8. ^{13}C NMR spectrum of **2** (measured in $CDCl_3$, 126 MHz).

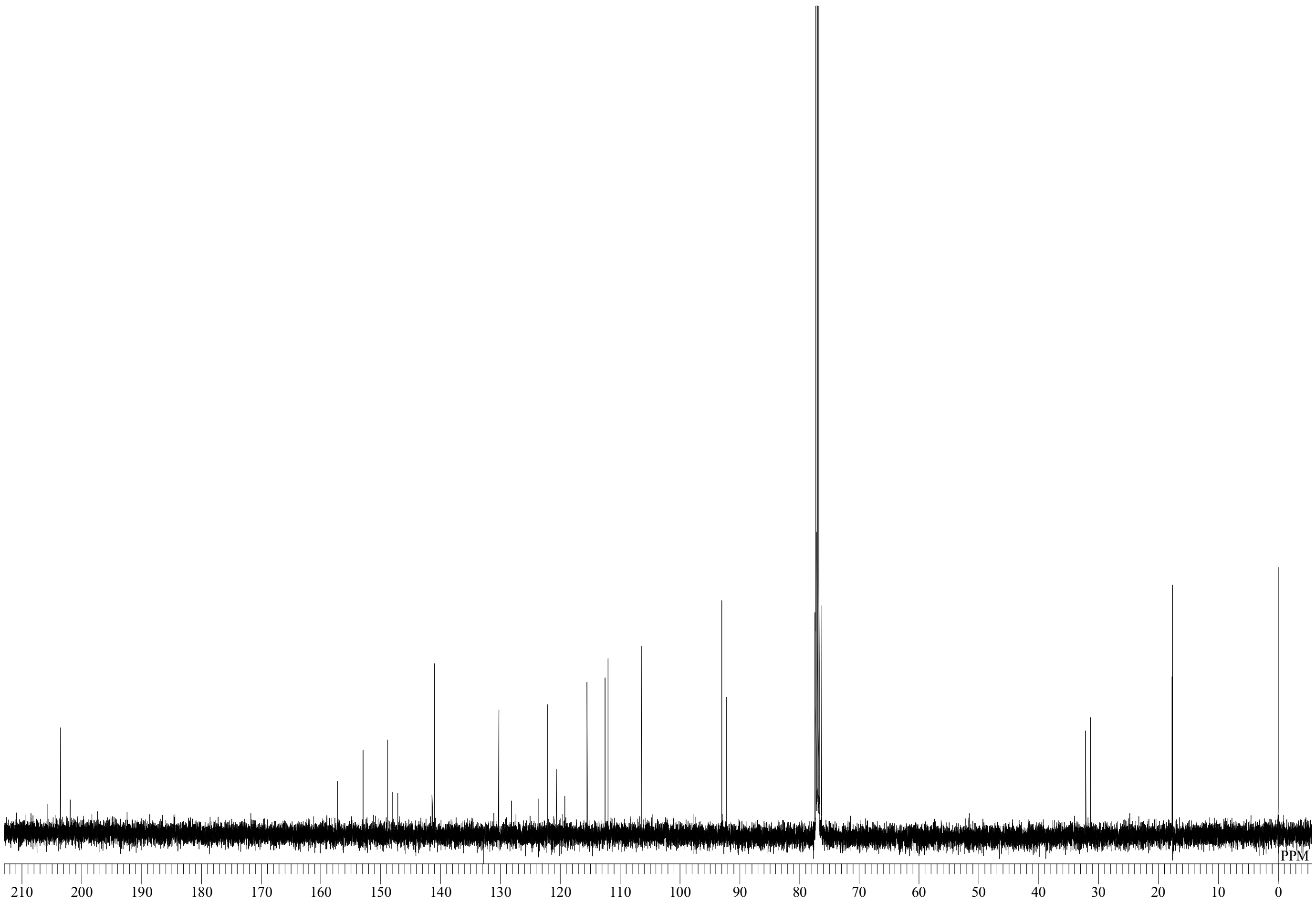

Figure S9. ^{1}H-^{1}H COSY spectrum of **2** (measured in $CDCl_3$, 500 MHz).

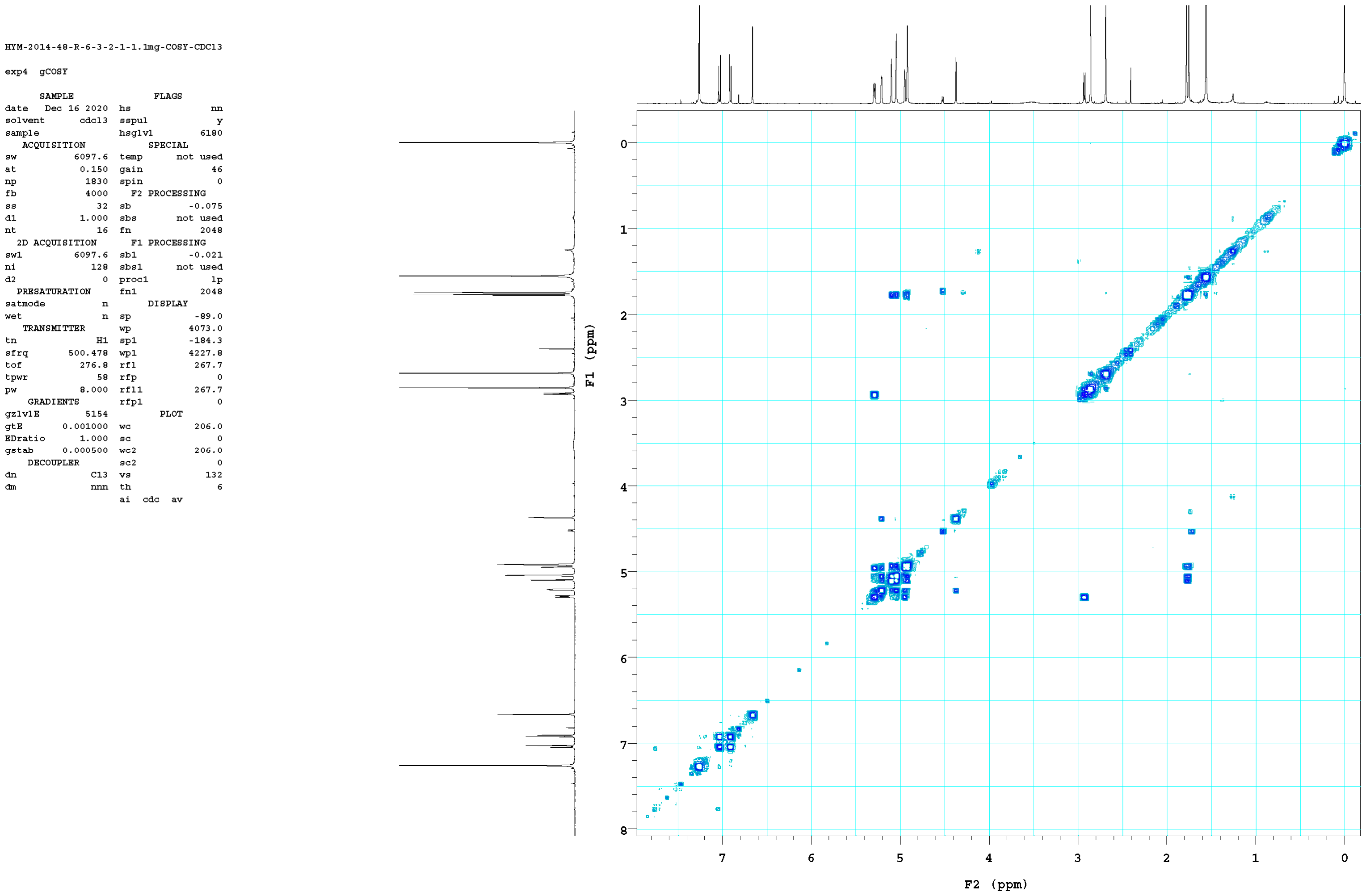

Figure S10. HSQC spectrum of **2** (measured in $CDCl_3$, 500 MHz).

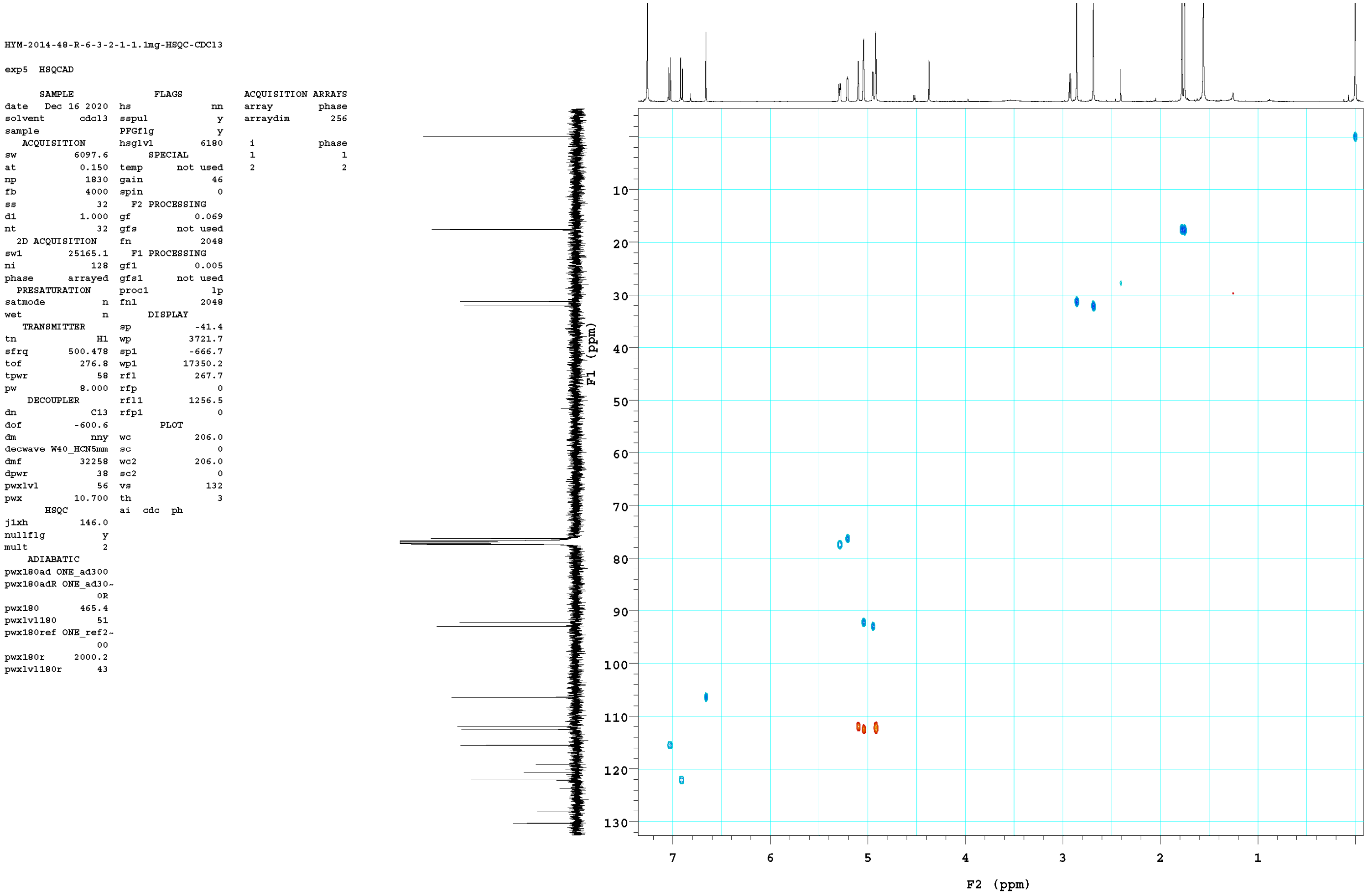

Figure S11. HMBC spectrum of **2** (measured in $CDCl_3$, 500 MHz).

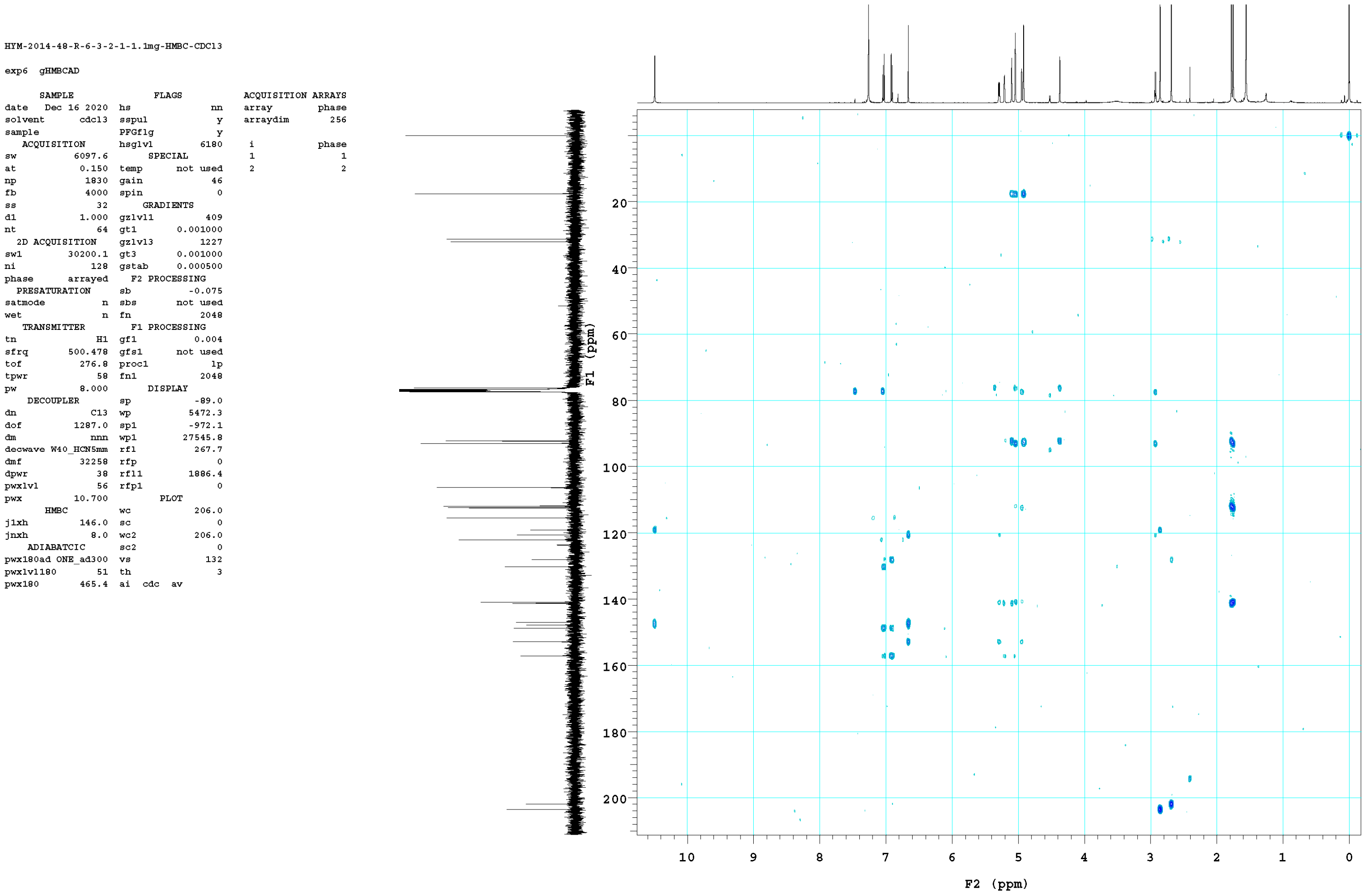

Figure S12. NOESY spectrum of **2** (measured in $CDCl_3$, 500 MHz).

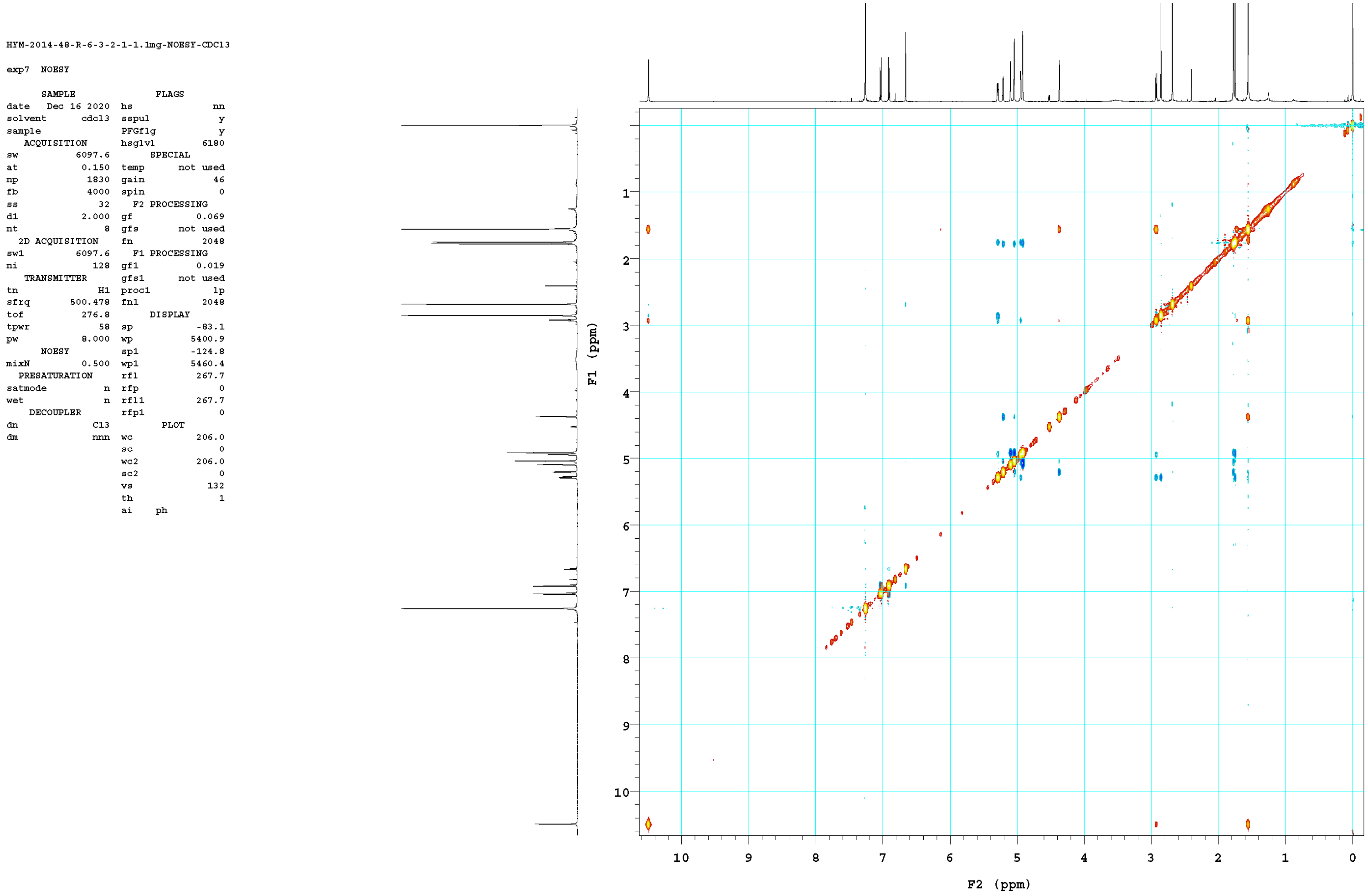

Figure S13. ^{1}H NMR spectrum of **3** (measured in $CDCl_3$, 500 MHz).

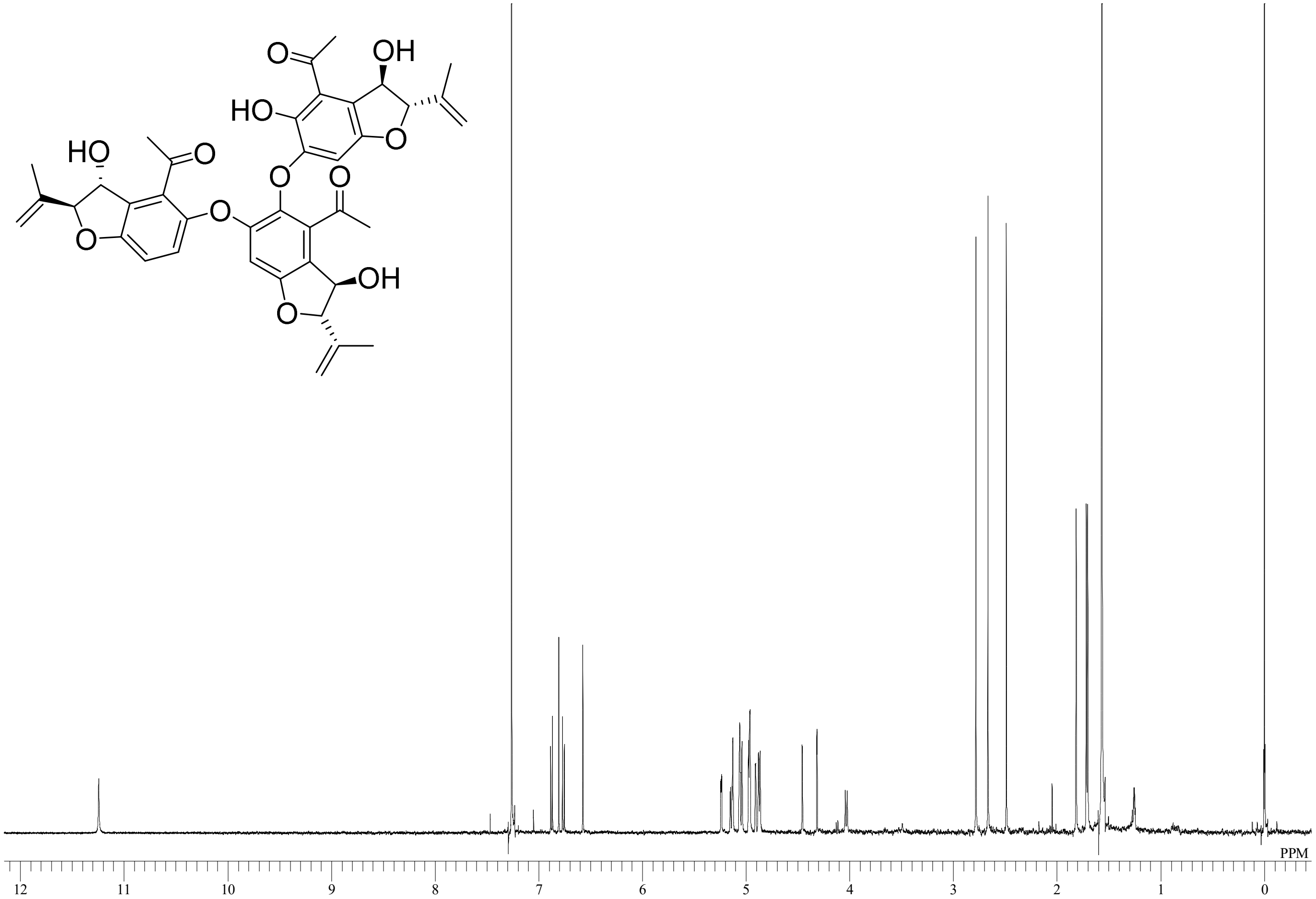

Figure S14. ^{13}C NMR spectrum of **3** (measured in $CDCl_3$, 126 MHz).

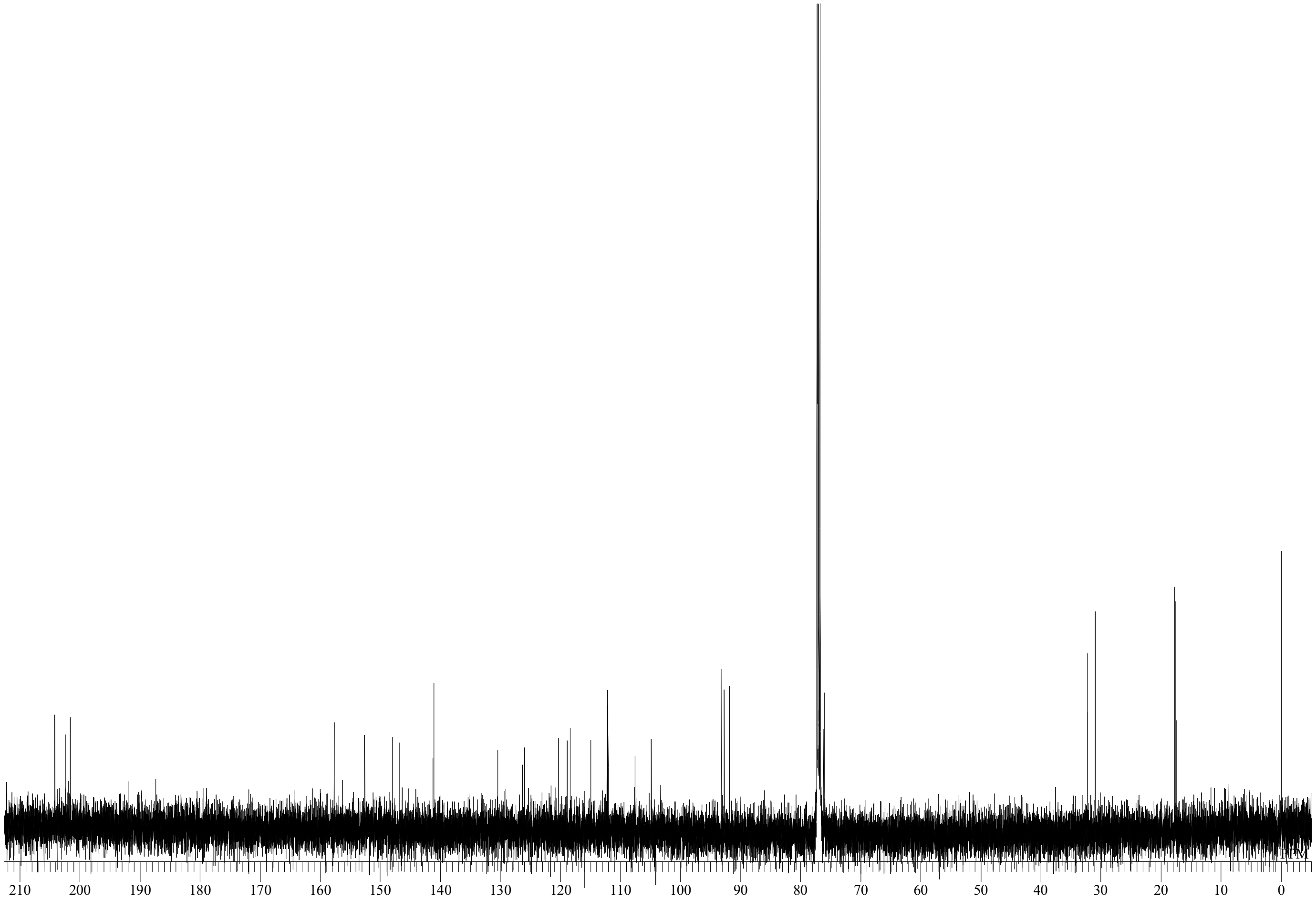

Figure S15. ^1H-^1H COSY spectrum of **3** (measured in $CDCl_3$, 500 MHz).

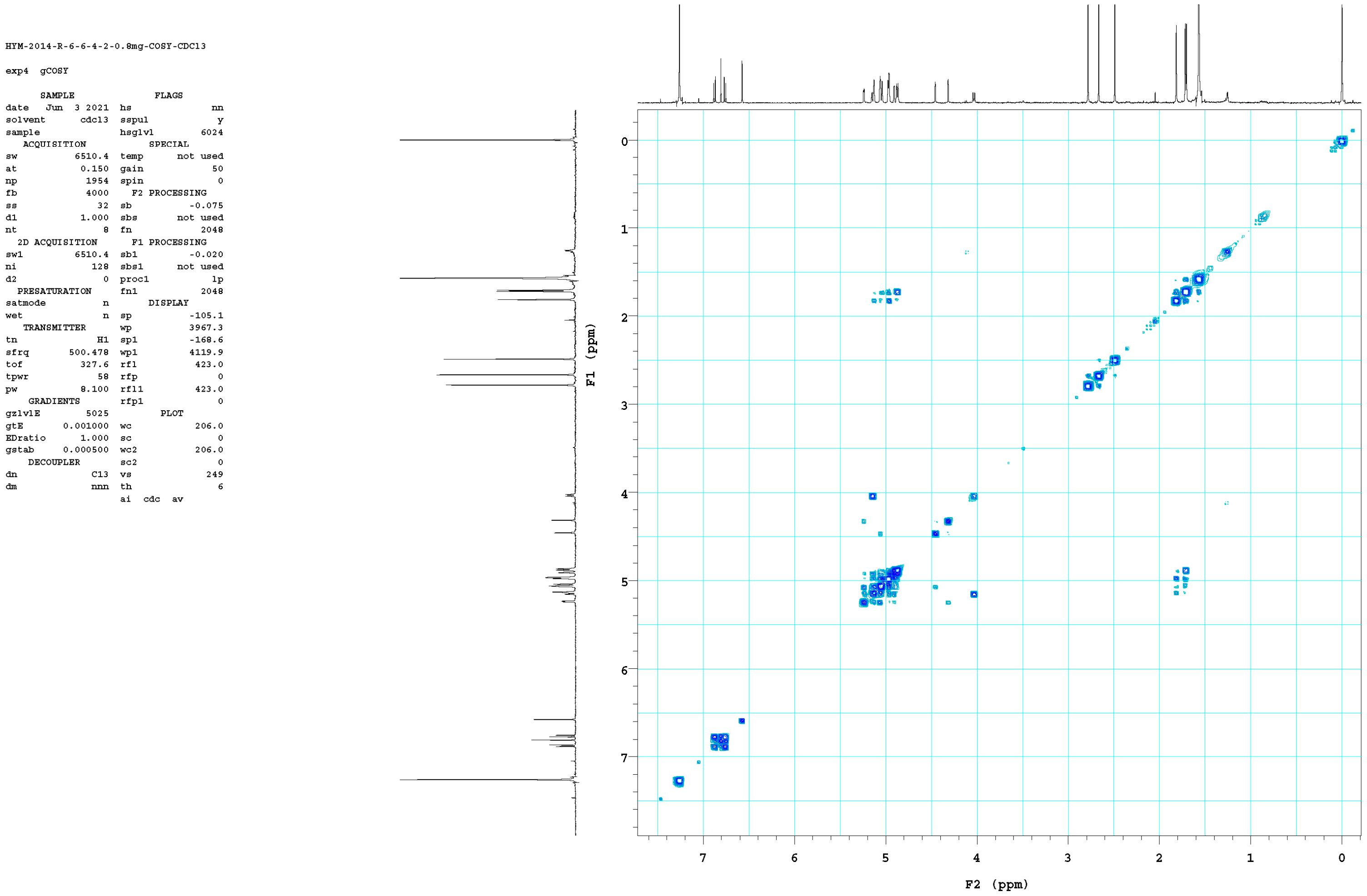

Figure S16. HSQC spectrum of **3** (measured in $CDCl_3$, 500 MHz).

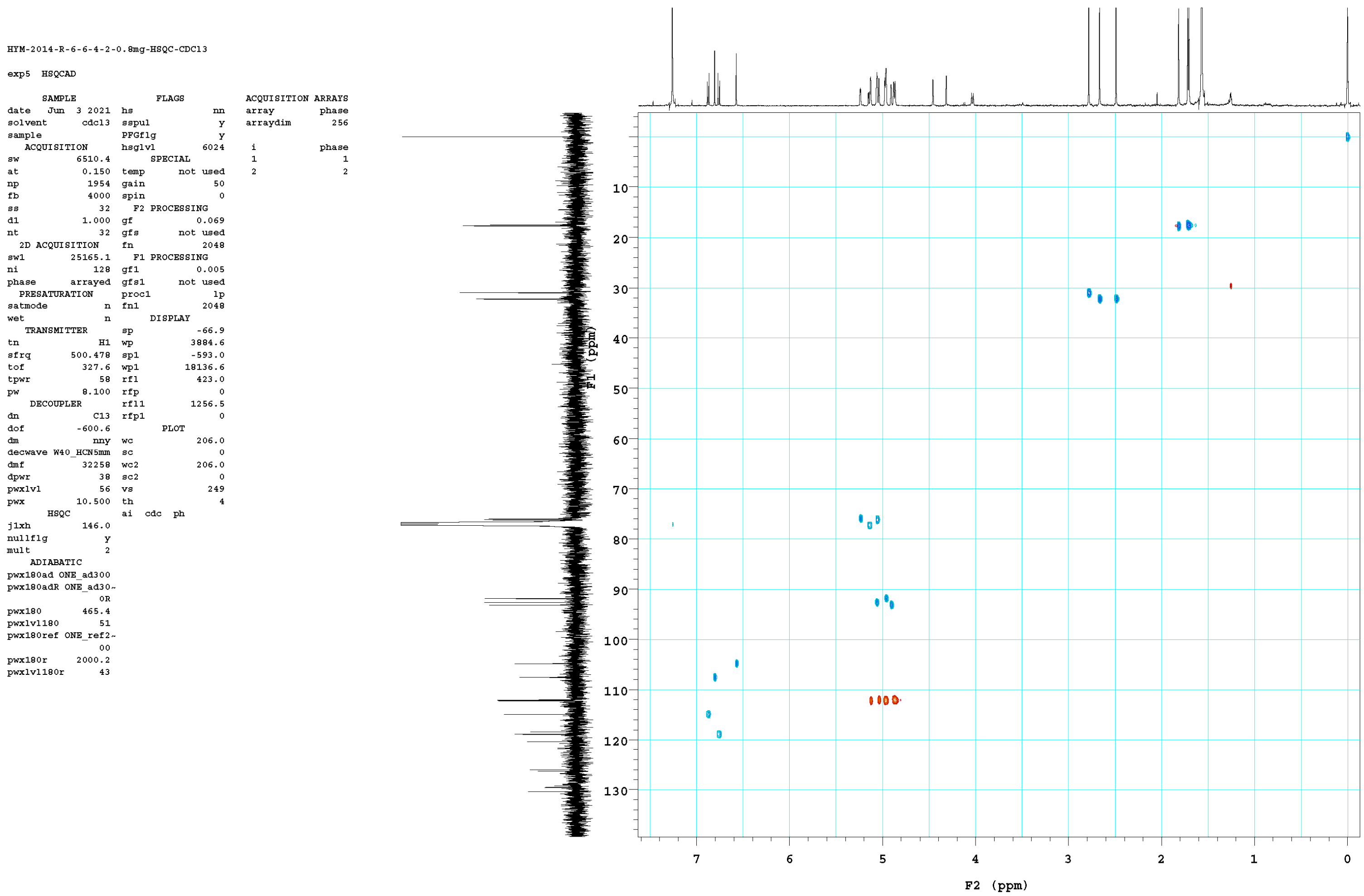

Figure S17. HMBC spectrum of **3** (measured in $CDCl_3$, 500 MHz).

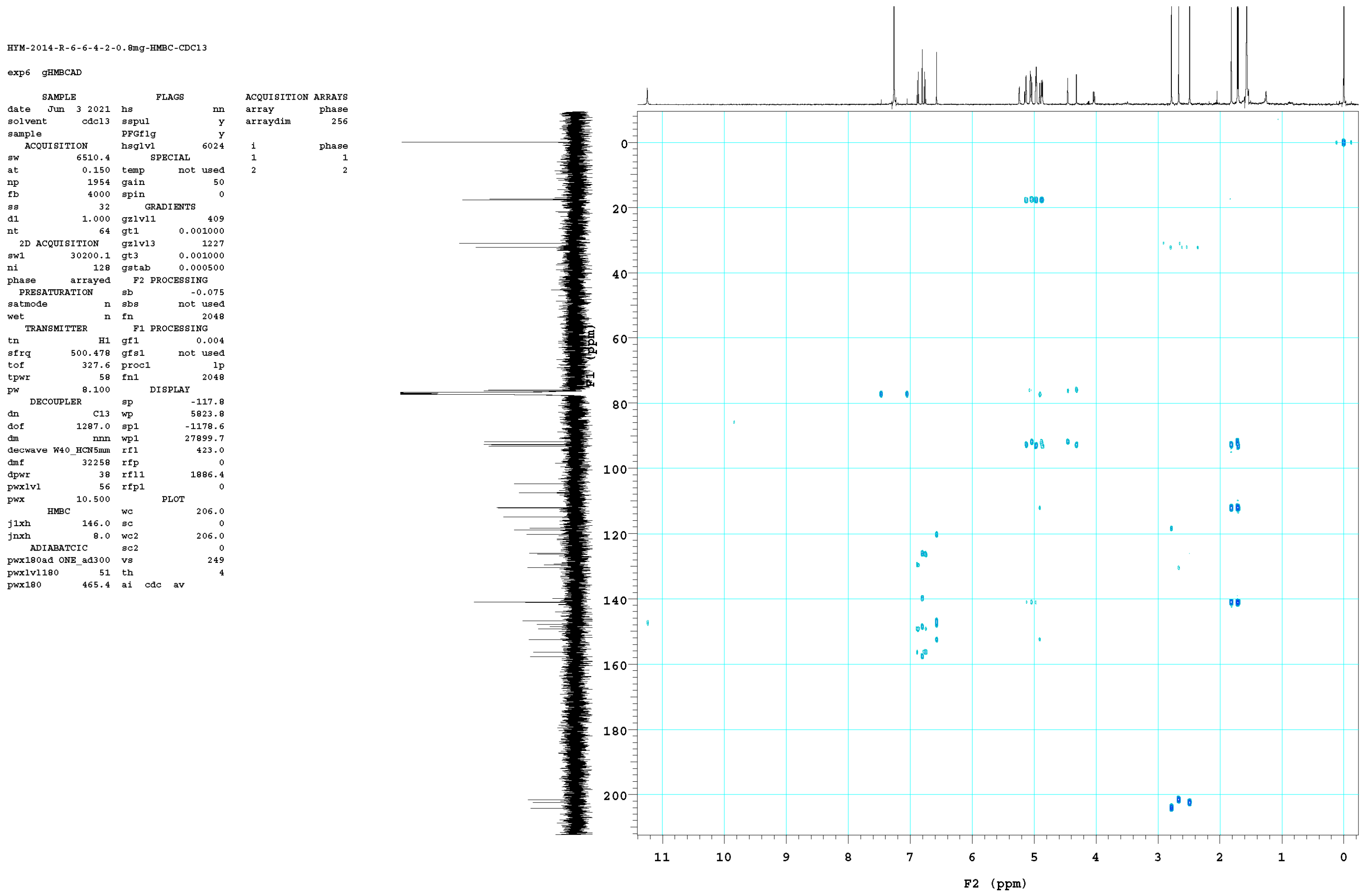

Figure S18. NOESY spectrum of **3** (measured in $CDCl_3$, 500 MHz).

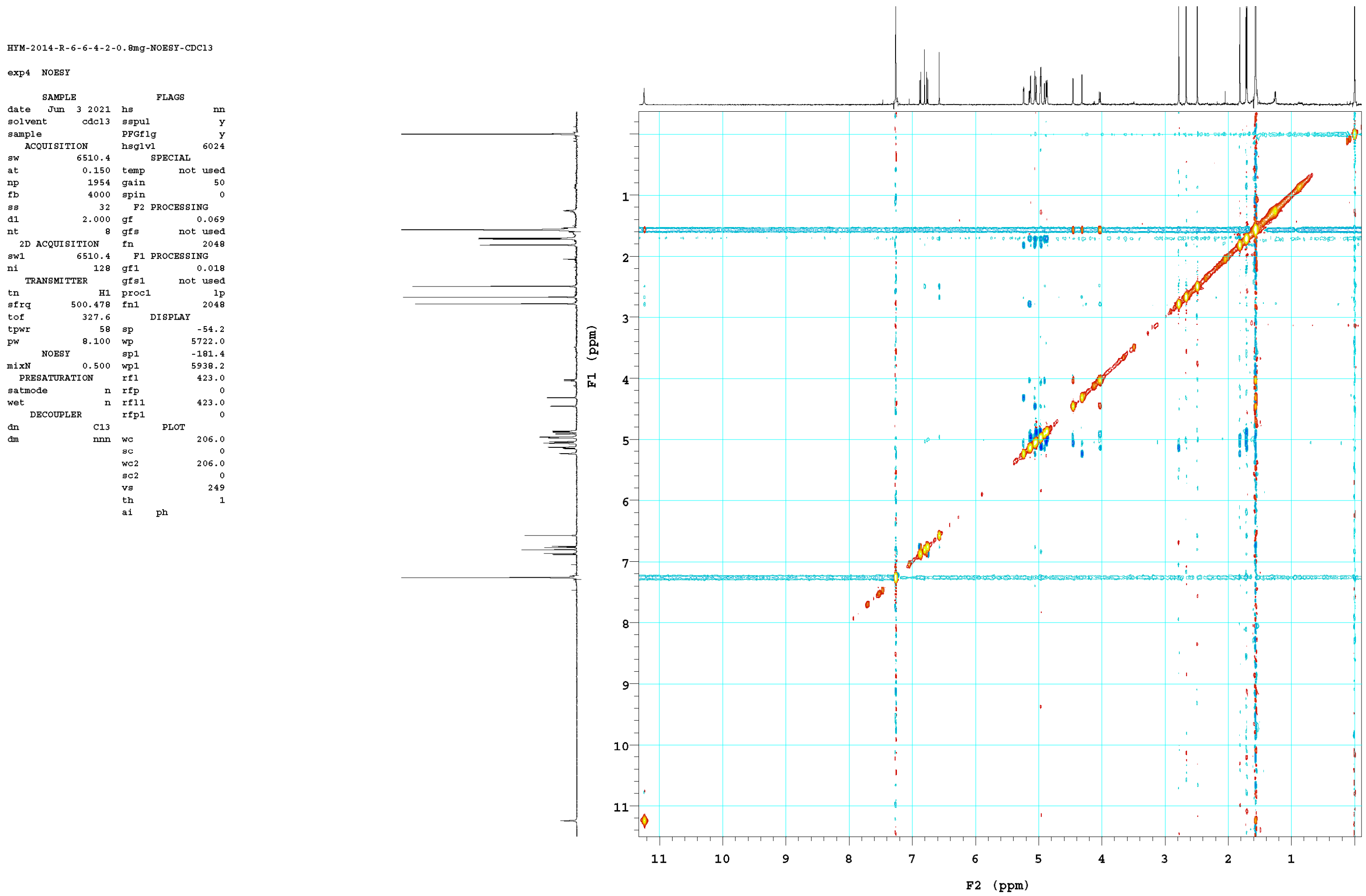

Figure S19. Experimental ECD spectrum of **29** and calculated ECD spectra of 2*S*,3*R*-**29** and 2*R*,3*R*-**29**.

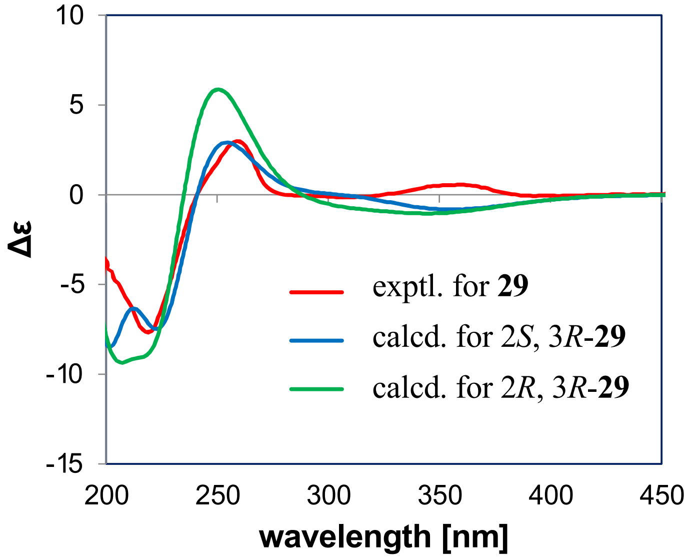

Calculated ECD spectra of 2*S*,3*R*-**29** and 2*R*,3*R*-**29** are very similar to each other. The Cotton effect around 350 nm is derived from torsion angle between benzofuran and olefin moieties (see Figure S20). Disagreement on this band between experimental and calculated ECD spectra may be due to the presence of some intermolecular interaction.

Figure S20. Optimized structures (B3LYP/6-31G(d,p) in MeOH (PCM)) and calculated ECD spectra (CAM-B3LYP/6-31G(d,p) in MeOH (PCM)) for each conformer of **29** (**29A**–**29F**).

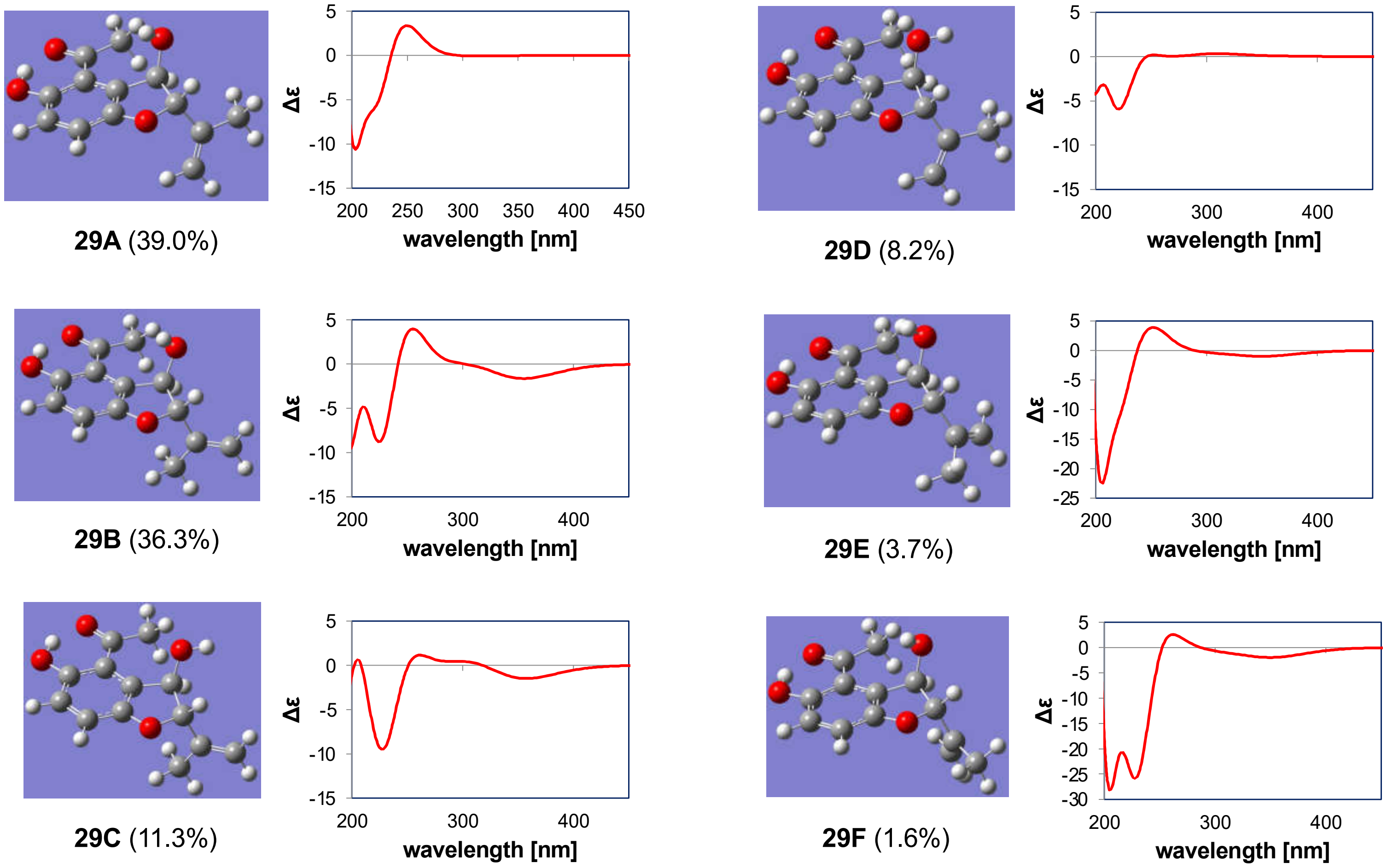

Figure S21. ^{1}H NMR spectrum of **4** (measured in $CDCl_3$, 500 MHz).

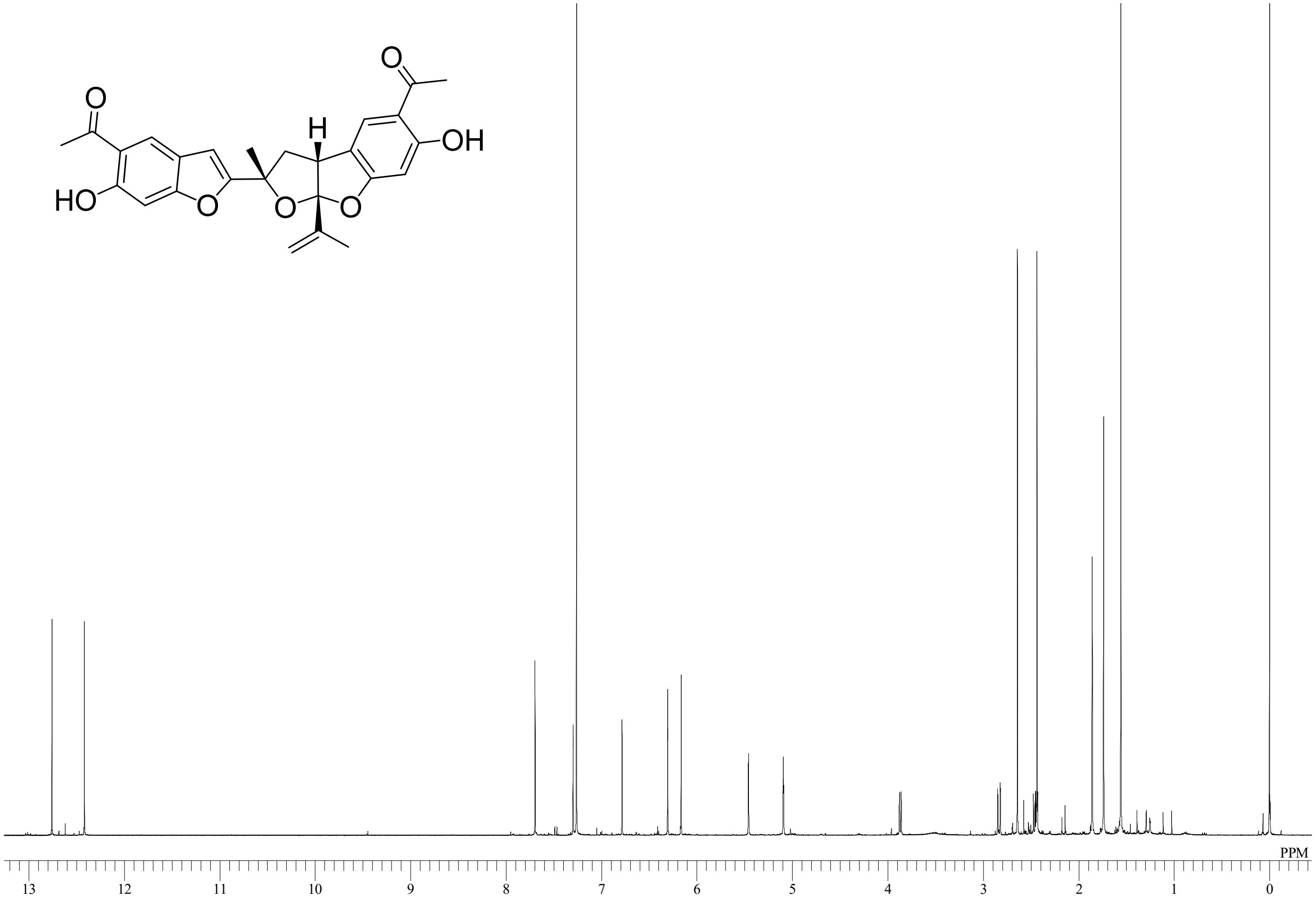

Figure S22. ^{13}C NMR spectrum of **4** (measured in $CDCl_3$, 126 MHz).

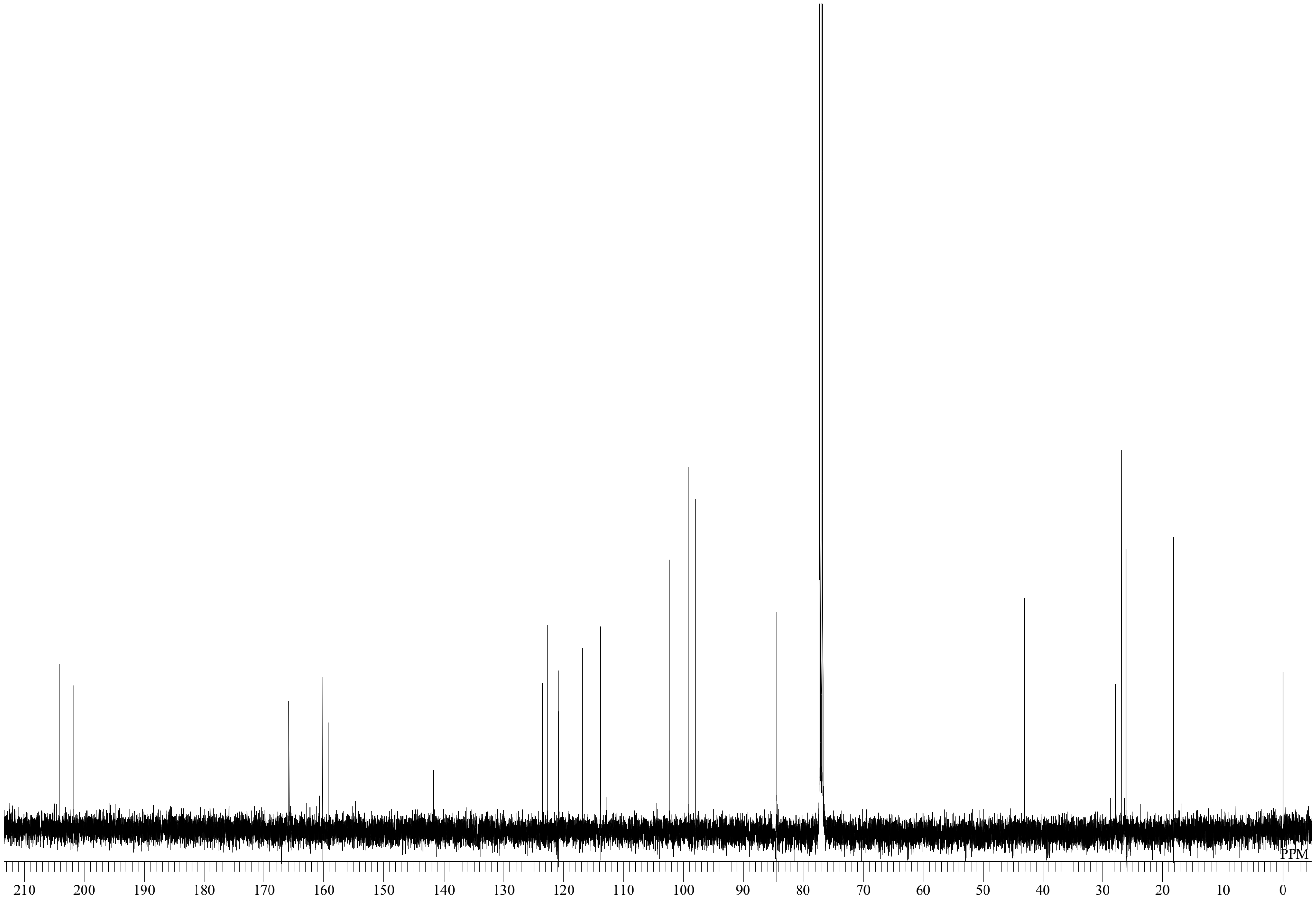

Figure S23. ^{1}H-^{1}H COSY spectrum of **4** (measured in $CDCl_3$, 500 MHz).

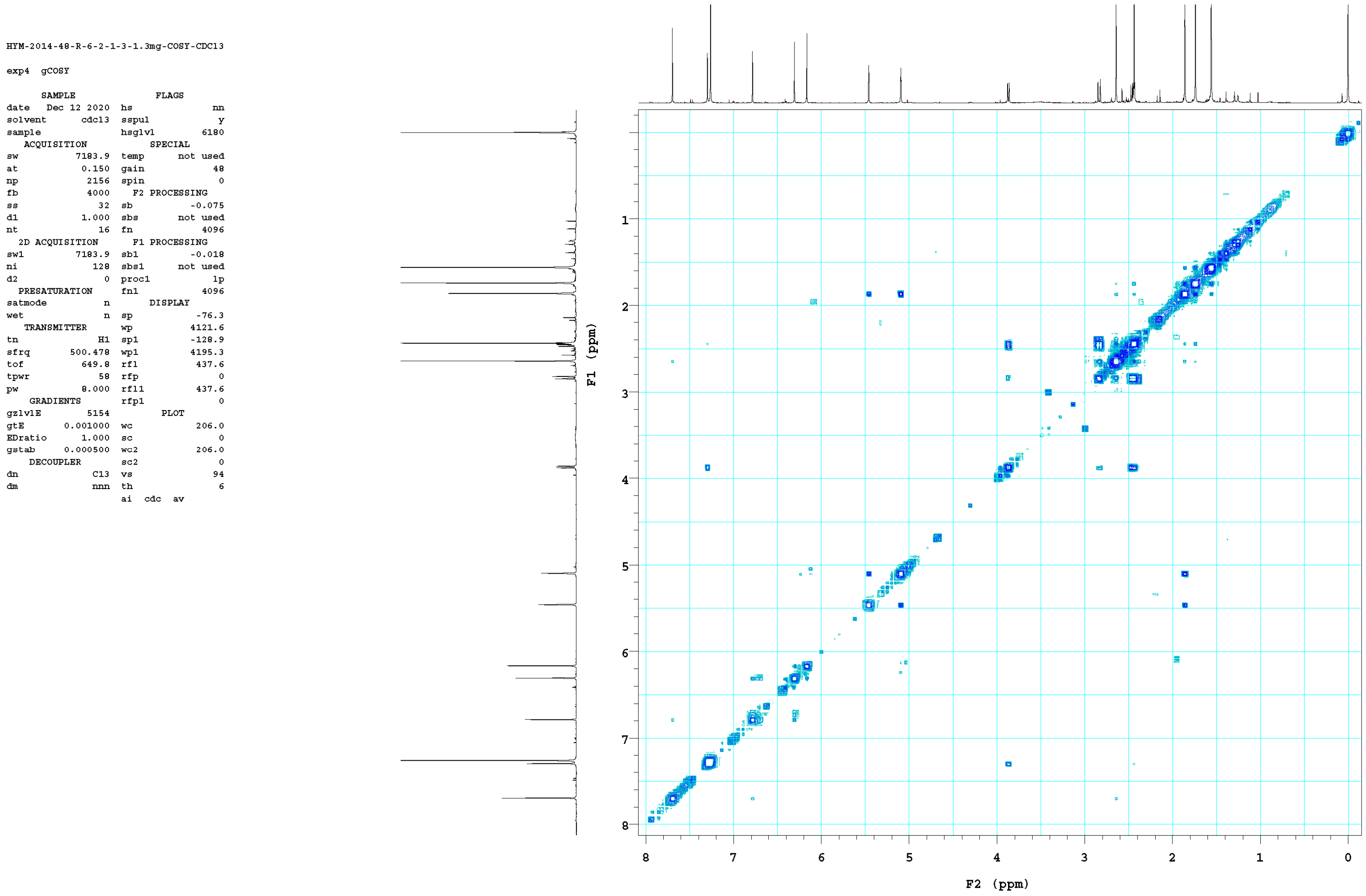

Figure S24. HSQC spectrum of **4** (measured in $CDCl_3$, 500 MHz).

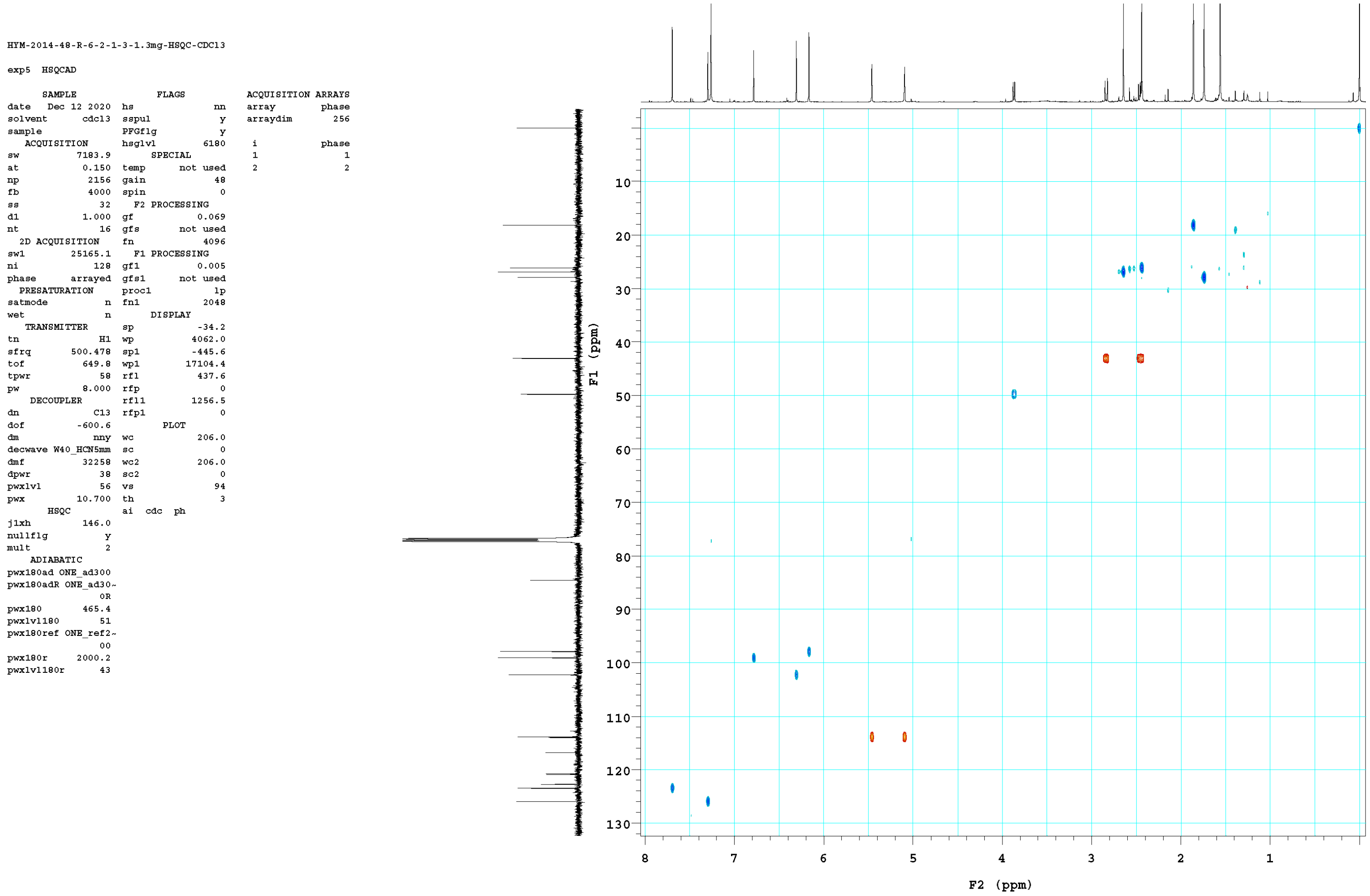

Figure S25. HMBC spectrum of **4** (measured in $CDCl_3$, 500 MHz).

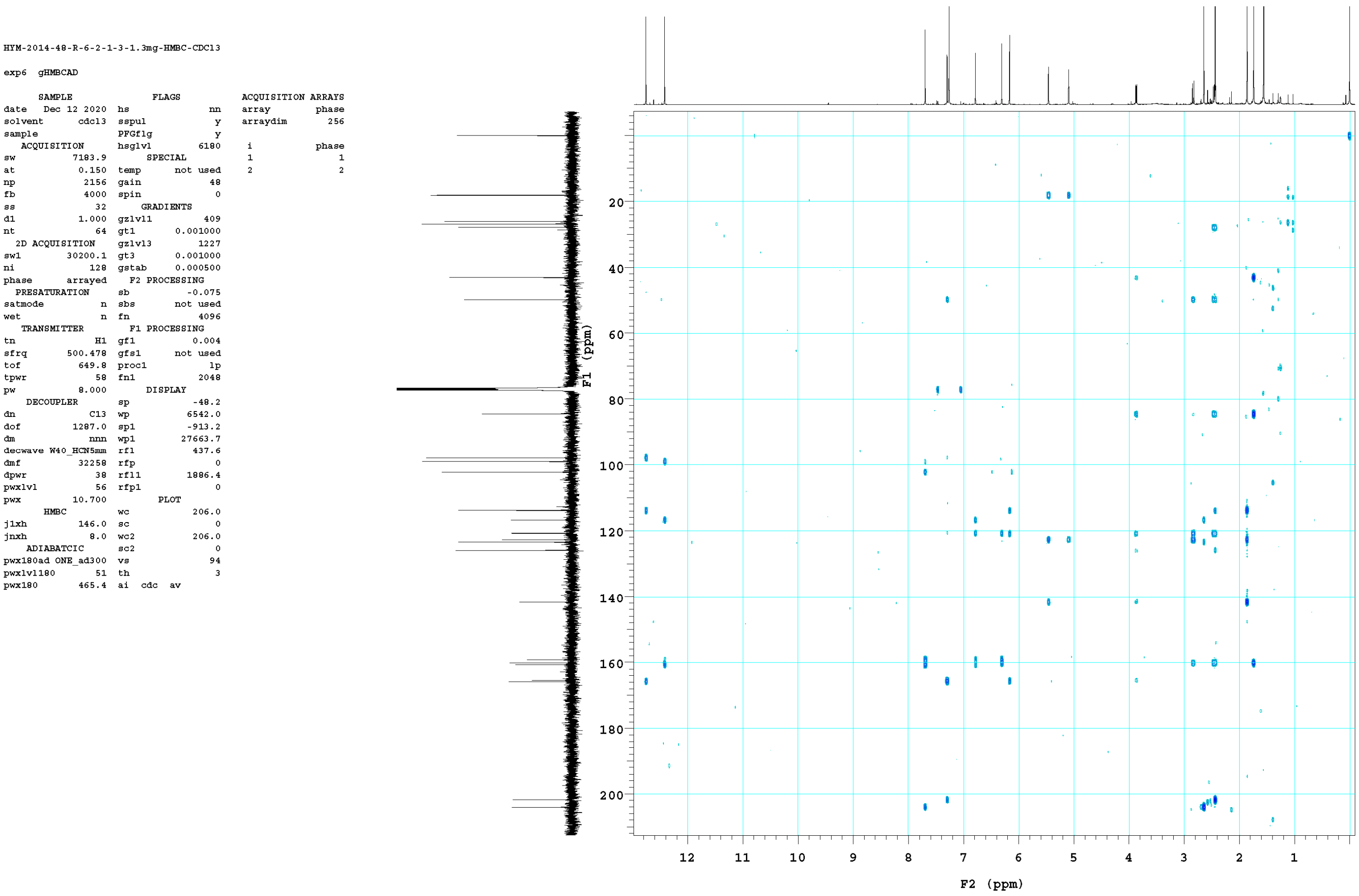

Figure S26. NOESY spectrum of **4** (measured in $CDCl_3$, 500 MHz).

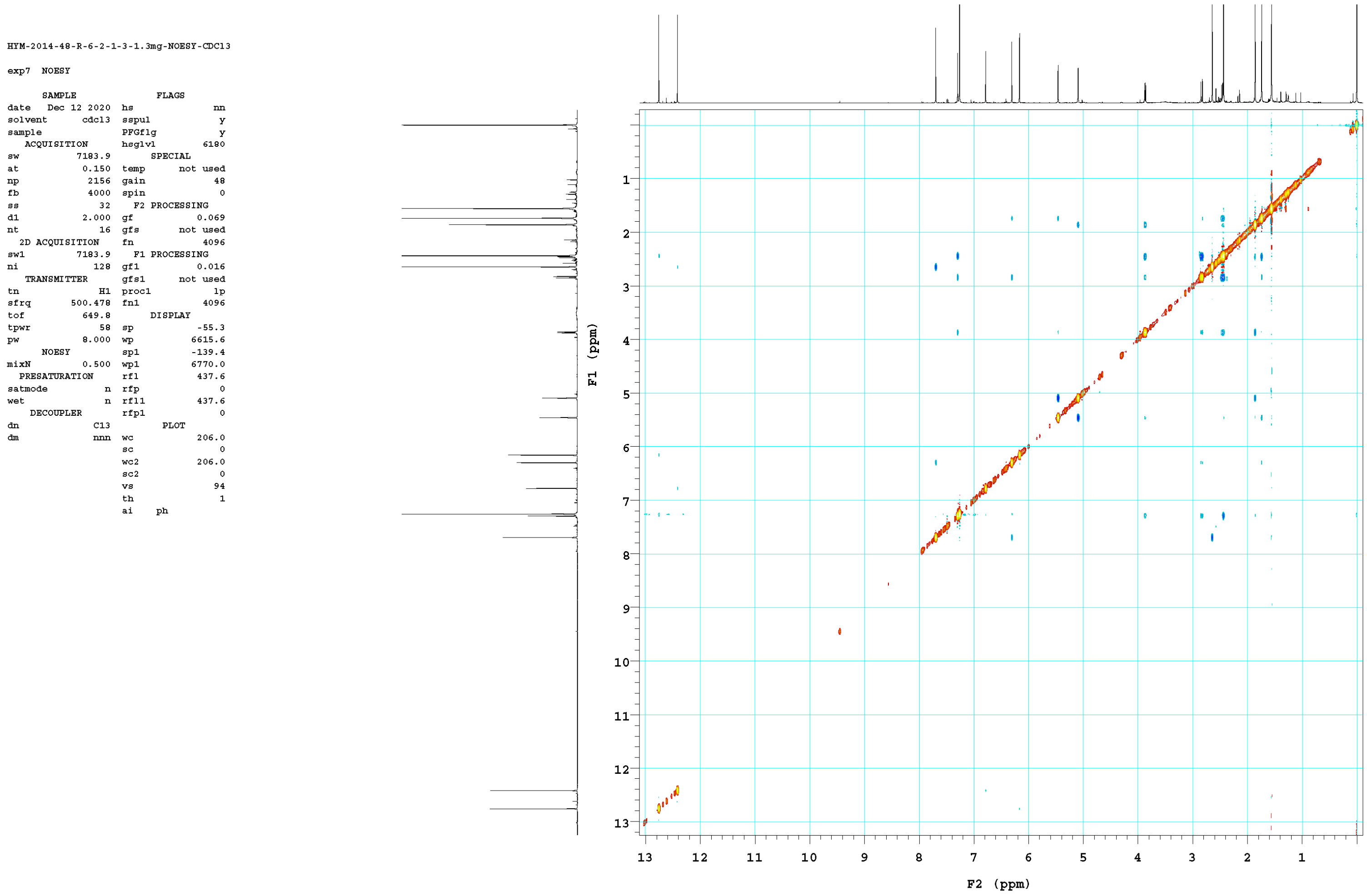

Figure S27. ^{1}H NMR spectrum of **5** (measured in $CDCl_3$, 500 MHz).

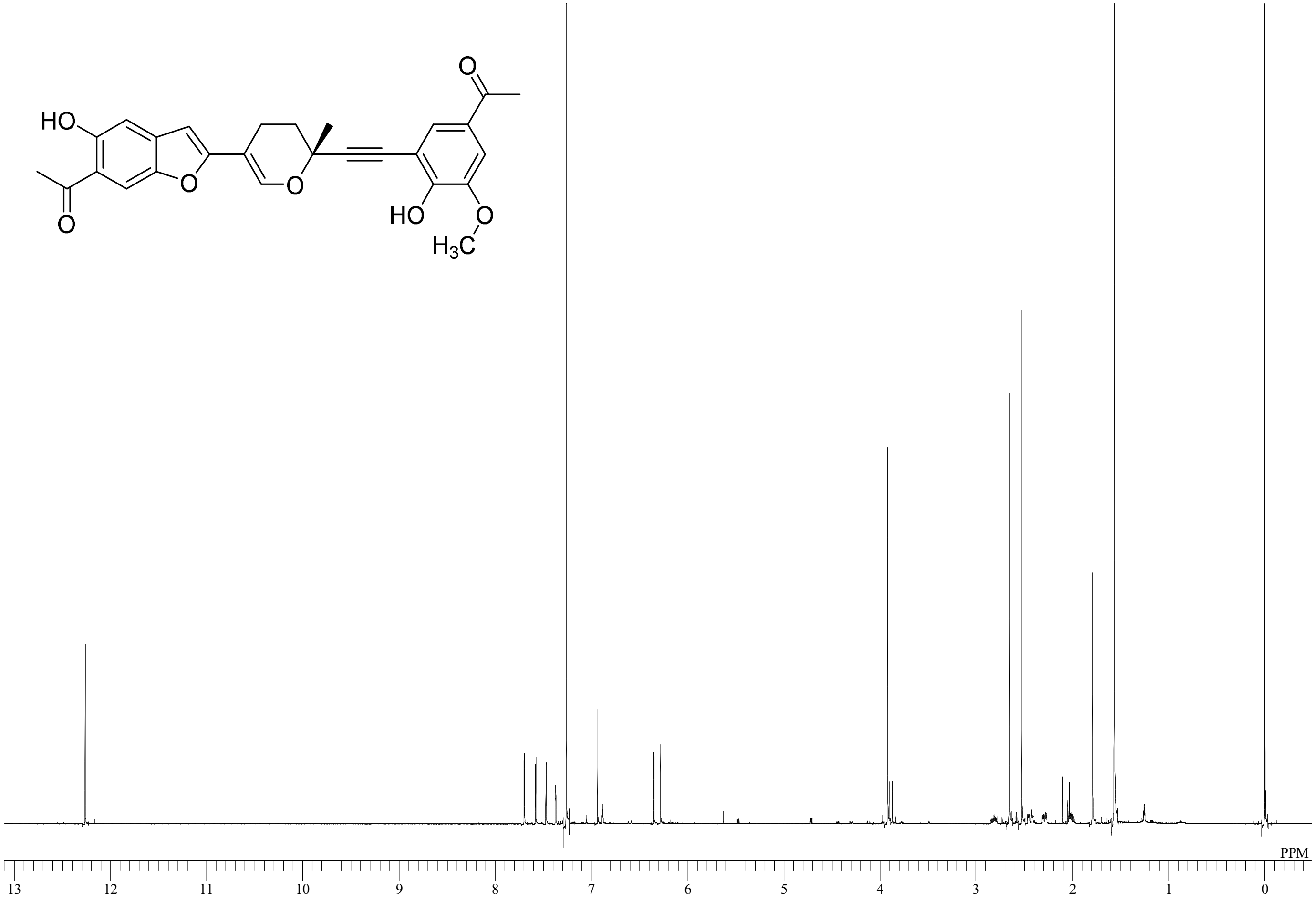

Figure S28. ^{13}C NMR spectrum of **5** (measured in $CDCl_3$, 126 MHz).

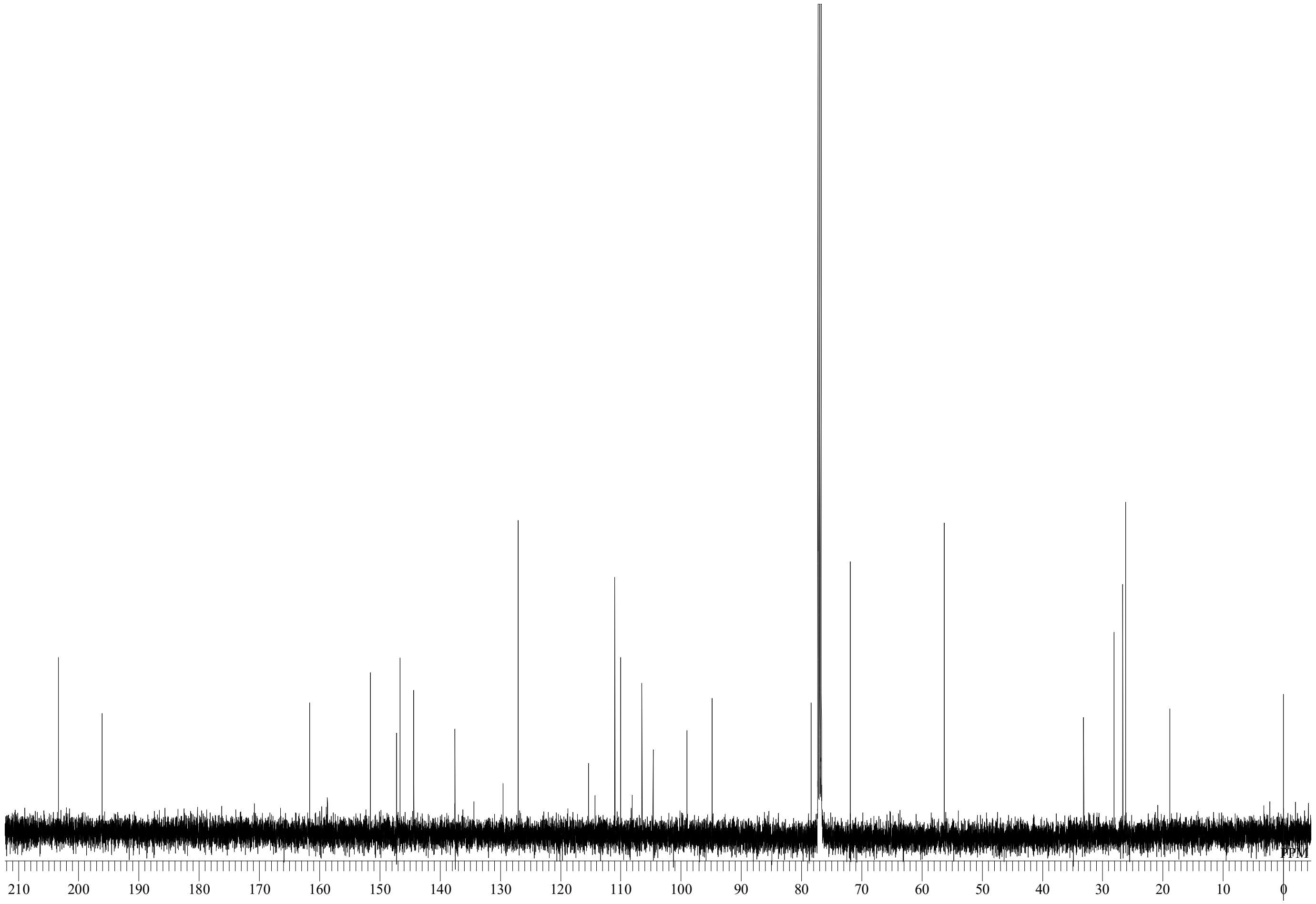

Figure S29. ^{1}H-^{1}H COSY spectrum of **5** (measured in $CDCl_3$, 500 MHz).

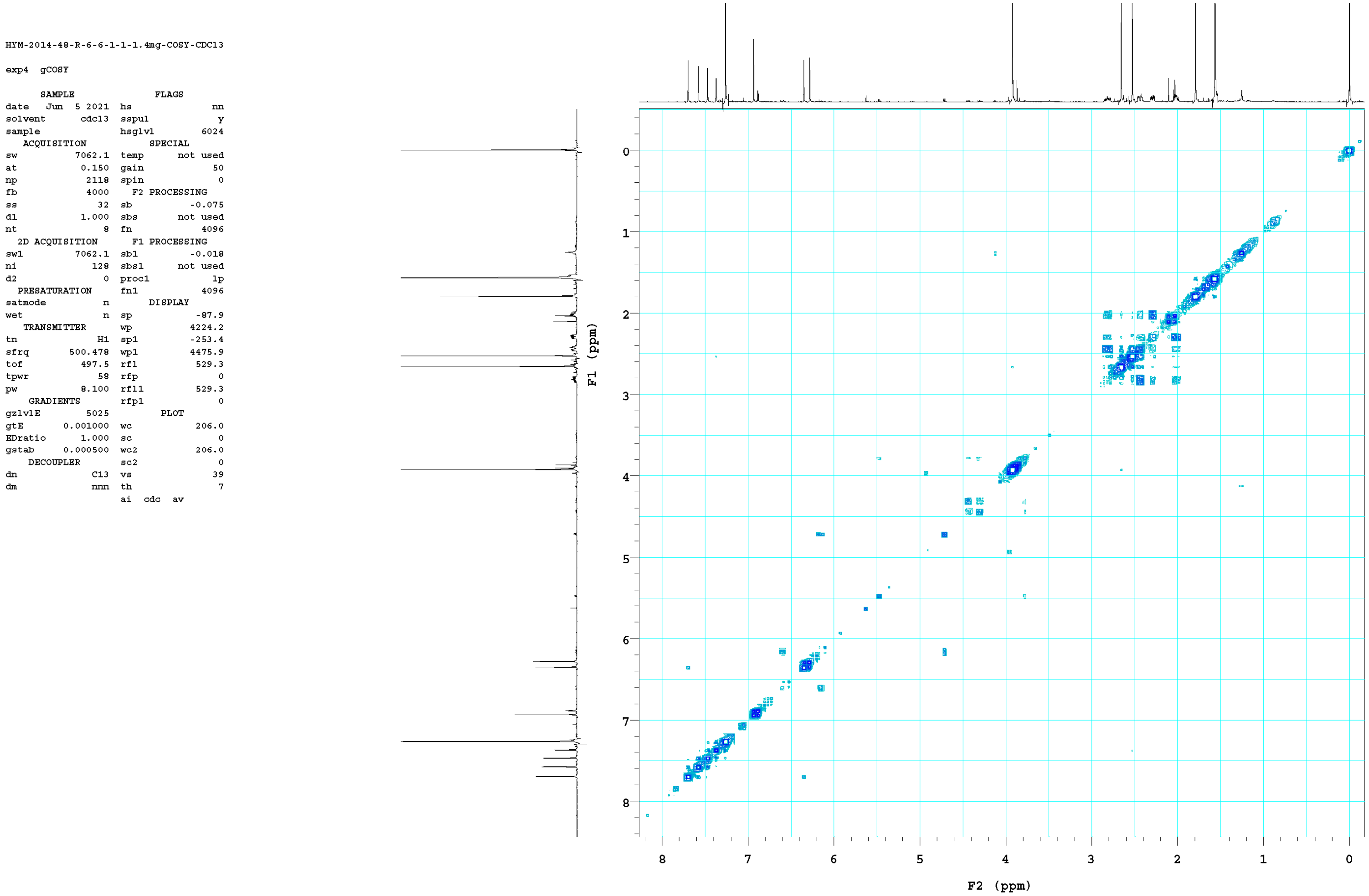

Figure S30. HSQC spectrum of **5** (measured in $CDCl_3$, 500 MHz).

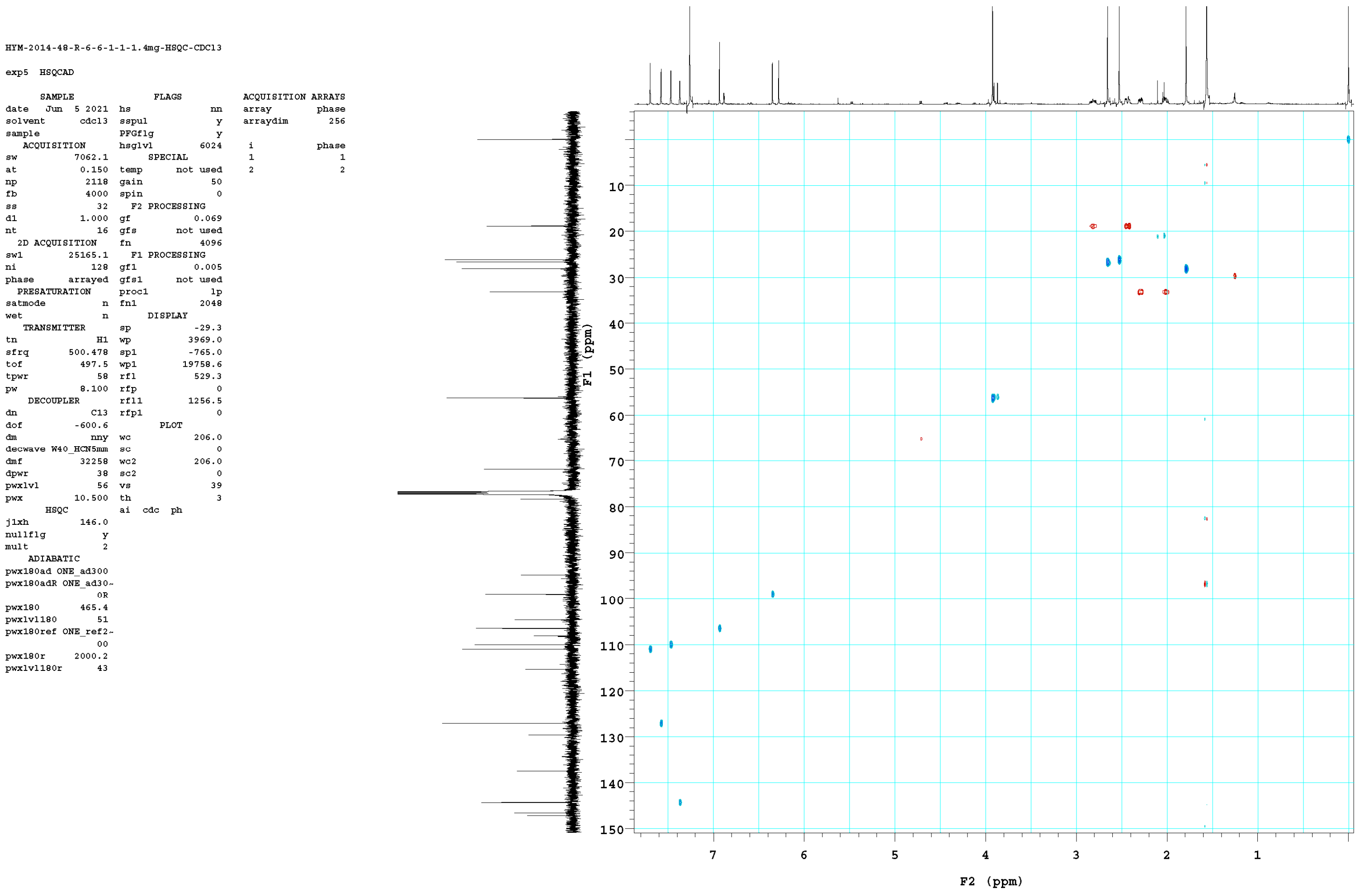

Figure S31. HMBC spectrum of **5** (measured in $CDCl_3$, 500 MHz).

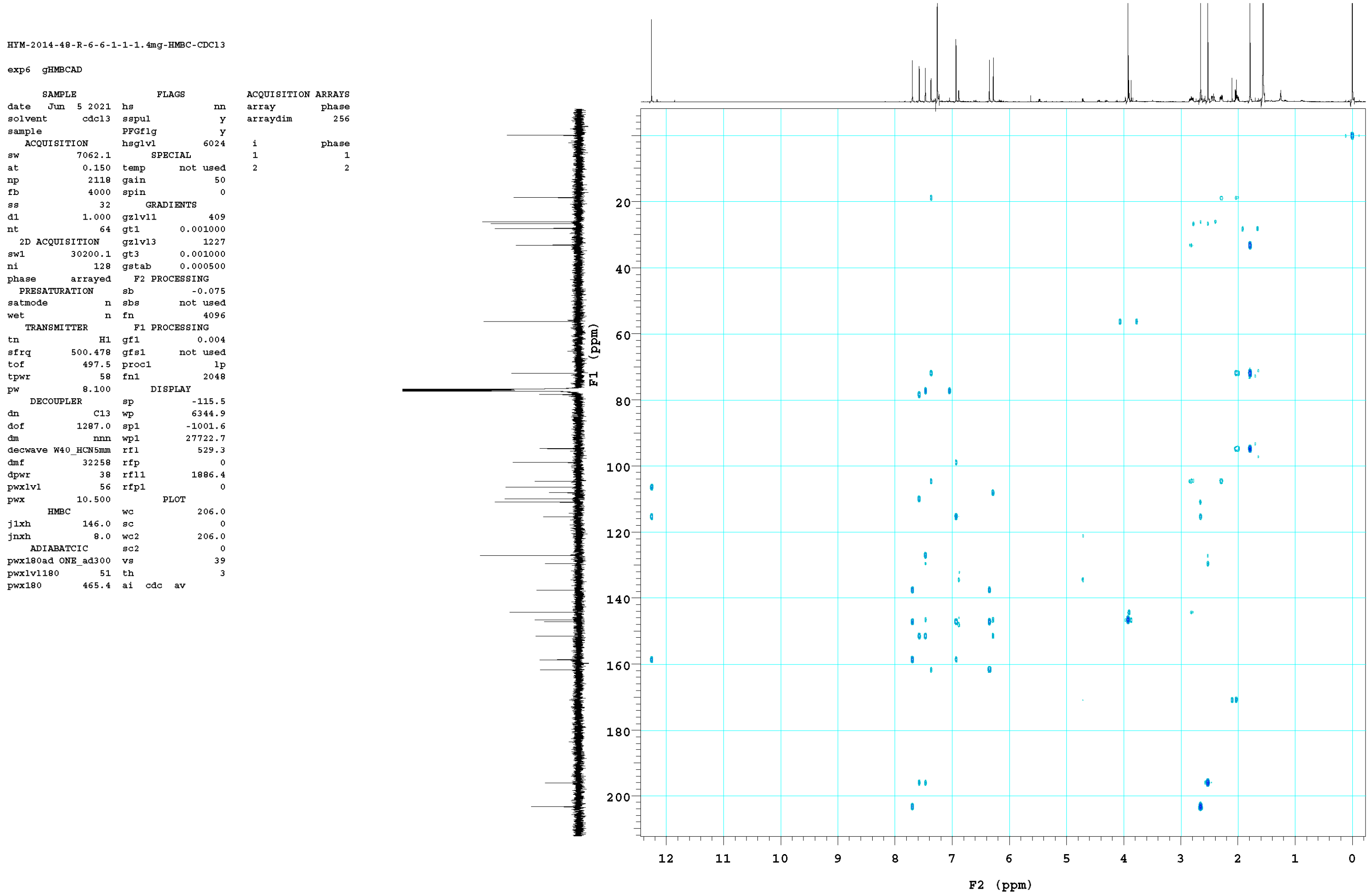

Figure S32. NOESY spectrum of **5** (measured in $CDCl_3$, 500 MHz).

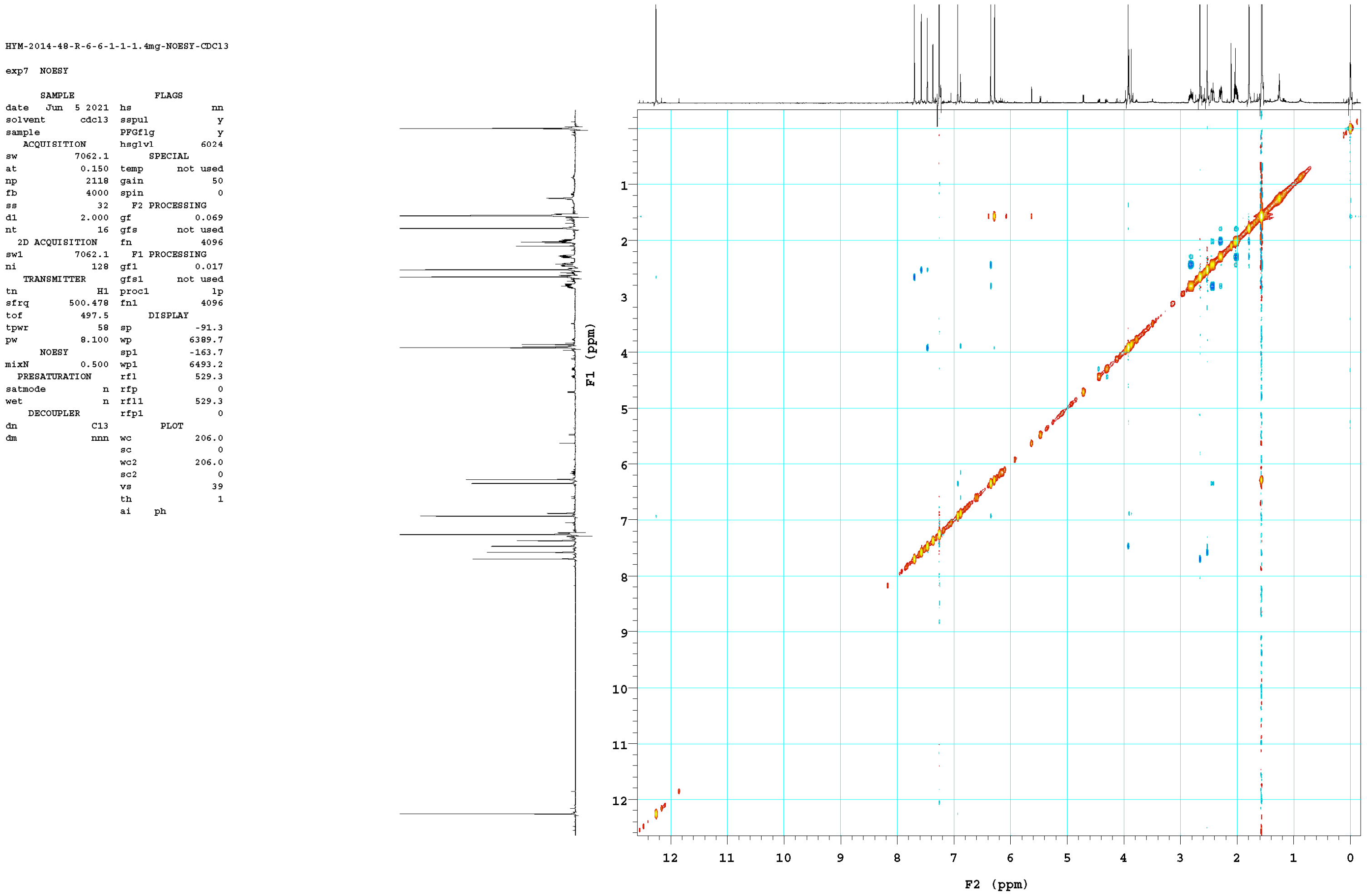

Figure S33. Experimental and calculated ECD spectra and chiral HPLC analysis of **4** and **5**.

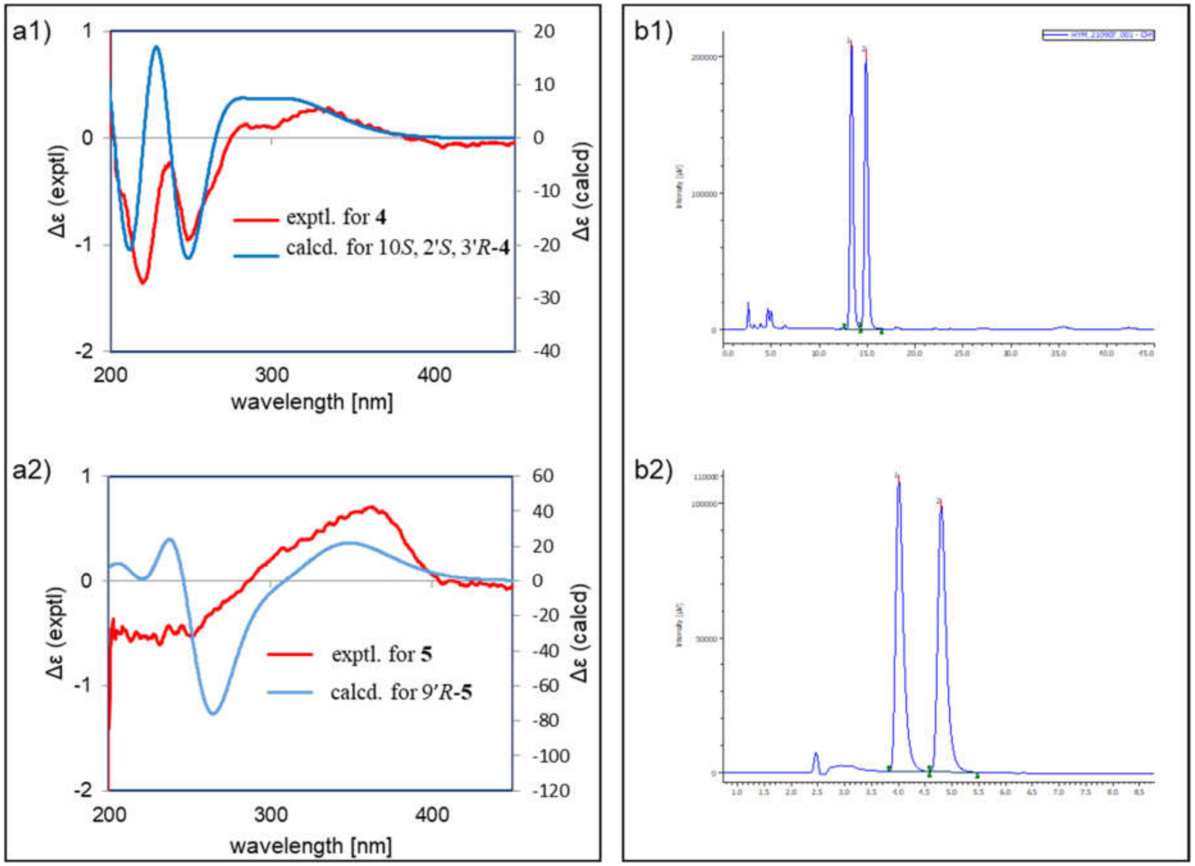

a) Experimental and calculated ECD spectra of **4** (a1, $c = 5.98 \times 10^{-5}$ mol/L in MeOH) and **5** (a2, $c = 4.87 \times 10^{-5}$ mol/L in MeOH). Left and right axes represent Δε values of experimental and calculated spectra, respectively.; b) Chiral HPLC analysis of **4** (b1) and **5** (b2).

Figure S34. ^{1}H NMR spectrum of **14** (measured in $CDCl_3$, 500 MHz).

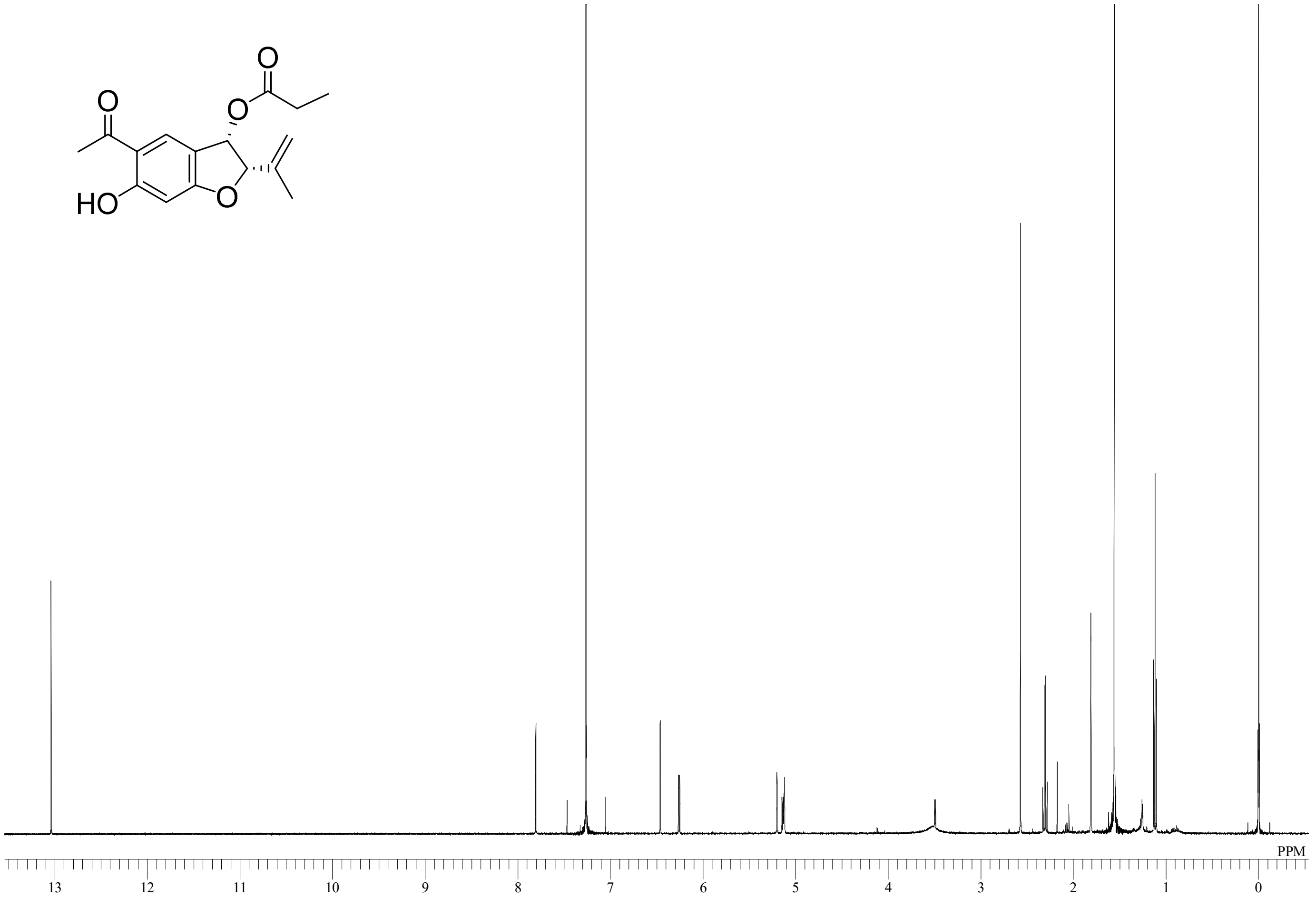

Figure S35. ^{13}C NMR spectrum of **14** (measured in $CDCl_3$, 126 MHz).

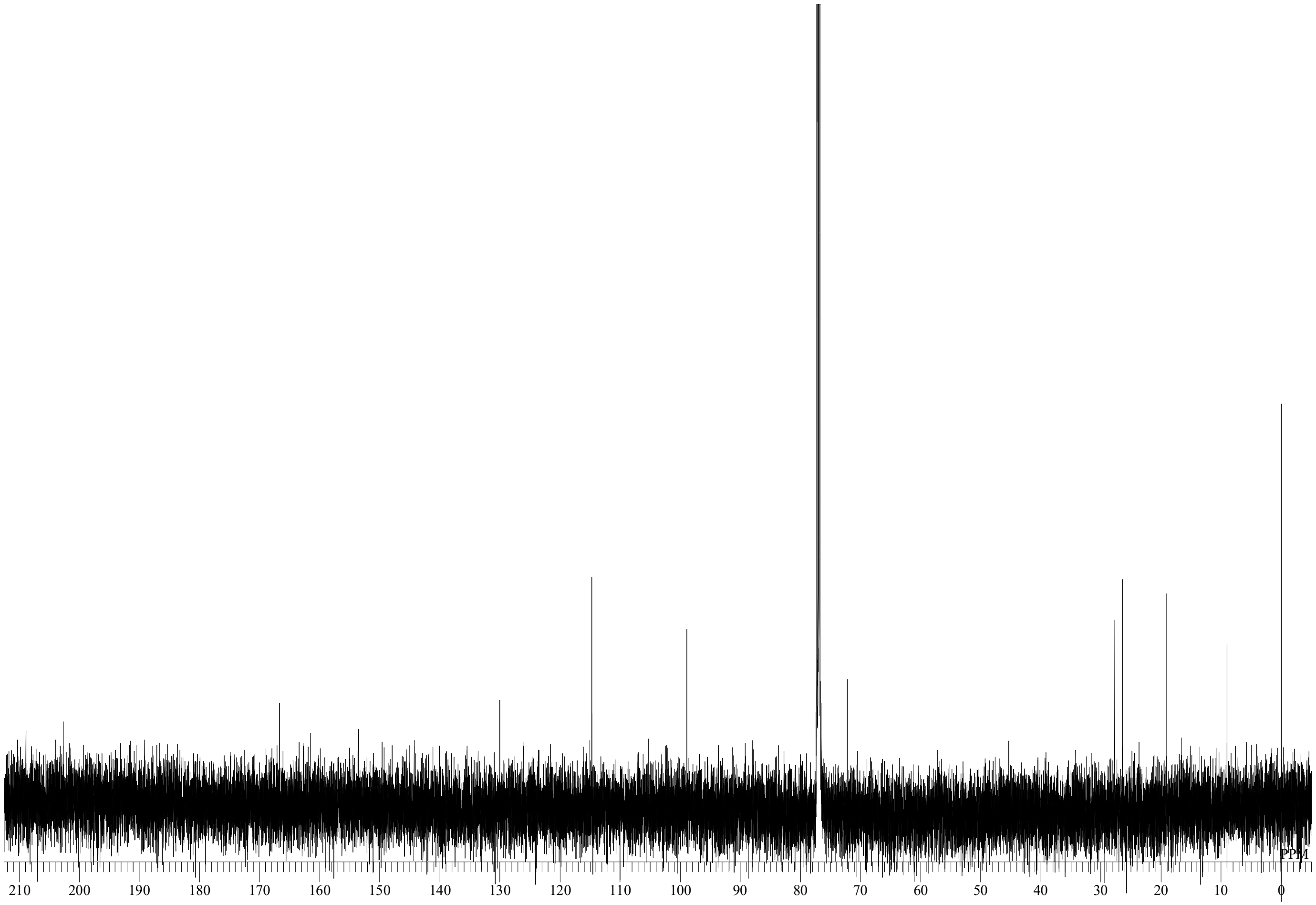

Figure S36. ^1H-^1H COSY spectrum of **14** (measured in $CDCl_3$, 500 MHz).

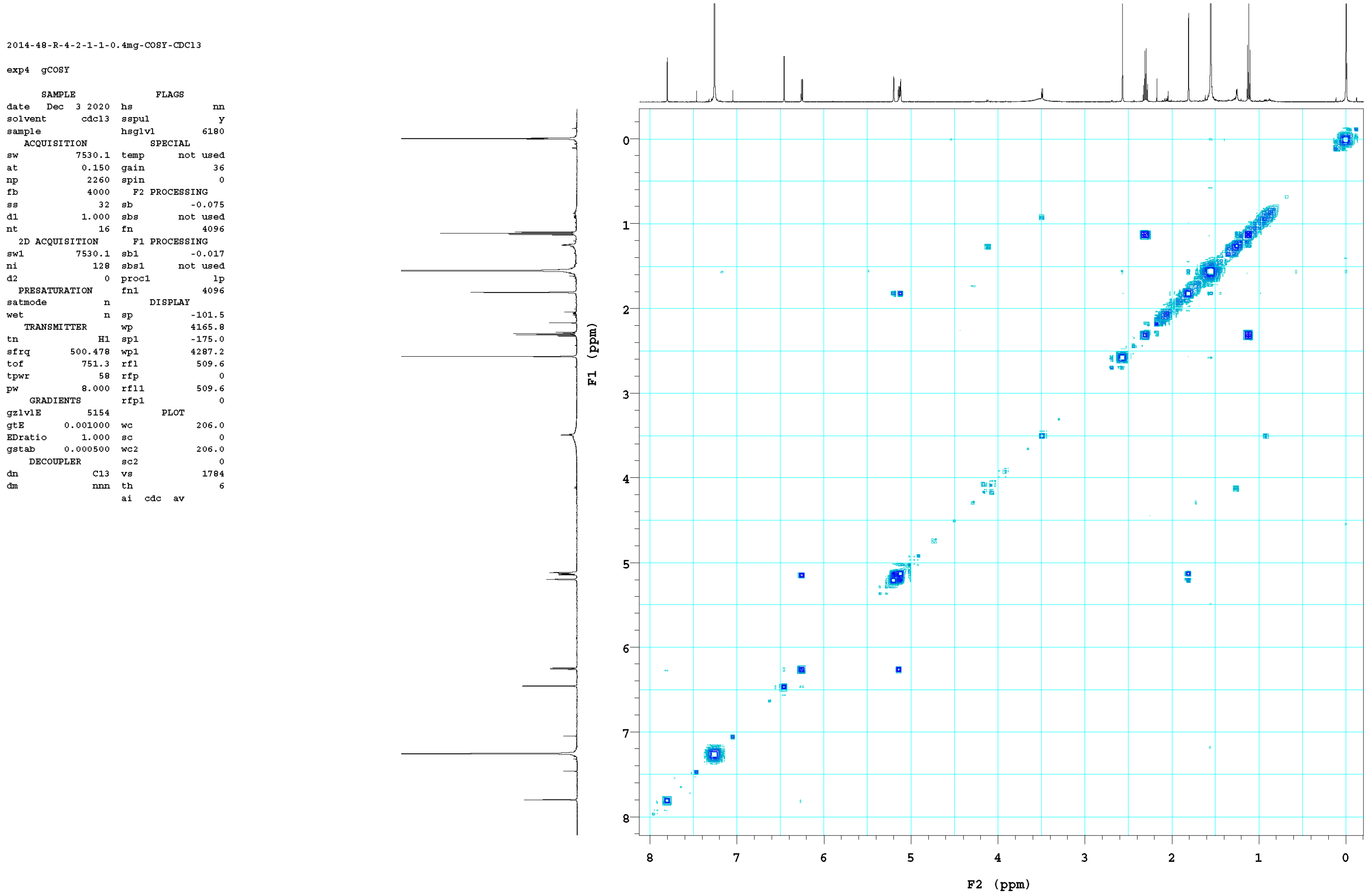

Figure S37. HSQC spectrum of **14** (measured in $CDCl_3$, 500 MHz).

```
HYM-2014-48-R-4-2-1-1-0.4mg-HSQC-CDCl3

exp5  HSQCAD

      SAMPLE               FLAGS           ACQUISITION ARRAYS
date    Dec  3 2020  hs                nn  array          phase
solvent       cdcl3  sspul              y  arraydim         256
sample               PFGflg             y
   ACQUISITION       hsglvl          6180   i             phase
sw           7530.1        SPECIAL          1                 1
at            0.150  temp        not used   2                 2
np             2260  gain              36
fb             4000  spin               0
ss               32    F2 PROCESSING
d1            1.000  gf             0.069
nt               32  gfs         not used
  2D ACQUISITION     fn              4096
sw1         25165.1    F1 PROCESSING
ni              128  gf1            0.005
phase      arrayed   gfs1        not used
  PRESATURATION      proc1             lp
satmode           n  fn1             2048
wet               n        DISPLAY
   TRANSMITTER       sp             -86.8
tn               H1  wp            4202.6
sfrq        500.478  sp1          -1010.8
tof           751.3  wp1          18529.8
tpwr             58  rfl            509.6
pw            8.000  rfp                0
    DECOUPLER        rfl1          1256.5
dn              C13  rfp1               0
dof          -600.6           PLOT
dm              nny  wc             206.0
decwave W40_HCN5mm   sc                 0
dmf           32258  wc2            206.0
dpwr             38  sc2                0
pwxlvl           56  vs              1784
pwx          10.700  th                 4
       HSQC          ai   cdc   ph
j1xh          146.0
nullflg           y
mult              2
    ADIABATIC
pwx180ad ONE_ad300
pwx180adR ONE_ad30~
                 0R
pwx180        465.4
pwxlvl180        51
pwx180ref ONE_ref2~
                 00
pwx180r      2000.2
pwxlvl180r       43
```

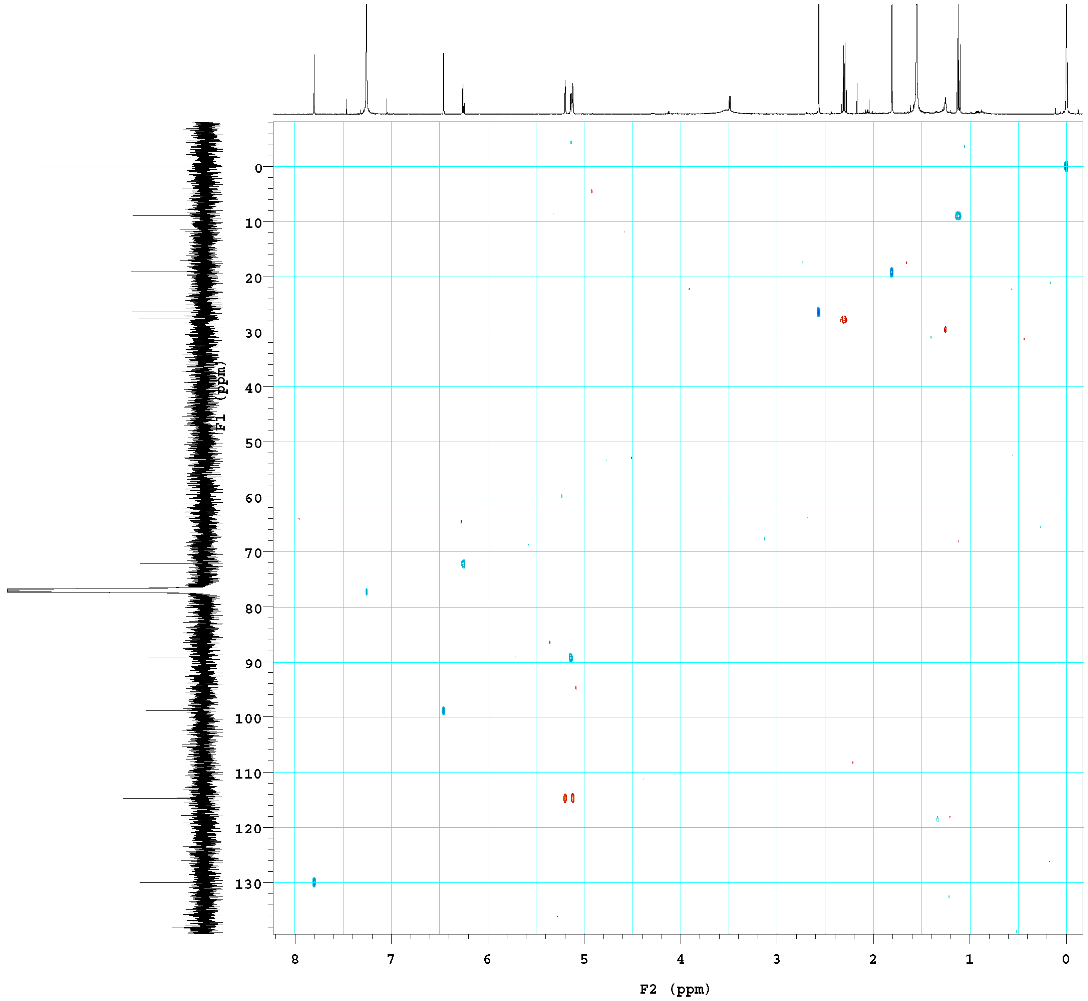

Figure S38. HMBC spectrum of **14** (measured in $CDCl_3$, 500 MHz).

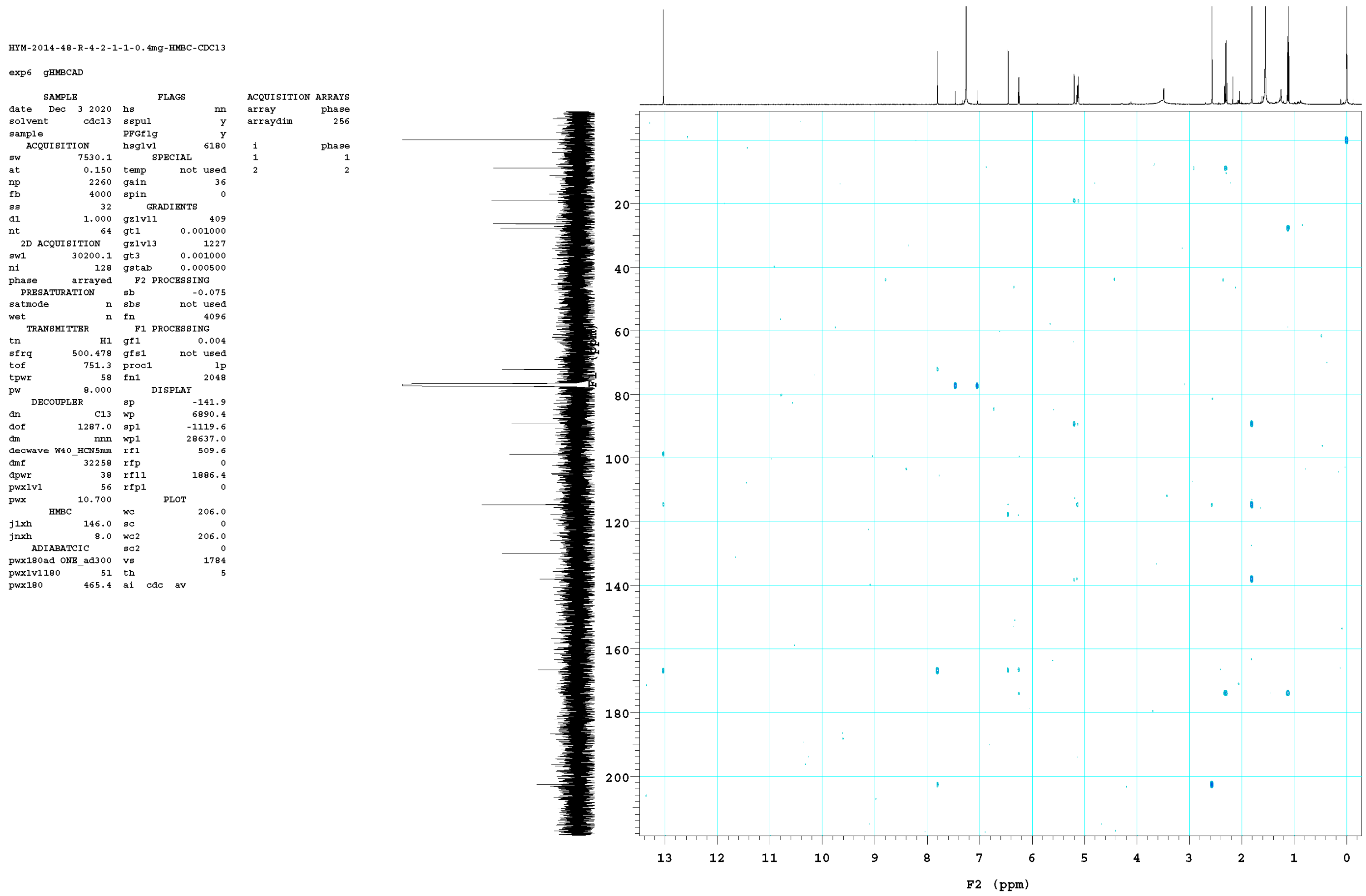

Figure S39. NOESY spectrum of **14** (measured in $CDCl_3$, 500 MHz).

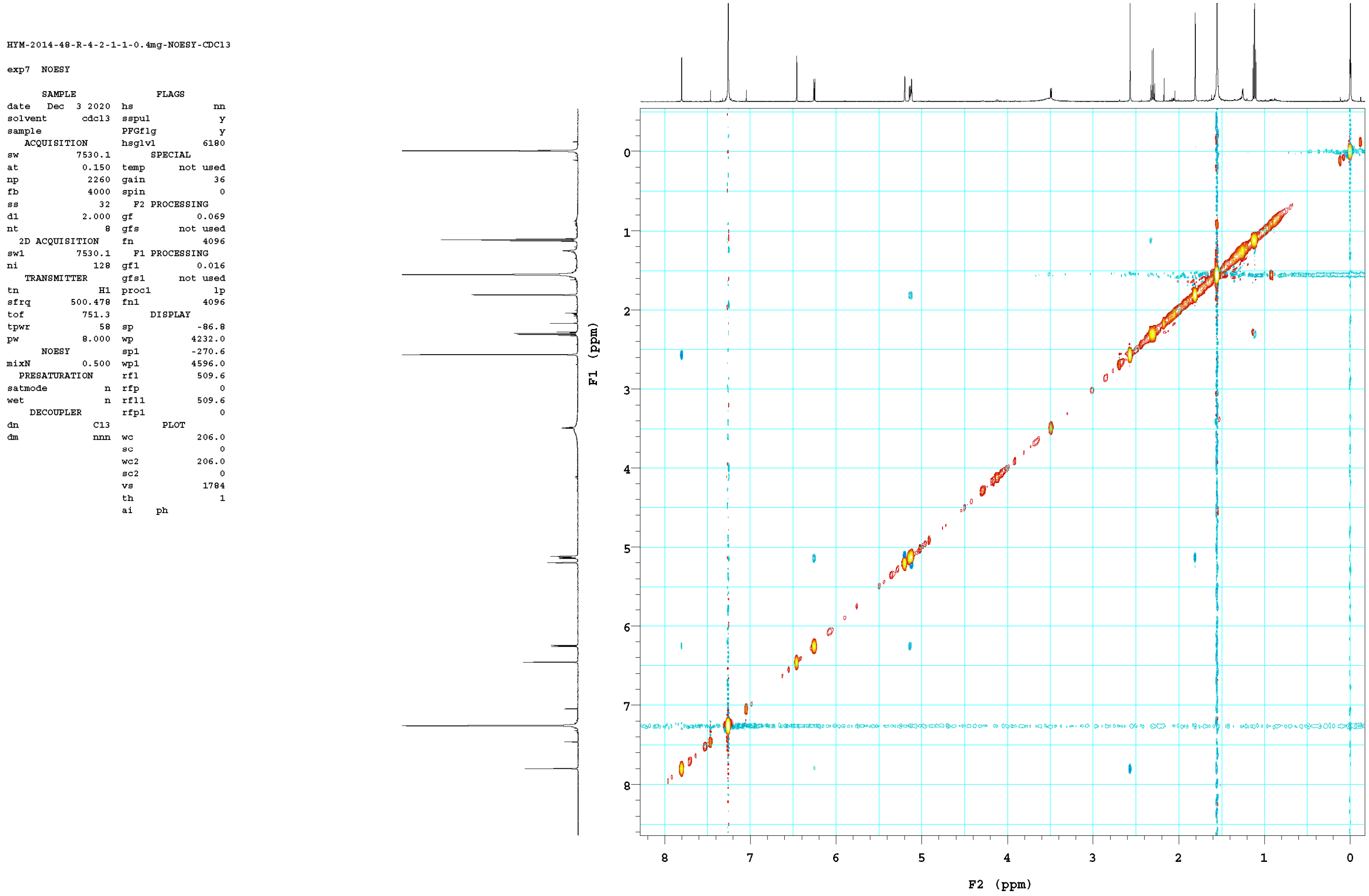

Figure S40. ^{1}H NMR spectrum of **17** (measured in $CDCl_3$, 500 MHz).

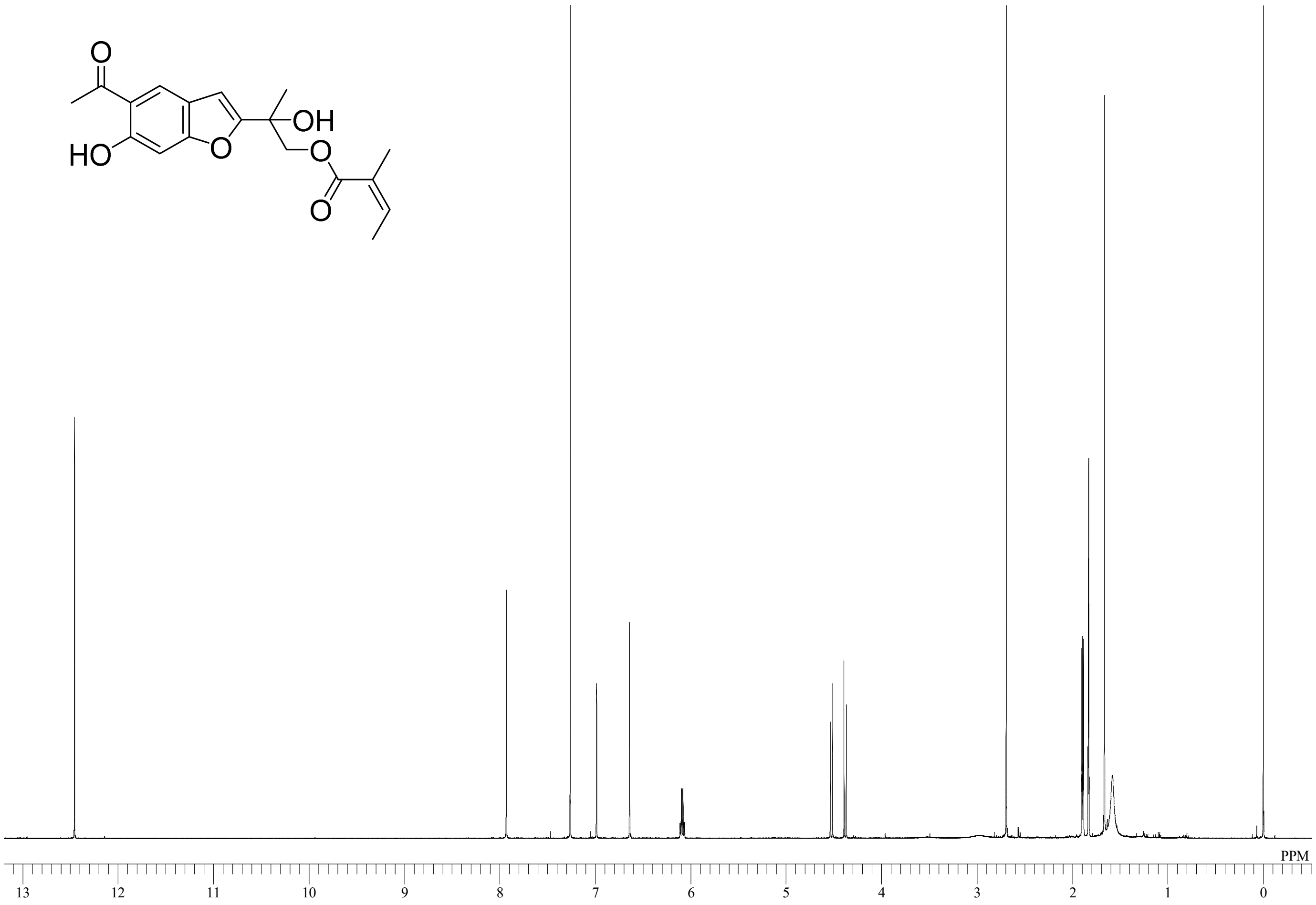

Figure S41. ^{13}C NMR spectrum of **17** (measured in $CDCl_3$, 126 MHz).

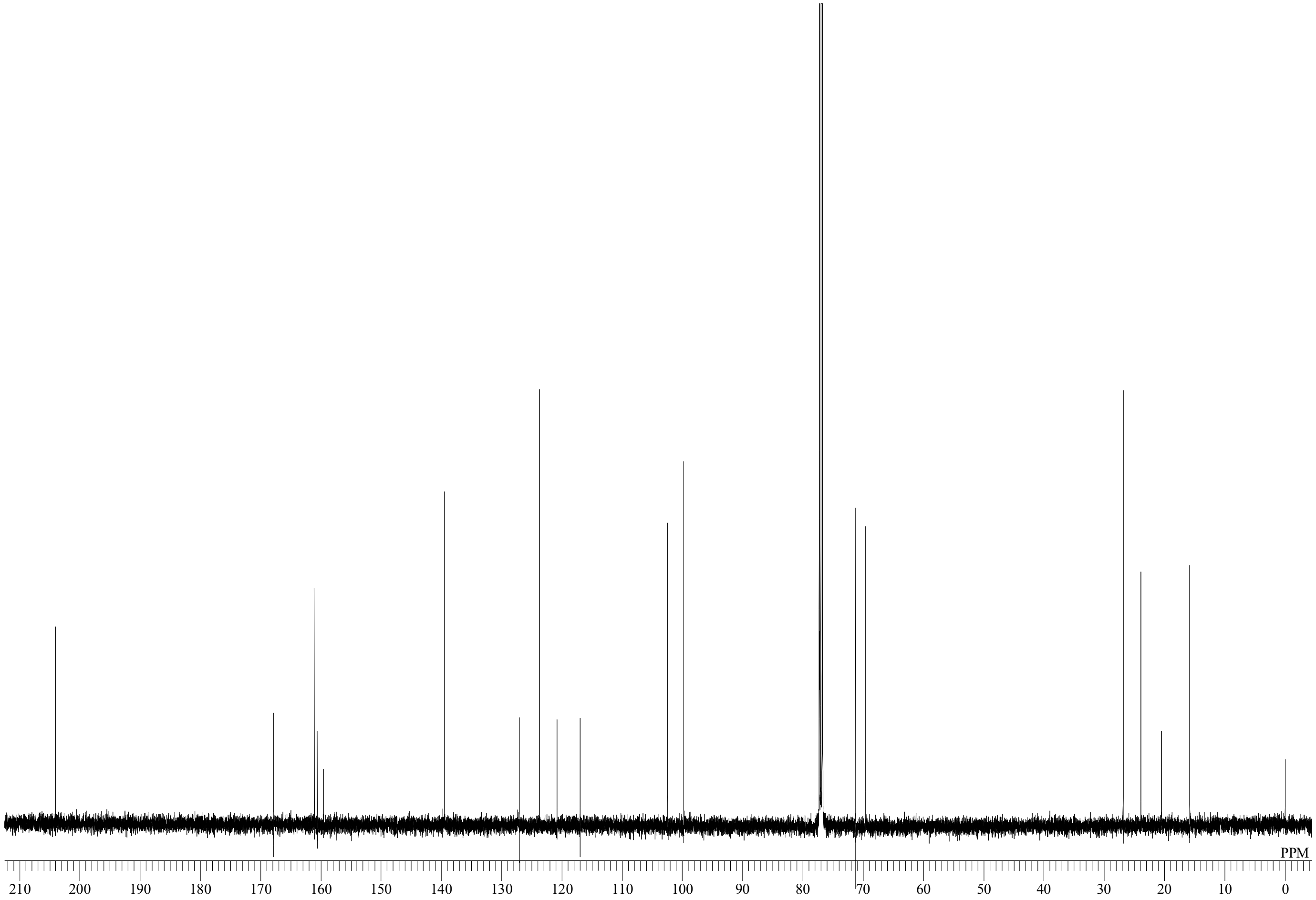

Figure S42. ^{1}H-^{1}H COSY spectrum of **17** (measured in $CDCl_3$, 500 MHz).

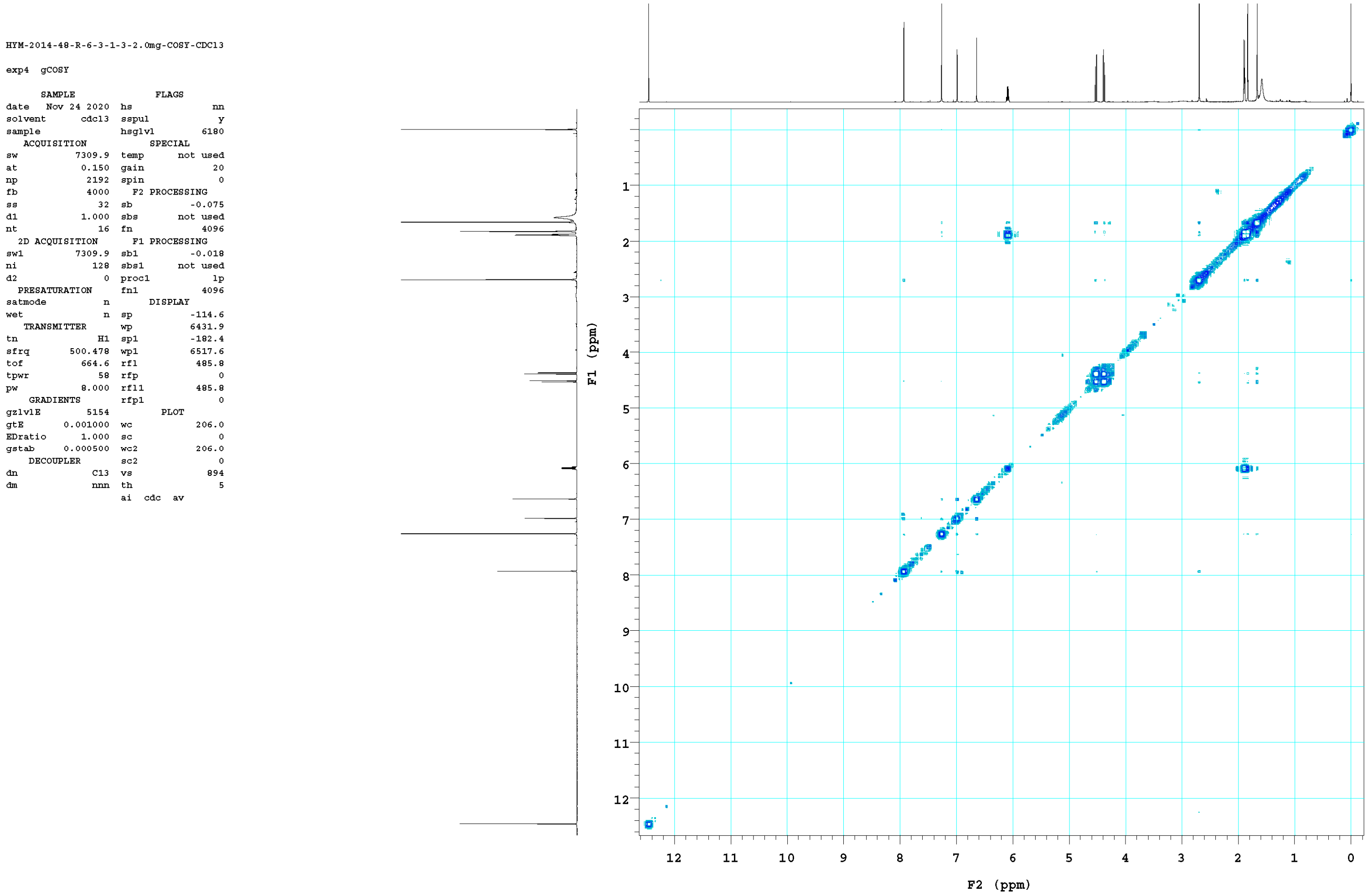

Figure S43. HSQC spectrum of **17** (measured in $CDCl_3$, 500 MHz).

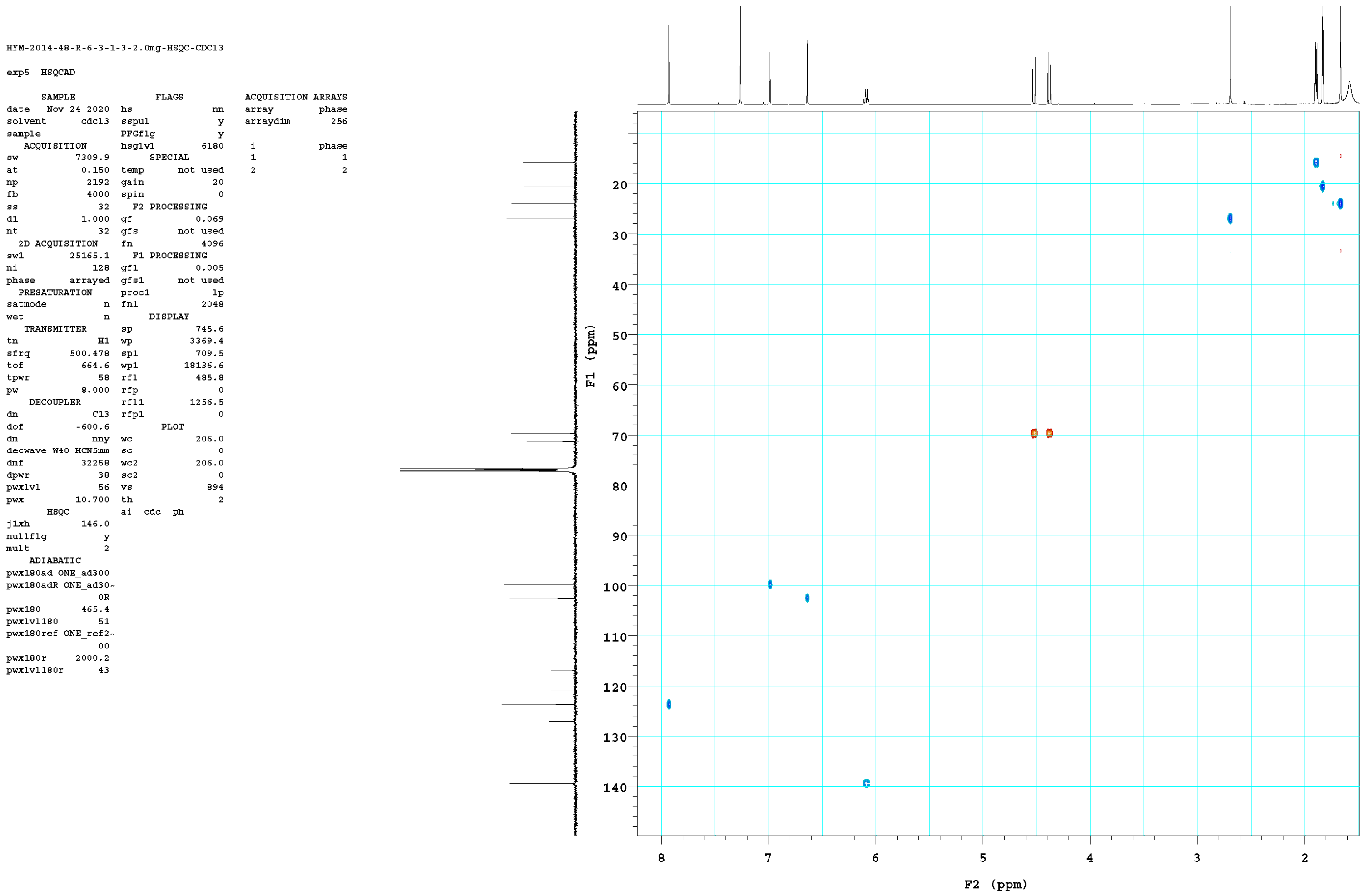

Figure S44. HMBC spectrum of **17** (measured in $CDCl_3$, 500 MHz).

```
HYM-2014-48-R-6-3-1-3-2.0mg-HMBC-CDCl3

exp6  gHMBCAD

     SAMPLE                 FLAGS            ACQUISITION ARRAYS
date   Nov 24 2020   hs               nn     array          phase
solvent      cdcl3   sspul             y     arraydim         256
sample               PFGflg            y
   ACQUISITION       hsglvl         6180      i             phase
sw          7309.9         SPECIAL            1                 1
at           0.150   temp       not used      2                 2
np            2192   gain             20
fb            4000   spin              0
ss              32       GRADIENTS
d1           1.000   gzlvl1          409
nt              32   gt1        0.001000
  2D ACQUISITION     gzlvl3         1227
sw1        30200.1   gt3        0.001000
ni             128   gstab      0.000500
phase      arrayed     F2 PROCESSING
  PRESATURATION      sb           -0.075
satmode          n   sbs        not used
wet              n   fn             4096
   TRANSMITTER         F1 PROCESSING
tn              H1   gf1           0.004
sfrq       500.478   gfs1       not used
tof          664.6   proc1            lp
tpwr            58   fn1            2048
pw           8.000         DISPLAY
    DECOUPLER        sp            -96.7
dn             C13   wp           6506.8
dof         1287.0   sp1          -824.7
dm             nnn   wp1         27663.7
decwave W40_HCN5mm   rfl           485.8
dmf          32258   rfp               0
dpwr            38   rfl1         1886.4
pwxlvl          56   rfp1              0
pwx         10.700          PLOT
       HMBC          wc            206.0
j1xh         146.0   sc                0
jnxh           8.0   wc2           206.0
    ADIABATCIC       sc2               0
pwx180ad ONE_ad300   vs              894
pwxlvl180       51   th                2
pwx180       465.4   ai   cdc   av
```

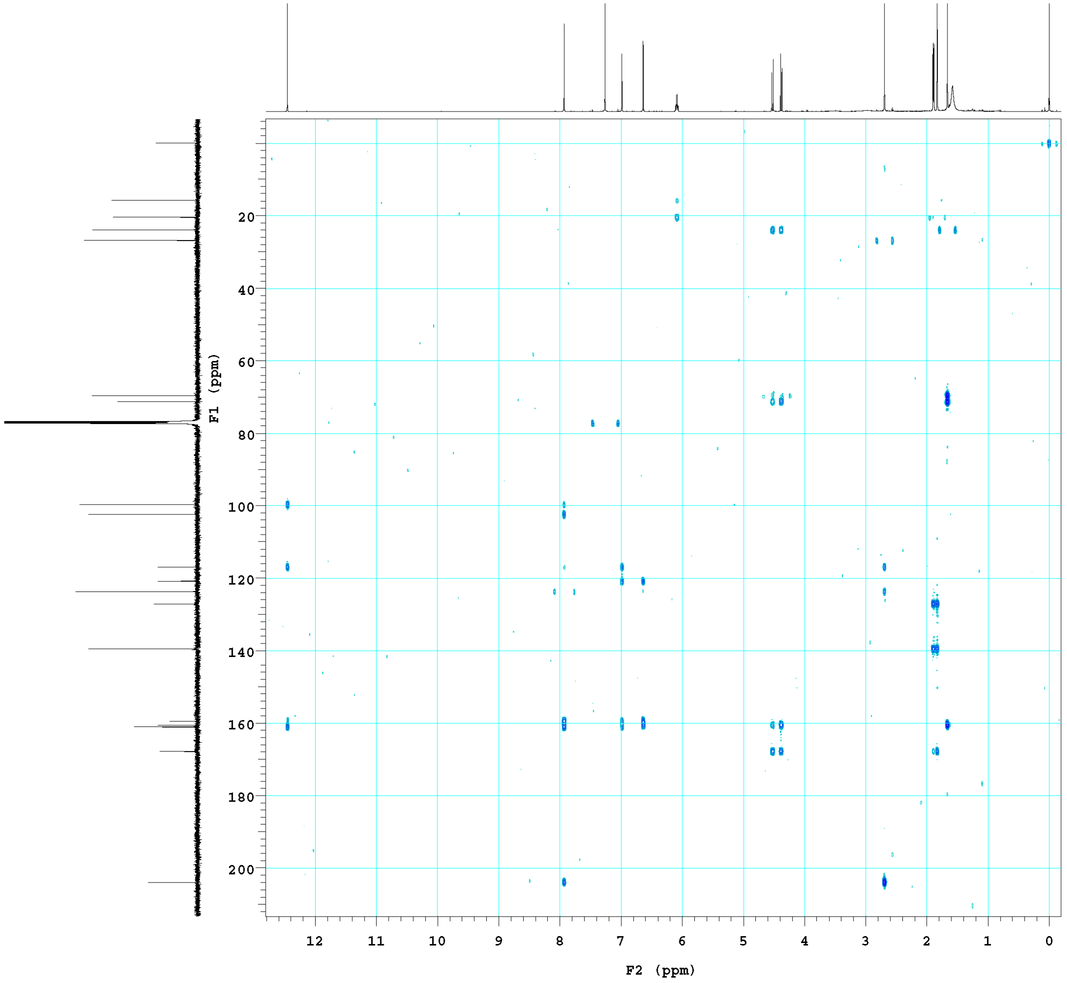

Figure S45. NOESY spectrum of **17** (measured in $CDCl_3$, 500 MHz).

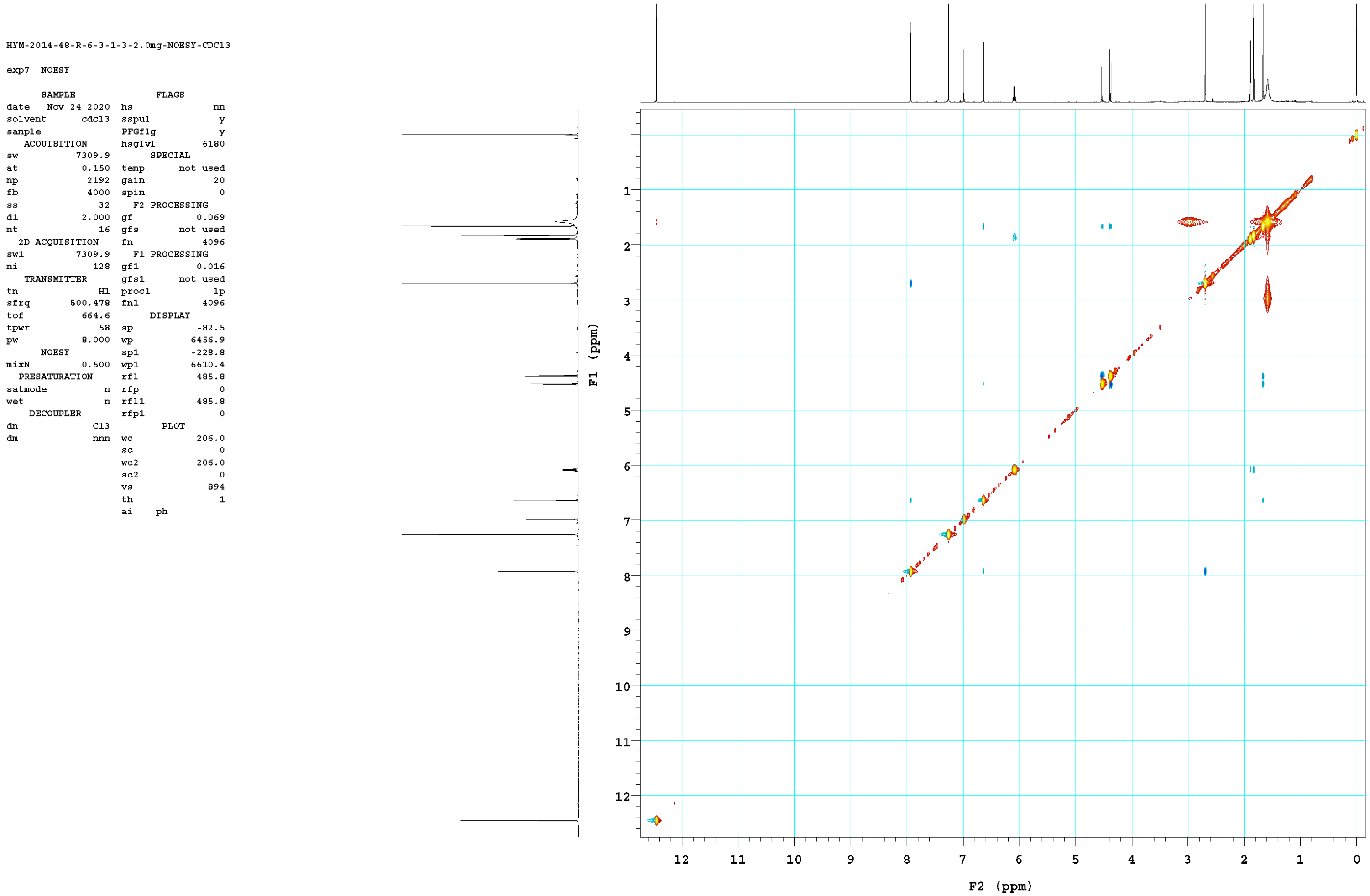

Figure S46. ^{1}H NMR spectrum of **21** (measured in $CDCl_3$, 500 MHz).

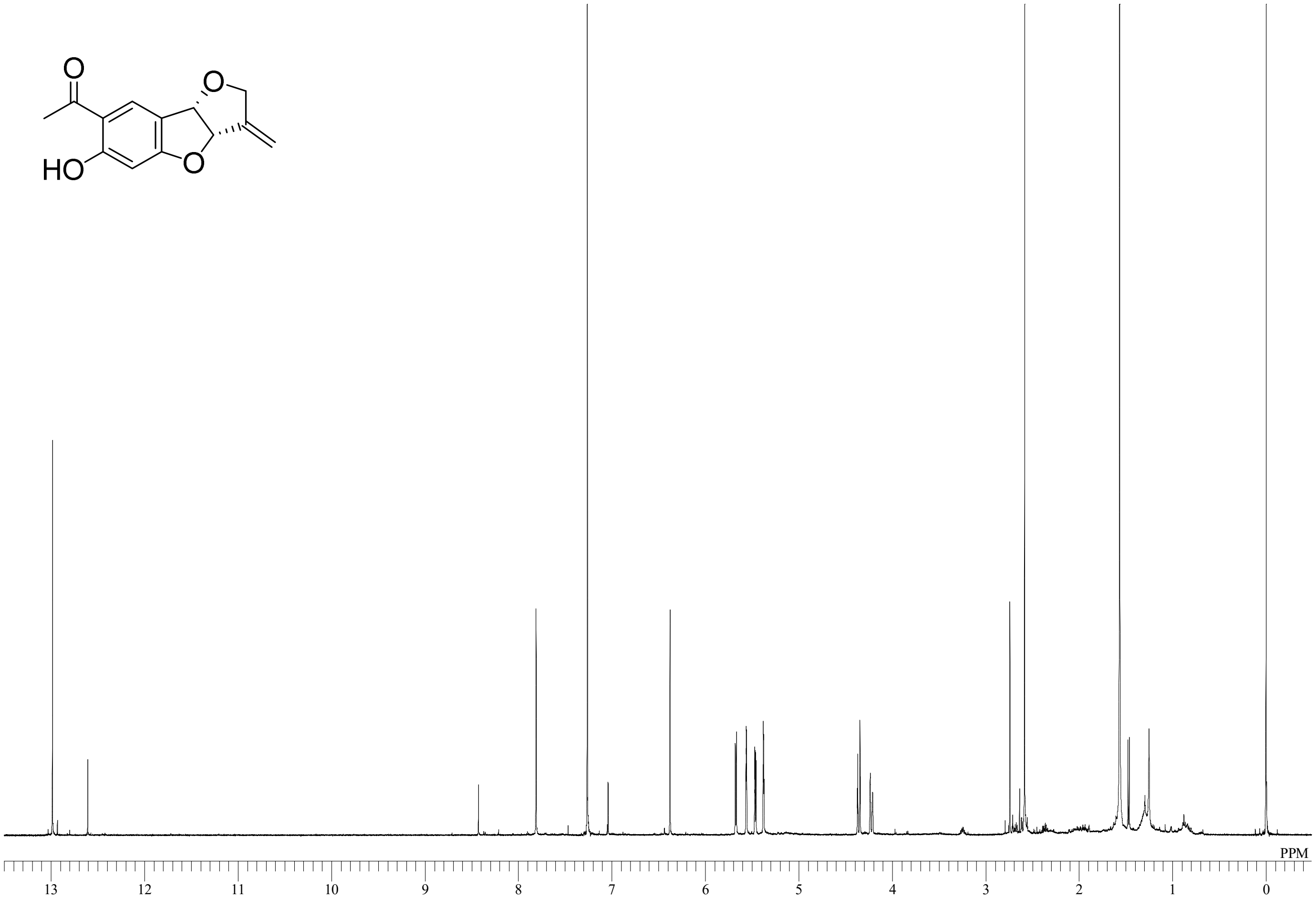

Figure S47. ^{13}C NMR spectrum of **21** (measured in $CDCl_3$, 126 MHz).

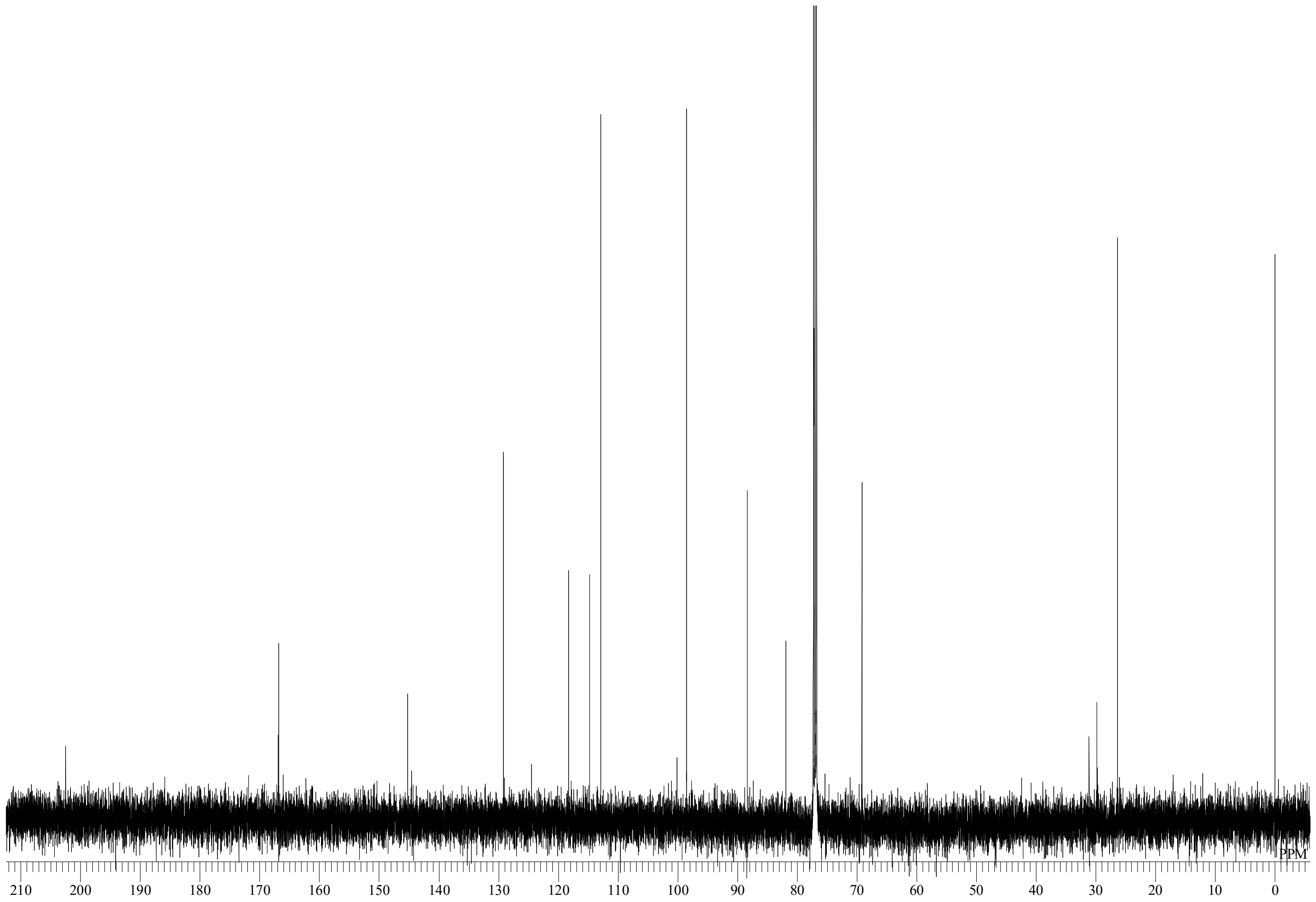

Figure S48. ^{1}H-^{1}H COSY spectrum of **21** (measured in $CDCl_3$, 500 MHz).

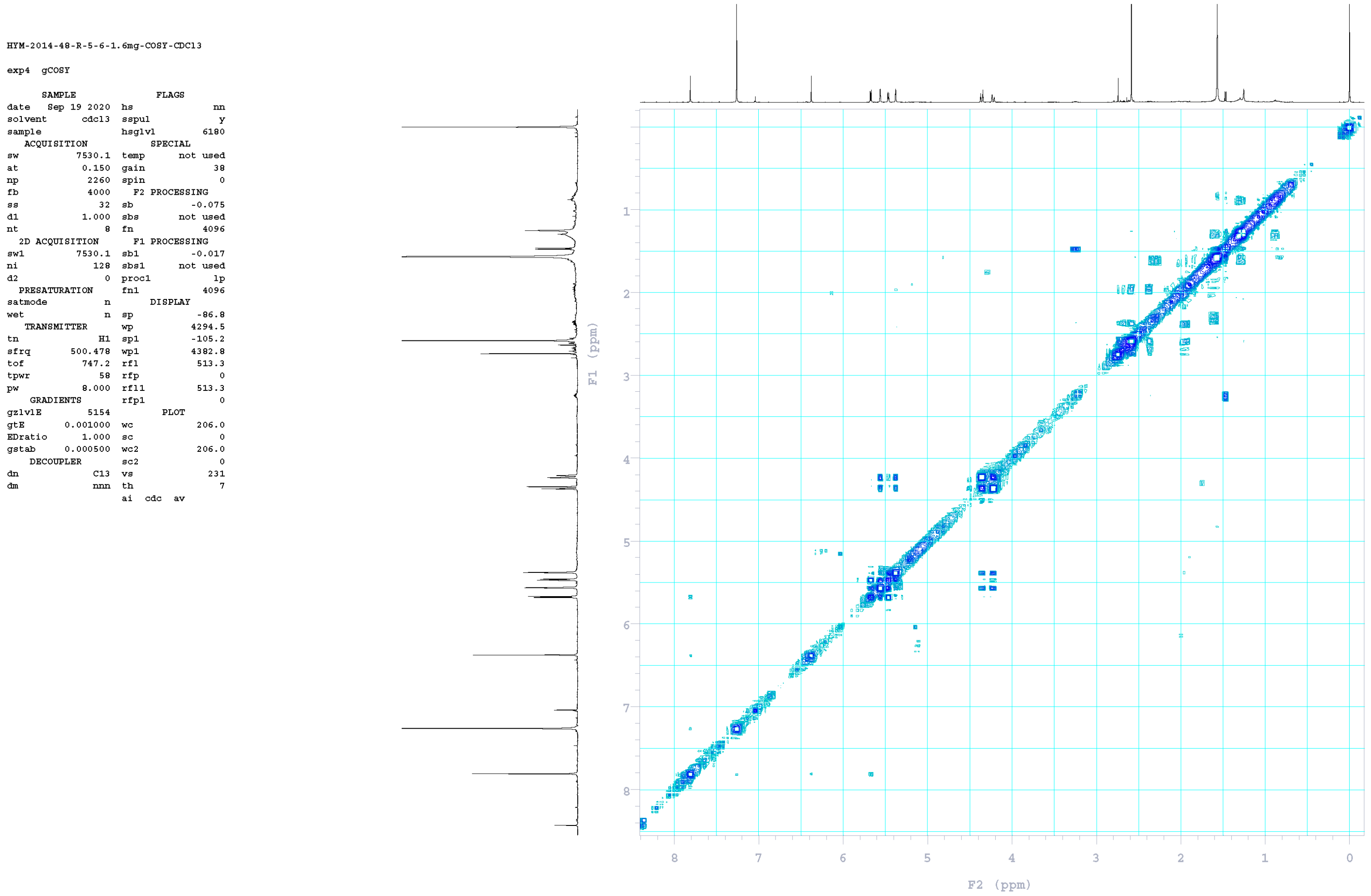

Figure S49. HSQC spectrum of **21** (measured in $CDCl_3$, 500 MHz).

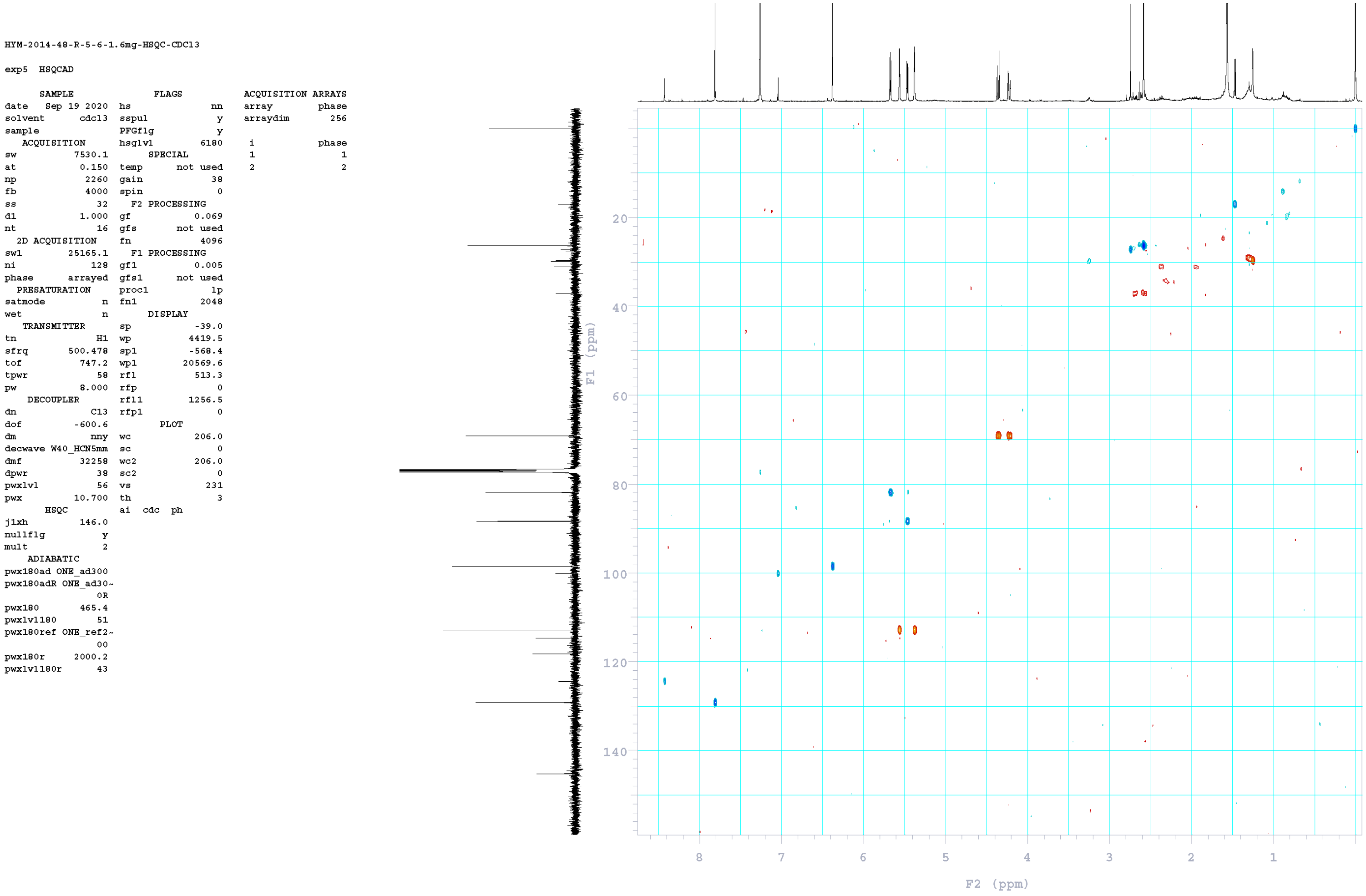

Figure S50. HMBC spectrum of **21** (measured in $CDCl_3$, 500 MHz).

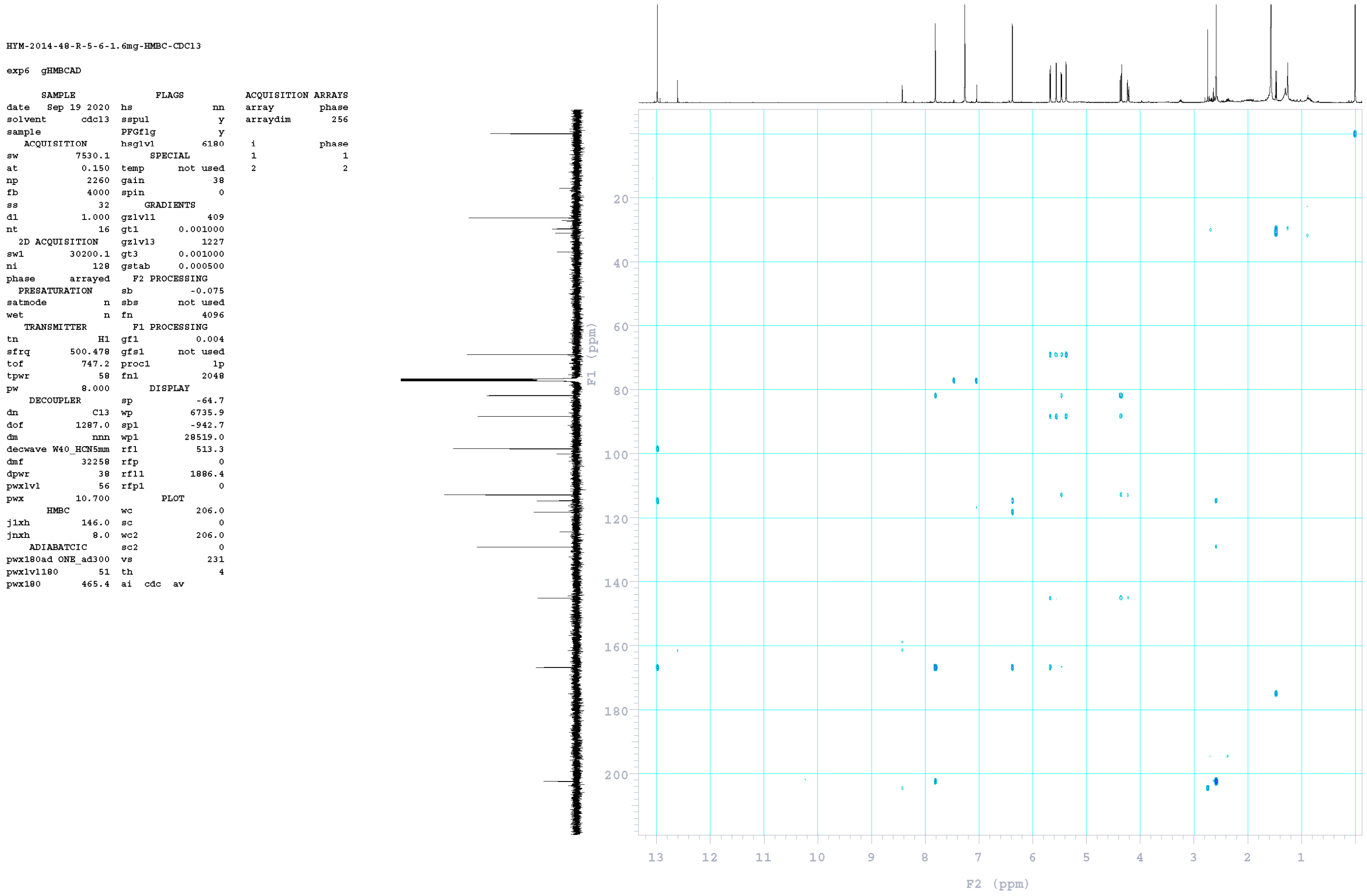

Figure S51. NOESY spectrum of **21** (measured in $CDCl_3$, 500 MHz).

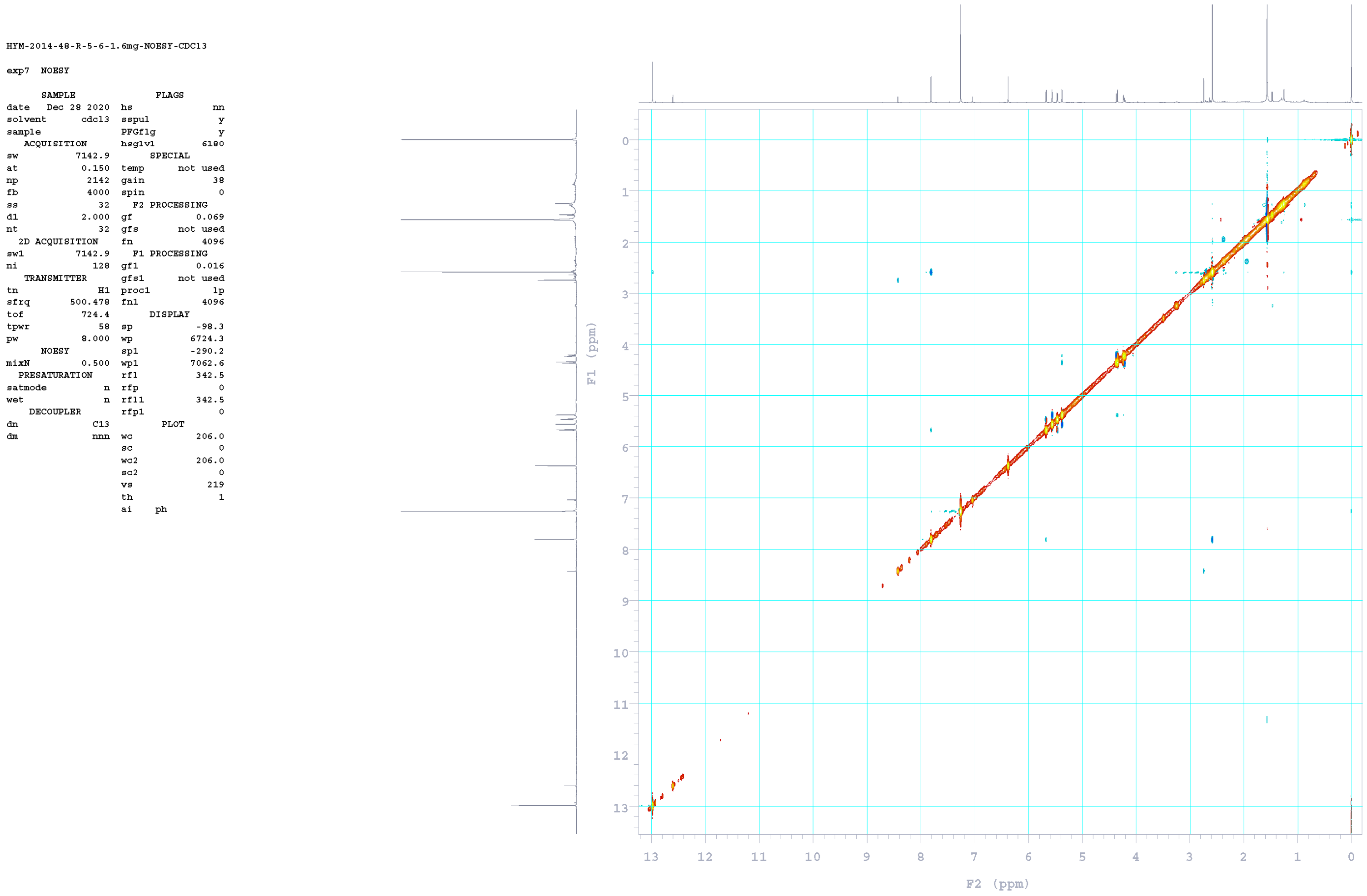

Figure S52. ^{1}H NMR spectrum of **25** (measured in $CDCl_3$, 500 MHz).

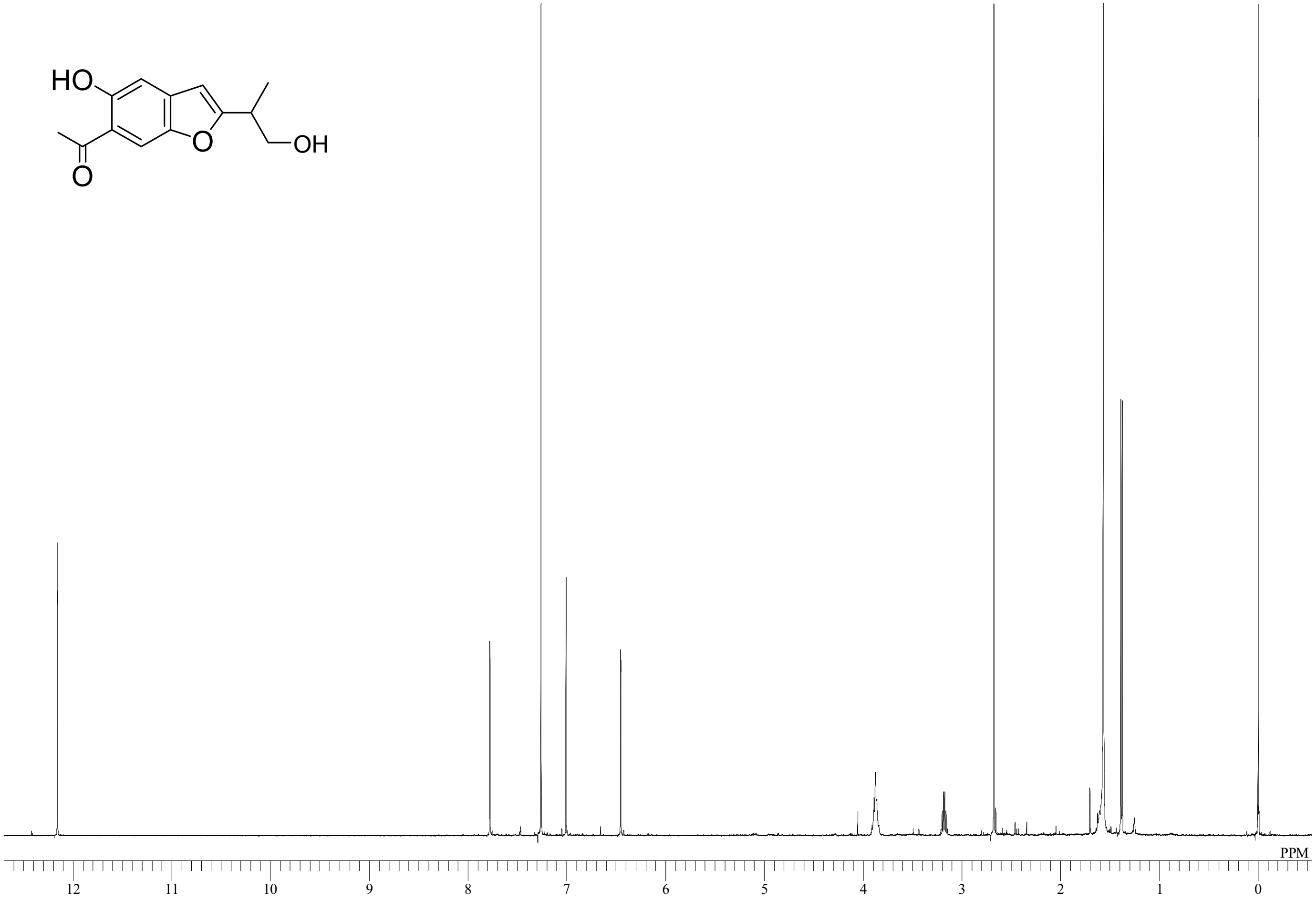

Figure S53. ^{13}C NMR spectrum of **25** (measured in $CDCl_3$, 126 MHz).

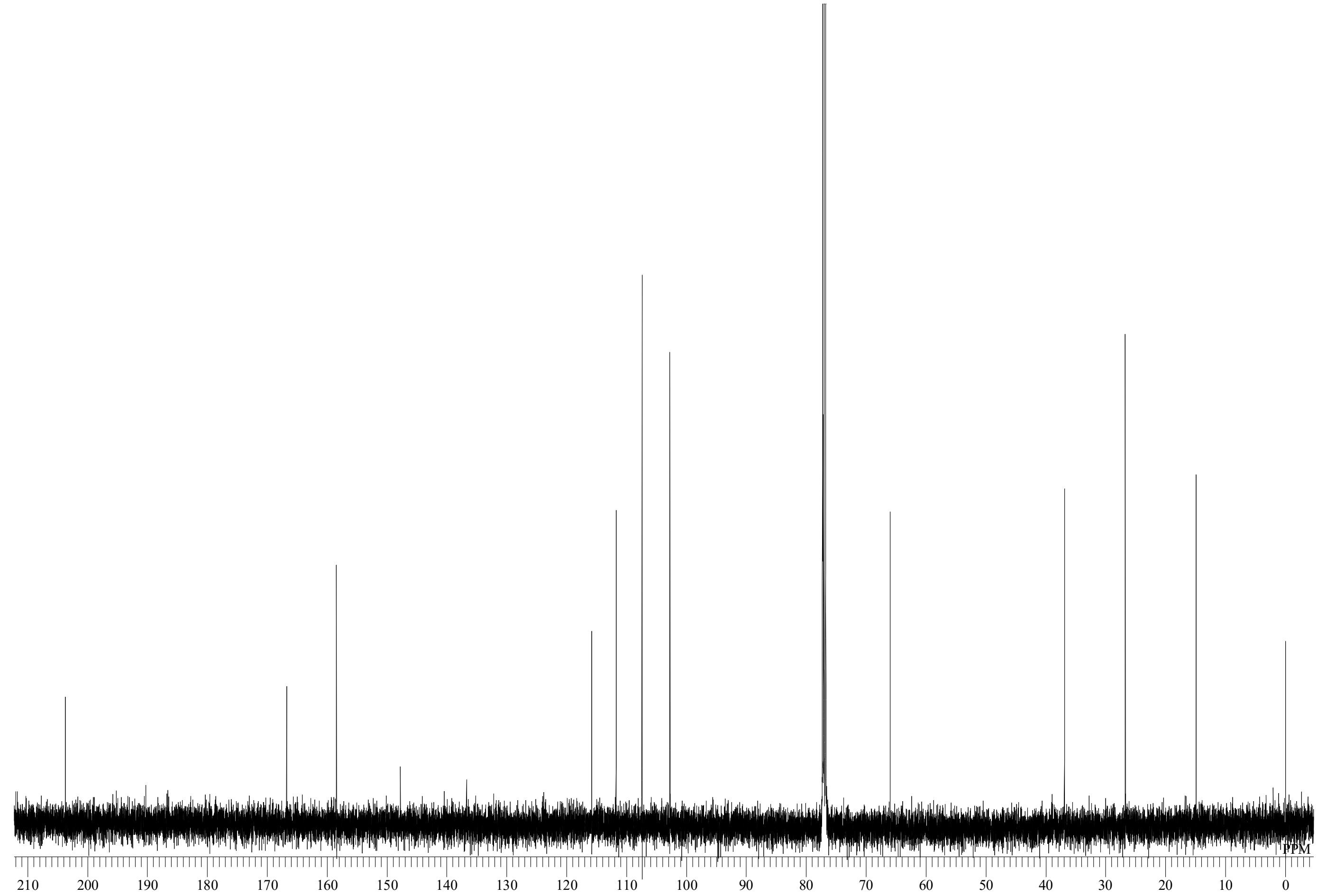

Figure S54. ^{1}H-^{1}H COSY spectrum of **25** (measured in $CDCl_3$, 500 MHz).

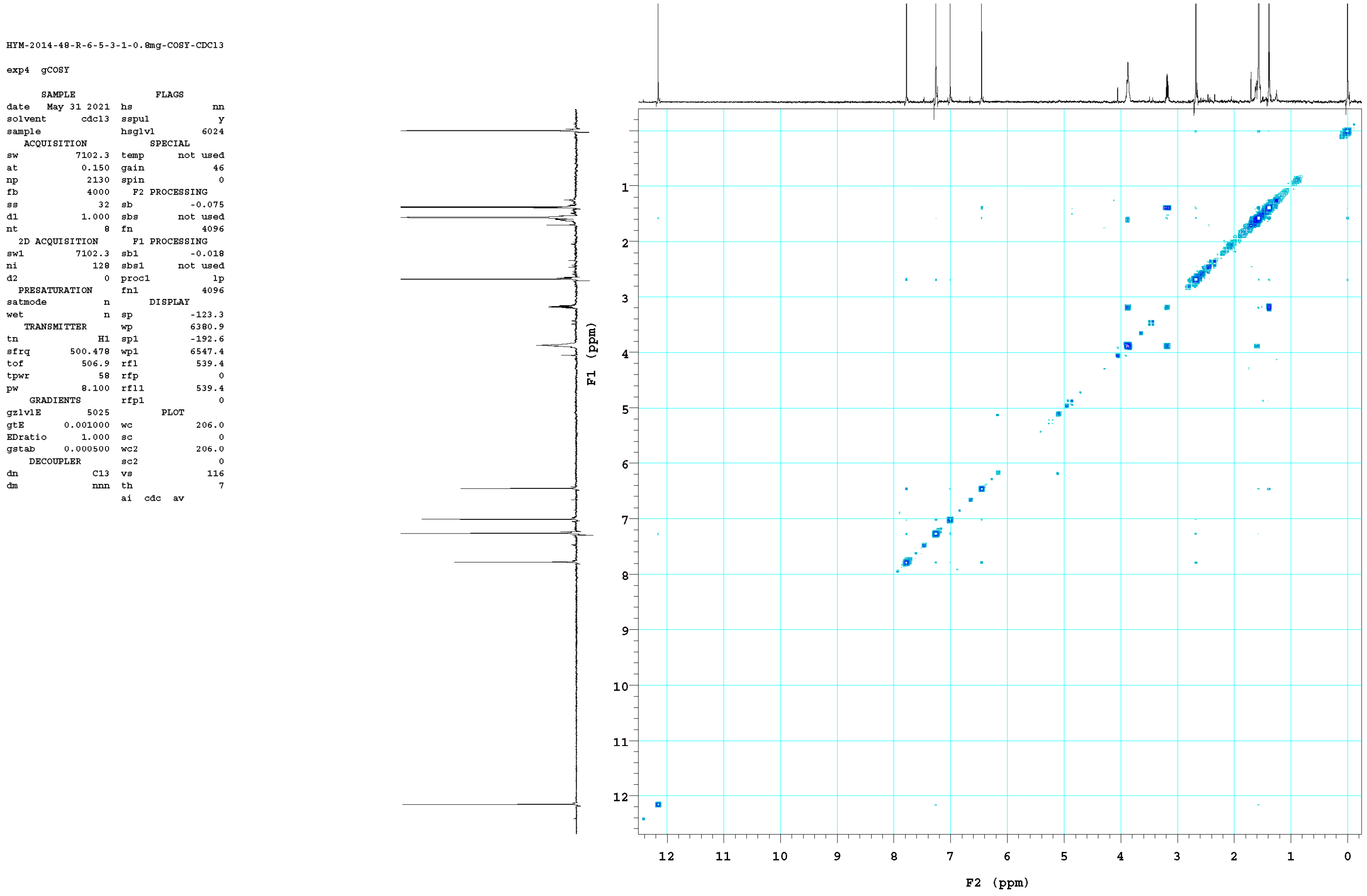

Figure S55. HSQC spectrum of **25** (measured in $CDCl_3$, 500 MHz).

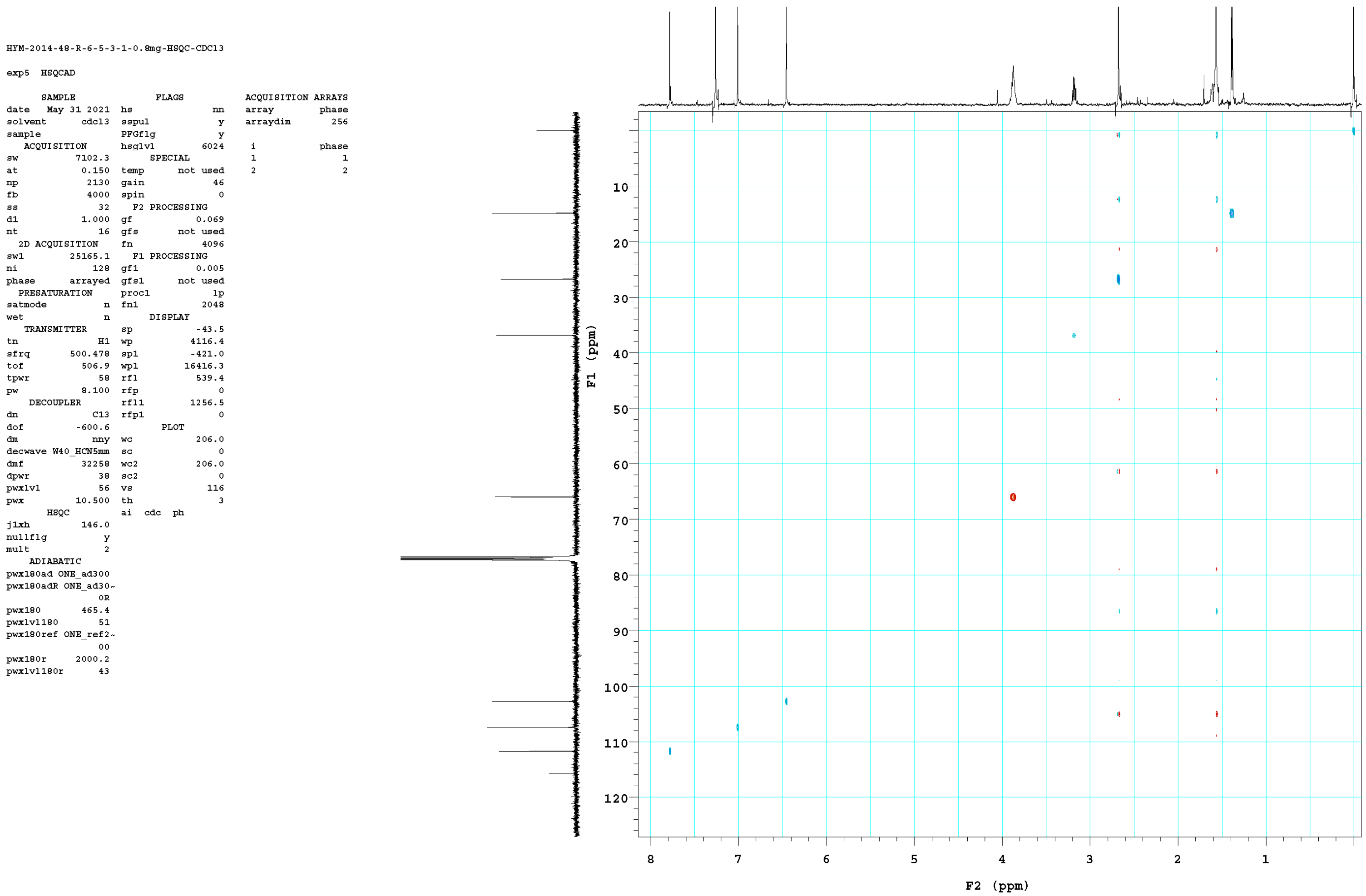

Figure S56. HMBC spectrum of **25** (measured in $CDCl_3$, 500 MHz).

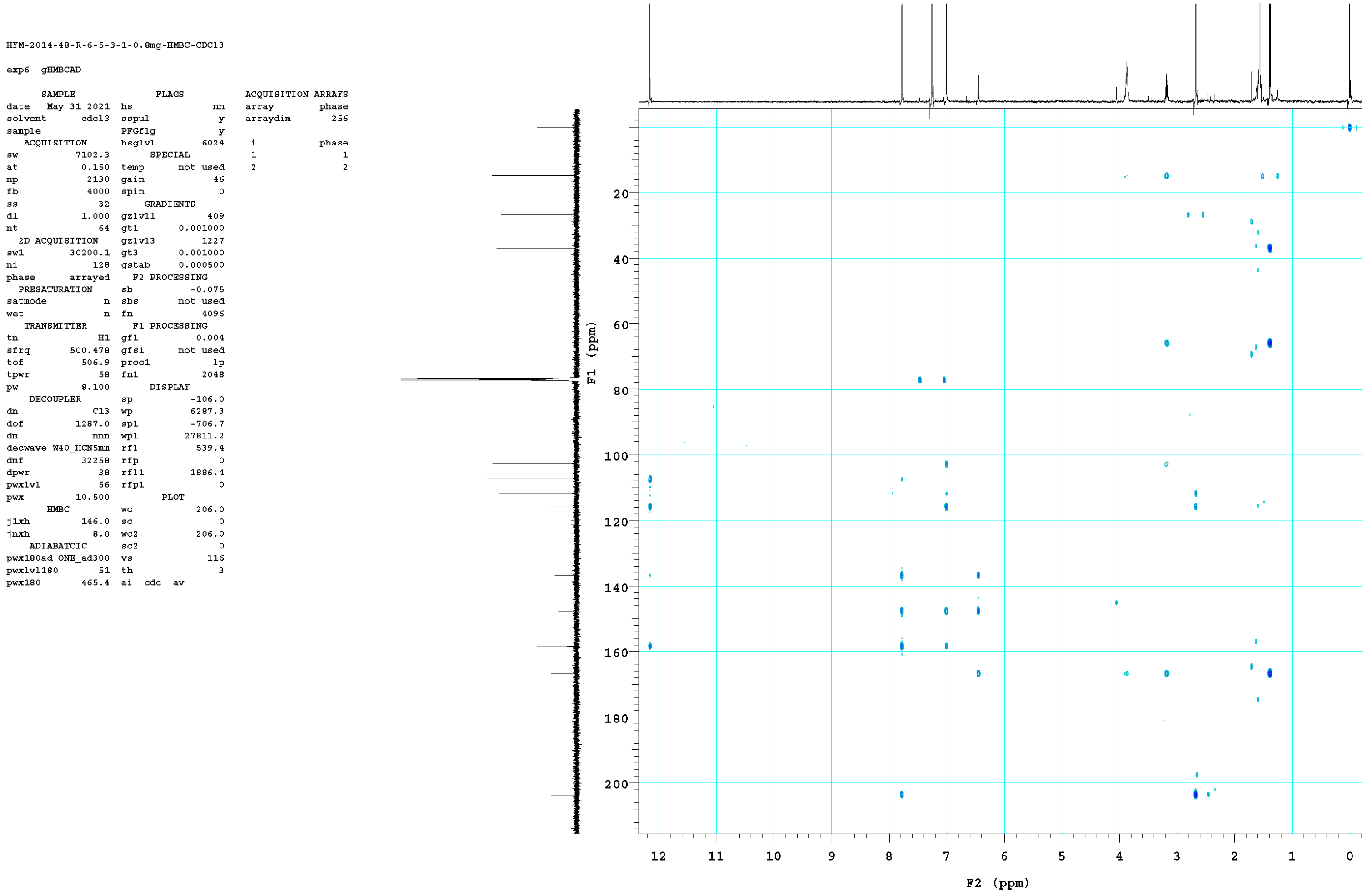

Figure S57. NOESY spectrum of **25** (measured in $CDCl_3$, 500 MHz).

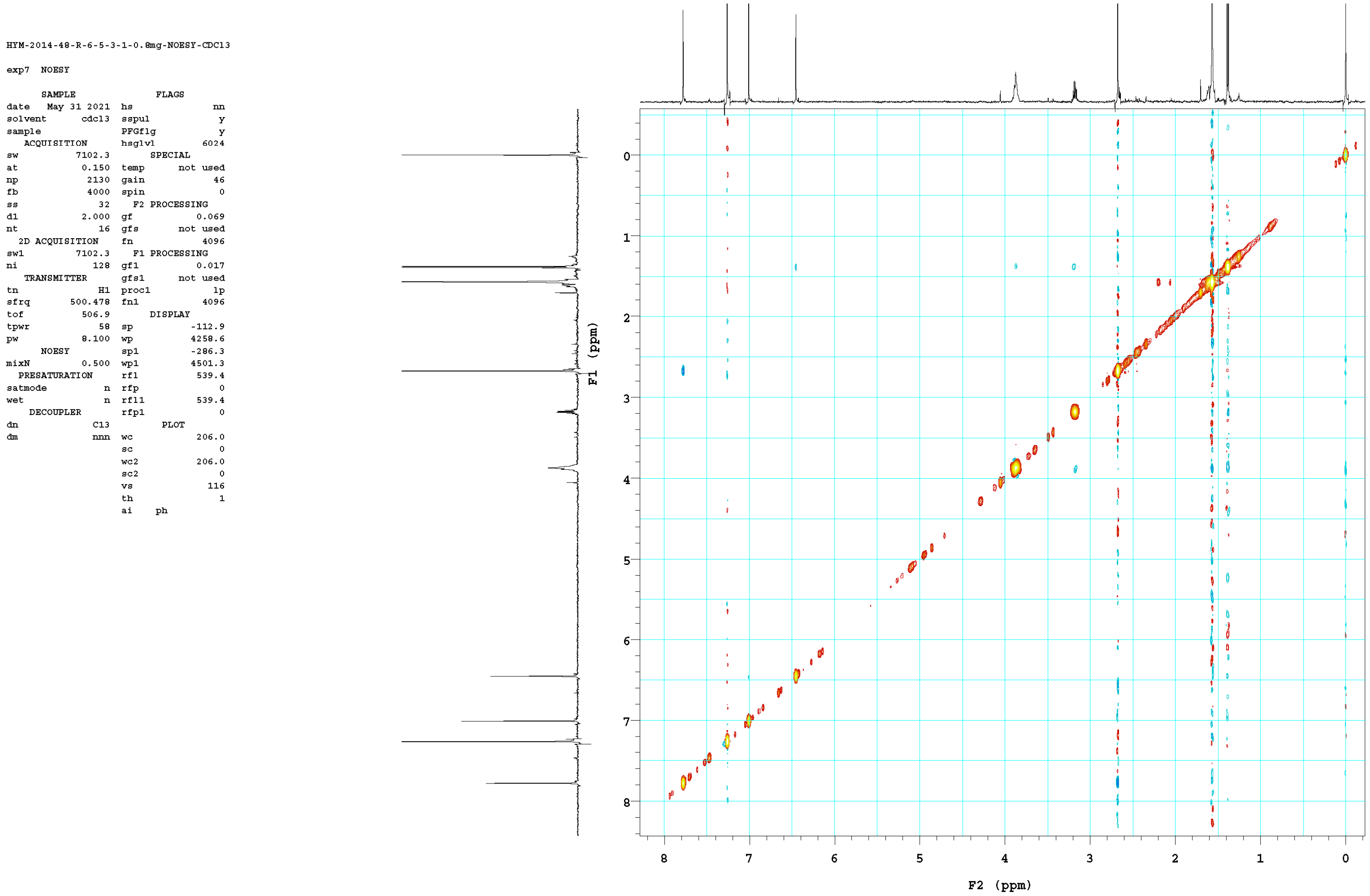

Figure S58. ^{1}H NMR spectrum of **26** (measured in $CDCl_3$, 500 MHz).

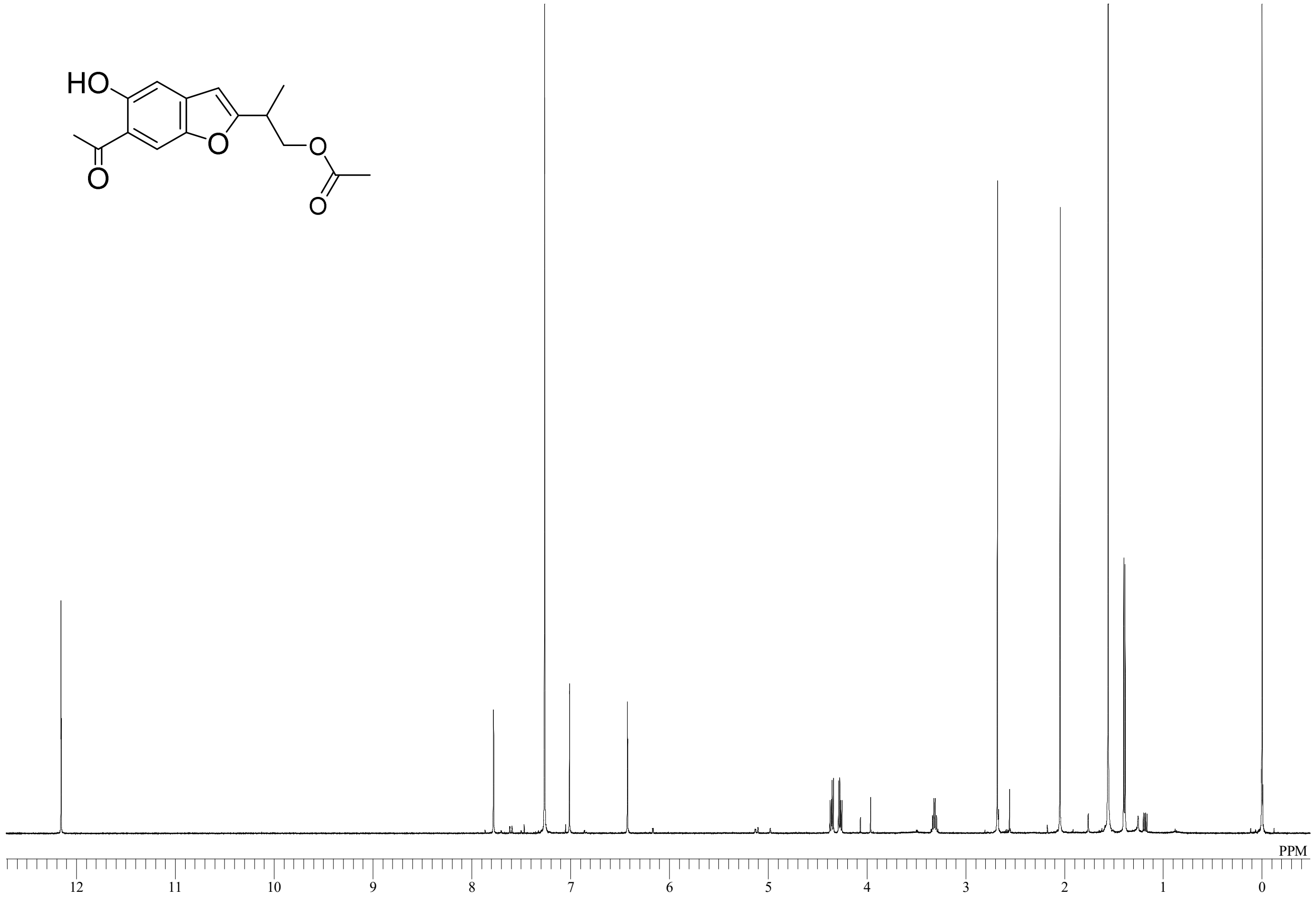

Figure S59. ^{13}C NMR spectrum of **26** (measured in $CDCl_3$, 126 MHz).

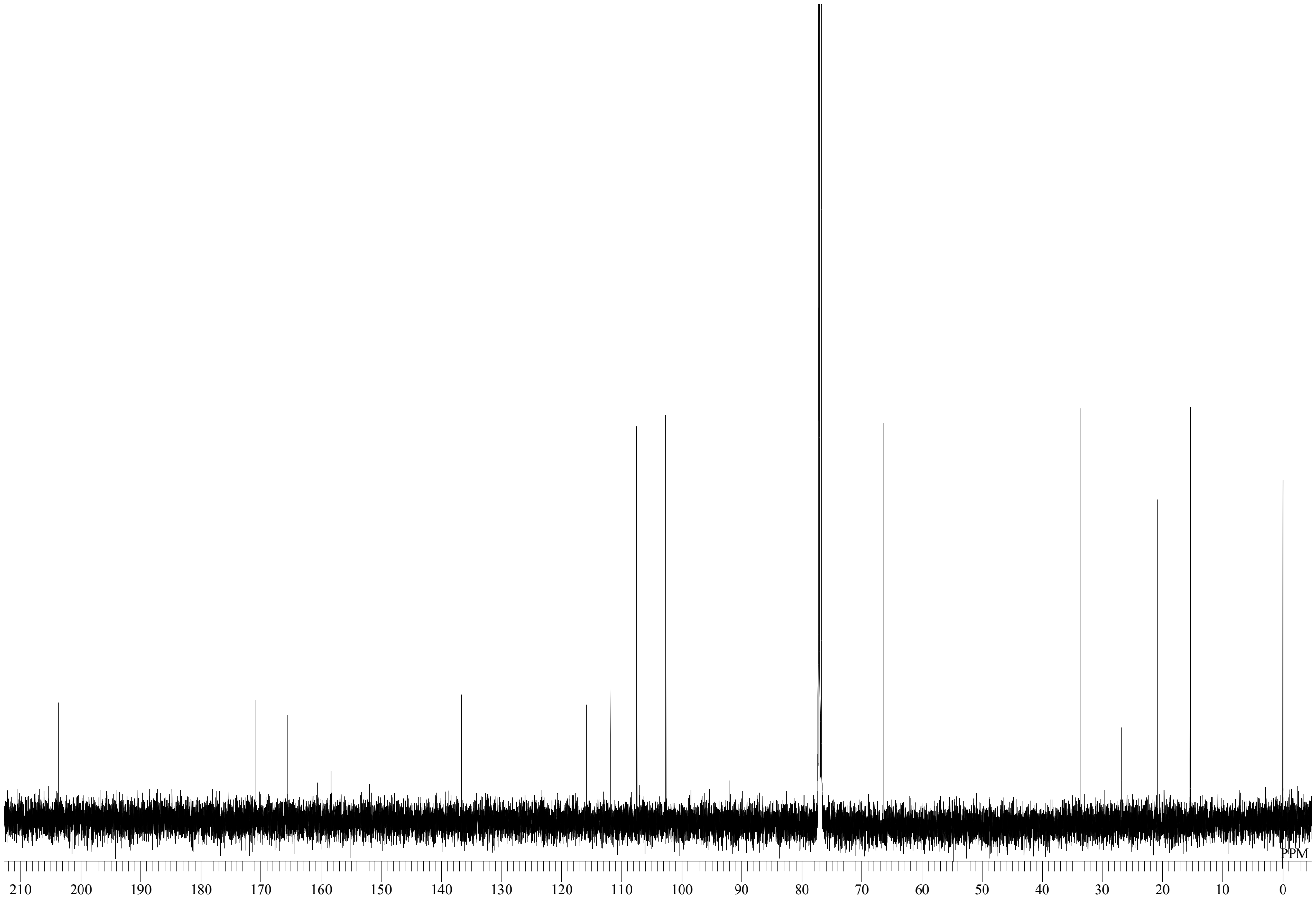

Figure S60. ^{1}H-^{1}H COSY spectrum of **26** (measured in $CDCl_3$, 500 MHz).

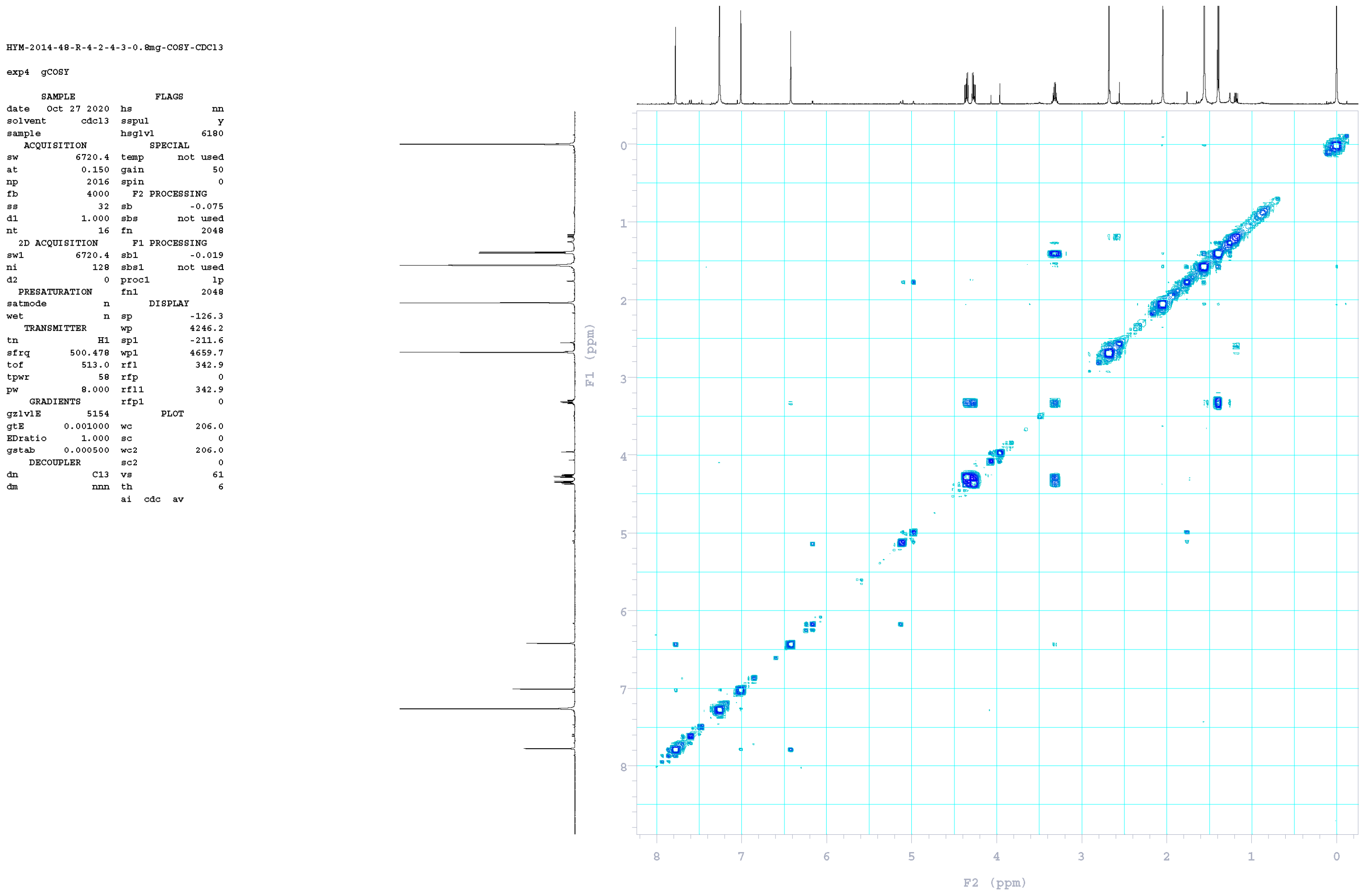

Figure S61. HSQC spectrum of **26** (measured in $CDCl_3$, 500 MHz).

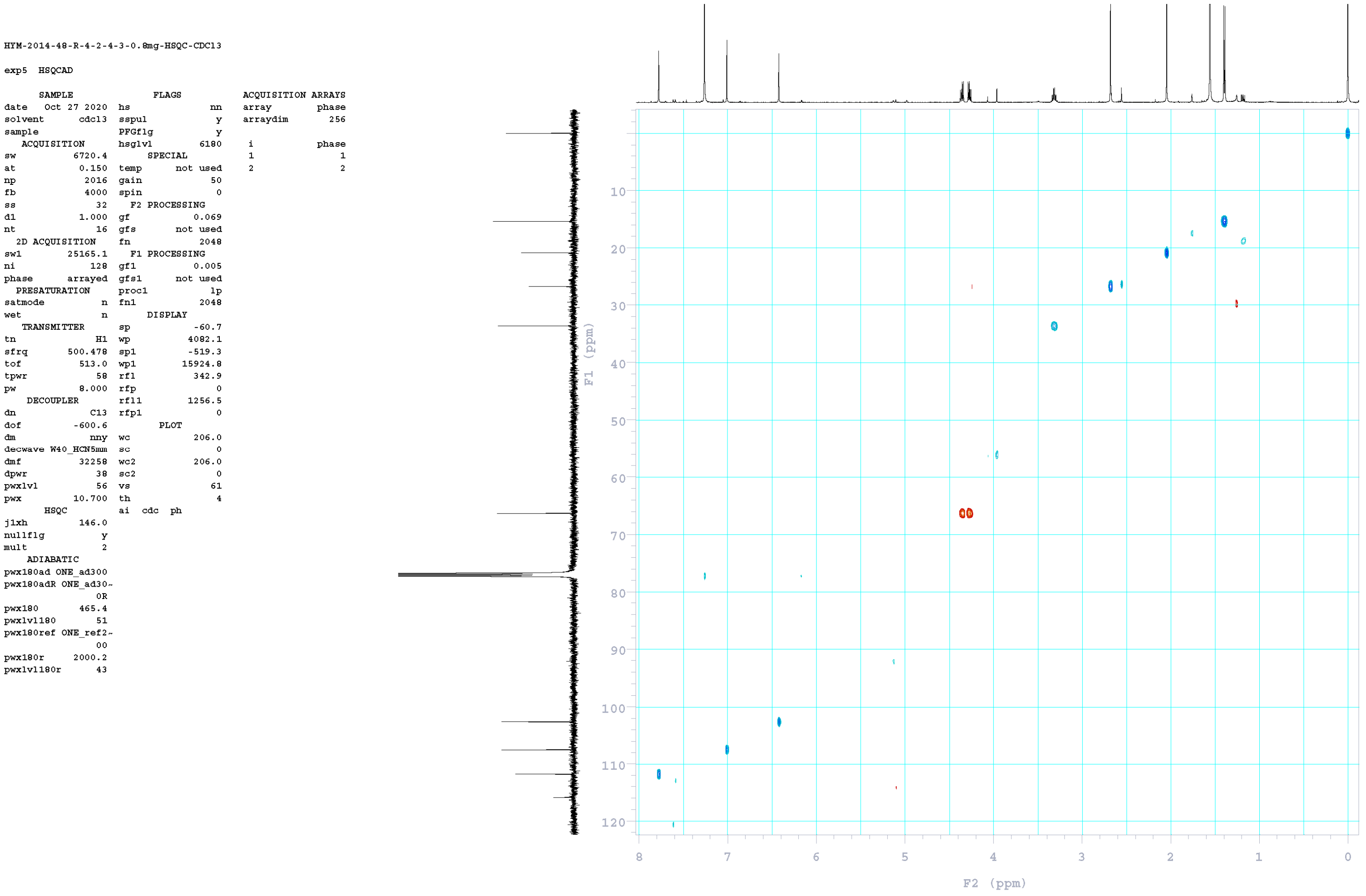

Figure S62. HMBC spectrum of **26** (measured in $CDCl_3$, 500 MHz).

```
HYM-2014-48-R-4-2-4-3-0.8mg-HMBC-CDCl3

exp6  gHMBCAD

      SAMPLE               FLAGS          ACQUISITION ARRAYS
date   Oct 27 2020  hs               nn   array         phase
solvent      cdcl3  sspul             y   arraydim        256
sample              PFGflg            y
   ACQUISITION      hsglvl         6180    i            phase
sw          6720.4        SPECIAL          1                1
at           0.150  temp       not used    2                2
np            2016  gain             50
fb            4000  spin              0
ss              32       GRADIENTS
d1           1.000  gzlvl1          409
nt              32  gt1        0.001000
  2D ACQUISITION    gzlvl3         1227
sw1        30200.1  gt3        0.001000
ni             128  gstab      0.000500
phase      arrayed    F2 PROCESSING
  PRESATURATION     sb           -0.075
satmode          n  sbs        not used
wet              n  fn             2048
   TRANSMITTER        F1 PROCESSING
tn              H1  gf1           0.004
sfrq       500.478  gfs1       not used
tof          513.0  proc1            lp
tpwr            58  fn1            2048
pw           8.000        DISPLAY
    DECOUPLER       sp            -93.5
dn             C13  wp           6307.0
dof         1287.0  sp1          -824.7
dm             nnn  wp1         28047.1
decwave W40_HCN5mm  rf1           342.9
dmf          32258  rfp               0
dpwr            38  rfl1         1886.4
pwxlvl          56  rfp1              0
pwx         10.700          PLOT
       HMBC         wc            206.0
j1xh         146.0  sc                0
jnxh           8.0  wc2           206.0
    ADIABATCIC      sc2               0
pwx180ad ONE_ad300  vs               61
pwxlvl180       51  th                4
pwx180       465.4  ai   cdc   av
```

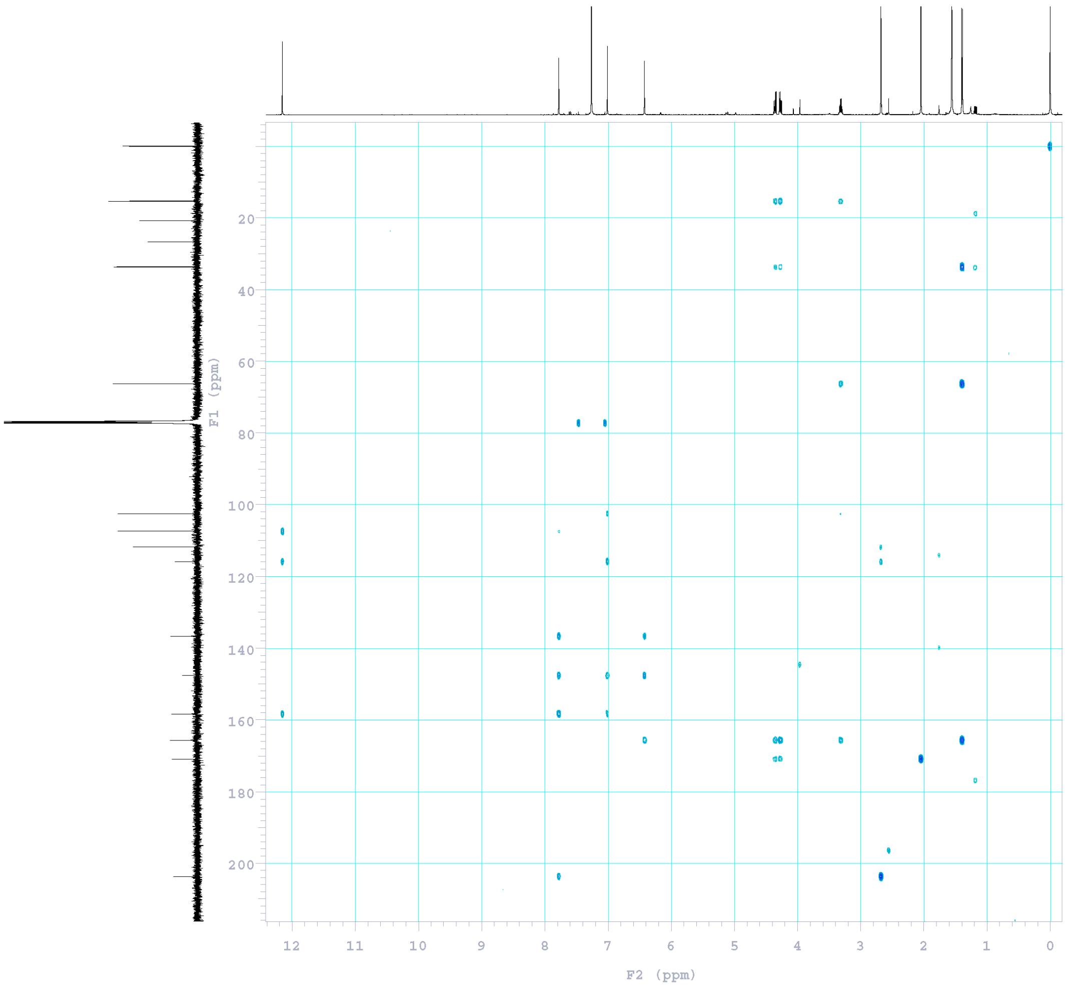

Figure S63. NOESY spectrum of **26** (measured in $CDCl_3$, 500 MHz).

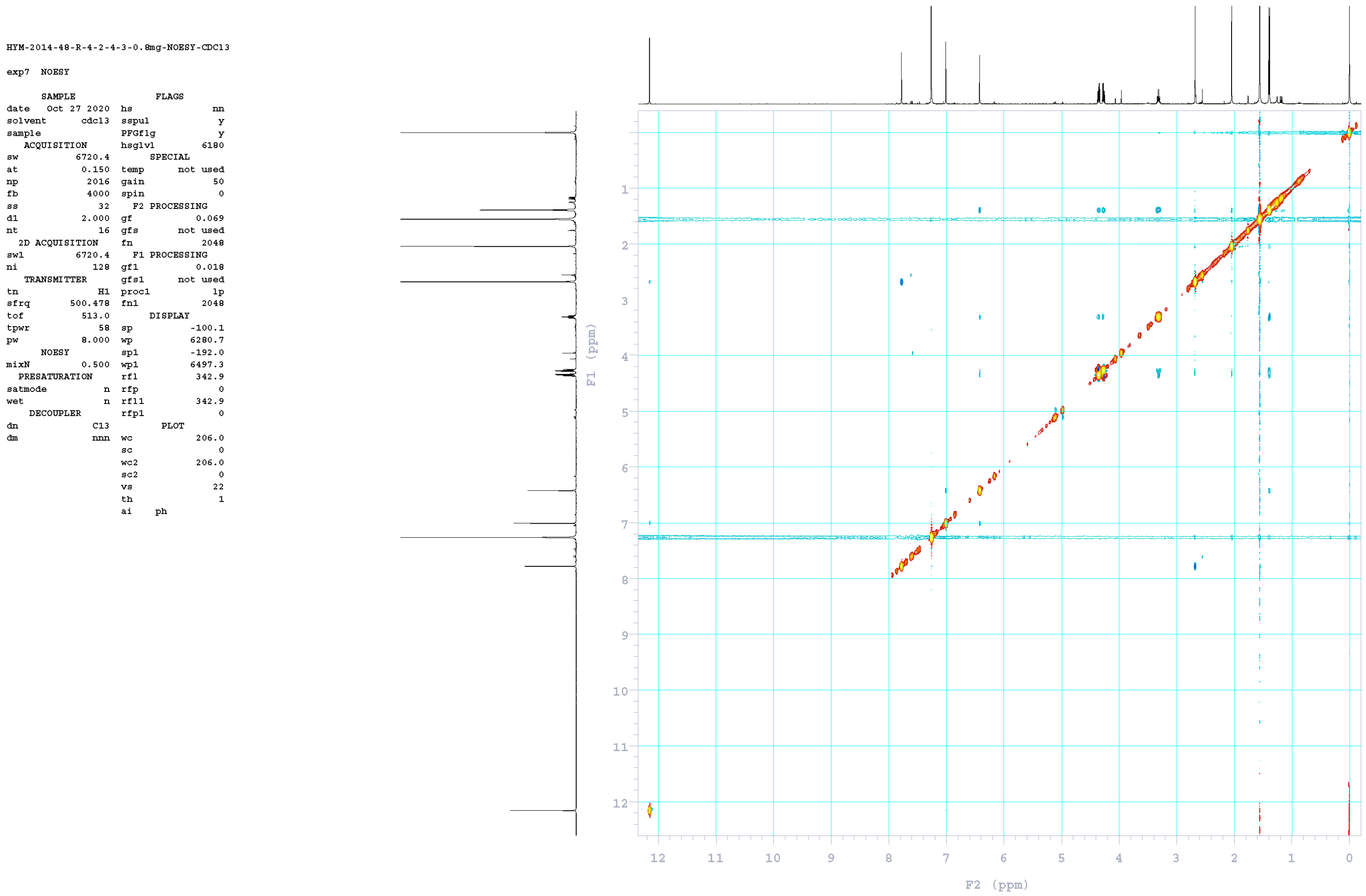

Figure S64. ^{1}H NMR spectrum of **31** (measured in $CDCl_3$, 500 MHz).

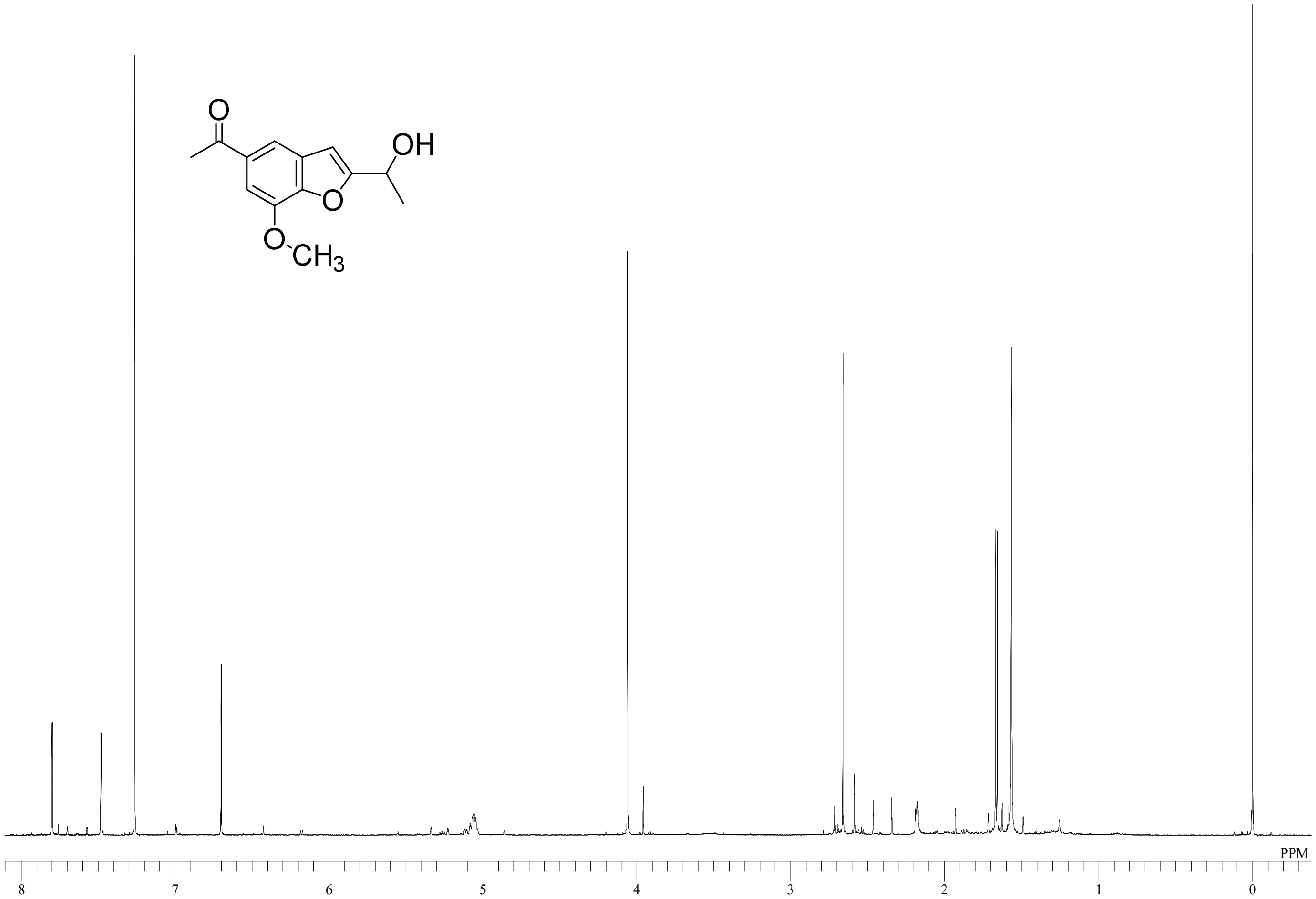

Figure S65. ^{13}C NMR spectrum of **31** (measured in $CDCl_3$, 126 MHz).

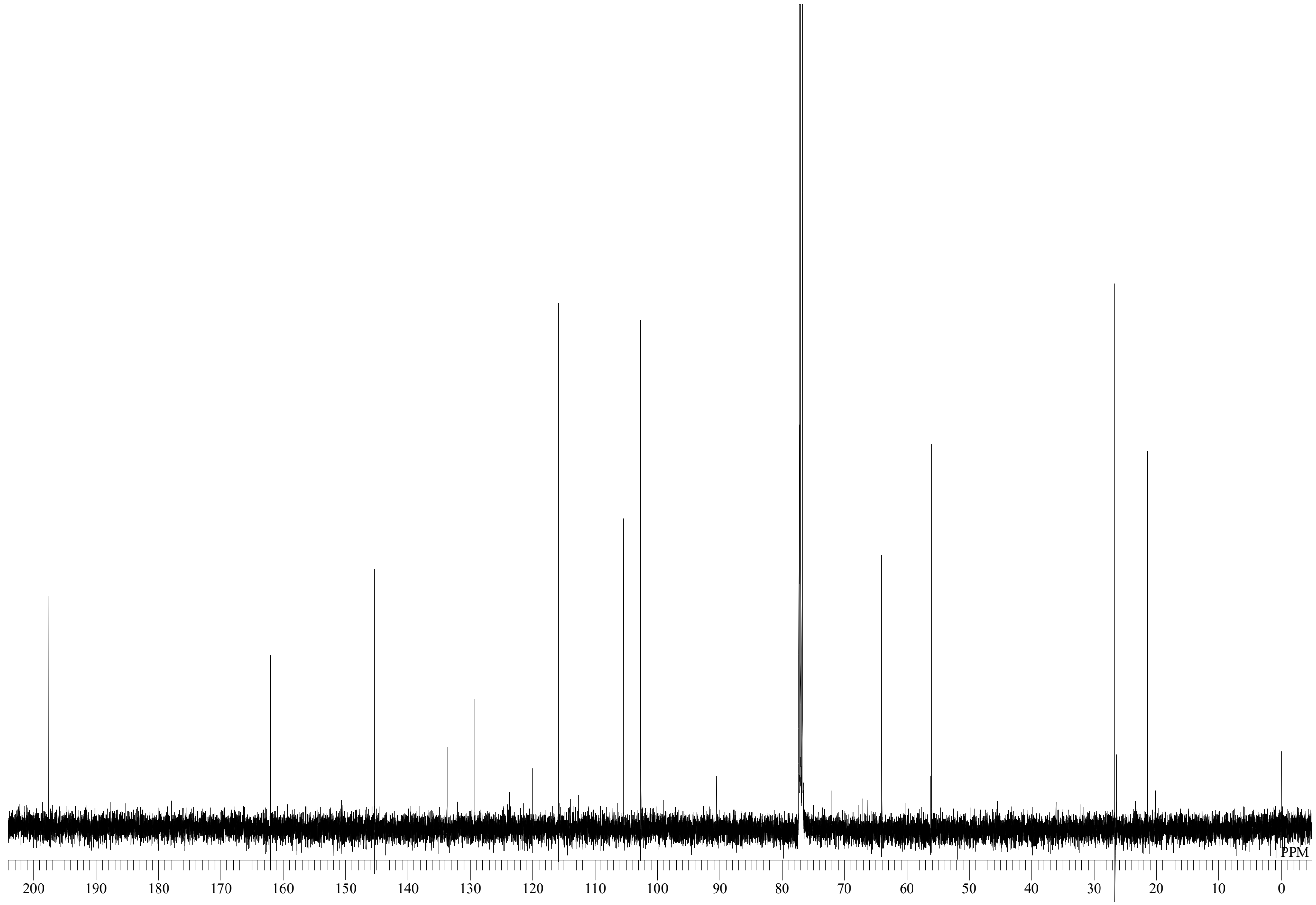

Figure S66. ^{1}H-^{1}H COSY spectrum of **31** (measured in $CDCl_3$, 500 MHz).

HYM-2014-48-R-6-6-3-1.4mg-COSY-CDCl3

exp4 gCOSY

SAMPLE		FLAGS	
date	Nov 22 2020	hs	nn
solvent	cdcl3	sspul	y
sample		hsglvl	6180
ACQUISITION		SPECIAL	
sw	4734.8	temp	not used
at	0.150	gain	48
np	1420	spin	0
fb	4000	F2 PROCESSING	
ss	32	sb	-0.075
d1	1.000	sbs	not used
nt	16	fn	2048
2D ACQUISITION		F1 PROCESSING	
sw1	4734.8	sb1	-0.027
ni	128	sbs1	not used
d2	0	proc1	lp
PRESATURATION		fn1	2048
satmode	n	DISPLAY	
wet	n	sp	-87.2
TRANSMITTER		wp	4226.2
tn	H1	sp1	-105.7
sfrq	500.477	wp1	4309.5
tof	-483.0	rfl	346.1
tpwr	58	rfp	0
pw	8.000	rfl1	346.1
GRADIENTS		rfp1	0
gzlvlE	5154	PLOT	
gtE	0.001000	wc	206.0
EDratio	1.000	sc	0
gstab	0.000500	wc2	206.0
DECOUPLER		sc2	0
dn	C13	vs	126
dm	nnn	th	6
		ai cdc av	

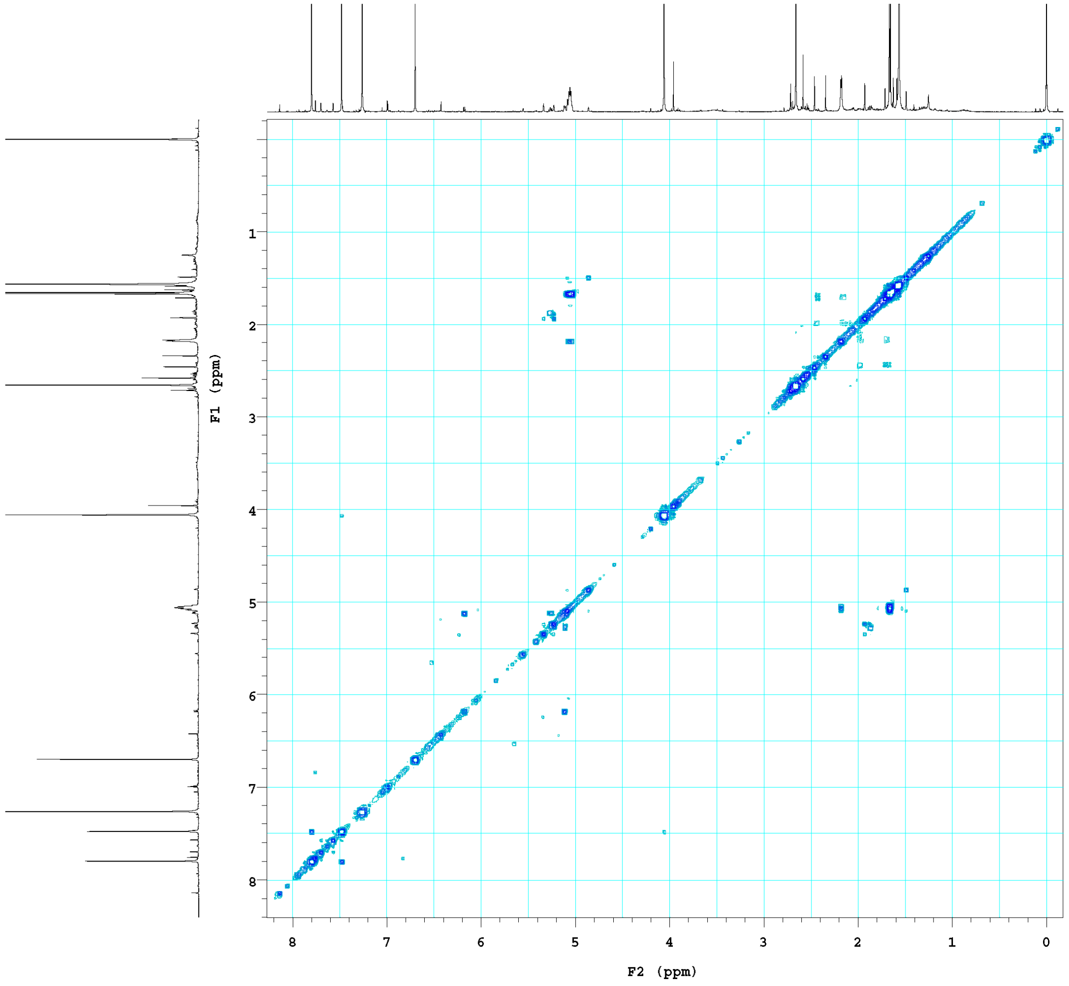

Figure S67. HSQC spectrum of **31** (measured in $CDCl_3$, 500 MHz).

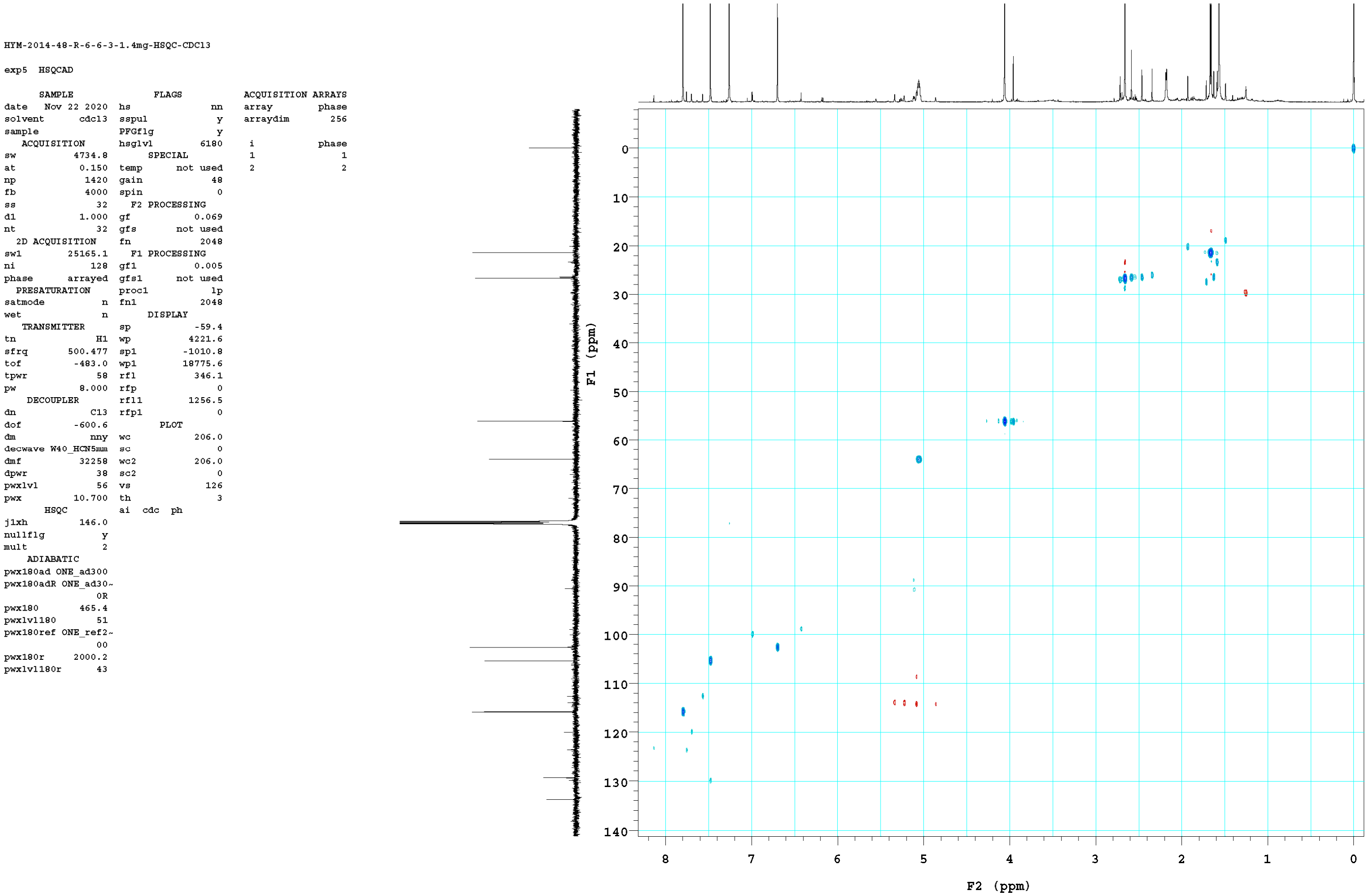

Figure S68. HMBC spectrum of **31** (measured in $CDCl_3$, 500 MHz).

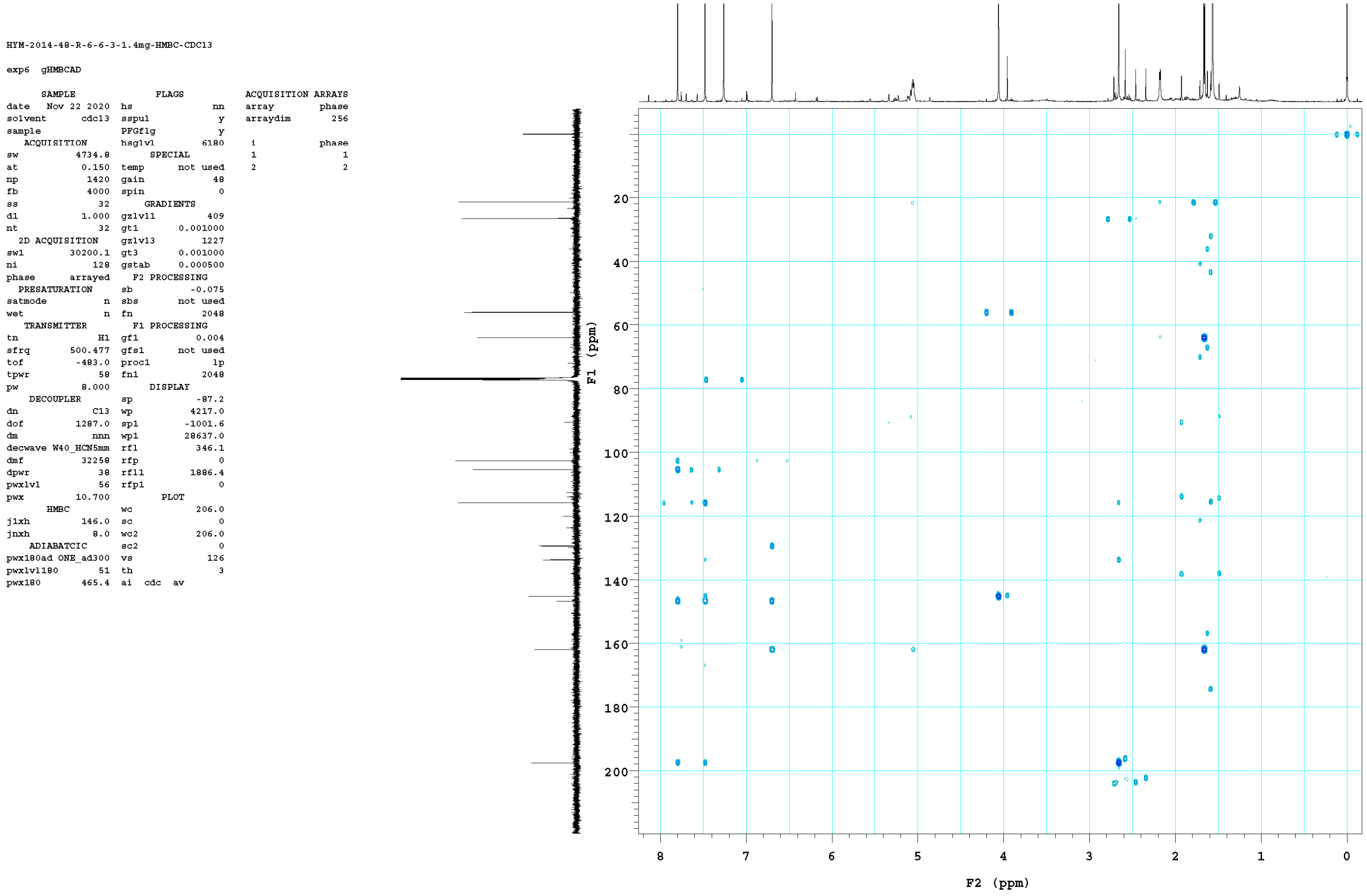

Figure S69. NOESY spectrum of **31** (measured in $CDCl_3$, 500 MHz).

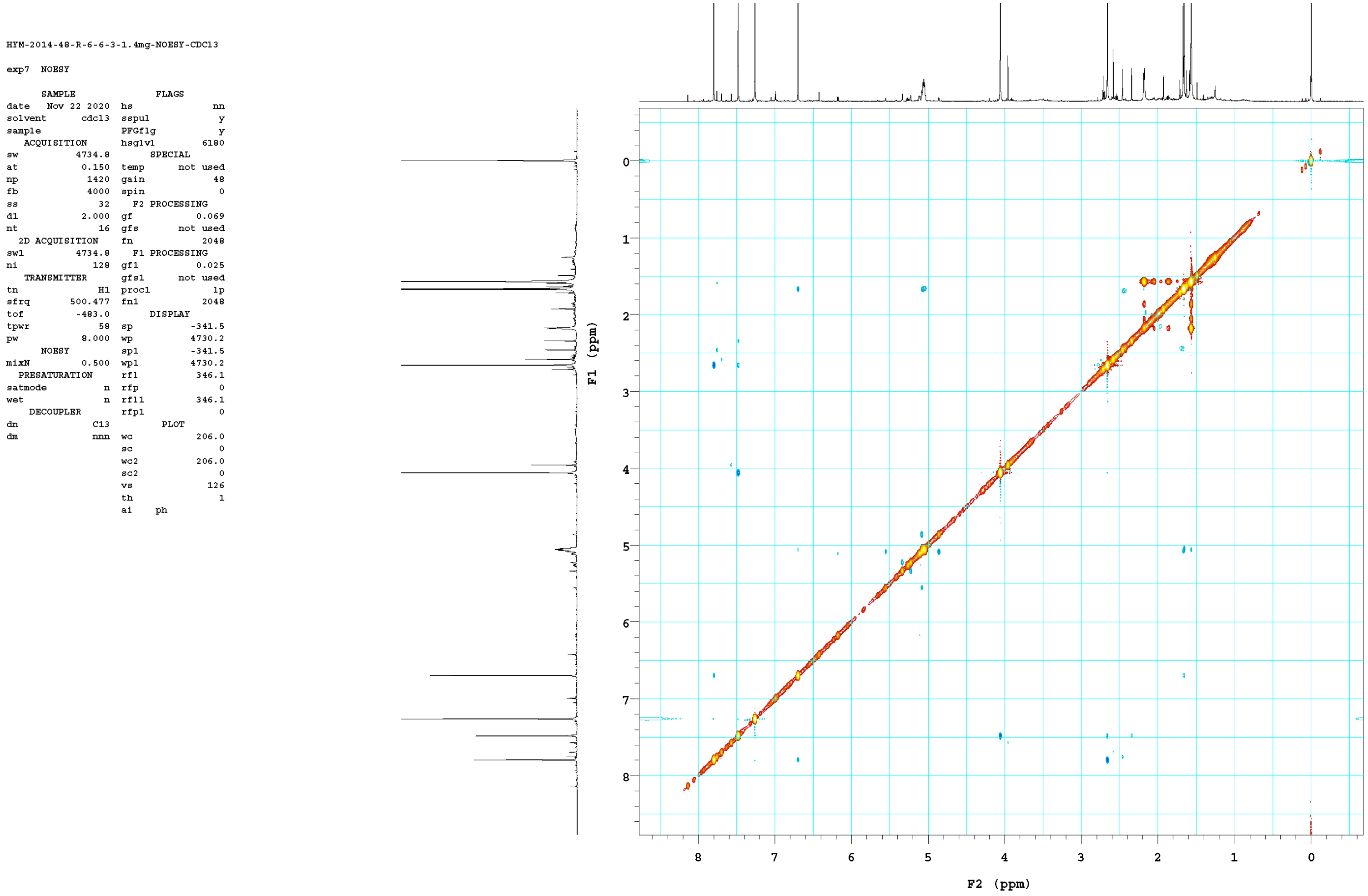

Figure S70. ^{1}H NMR spectrum of **32** (measured in $CDCl_3$, 500 MHz).

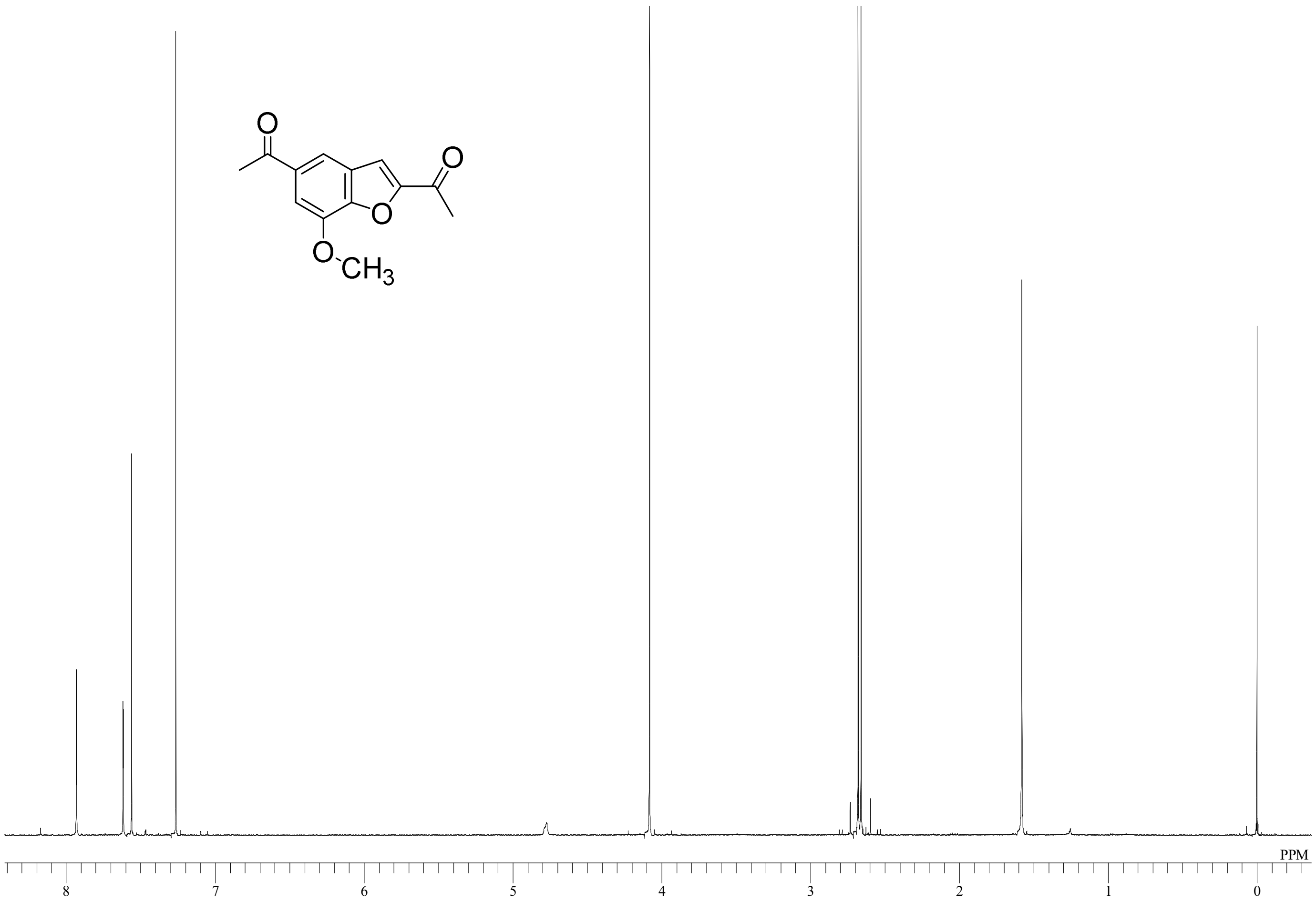

Figure S71. ^{13}C NMR spectrum of **32** (measured in $CDCl_3$, 126 MHz).

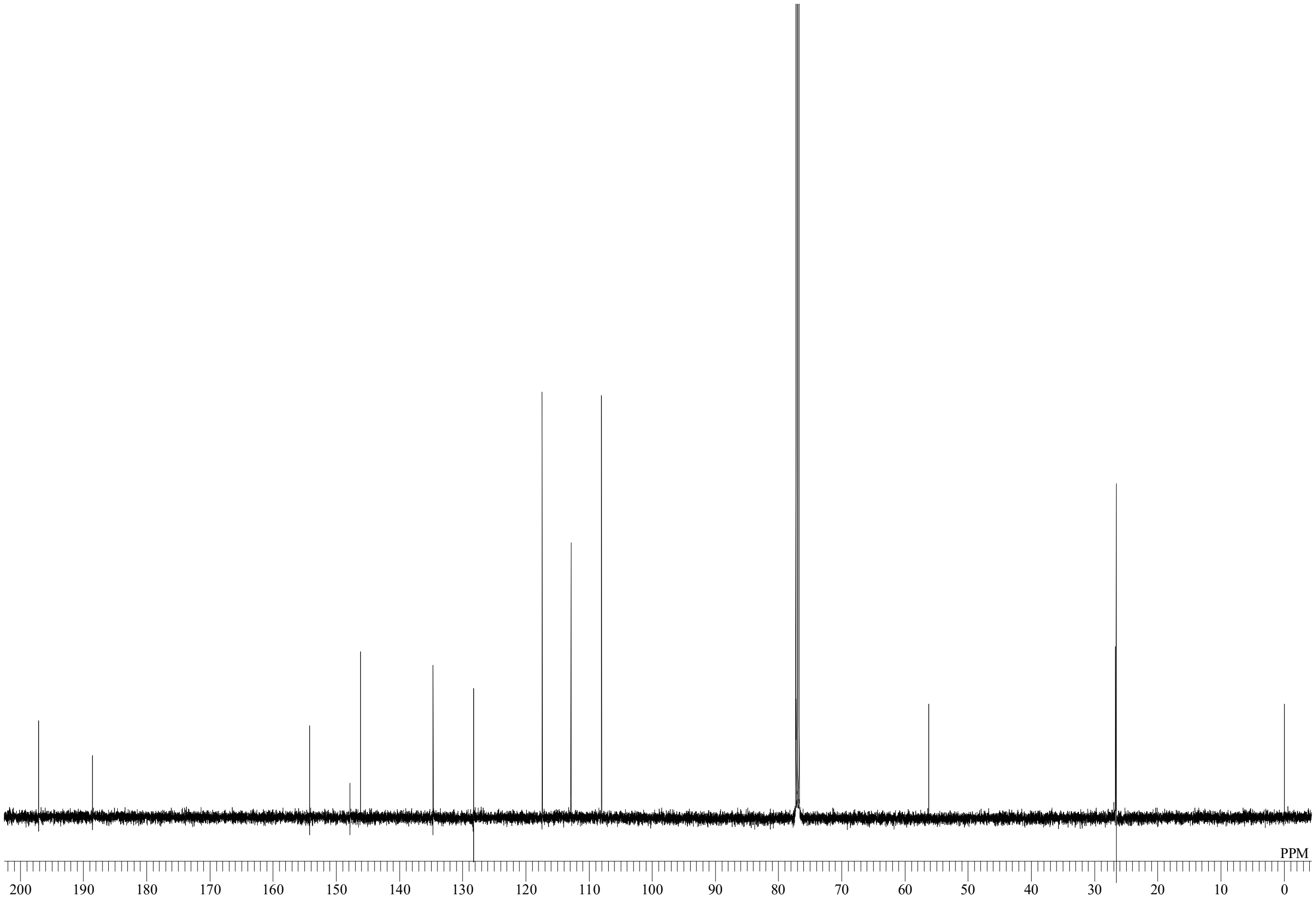

Figure S72. ^{1}H-^{1}H COSY spectrum of **32** (measured in $CDCl_3$, 500 MHz).

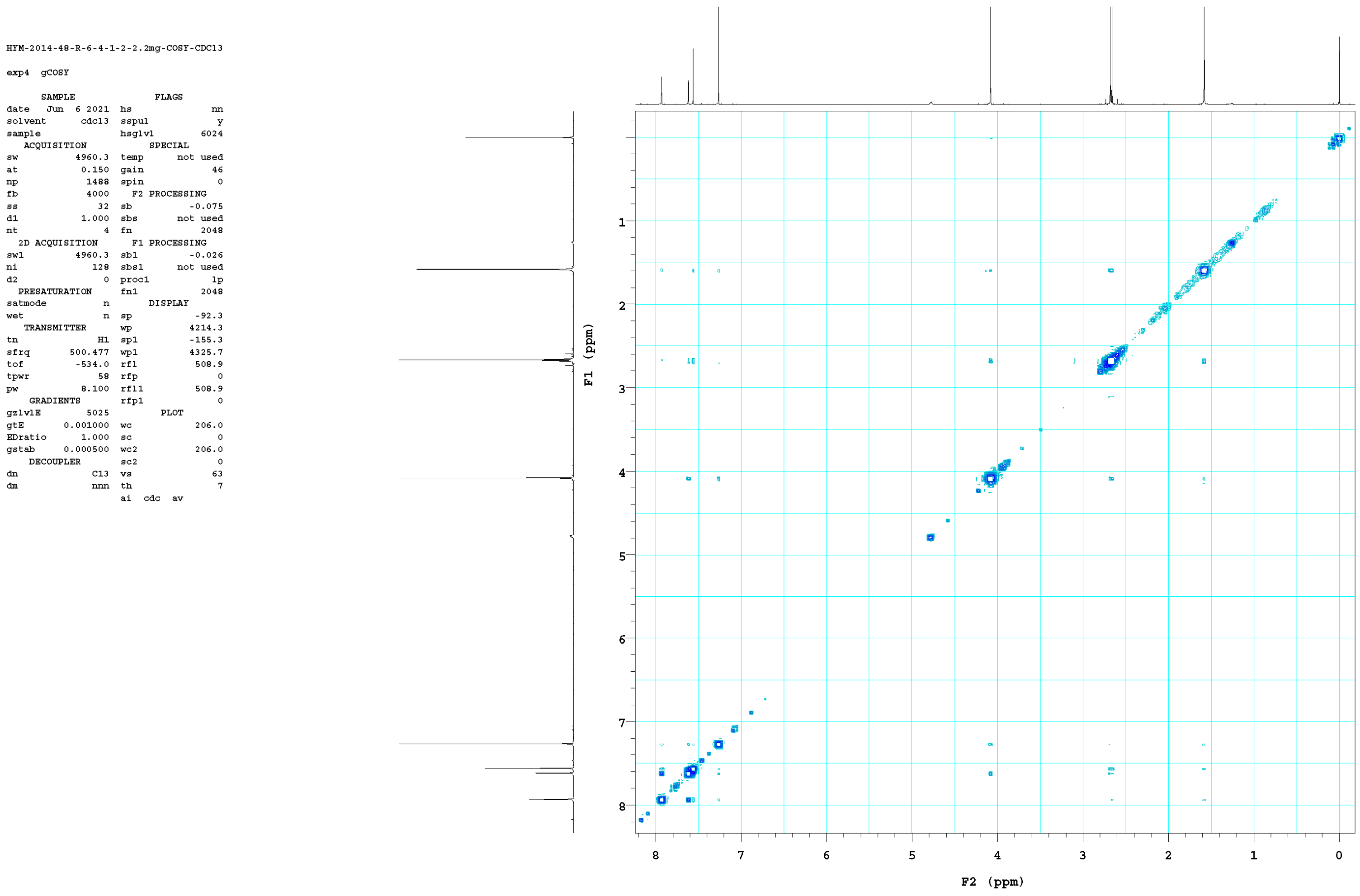

Figure S73. HSQC spectrum of **32** (measured in $CDCl_3$, 500 MHz).

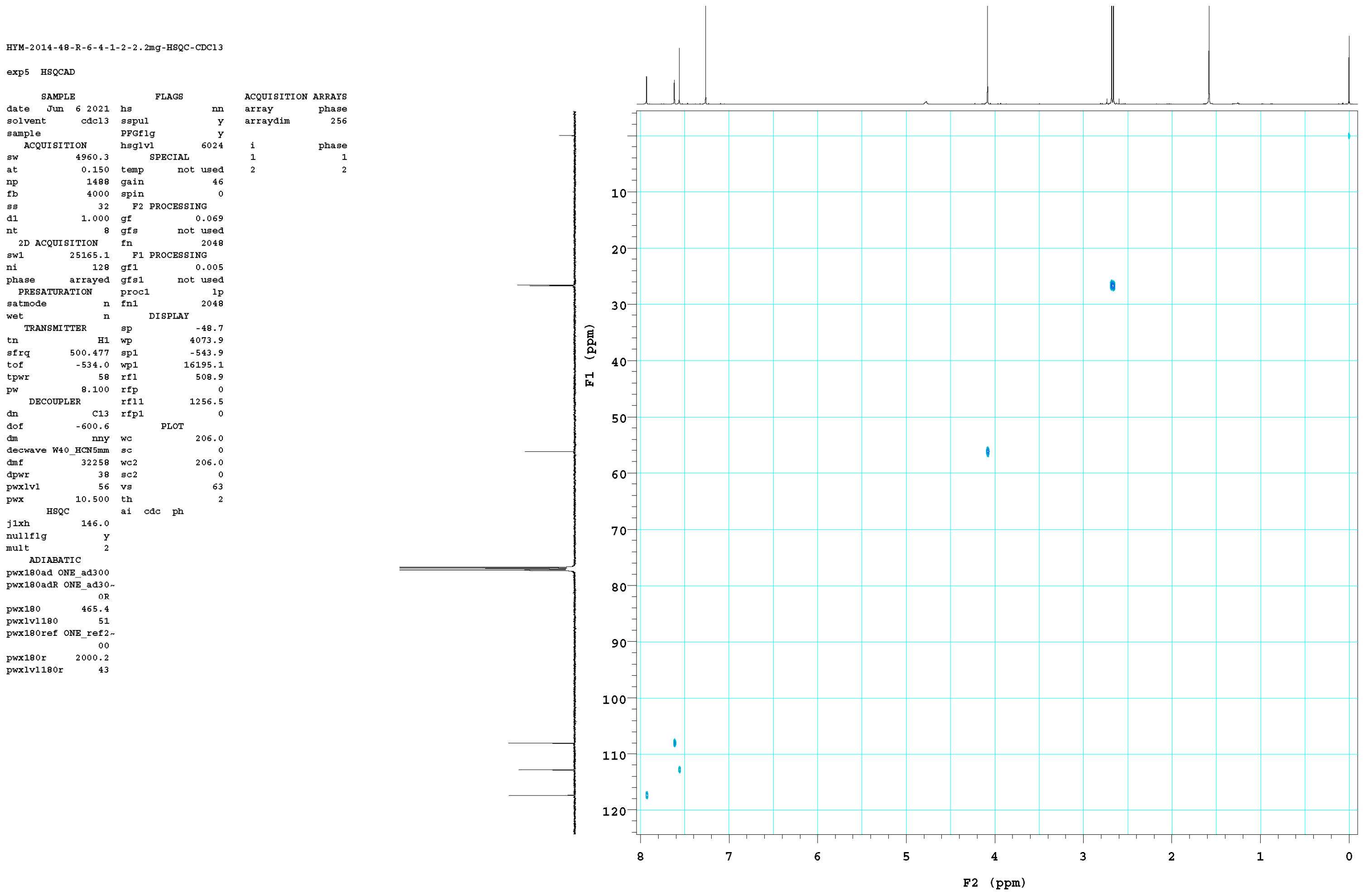

Figure S74. HMBC spectrum of **32** (measured in $CDCl_3$, 500 MHz).

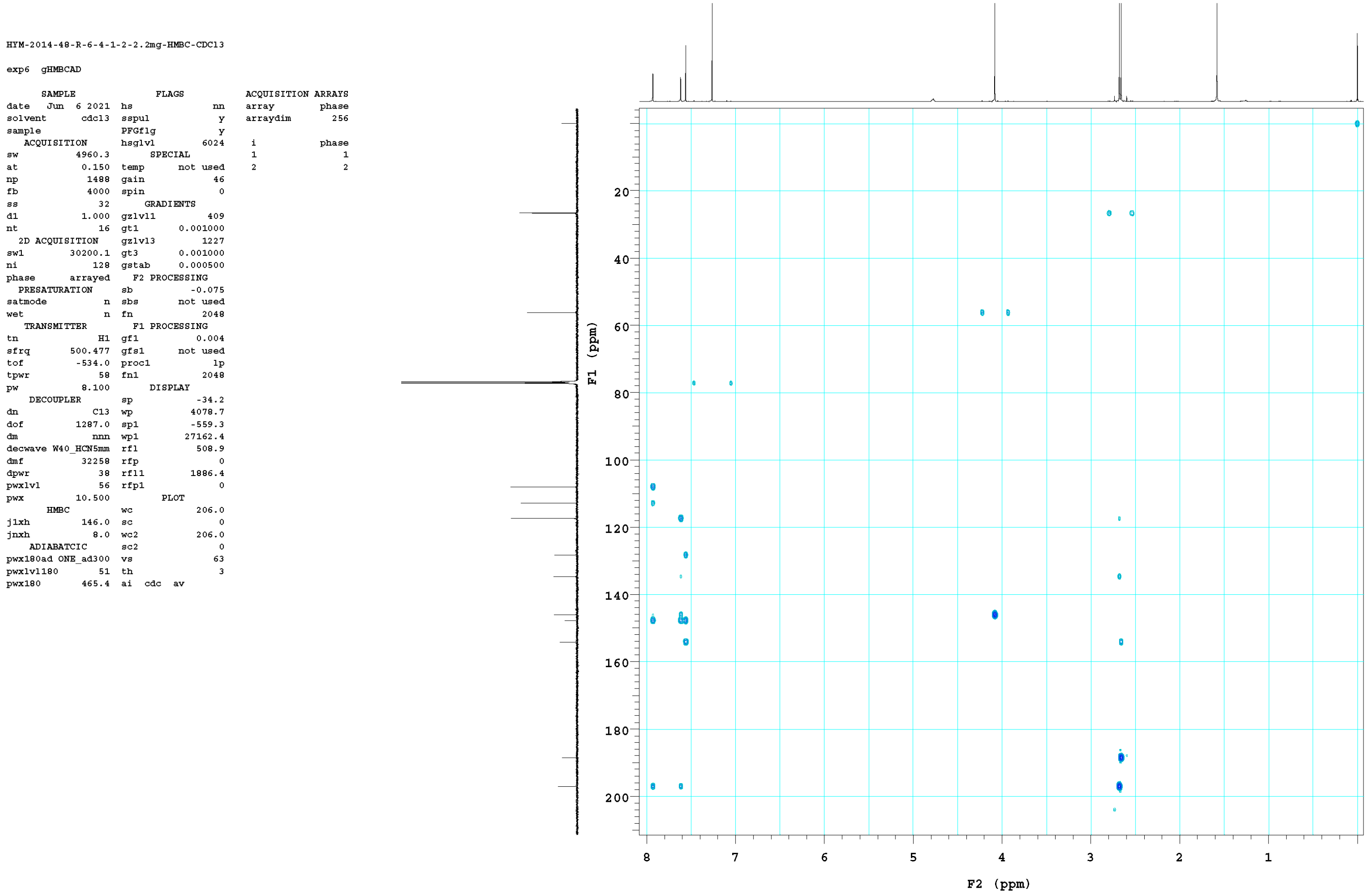

Figure S75. NOESY spectrum of **32** (measured in $CDCl_3$, 500 MHz).

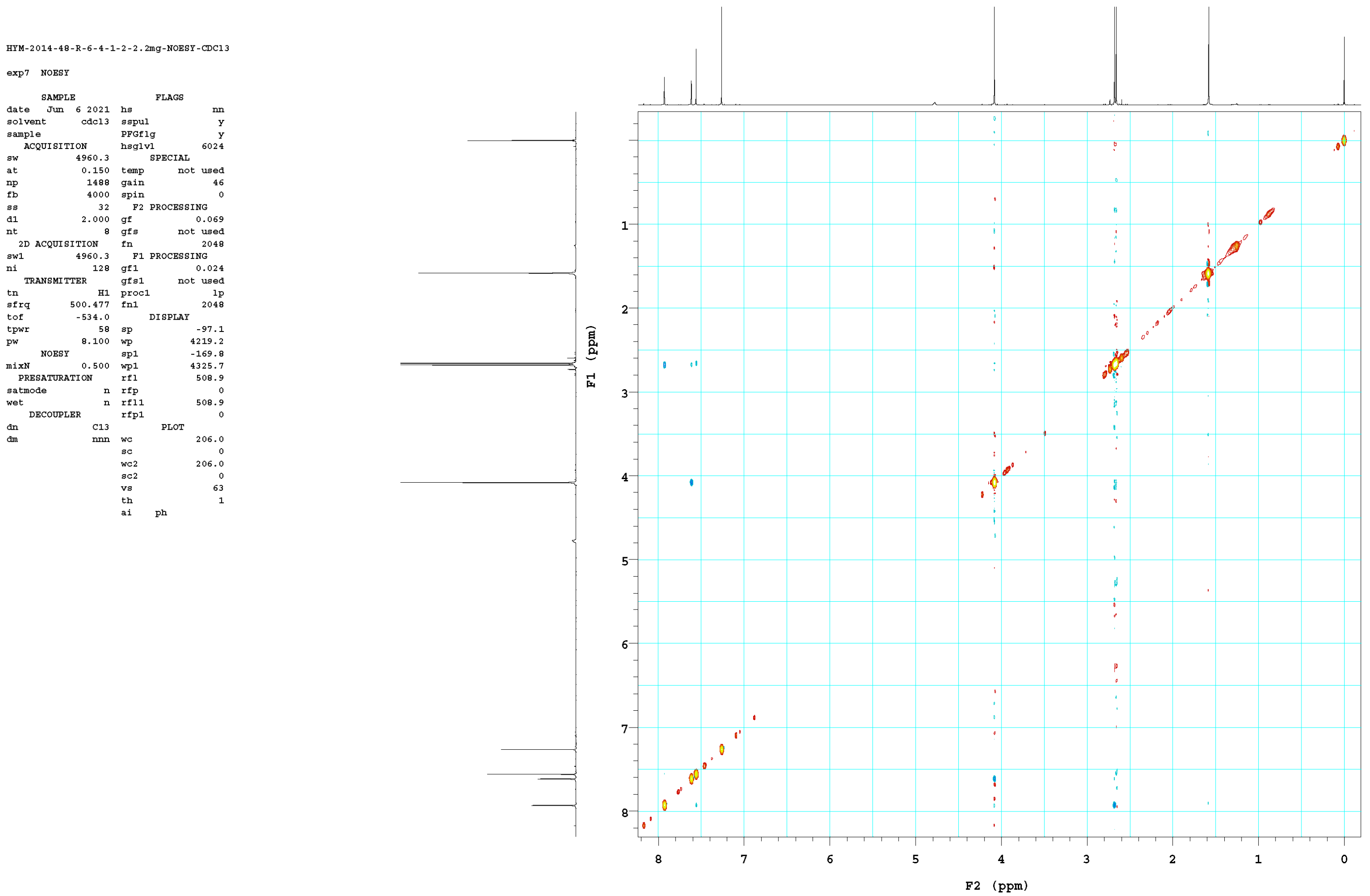

Figure S76. ^{1}H NMR spectrum of **34** (measured in $CDCl_3$, 500 MHz).

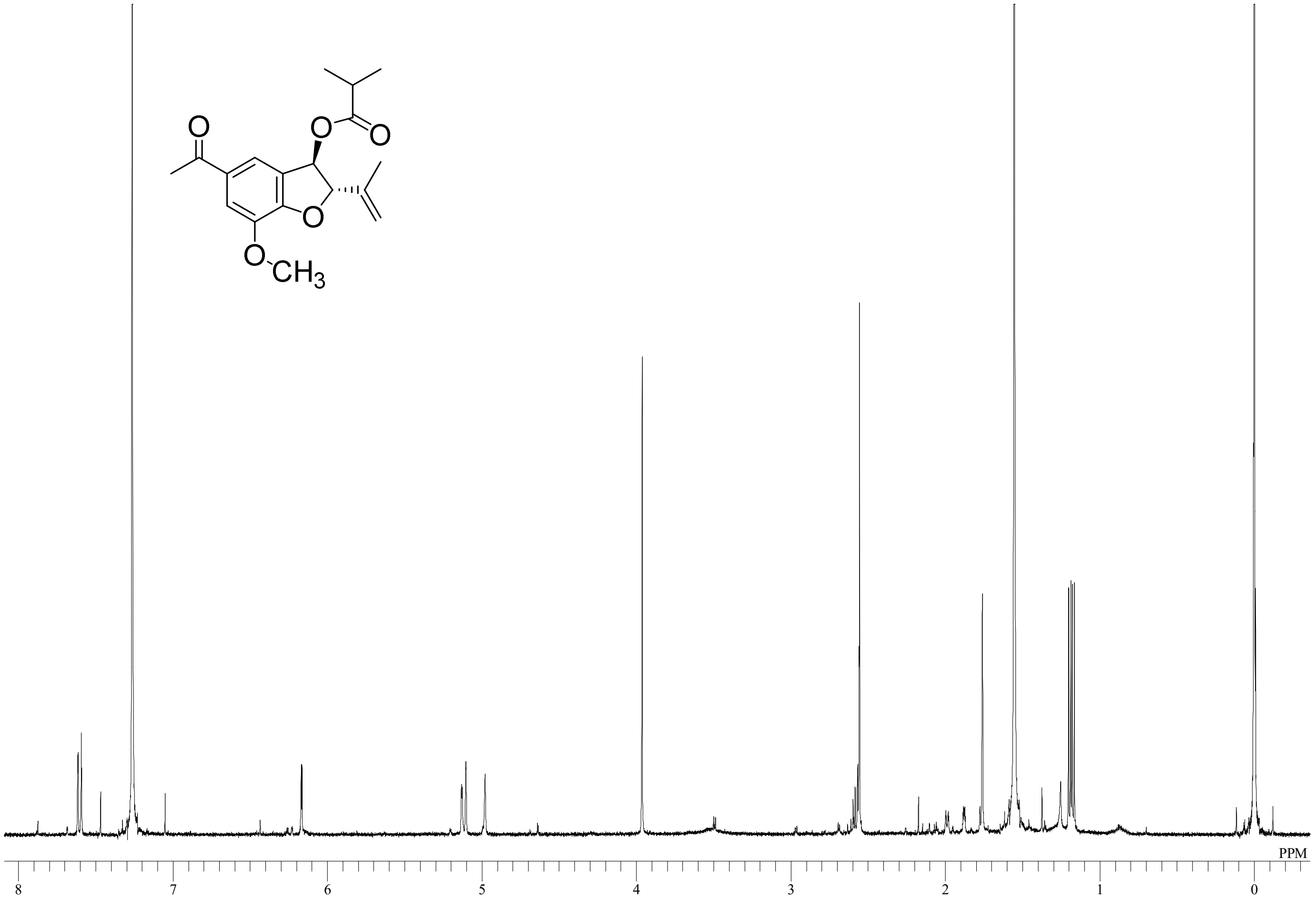

Figure S77. ^{13}C NMR spectrum of **34** (measured in $CDCl_3$, 126 MHz).

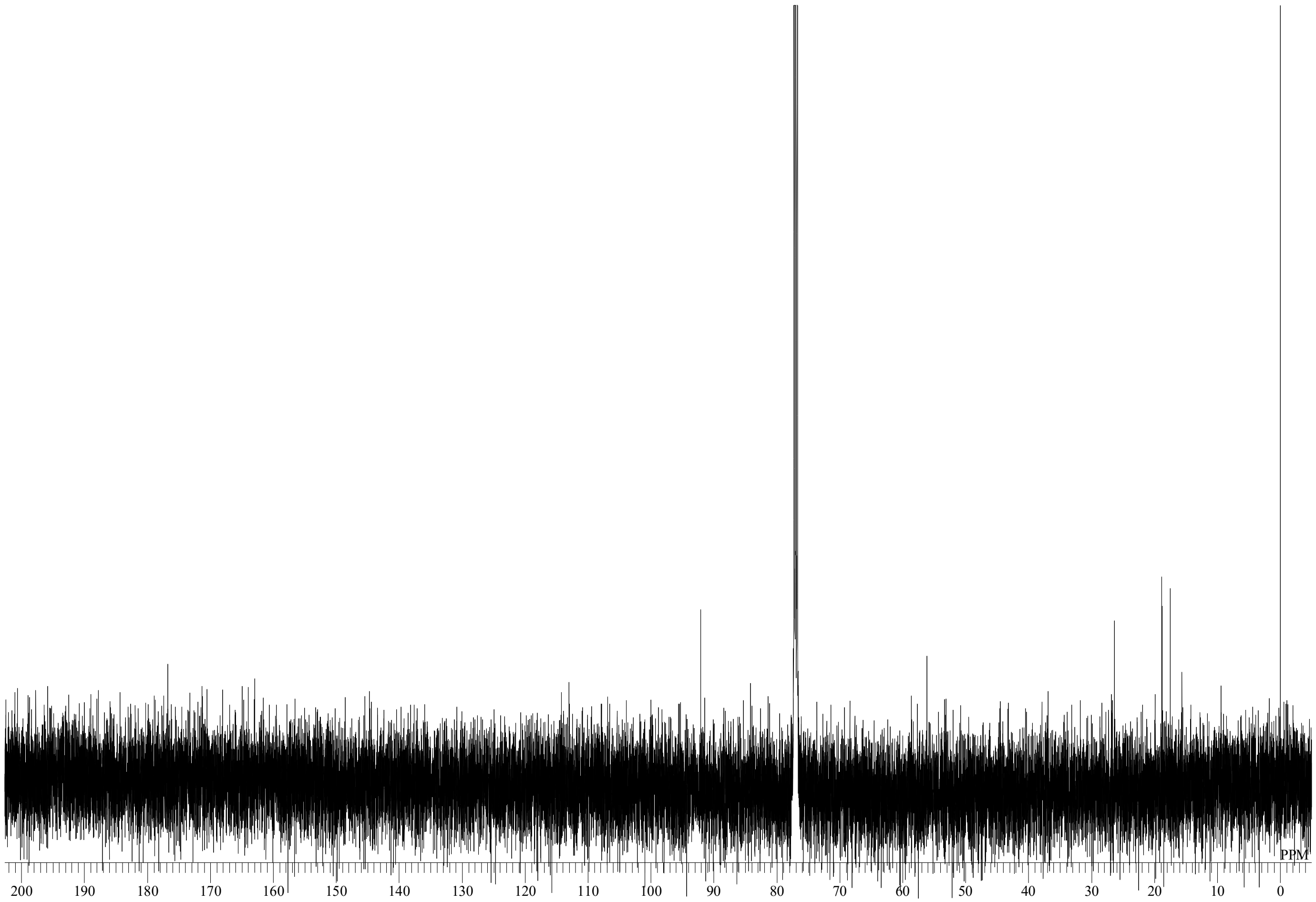

Figure S78. ^{1}H-^{1}H COSY spectrum of **34** (measured in $CDCl_3$, 500 MHz).

```
HYM-2014-48-R-4-2-4-2-0.2mg-COSY-CDCl3

exp4  gCOSY

      SAMPLE                FLAGS
date    Oct 30 2020   hs               nn
solvent       cdcl3   sspul             y
sample                hsglvl         6180
   ACQUISITION           SPECIAL
sw           5060.7   temp       not used
at            0.150   gain             54
np             1518   spin              0
fb             4000     F2 PROCESSING
ss               32   sb           -0.075
d1            1.000   sbs        not used
nt               16   fn             2048
  2D ACQUISITION        F1 PROCESSING
sw1          5060.7   sb1          -0.025
ni              128   sbs1       not used
d2                0   proc1            lp
  PRESATURATION       fn1            2048
satmode           n        DISPLAY
wet               n   sp            -98.0
   TRANSMITTER        wp           4215.6
tn               H1   sp1          -147.4
sfrq        500.477   wp1          4556.6
tof          -511.8   rfl           537.9
tpwr             58   rfp               0
pw            8.000   rfl1          537.9
    GRADIENTS         rfp1              0
gzlvlE         5154          PLOT
gtE        0.001000   wc            206.0
EDratio       1.000   sc                0
gstab      0.000500   wc2           206.0
    DECOUPLER         sc2               0
dn              C13   vs               34
dm              nnn   th                6
                      ai   cdc   av
```

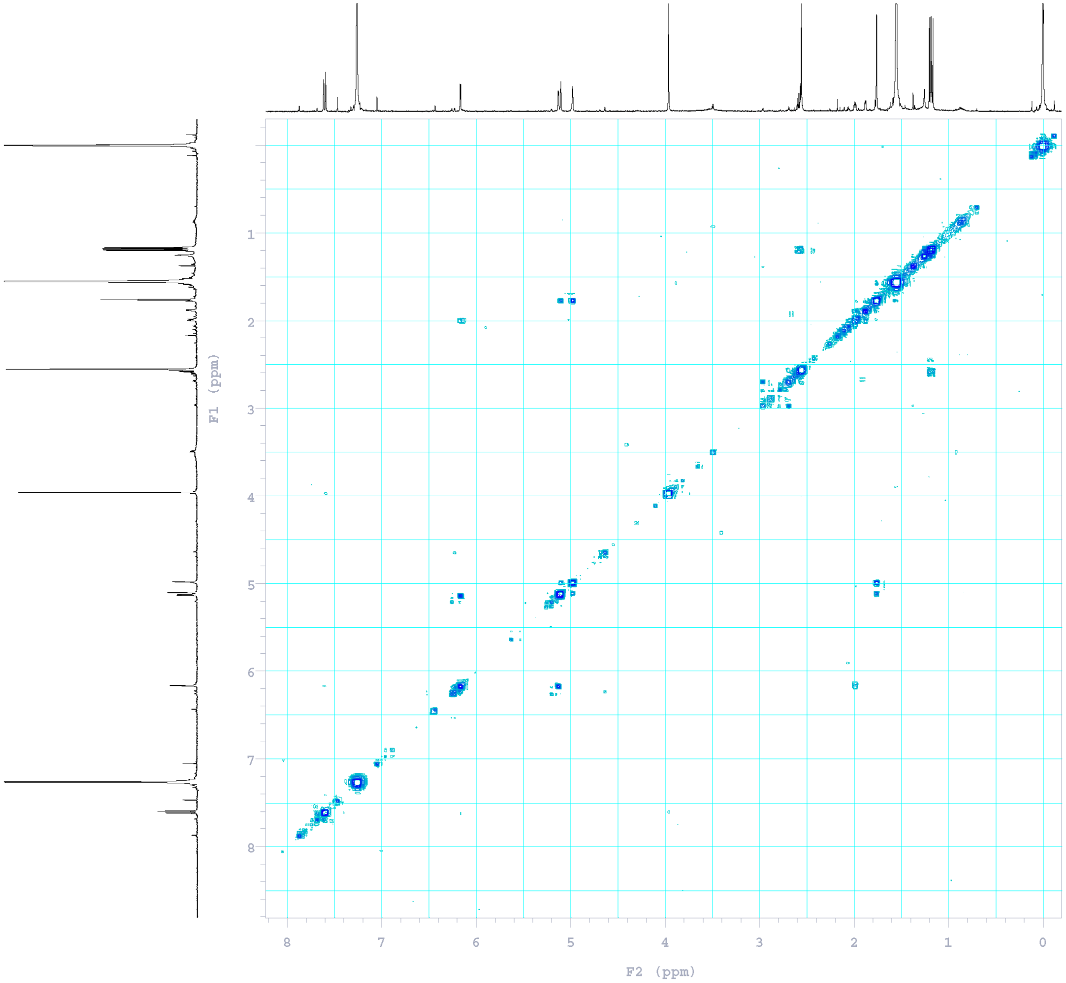

Figure S79. HSQC spectrum of **34** (measured in $CDCl_3$, 500 MHz).

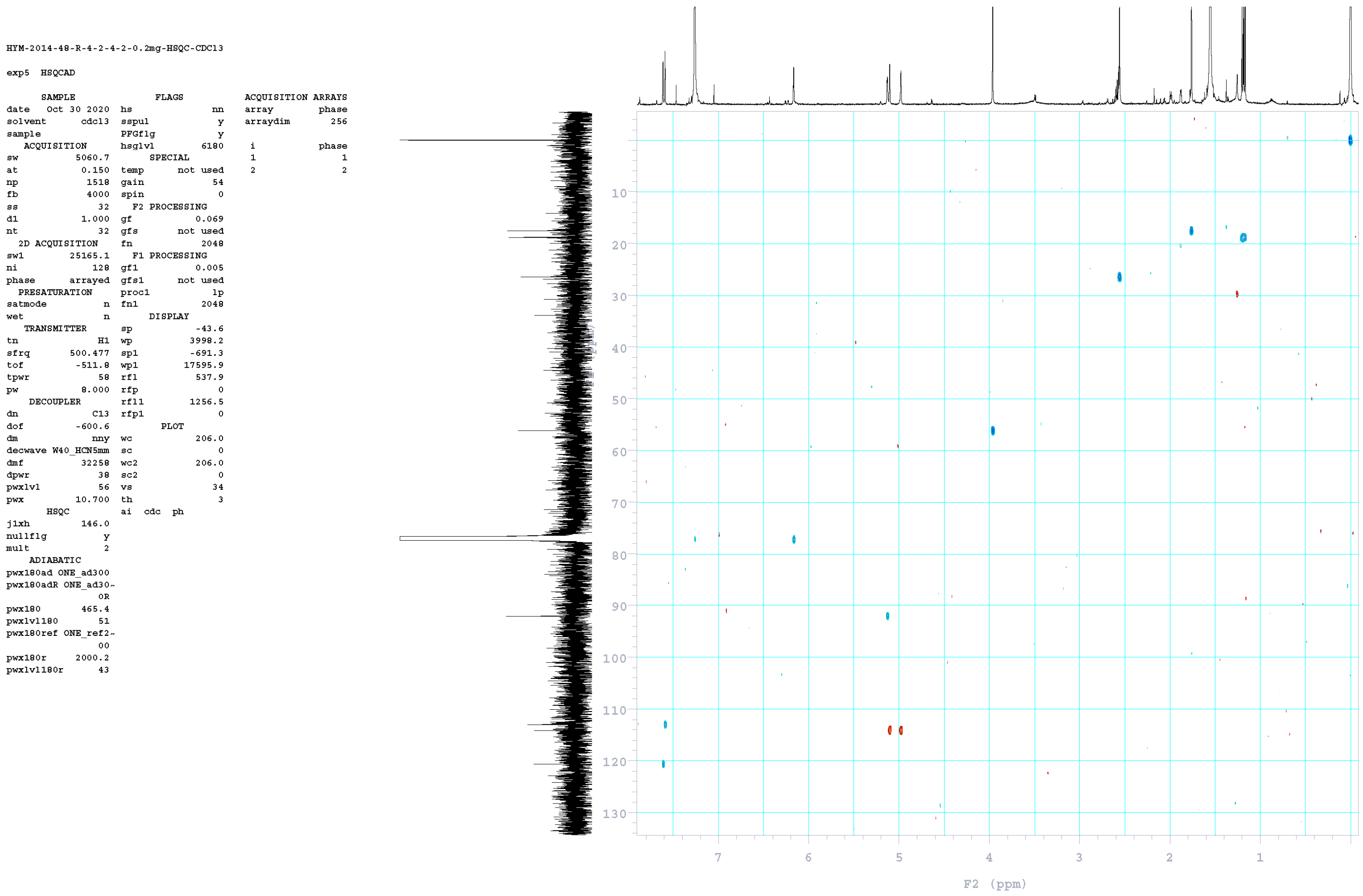

Figure S80. HMBC spectrum of **34** (measured in $CDCl_3$, 500 MHz).

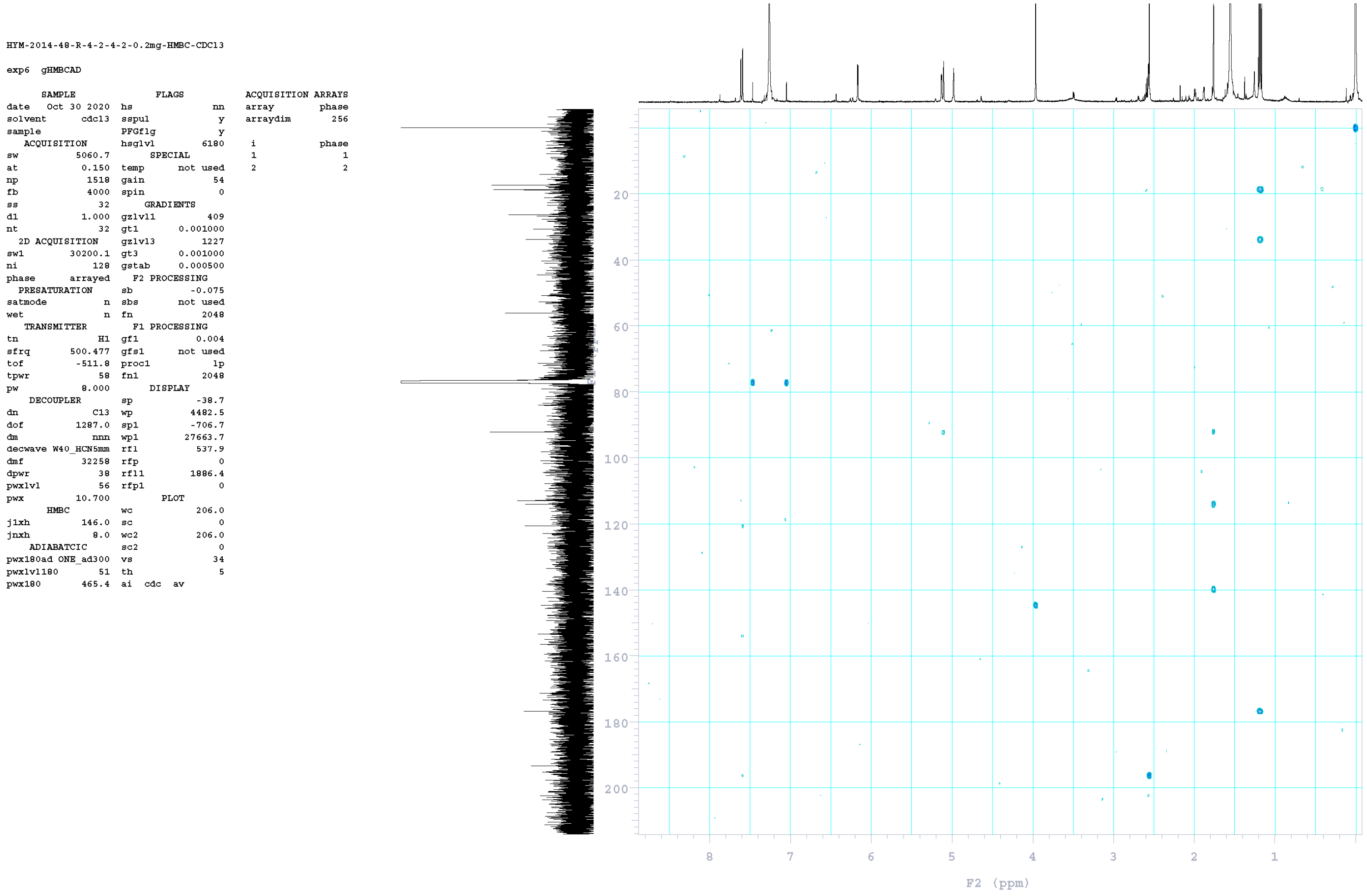

Figure S81. NOESY spectrum of **34** (measured in $CDCl_3$, 500 MHz).

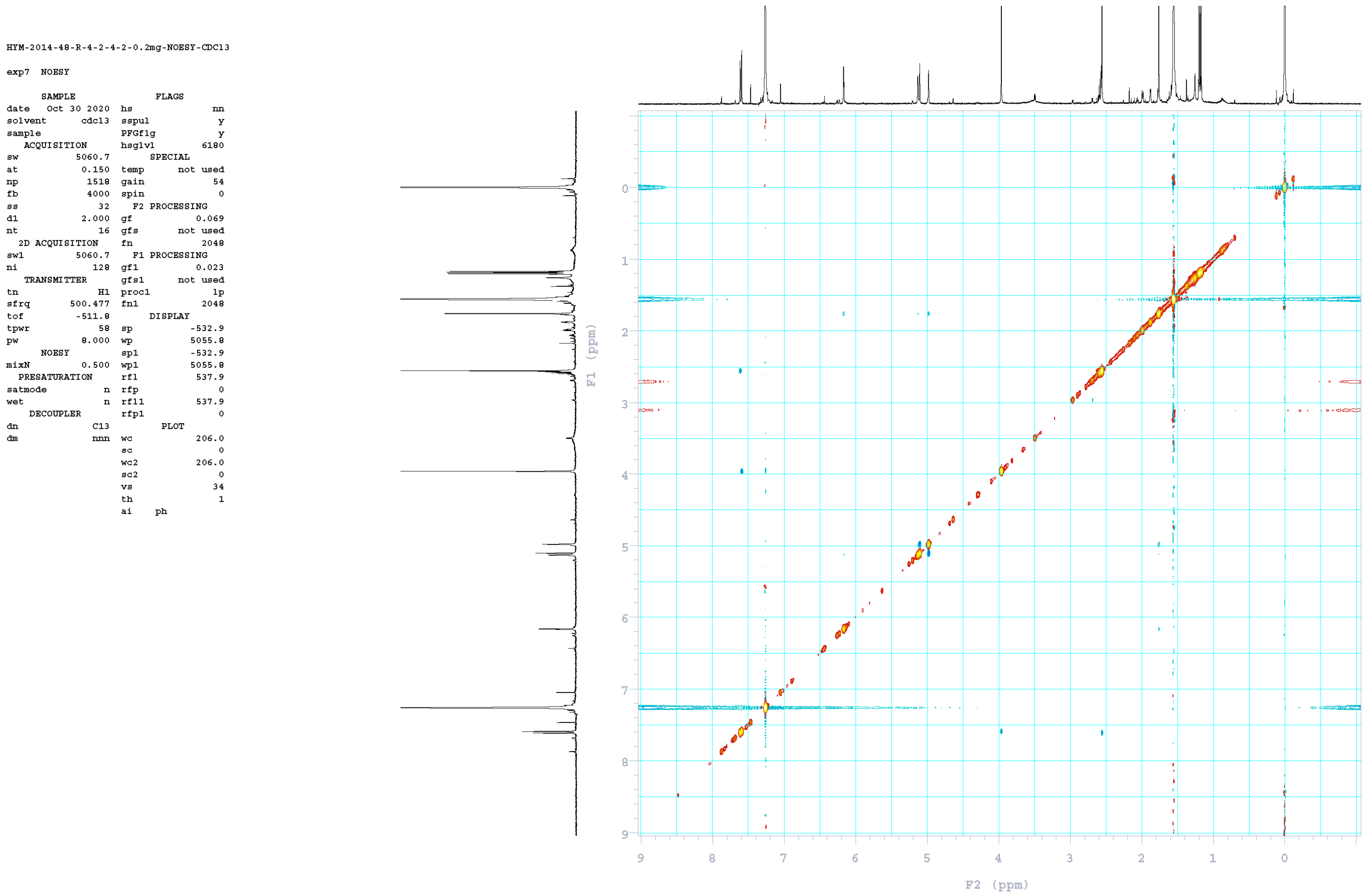

Figure S82. ^{1}H NMR spectrum of **36** (measured in $CDCl_3$, 500 MHz).

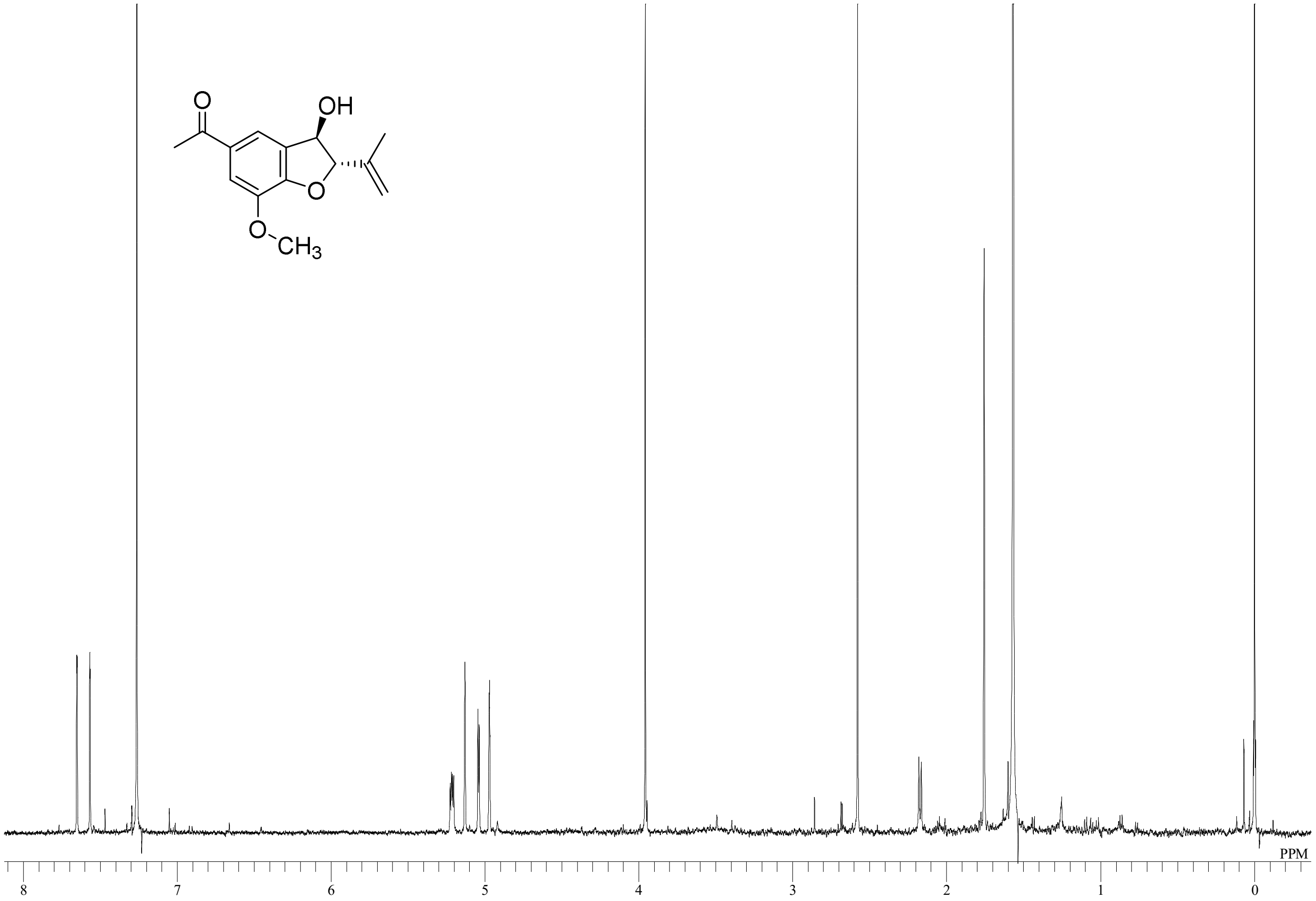

Figure S83. ^{1}H-^{1}H COSY spectrum of **36** (measured in $CDCl_3$, 500 MHz).

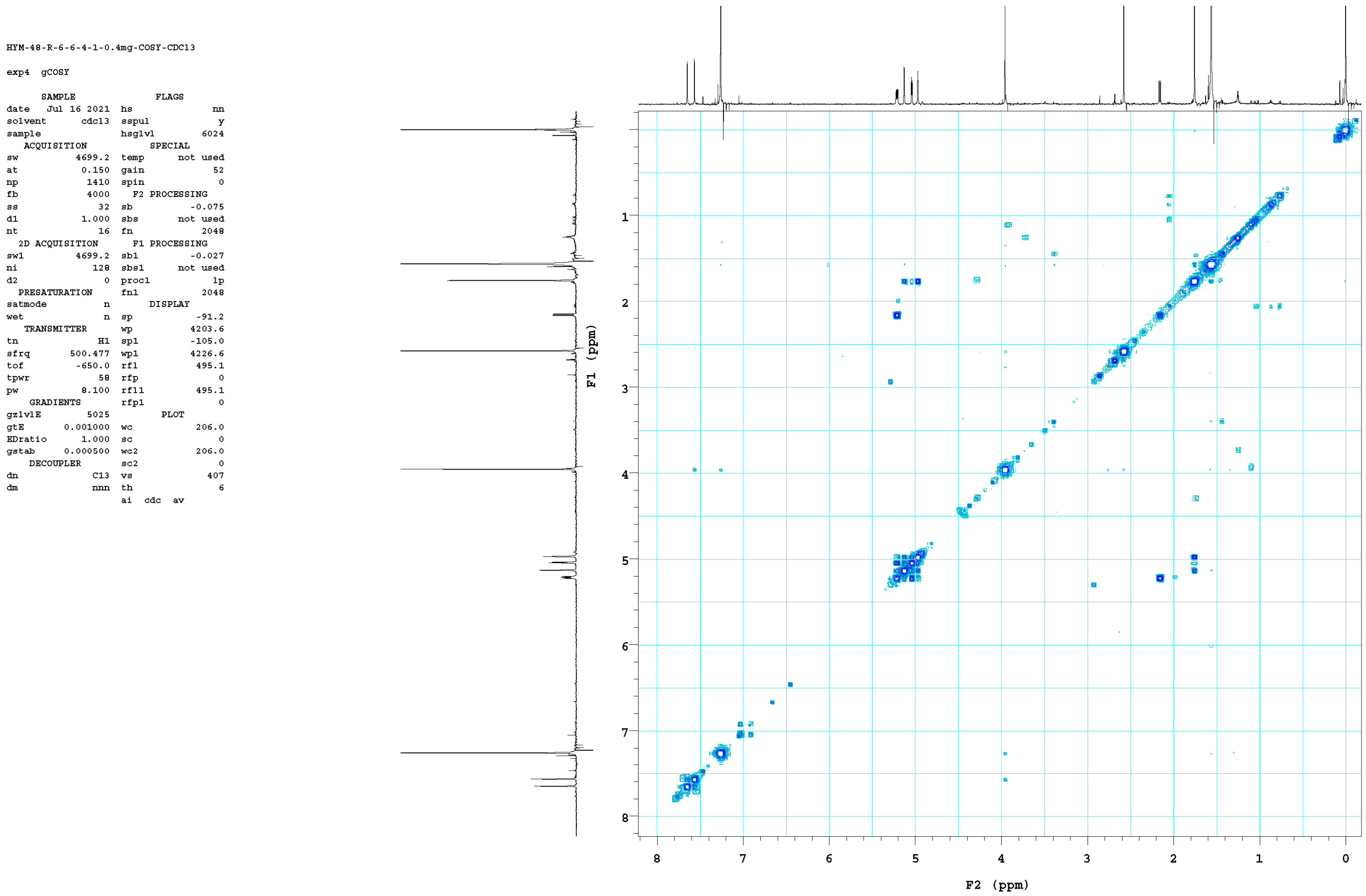

Figure S84. NOESY spectrum of **36** (measured in $CDCl_3$, 500 MHz).

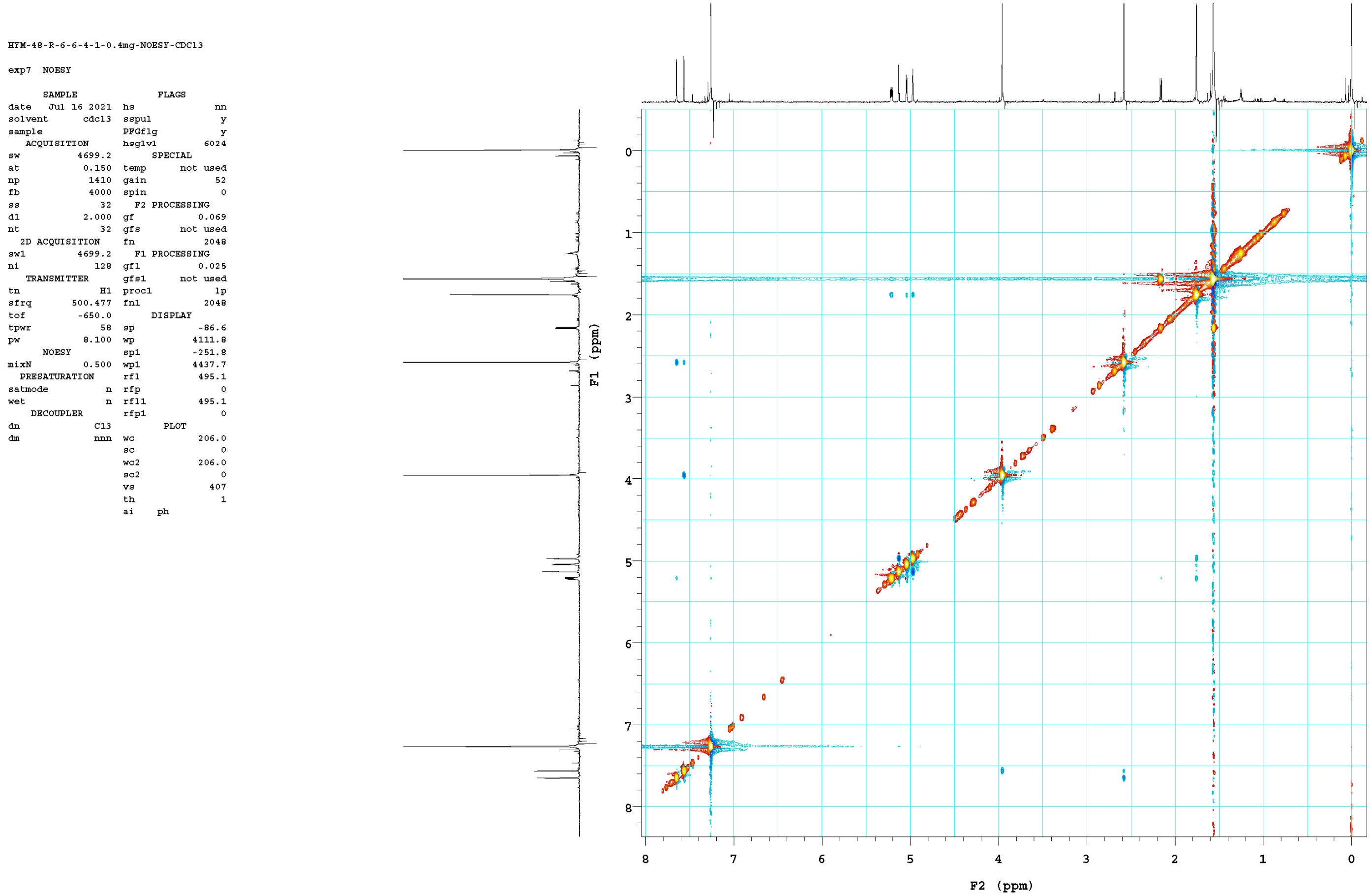

Figure S85. ^{1}H NMR spectrum of **39** (measured in $CDCl_3$, 500 MHz).

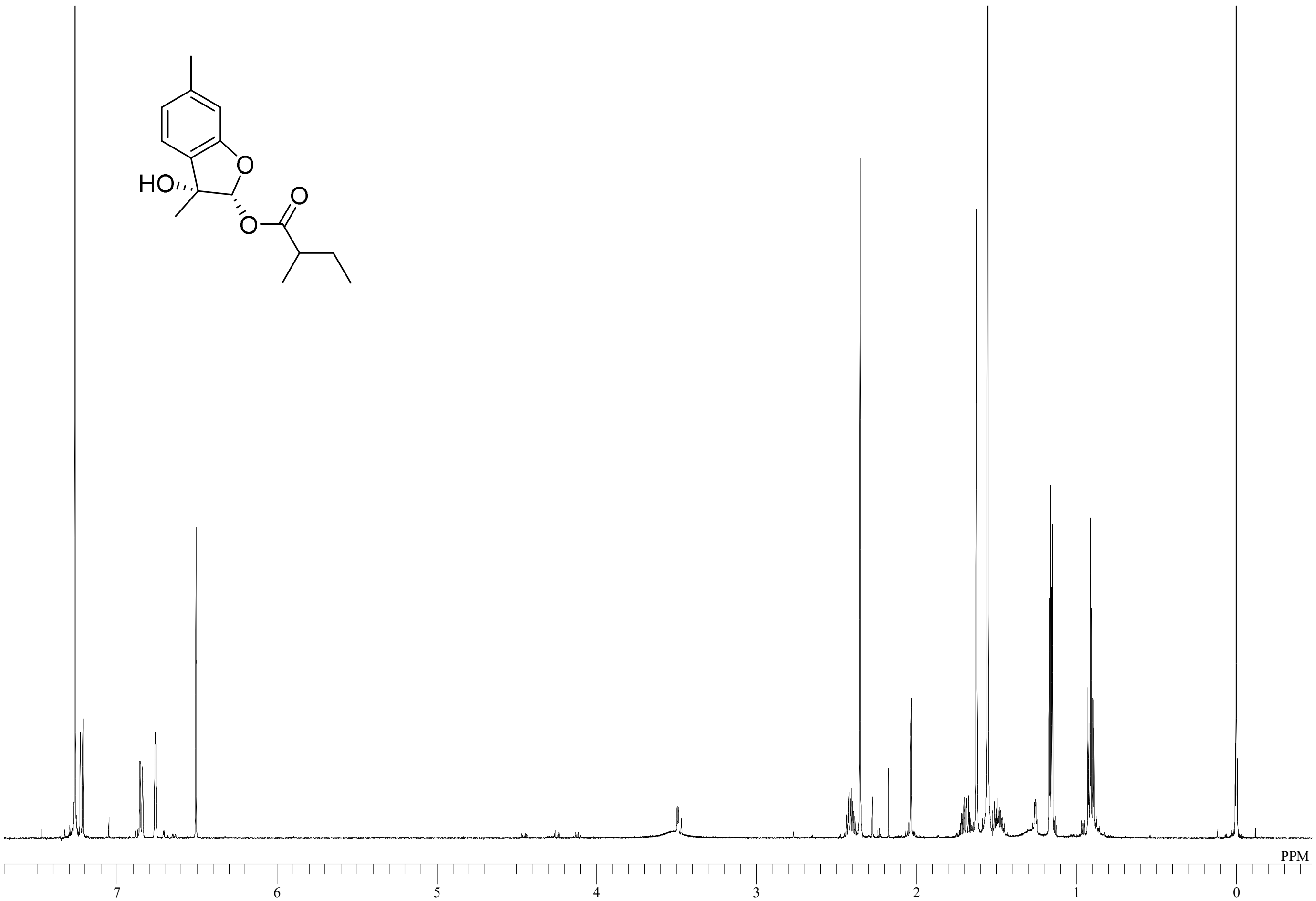

Figure S86. ^{13}C NMR spectrum of **39** (measured in $CDCl_3$, 126 MHz).

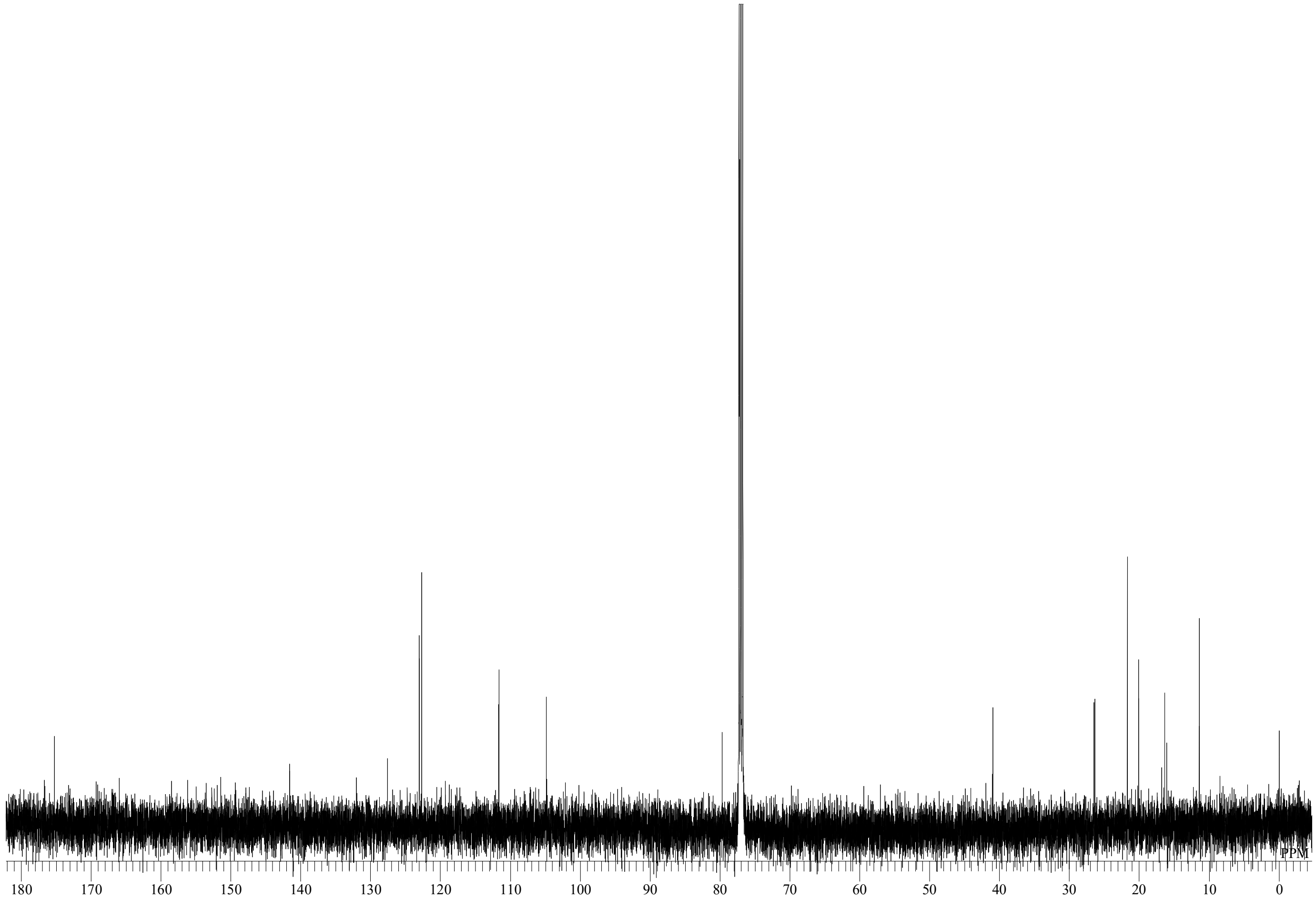

Figure S87. ^{1}H-^{1}H COSY spectrum of **39** (measured in $CDCl_3$, 500 MHz).

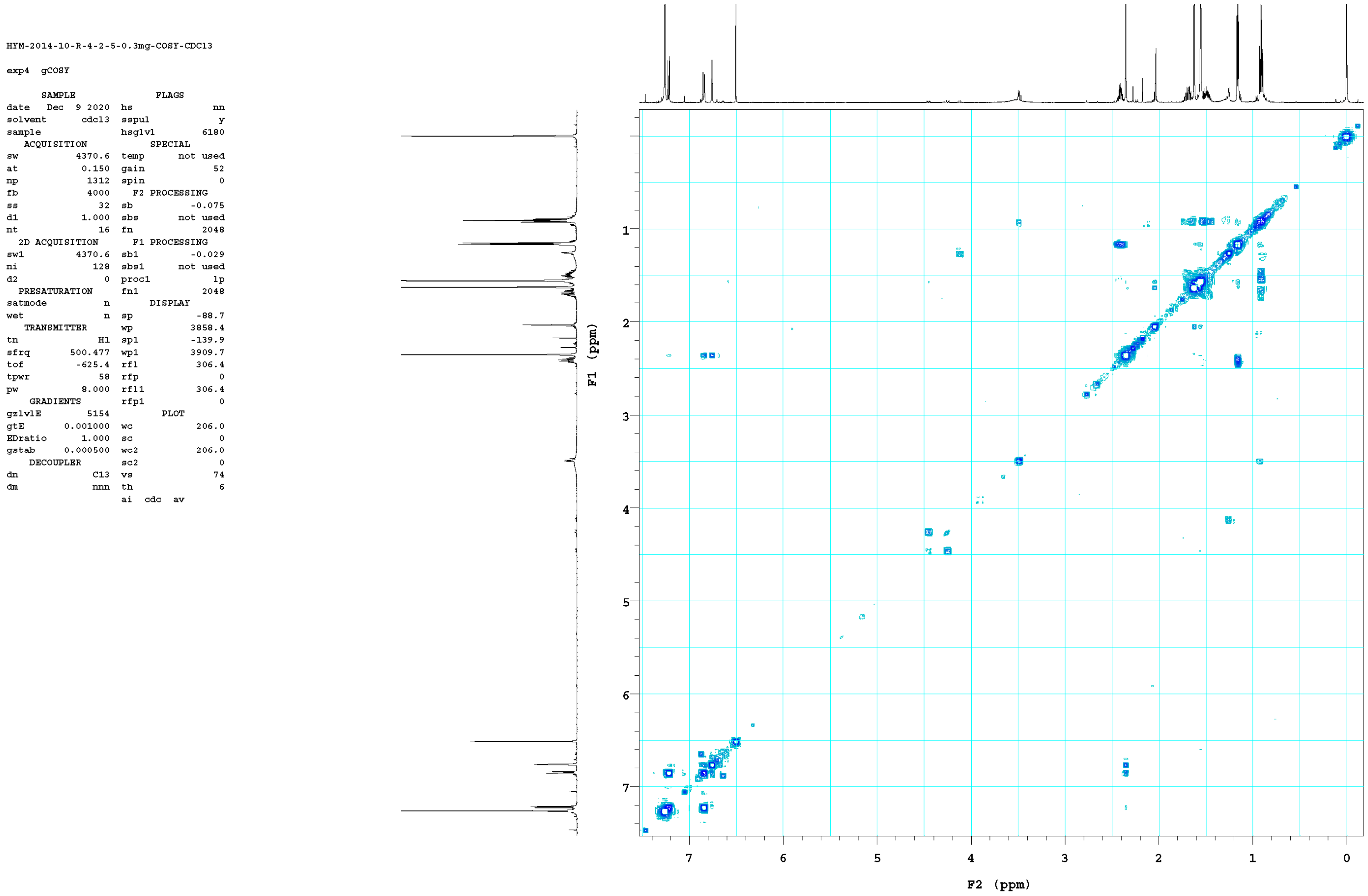

Figure S88. HSQC spectrum of **39** (measured in $CDCl_3$, 500 MHz).

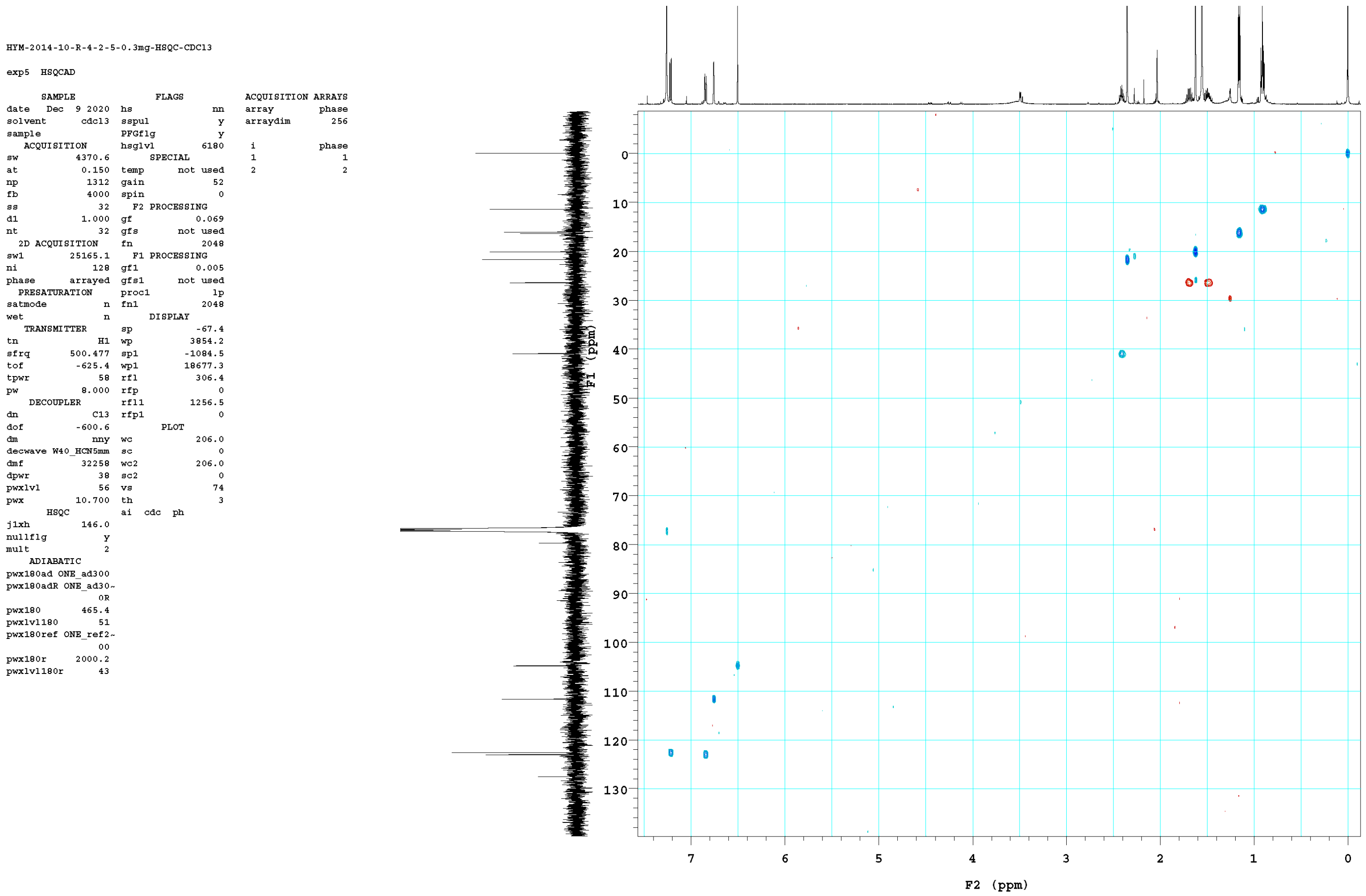

Figure S89. HMBC spectrum of **39** (measured in $CDCl_3$, 500 MHz).

```
HYM-2014-10-R-4-2-5-0.3mg-HMBC-CDCl3

exp6  gHMBCAD

     SAMPLE               FLAGS            ACQUISITION ARRAYS
date   Dec  9 2020  hs                nn   array         phase
solvent      cdcl3  sspul              y   arraydim        256
sample              PFGflg             y
   ACQUISITION      hsglvl          6180    i            phase
sw          4370.6        SPECIAL           1                1
at           0.150  temp        not used    2                2
np            1312  gain              52
fb            4000  spin               0
ss              32       GRADIENTS
d1           1.000  gzlvl1           409
nt              64  gt1         0.001000
  2D ACQUISITION    gzlvl3          1227
sw1        30200.1  gt3         0.001000
ni             128  gstab       0.000500
phase      arrayed    F2 PROCESSING
  PRESATURATION     sb            -0.075
satmode          n  sbs         not used
wet              n  fn              2048
   TRANSMITTER        F1 PROCESSING
tn              H1  gf1            0.004
sfrq       500.477  gfs1        not used
tof         -625.4  proc1             lp
tpwr            58  fn1             2048
pw           8.000       DISPLAY
    DECOUPLER       sp             -41.8
dn             C13  wp            3837.1
dof         1287.0  sp1           -559.3
dm             nnn  wp1          24213.1
decwave W40_HCN5mm  rf1            306.4
dmf          32258  rfp                0
dpwr            38  rf11          1886.4
pwxlvl          56  rfp1               0
pwx         10.700         PLOT
       HMBC         wc             206.0
j1xh         146.0  sc                 0
jnxh           8.0  wc2            206.0
    ADIABATCIC      sc2                0
pwx180ad ONE_ad300  vs                74
pwxlvl180       51  th                 4
pwx180       465.4  ai   cdc   av
```

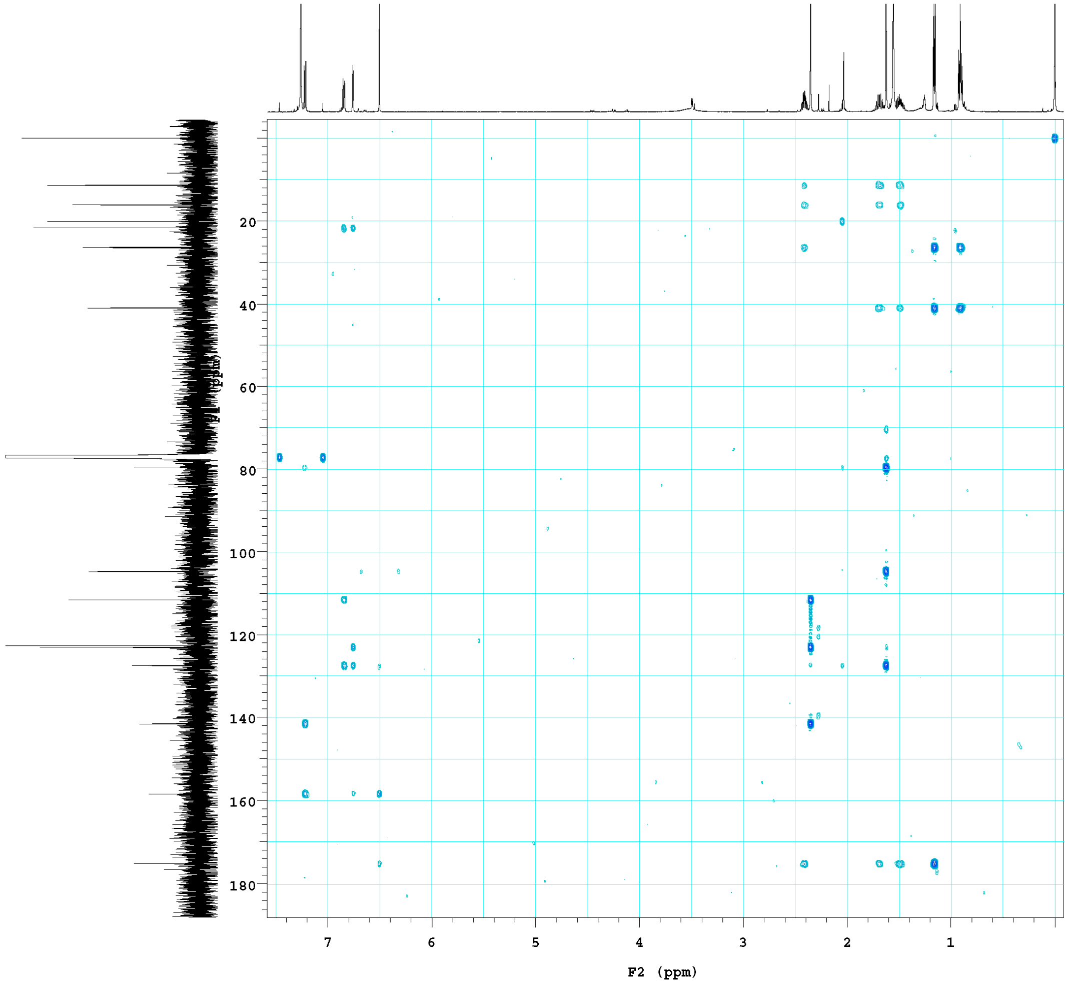

Figure S90. NOESY spectrum of **39** (measured in $CDCl_3$, 500 MHz).

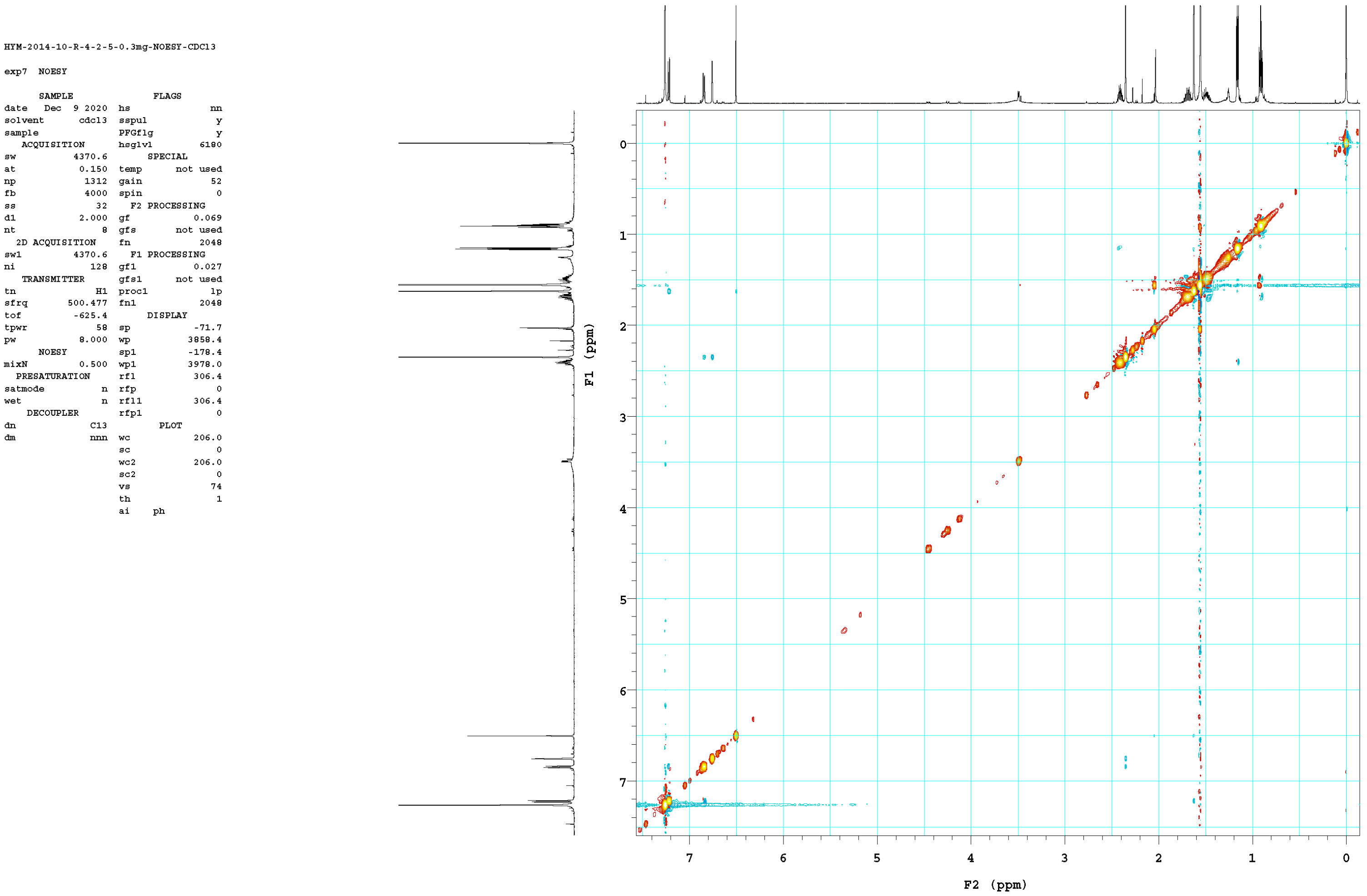

Figure S91. ^{1}H NMR spectrum of **20** (measured in $CDCl_3$, 500 MHz).

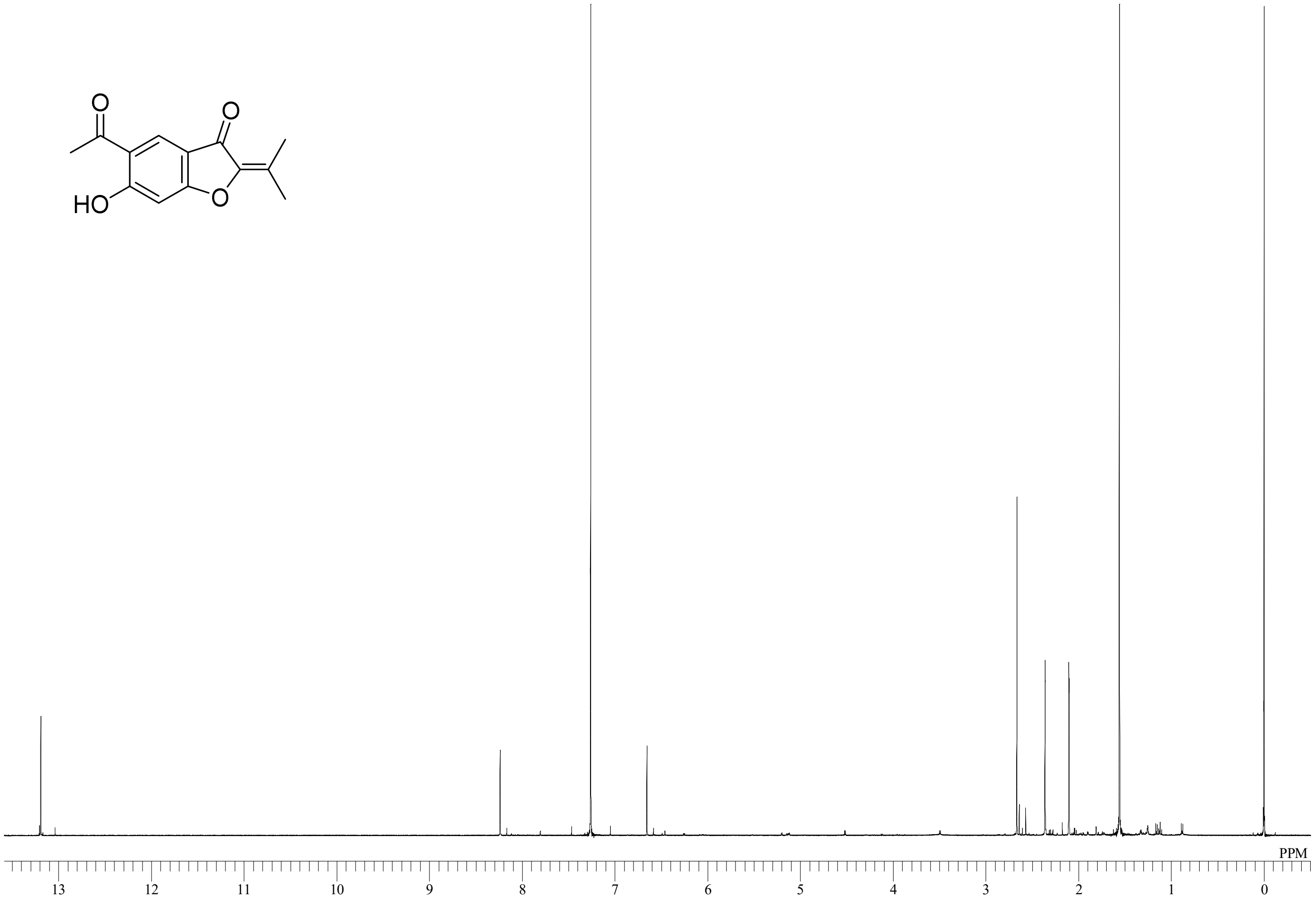

Figure S92. ^{13}C NMR spectrum of **20** (measured in $CDCl_3$, 126 MHz).

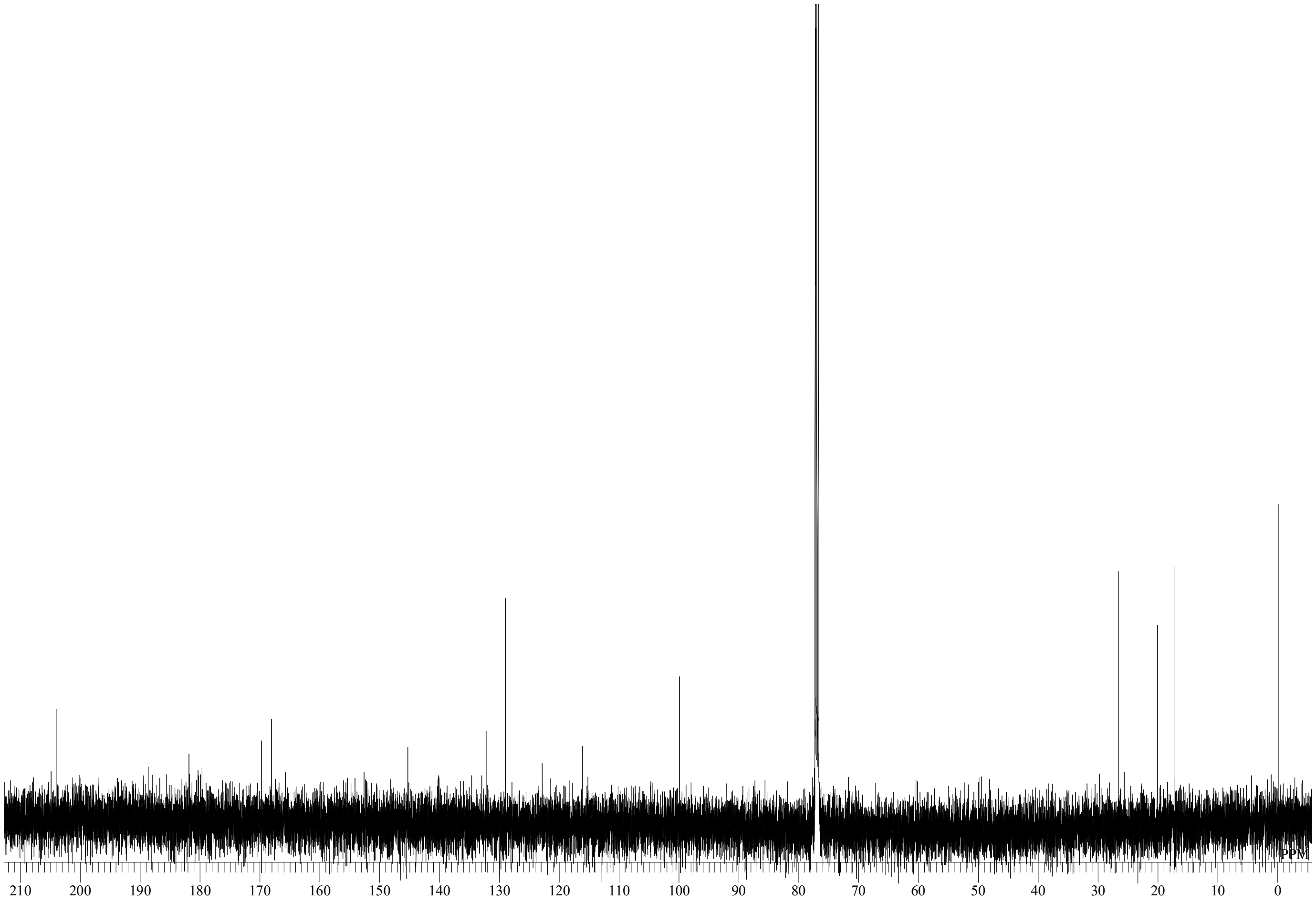

Figure S93. ^{1}H-^{1}H COSY spectrum of **20** (measured in $CDCl_3$, 500 MHz).

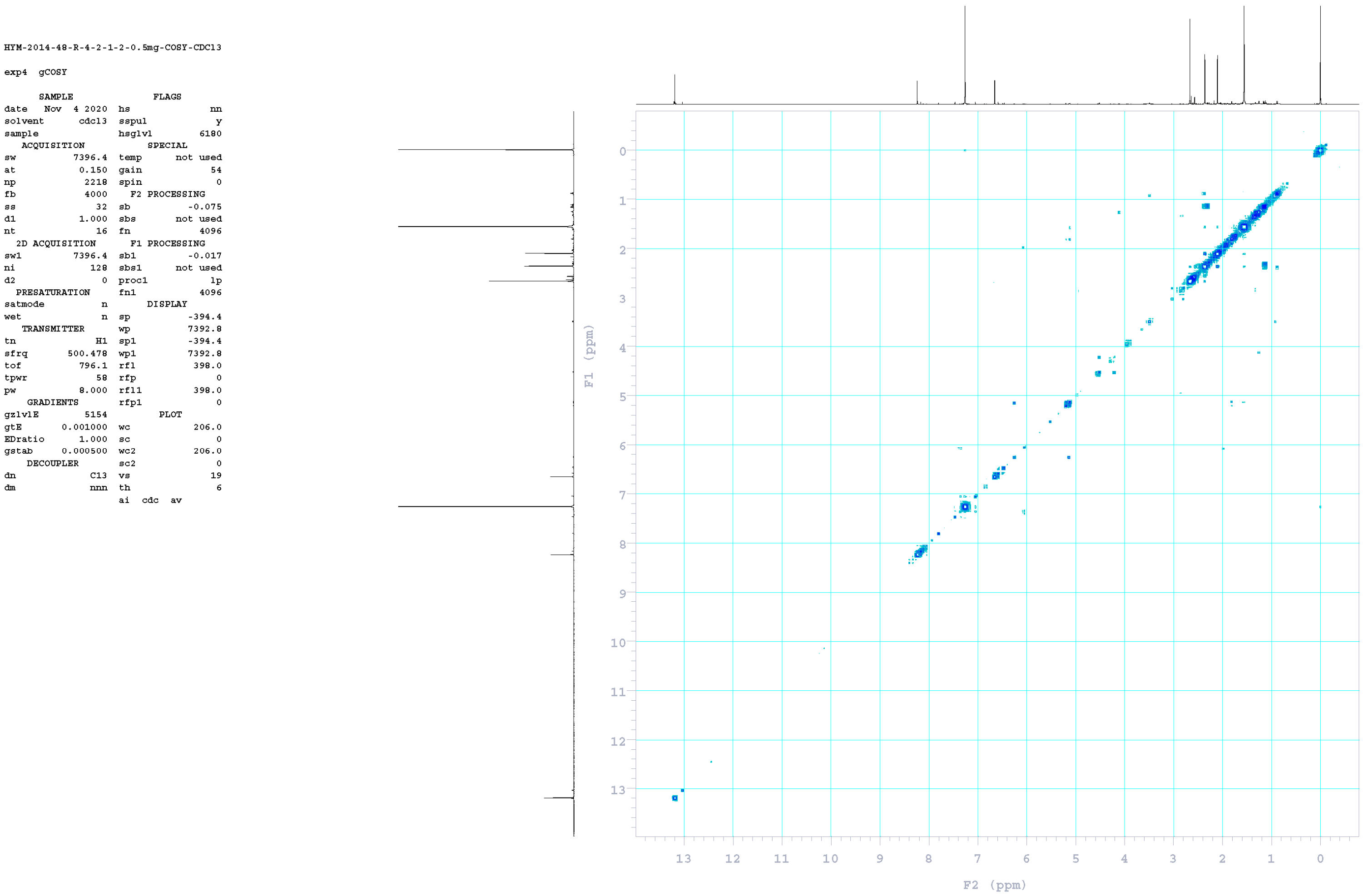

Figure S94. HSQC spectrum of **20** (measured in $CDCl_3$, 500 MHz).

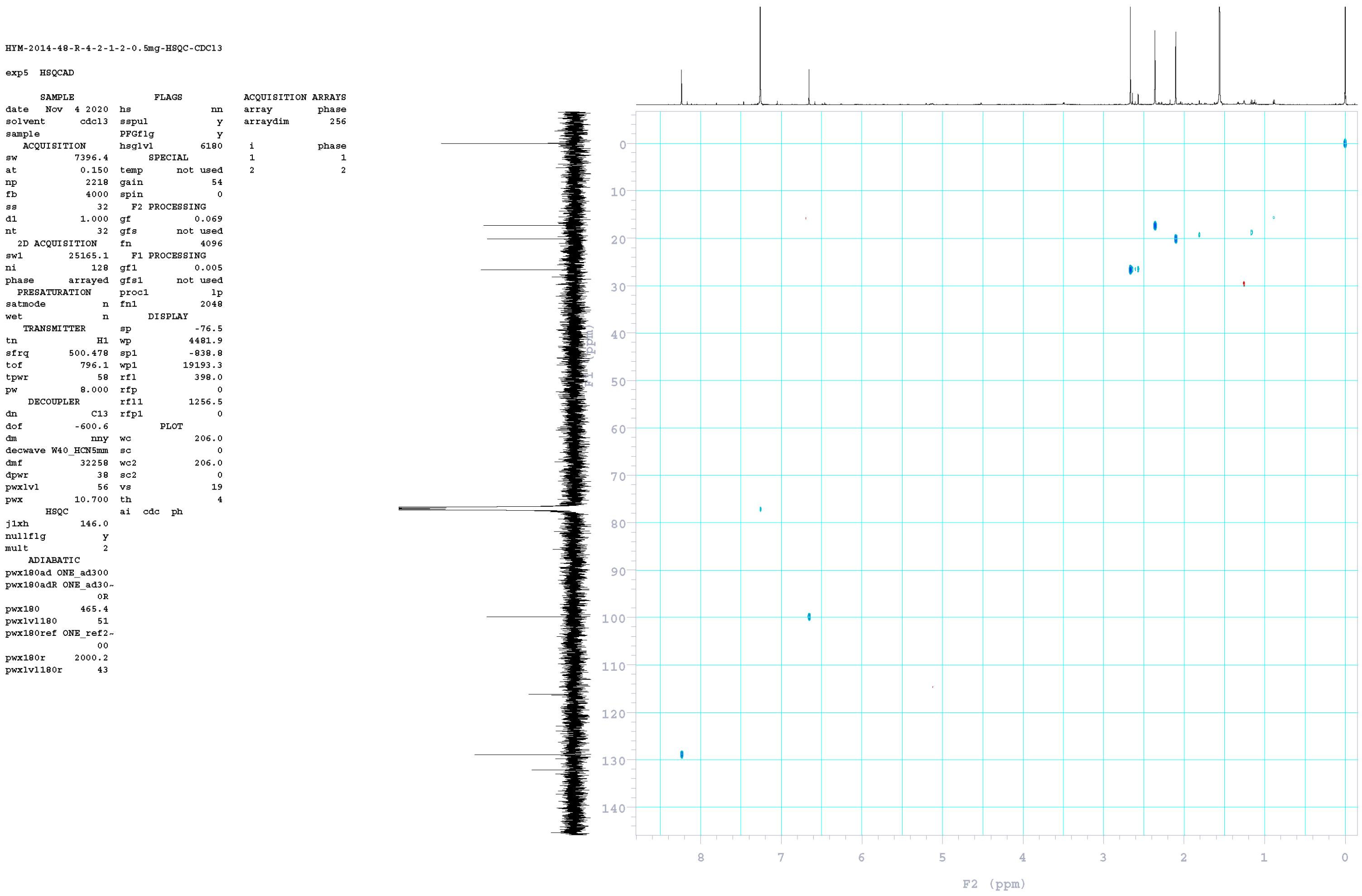

Figure S95. HMBC spectrum of **20** (measured in $CDCl_3$, 500 MHz).

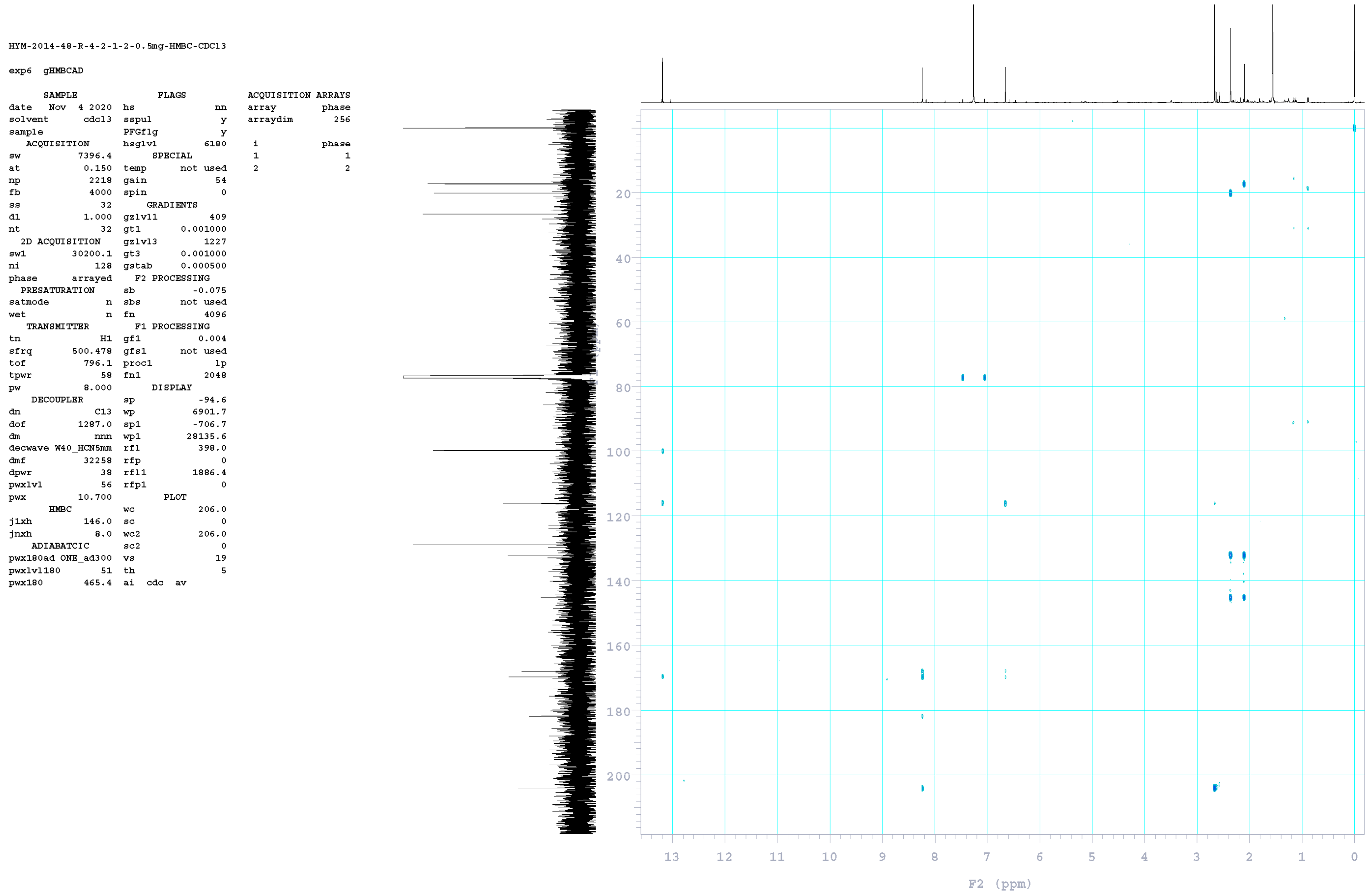

Figure S96. NOESY spectrum of **20** (measured in $CDCl_3$, 500 MHz).

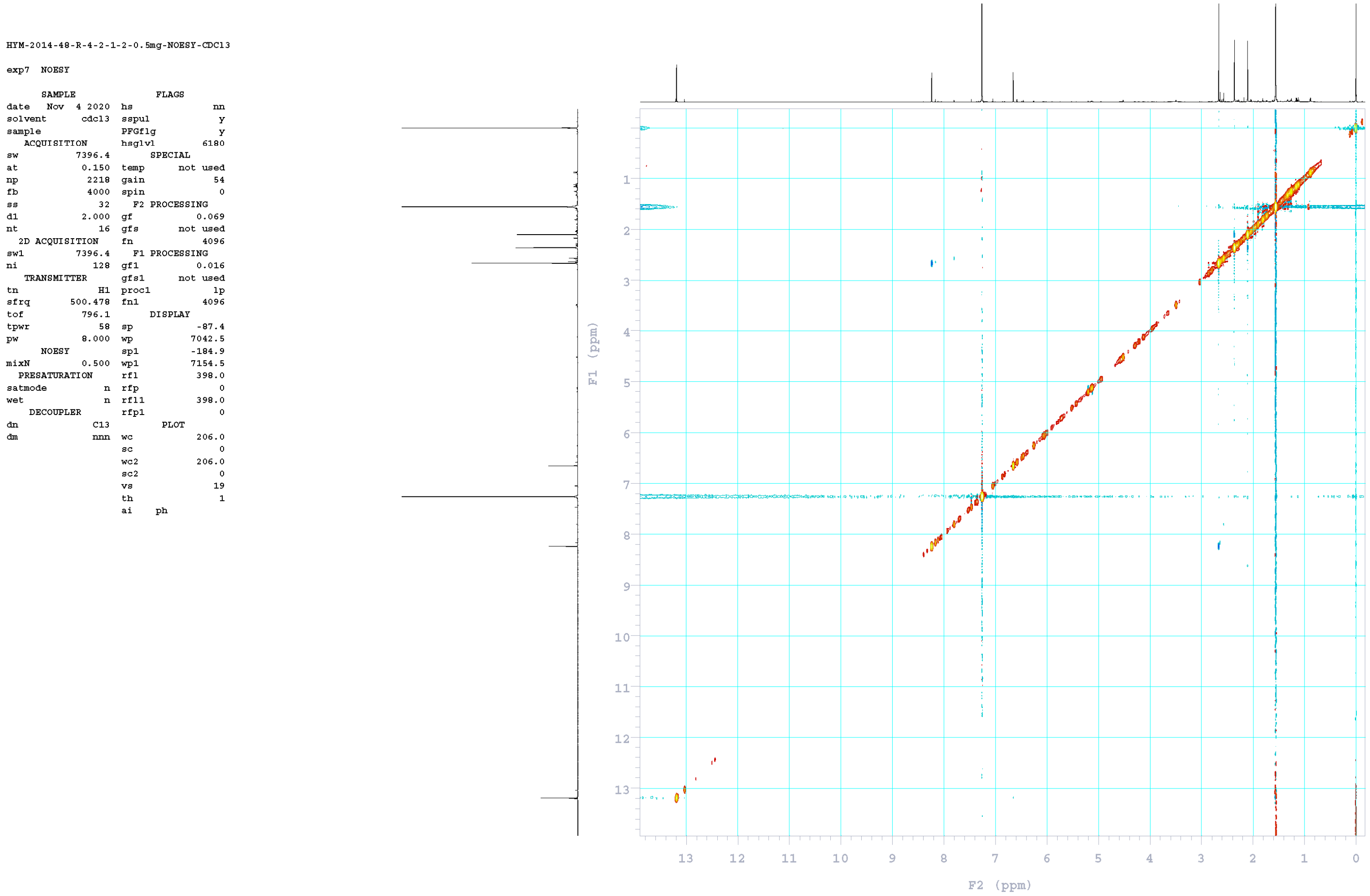

Figure S97. ^{1}H NMR spectrum of **28** (measured in $CDCl_3$, 500 MHz).

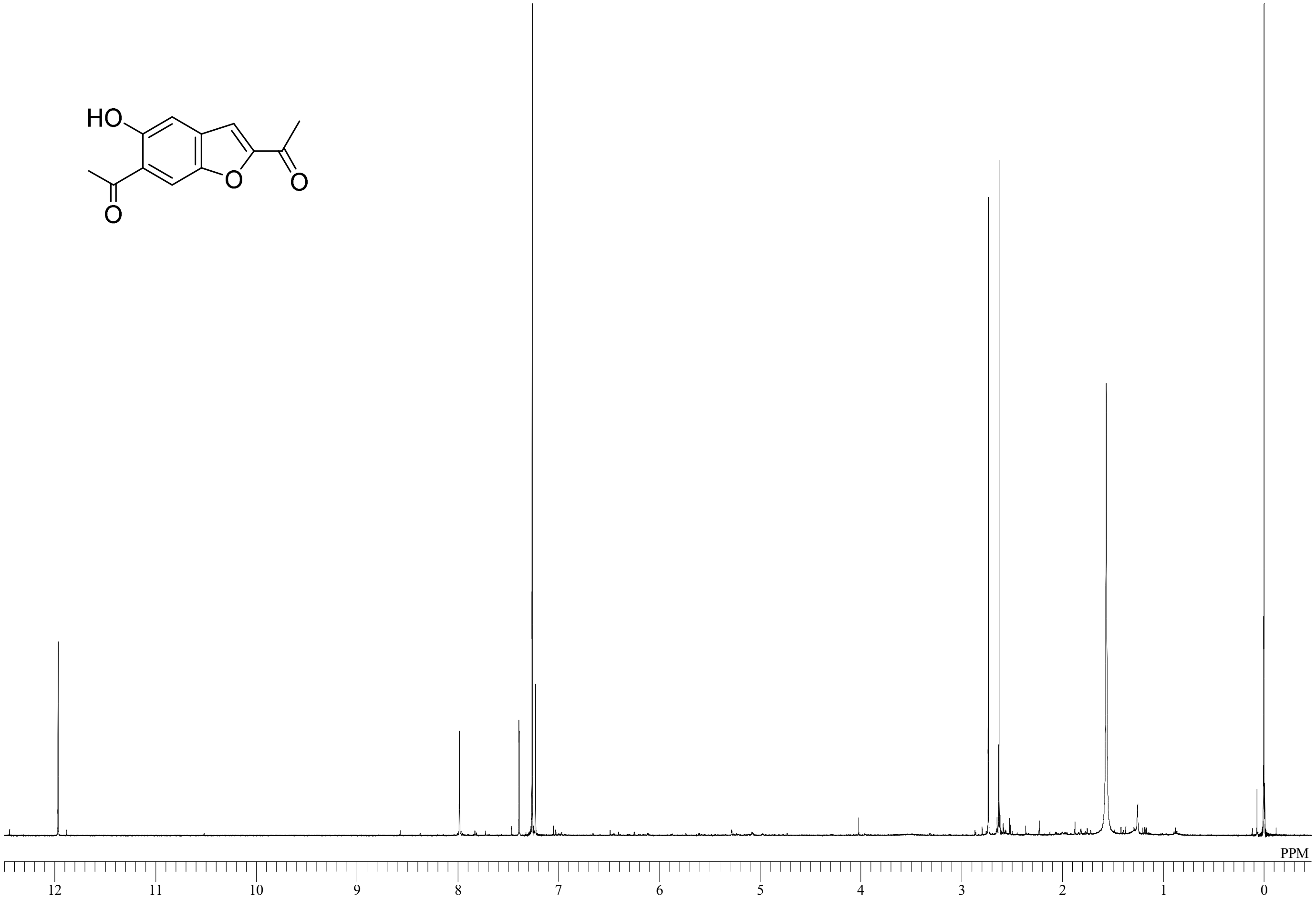

Figure S98. ^{13}C NMR spectrum of **28** (measured in $CDCl_3$, 126 MHz).

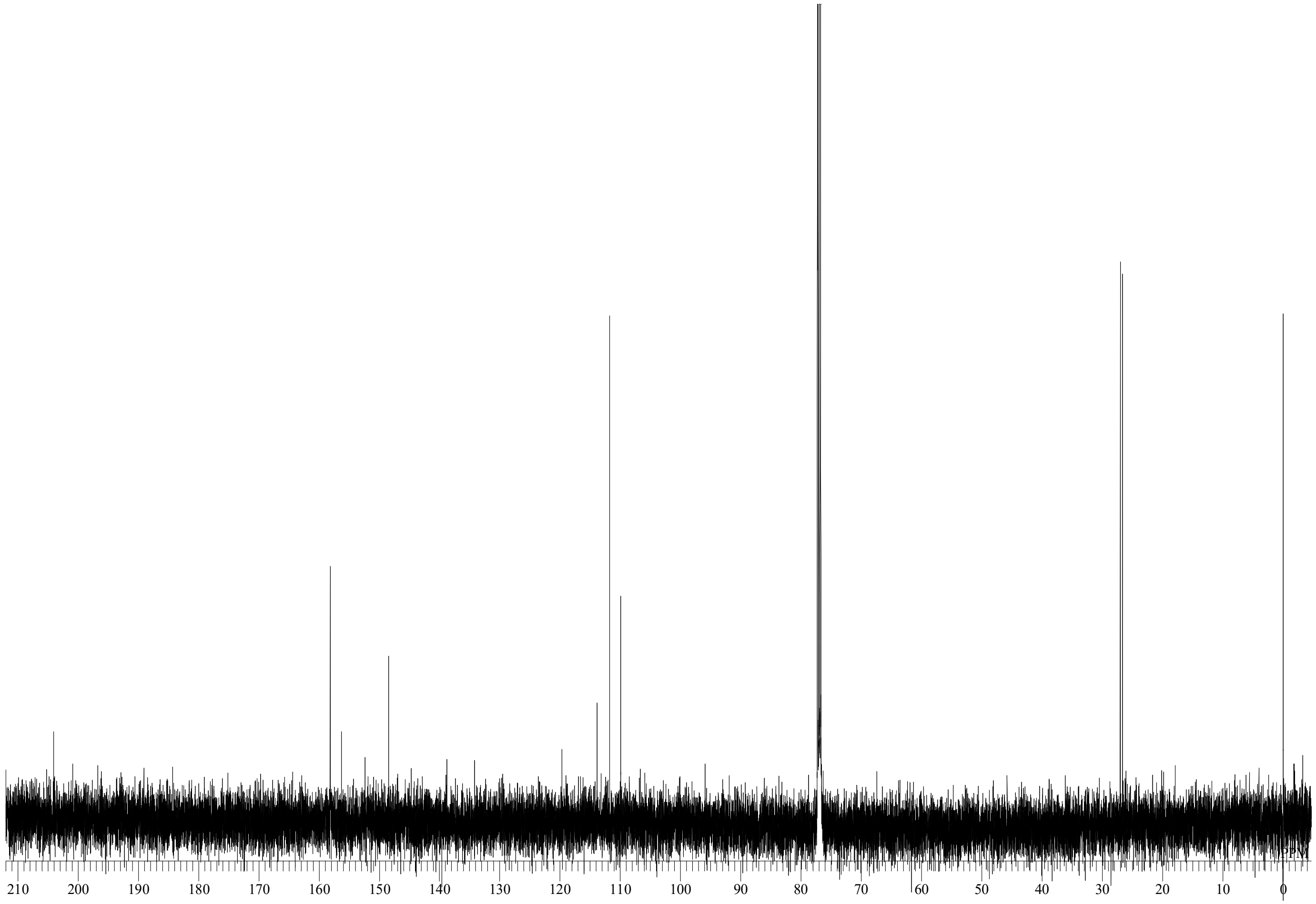

Figure S99. ^{1}H-^{1}H COSY spectrum of **28** (measured in $CDCl_3$, 500 MHz).

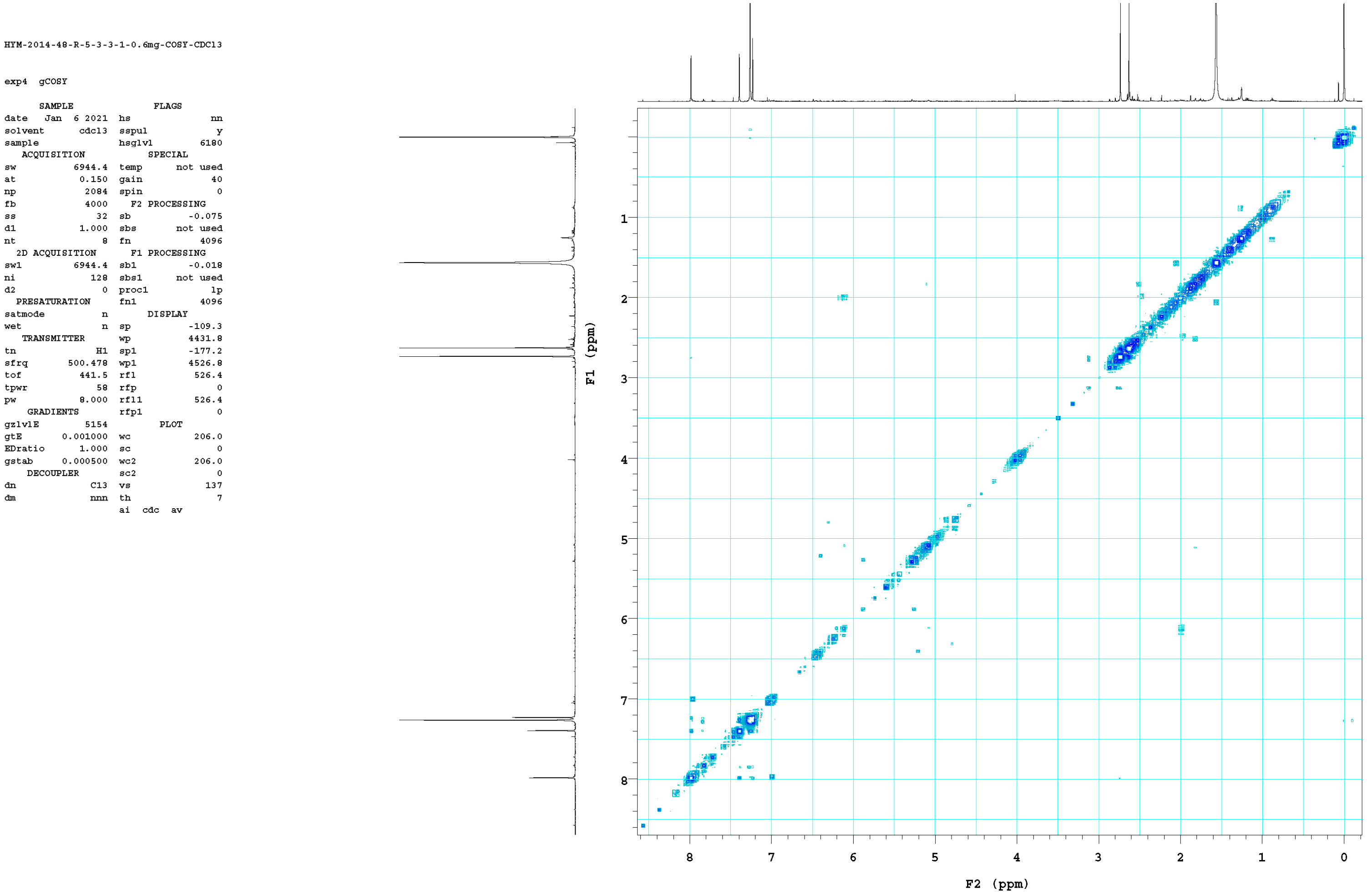

Figure S100. HSQC spectrum of **28** (measured in $CDCl_3$, 500 MHz).

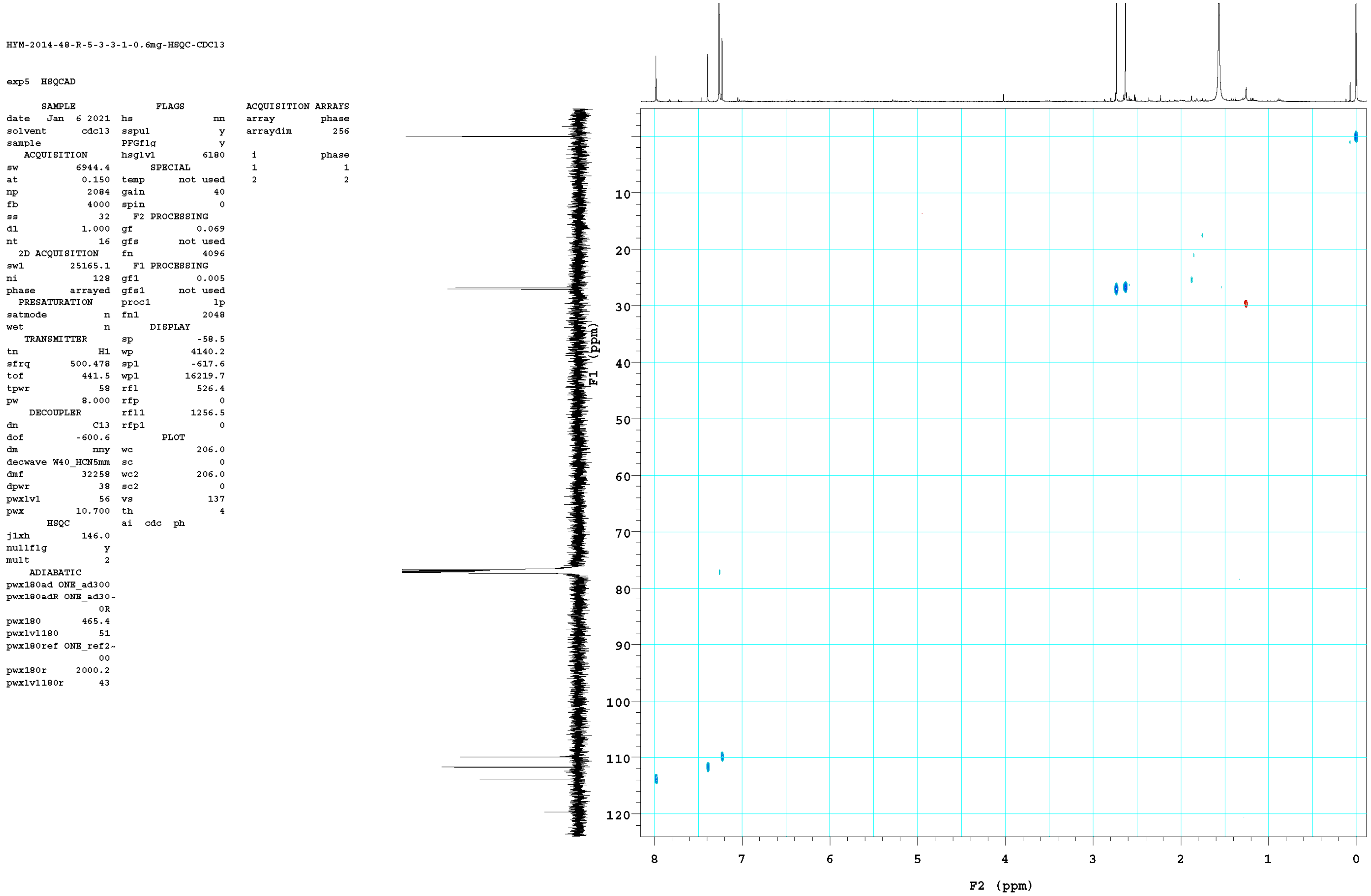

Figure S101. HMBC spectrum of **28** (measured in $CDCl_3$, 500 MHz).

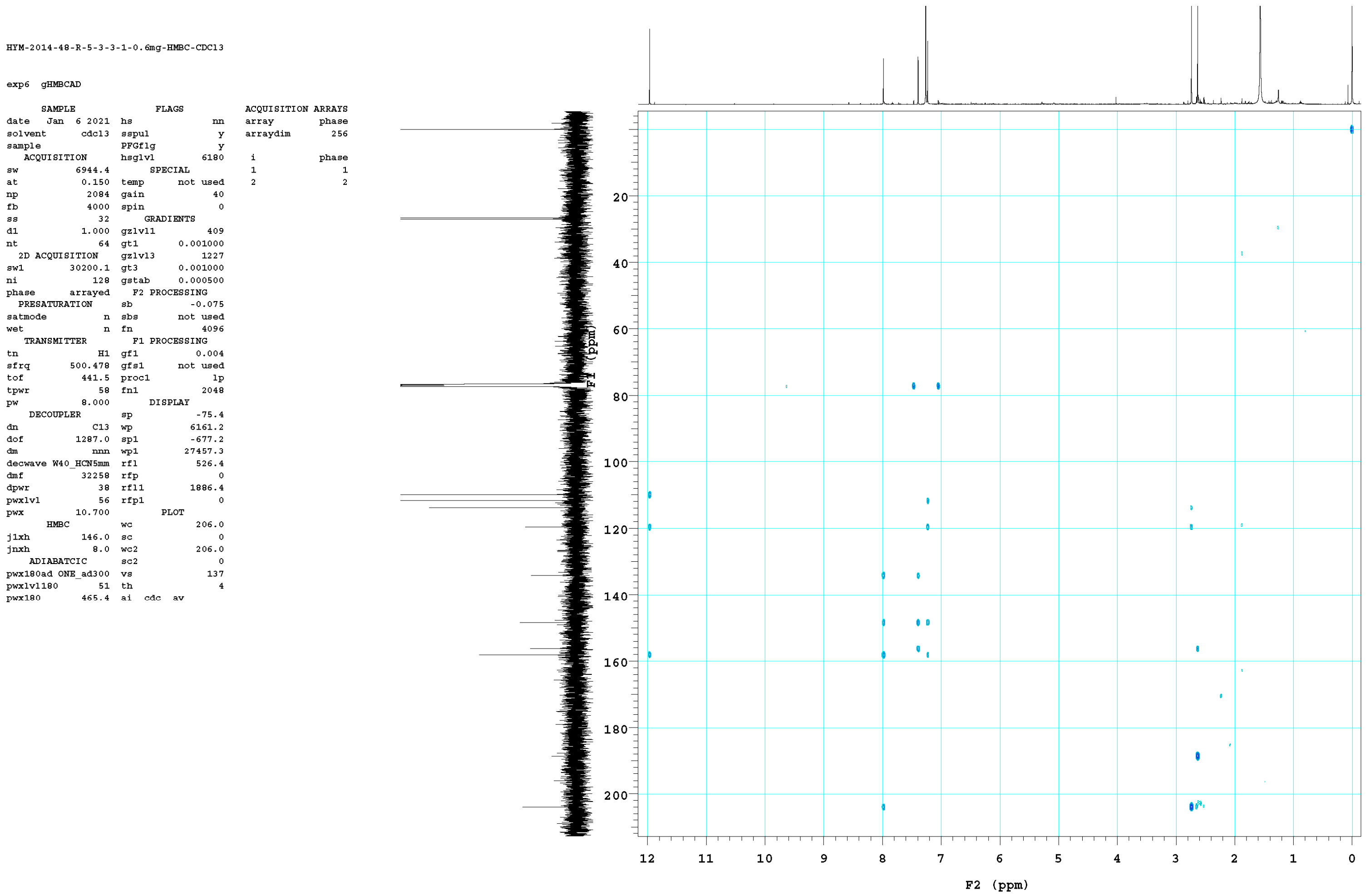

Figure S102. NOESY spectrum of **28** (measured in $CDCl_3$, 500 MHz).

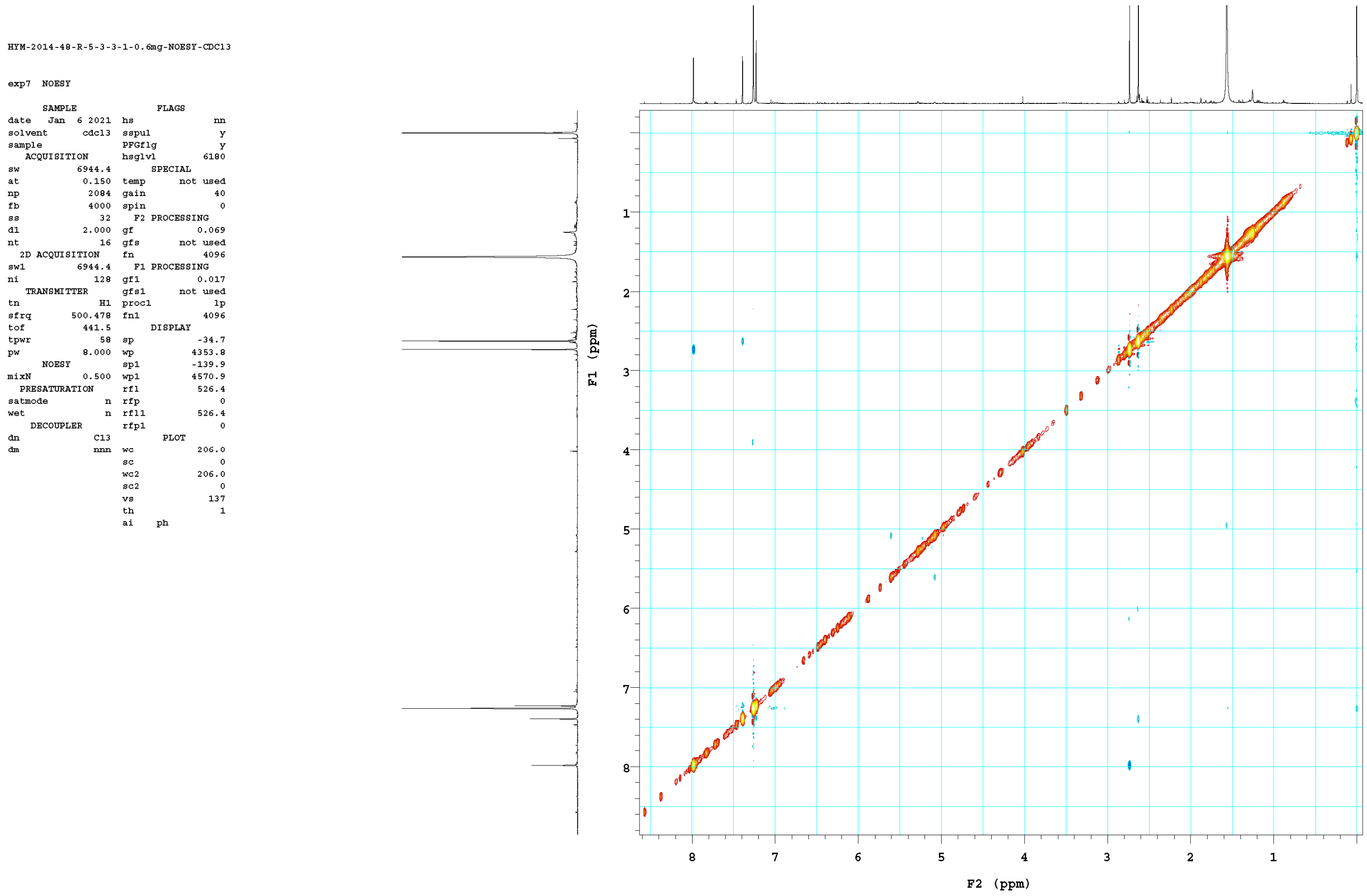

Supplement: Supplementary file 1 [file molecules-27-08856-s001.zip › molecules-2081772-supplementary.pdf]
